# Theoretical investigation of the anti-nitrosant mechanism of syringol and its derivatives

**DOI:** 10.1039/d5ra05587c

**Published:** 2025-09-17

**Authors:** Rahmanto Aryabraga Rusdipoetra, Hery Suwito, Ni Nyoman Tripuspaningsih, Kautsar Ul Haq

**Affiliations:** a Bioinformatic Research Group, Research Centre of Bio-Molecule Engineering (BIOME), Airlangga University Jl. Ir. H. Soekarno Mulyorejo Surabaya Indonesia; b Department of Chemistry, Faculty of Science and Technology, Airlangga University Jl. Ir. H. Soekarno Mulyorejo Surabaya Indonesia kautsar.ul.haq@fst.unair.ac.id; c Proteomic Research Group, Research Centre of Bio-Molecule Engineering (BIOME), Airlangga University Jl. Ir. H. Soekarno Mulyorejo Surabaya Indonesia

## Abstract

The depolymerization of lignin is known to yield syringol and its derivatives, namely 4-allylsyringol, 4-propenylsyringol, and 4-propylsyringol, which have been demonstrated to exhibit strong antioxidant properties against HOO˙ radicals. However, the potential of these compounds as antinitrosants remains largely unexplored. Reactive nitrogen species (RNS), such as NO˙ and NOO˙ radicals, are equally as harmful as reactive oxygen species (ROS), contributing to an increased risk of diseases such as diabetes and neurodegenerative disorders. In this study, we employed a density functional theory (DFT) approach using the QM-ORSA protocol to comprehensively investigate the antinitrosant activity of these compounds in both polar and non-polar media, as well as the underlying scavenging mechanisms that influence their activity. This protocol includes thermodynamic and kinetic parameter calculations, along with comparative analyses. The results suggest that NOO˙ radical scavenging by these compounds occurs at a diffusion-limited rate, with hydrogen atom transfer (HAT) being the predominant pathway. Among the investigated compounds, 4-propenylsyringol is predicted to exhibit the highest activity, with *k*_overall_ = 1.10 × 10^10^ M^−1^ s^−1^ (water) and 8.39 × 10^9^ M^−1^ s^−1^ (pentyl ethanoate). Furthermore, this study highlights the role of conjugated double bonds in enhancing the antinitrosant activity of syringol derivatives. Overall, all four investigated compounds demonstrate effective NOO˙ radical scavenging capabilities across different solvent environments.

## Abstract 2

Quantum insights into how syringol derivatives counter nitrosants.

## Graphical abstract

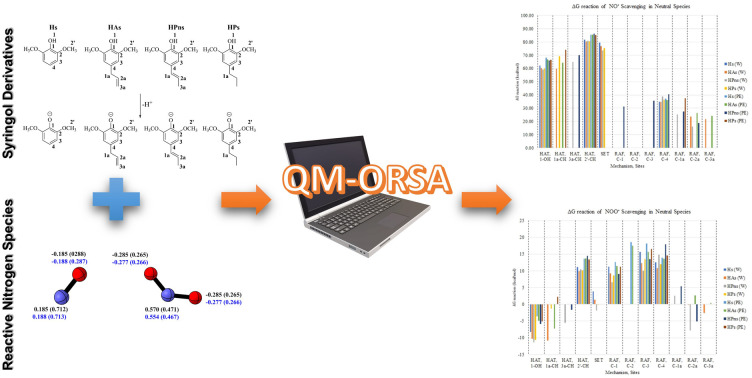

## Introduction

Reactive nitrogen species (RNS) are produced by the body as they play essential roles in signaling pathways and defense mechanisms against bacterial infections. Nitric oxide (NO˙) radicals, the primary RNS, are endogenously generated by the nitric oxide synthase (NOS) family, participating in various cellular processes such as cardiac muscle vasodilation, neurotransmission, apoptosis, and vascular homeostasis.^1^ However, RNS activity has also been linked to lipid peroxidation.^2^ This is because NO˙ radicals can react with superoxide anions at the diffusion-limited rate, forming peroxynitrite anions, which subsequently reduce enzymatic antioxidant levels.^3,4^ Another secondary RNS, NOO˙ radicals, has also been reported to exhibit cytotoxic effects, as it can alter tyrosine amino acid residues, leading to the formation of 3-nitrotyrosine (3-NT).^5^ Elevated 3-NT levels have been widely observed in patients with diabetes and neurodegenerative disorders such as Alzheimer's and Parkinson's diseases.^6,7^ This is because tyrosine residues in proteins and enzymes are particularly susceptible to nitration by NOO˙ radicals, resulting in increased α-synuclein aggregation,^8^ reduced MnSOD catalytic activity,^9^ and decreased dopamine levels.^10^ Additionally, exogenous sources of NOO˙ radicals, such as those generated from gasoline combustion, further increase the risk of elevated 3-NT concentrations. Therefore, the discovery of potent antioxidants capable of mitigating RNS activity is crucial for addressing these challenges.

Syringol, a key constituent of lignin, belongs to the polyphenol group and can be obtained through various lignin depolymerization methods, with yields reaching up to 21%.^11,12^ Moreover, its derivatives can be produced in significantly higher amounts.^13,14^ Lignin depolymerization encompasses all methods that cleave the β-O-4 aryl ether bonds, whether through thermal, chemical, or microbial processes.^15^ The primary objective is to generate valuable compounds and materials, which can offer up to six times higher economic value compared to their use as fuel alone.^16^ Several studies have reported that lignin depolymerization products exhibit potent antioxidant properties. Among lignin monomers, syringol has been identified as having the strongest antioxidant activity, even surpassing vitamin C in scavenging DPPH radicals, a common reactive nitrogen species (RNS) model.^17,18^ Furthermore, syringol derivatives bearing electron-donating substituents at the para position, such as allyl and alkyl groups, have demonstrated enhanced radical scavenging efficiency in similar assays. Our previous study, conducted using the DFT method, supports this argument, showing that 4-propylsyringol exhibits superior HOO˙ radical scavenging ability compared to lycopene and torulene in lipophilic environments.^19^ However, a comprehensive investigation into the overall activity and primary mechanisms underlying the RNS scavenging ability of syringol derivatives remains unexplored.

Computational methods have been widely employed to investigate the antioxidant properties of various compounds. One such method is QM-ORSA, developed by Galano *et al.*, which specifically evaluates antioxidant capacity by considering all possible scavenging mechanisms, namely hydrogen atom transfer (HAT), single electron transfer – proton transfer (SET), sequential proton loss electron transfer (SPLET), and radical adduct formation (RAF).^20^ Compared to other computational approaches, such as molecular orbital analysis,^21^ global descriptor analysis,^22^ and intrinsic reactivity analysis,^23^ the QM-ORSA method employs a thermodynamic- and kinetic-based approach. Consequently, this method can accurately determine rate constants, as demonstrated in the antioxidant potential assessments of ascorbic acid, melatonin, glutathione, trolox, and eight other antioxidant compounds.^24^

**Fig. 1:**
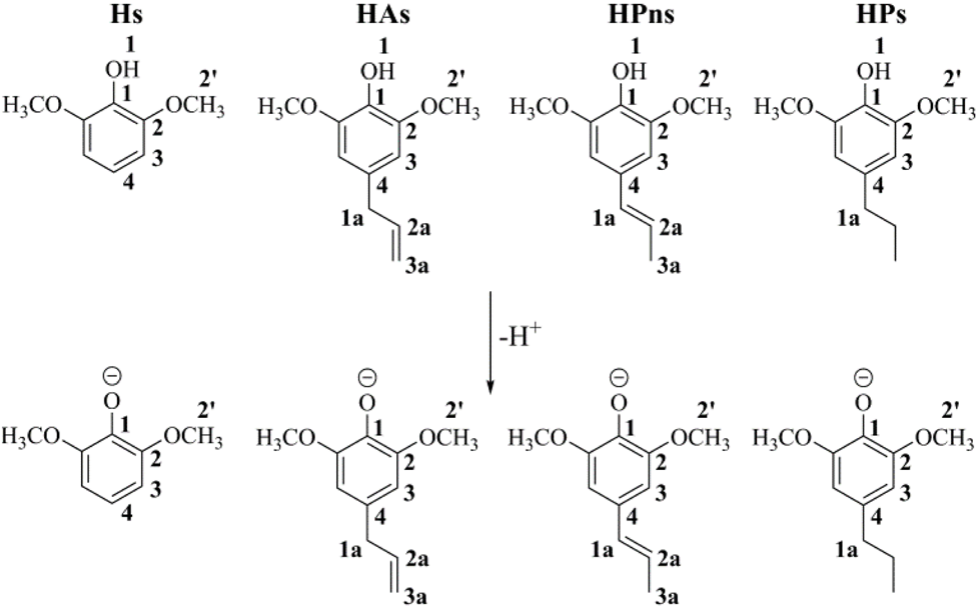
The structures of syringol derivatives and their protonated forms, along with atom numbering.

Despite these advantages, research on the potential of compounds in scavenging RNS using the QM-ORSA method remains limited. Over the past 15 years, we have found fewer than eight articles investigating this topic. Additionally, we discovered discrepancies in the selection of adduction sites on the NOO˙ radical in the RAF process. Cerón-Carrasco *et al.* were the first to conduct a similar antioxidant study, selecting the nitrogen atom of the NOO˙ radical as the reactive site in the RAF process.^25^ However, in recent years, many researchers have favored oxygen atom attack as the dominant model.^26–28^ Therefore, in this study, we applied the QM-ORSA protocol to investigate the antinitrosant potential of syringol derivatives against NO˙ and NOO˙ radicals. Syringol (Hs) and its three derivatives—4-allylsyringol (HAs), 4-propenylsyringol (HPns), and 4-propylsyringol (HPs)—were selected as the studied compounds. Alongside this research, we also aim to determine the suitable radical site on NOO˙ for the radical addition mechanism (Fig. 1).

## Computational methods

All calculations were performed using the Gaussian 16 software package under standard gas-phase conditions (1 atm, 298.15 K).^29^ The hybrid-meta GGA functional M06-2X and the polarized Pople basis set 6-311++G(d,p) were chosen as the DFT method and basis set, respectively, for all computational processes.^30,31^ To account for solvent effects, the solvation model based on electron density (SMD) was applied, with water and pentyl ethanoate selected as solvent models to simulate biological fluids and lipid bilayer environments, respectively.^32^ For radical structure optimizations, unrestricted calculations were employed. The optimized minimum energy structures and transition states were validated based on the presence or absence of an imaginary frequency (^i^*f* = 0 for minima; ^i^*f* = 1 for transition states). Furthermore, transition states were confirmed to connect the reactant and product complexes *via* Intrinsic Reaction Coordinate (IRC) analysis.

Four possible direct radical scavenging mechanisms for syringol derivatives are considered:

HAT (Hydrogen Atom Transfer)

1 AOH + R˙ → AO˙ + RH

SET (Single Electron Transfer – Proton Transfer)

2a AOH + R˙ → AO˙^+^ + R^−^

2b AO˙^+^ + R^−^ → AO˙ + RH

SPLET (Sequential Proton-Loss Electron Transfer)

3a AOH + R˙ → AO^−^ + RH˙^+^

3b AO^−^ + RH˙^+^ → AO˙ + RH

RAF (Radical Adduct Formation)

4 AOH + R˙ → [AO − R]˙

In this study, only the HAT and RAF mechanisms were modeled in pentyl ethanoate, as the SET and SPLET mechanisms involve charged species that do not spontaneously form in non-polar solvents.^33^ For the same reason, in pentyl ethanoate, this study only investigates the scavenging mechanisms of the neutral species of each syringol derivative. In contrast, all possible scavenging mechanisms of both neutral and anionic species were examined in aqueous environments. In the HAT mechanism investigation, only electron-rich O–H and sp^3^ C–H bonds were considered as hydrogen atom donors. Meanwhile, in the RAF mechanism, all unsaturated carbon atoms in each syringol derivative were investigated as potential radical adduct formation sites.

**Fig. 2:**
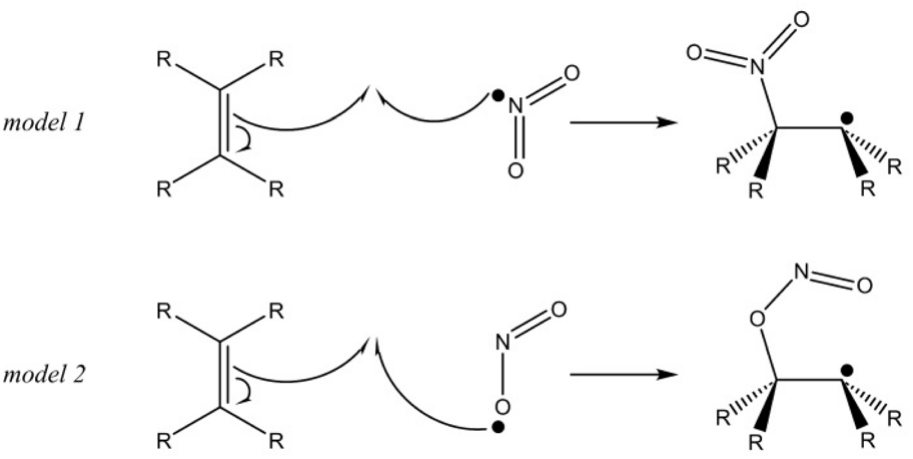
Possible RAF mechanism model in reaction with NOO˙ radical.

The exploration of NOO˙ radical adduction sites by syringol derivatives was conducted by comparing the thermodynamic feasibility of the reaction. As shown in Fig. 2, the sp^2^ carbon atoms can attack either the nitrogen (N) or oxygen (O) atoms of the NOO˙ radical. In this study, the attack on the oxygen atom, commonly used in the RAF mechanism model, is referred to as Model 1, while the attack on the nitrogen atom is designated as Model 2.

The relative free energy values of all reactions, initially calculated under standard gas-phase conditions, must be converted to the standard solution phase (1 M, 298.15 K) using eqn (5). This conversion applies exclusively to bimolecular reactions.^34^ Subsequently, the values were further corrected using eqn (6) to account for solvent cage effects, which influence entropy changes during the reaction.^35^ In this equation, *n* represents the number of reactants in the reaction.

5 Δ*G*^°1M^_29815K_ = Δ*G*^°1atm^_29815K_ − *RT* ln(24.5)

6 Δ*G*^FV1M^_29815K_ = Δ*G*^1M^_29815K_ − *RT*{ln[*n*10^2*n*−2^] – (*n* – 1)}

Following the QM-ORSA protocol, thermodynamic analysis aims to coarsely select potential scavenging sites that may contribute to the antinitrosant activity of syringol derivatives. Only sites with spontaneous scavenging activity (Δ*G*° < 0 kcal mol^−1^) were further investigated in the kinetic analysis. In this study, the kinetic analysis began by calculating the activation free energy, which was required to determine the reaction rate constant using the Eyring equation (eqn (7)).^36–41^7
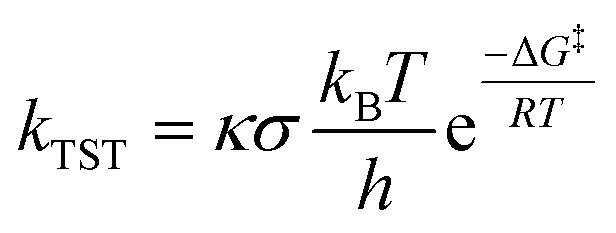
where *κ* is the Eckart quantum tunneling correction calculated using the Eyringpy program.^42^ Meanwhile, *σ* represents the symmetry factor. In the HAT and RAF mechanisms, the activation free energy is obtained after identifying the transition state structure. In contrast, for the electron transfer mechanism, the Δ*G*^‡^ value is determined using Marcus theory (eqn (8)).^43,44^8
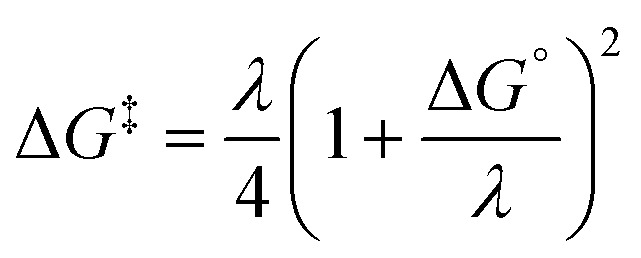
here *λ* is the reorganization energy, defined as the difference between Δ*G*° and Δ*E*° (the electronic energy difference between the vertical reactant and product states). If the calculated rate constant exceeds the diffusion limit (*k* > 10^8^ M^−1^ s^−1^), corrections are applied using three equations: the Collins-Kimball theory (eqn (9)),^45^ the Smoluchowski equation (eqn (10)),^46^ and the Stokes–Einstein equation (eqn (11)).^47,48^9
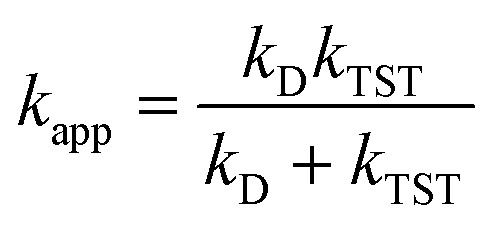

10 *k*_D_ = 4π*R*_AB_*D*_AB_*N*_A_

11
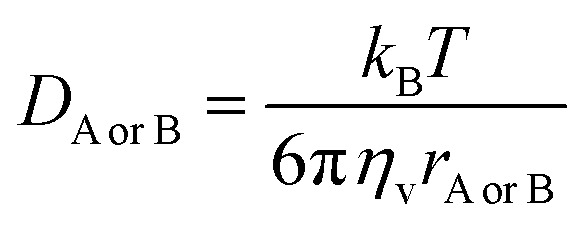
where *k*_D_ is steady state Smoluchowski rate constant, *R*_AB_ is reaction distance, *D*_AB_ is total mutual diffusion coefficient of free radical (A) and syringol derivatives (B), *N*_A_ is Avogadro number, *η* is viscocity of solvent, and *r* is radius of solute. For HT and RAF mechanisms, reaction distance was measured from two heavy atoms in transition states which were involved in transfer process. Meanwhile, reaction distance in SET and SPLET mechanisms was simply total radius of solutes.

In pentyl ethanoate solvent, the *k*_overall_ value is the sum of *k*_app_ values from all spontaneous scavenging sites of each syringol derivative, as described in eqn (12). In aqueous solvent, the *k*_overall_ value is obtained by summing the *k*_app_ values corrected for species availability (^M^*f*) at physiological pH, as shown in eqn (13)–(15).12
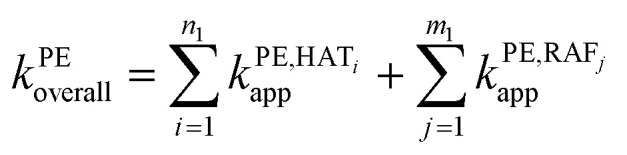
13
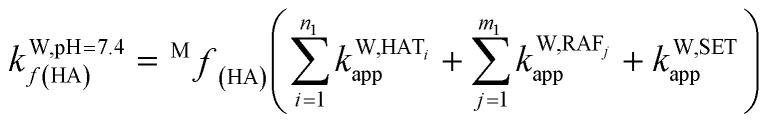
14
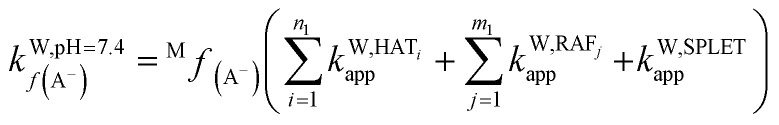
15
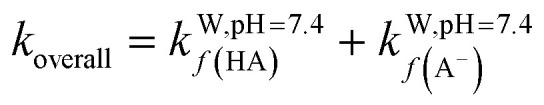


## Results & discussions

Based on previous studies, the four syringol derivatives belong to the group of weak monoprotic acids (HA).^19,49^ Under physiological conditions, only approximately 0.23–0.48% of these compounds are deprotonated into phenolate ions (A^−^). Although this percentage seems relatively small, it still contributes to free radical scavenging that cannot be ignored.^50^ This is because anionic species generally exhibit very high electron transfer rates.

**Fig. 3:**
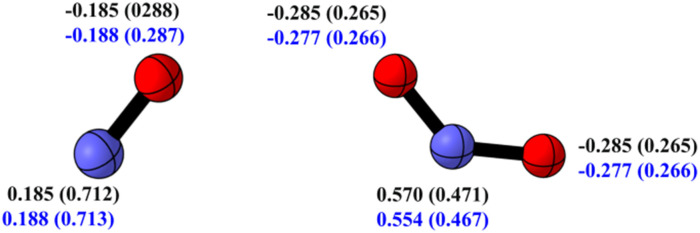
Natural charge and spin population (in parantheses) of NO˙ (left) and NOO˙ (right) radical in water (black) and pentyl ethanoate (blue) at the M06-2X/6-311++G(d,p) level of theory.

In scavenging RNS, the investigated syringol derivatives possess more than 17 possible reaction sites, which are described in detail in Table 1 and 2. These tables also present the calculated Gibbs free energy of reaction (Δ*G*°) for each scavenging site of all compounds. In Table 2, the Δ*G*° values listed for the RAF sites have considered the modeling analysis of the NOO˙ radical adduct site. Among the two reaction models commonly discussed in the scientific community, the less frequently used model turns out to be more thermodynamically favorable, with an average Δ*G*° value 3.5 kcal mol^−1^ lower (see SI Table S1). In this model, the nitrogen atom of the NOO˙ radical is the site attacked by the sp^2^ carbon atom. Additional electron spin population analysis on the NOO˙ radical, as depicted in Fig. 3, reveals that the nitrogen atom is more electropositive than the oxygen atom, yet it has the highest spin density. These results are consistent with the findings of Nash *et al.* in 2012, who explored the reactivity of the NOO˙ radical with nitrones.^51^ The high spin density indicates that the unpaired electron is localized on the nitrogen atom, making it more reactive in forming adducts with syringol derivatives.^52,53^

**Table 1:** Relative Gibbs free energy of reaction (Δ*G*°) in kcal mol^−1^ at 298.15 K for all possible NO˙ scavenging sites. HA: neutral state, A^−^: anionic state, PE: pentyl ethanoate, W: water

Mechanism, sites	HA (PE)	HA (W)	A^−^ (W)	HA (PE)	HA (W)	A^−^ (W)
	**Hs**			**HPs**		
HAT, 1-OH	68.21	62.29		66.65	59.97	
HAT, 1a-CH				74.09	69.20	66.09
HAT, 2′-CH	85.40	81.73	79.27	85.17	80.66	78.75
SET		79.46			75.46	
SPLET			48.83			45.96
RAF, C-1	—	—	—	—	—	—
RAF, C-2	—	—	—	—	—	—
RAF, C-3	—	—	—	—	—	—
RAF, C-4	37.25	36.64	19.59	37.68	36.41	17.92

	**HAs**			**HPns**		
HAT, 1-OH	66.90	60.20		65.96	59.17	
HAT, 1a-CH	64.49	59.68	56.08			
HAT, 3a-CH				70.18	65.01	61.95
HAT, 2′-CH	85.48	80.44	78.65	86.34	81.02	79.25
SET		76.81			73.64	
SPLET			46.64			46.15
RAF, C-1	—	—	—	31.38	29.93	—
RAF, C-2	—	—	—	—	—	—
RAF, C-3	—	—	—	35.75	—	—
RAF, C-4	36.22	34.86	15.31	40.62	38.97	—
RAF, C-1a				27.61	25.31	23.58
RAF, C-2a	26.51	23.87	22.90	18.96	16.25	10.56
RAF, C-3a	24.42	21.98	21.47			

**Table 2:** Relative Gibbs free energy of reaction (Δ*G*°) in kcal mol^−1^ at 298.15 K for all possible NOO˙ scavenging sites. HA: neutral state, A^−^: anionic state, PE: pentyl ethanoate, W: water

Mechanism, sites	HA (PE)	HA (W)	A^−^ (W)	HA (PE)	HA (W)	A^−^ (W)
	**Hs**			**HPs**		
HAT, 1-OH	−3.60	−8.27		−5.16	−10.59	
HAT, 1a-CH				2.28	−1.36	−4.47
HAT, 2′-CH	13.59	11.17	8.71	13.36	10.10	8.19
SET		3.9			−0.02	
SPLET			−26.65			−29.51
RAF, C-1	12.65	11.24	—	11.27	8.62	—
RAF, C-2	18.56	—	—	—	—	—
RAF, C-3	18.18	15.68	—	16.59	13.53	—
RAF, C-4	13.91	12.57	—	14.59	12.04	—

	**HAs**			**HPns**		
HAT, 1-OH	−4.92	−10.36		−5.86	−11.40	
HAT, 1a-CH	−7.32	−10.88	−14.48			
HAT, 3a-CH				−1.63	−5.55	−8.61
HAT, 2′-CH	13.67	9.88	8.09	14.53	10.46	8.69
SET		1.34			−1.84	
SPLET			−28.83			−29.33
RAF, C-1	11.42	9.23	—	9.04	6.55	—
RAF, C-2	17.54	—	—	—	—	—
RAF, C-3	15.69	12.30	—	13.53	10.04	5.68
RAF, C-4	13.58	10.85	—	17.96	14.79	—
RAF, C-1a				5.35	2.52	0.05
RAF, C-2a	2.68	−0.19	−2.13	−5.17	−7.86	−13.75
RAF, C-3a	0.36	−2.63	−3.07			

At the RAF site, not all Δ*G*° values could be obtained, as some of these reaction sites appear unlikely to form products. This interpretation was derived from potential energy surface (PES) scans of these reactions. The energy profile was constructed by recording the complex structure's energy at each change in the N(RNS)–C(Aromatic) bond length during the reaction process. The profile indicates that: (1) the reaction products have a higher relative energy than the reactant complex; (2) no stable minimum was found for the product structure, as the potential energy continued to increase even after the product geometry was formed.

It appears that all syringol derivatives are incapable of scavenging the NO˙ radical in both polar and non-polar environments. Syringic acid, a syringol derivative with an electron-withdrawing group (EWG) substituent, also experiences a similar phenomenon, indicating that the type of substituent does not influence the non-spontaneity of the NO˙ radical scavenging reaction.^28^ This difficulty arises due to the inherent stability of this radical when interacting with neutral compounds.^54^ Pérez-González, *et al.*, further stated that this radical does not actively attack macromolecules in the body directly.^55^ Instead, oxidative damage in the body tends to occur due to the formation of its radical derivatives, such as peroxynitrite ions and the NOO˙ radical.^56,57^

On the other hand, in an aqueous environment, HPns and HPs compounds can spontaneously scavenge the NOO˙ radical through the HAT, SET, SPLET, and RAF mechanisms. Meanwhile, the HAs compound can scavenge this radical through the HAT, SPLET, and RAF mechanisms only. In contrast, Hs exhibits spontaneous scavenging *via* the HAT and SPLET pathways. Unlike our previous findings,^19^ electron transfer to the NOO˙ radical exhibits a relatively lower Δ*G*° compared to the HOO˙ radical. Furthermore, the SPLET mechanism demonstrates a more spontaneous reaction energy than the HAT mechanism, which is typically the most favorable pathway when antioxidants scavenge the HOO˙ radical. A similar result was also observed in other antioxidants with different core structures, such as stilbenes,^27^ indicating that the spontaneity of the electron transfer mechanism is primarily driven by the intrinsic properties of the NOO˙ radical itself.

In a non-polar environment, only the HPns compound exhibits both HAT and RAF mechanisms as spontaneous pathways for scavenging the NOO˙ radical. Meanwhile, the other three compounds exhibit only the HAT mechanism. This phenomenon is related to the position of the double bond in the HPns side group, which influences the stability of the radical in the reaction product. In exergonic RAF product sites, the unpaired electron is localized on the C-1a atom, allowing resonance with the aromatic ring. Consequently, the resulting adduct is more stable. However, this stability is still insufficient to render the RAF mechanism more spontaneous than the HAT mechanism at the 1-OH site.

**Fig. 4:**
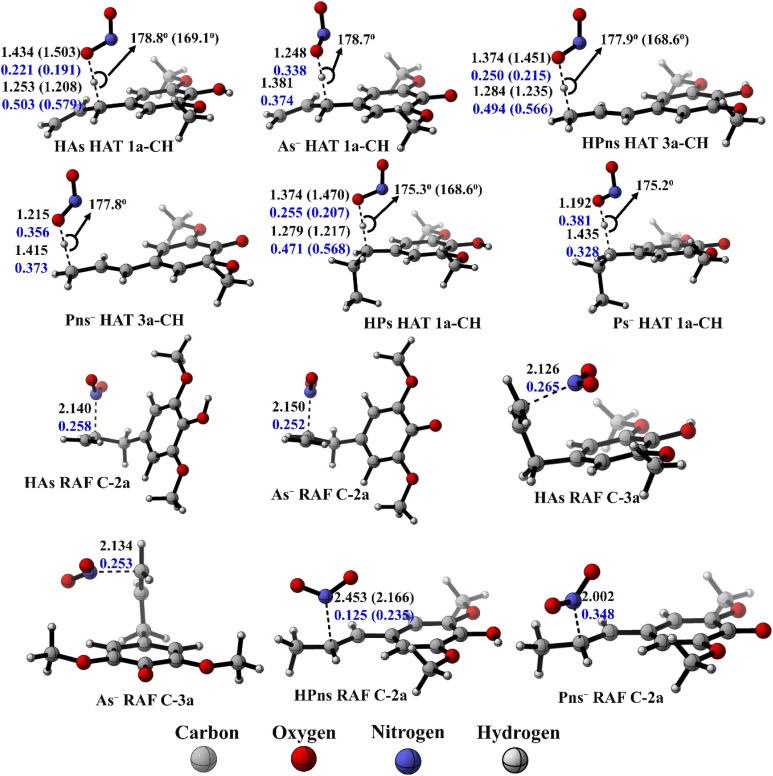
Transition state structures of the HT and RAF mechanism in each syringol derivatives, along with bond lengths (in black), bond angles (in black), and Wiberg bond orders (in blue) in aqueous solvent (pentyl ethanoate).

Based on thermodynamic analysis, only the NOO˙ radical scavenging mechanisms were further examined in the kinetic analysis. The calculated activation free energies and other kinetic parameters are summarized in Table 3. As previously explained, the identification of transition structures is crucial for determining the activation free energy in HAT and RAF mechanisms. The validated transition structures are compiled in Fig. 4. During this search, we were unable to identify the transition structure for hydrogen transfer by the 1-OH atom in the solvated phase, despite successfully obtaining it in the gas phase. However, during re-optimization under solvation conditions, the O–H bond distance in the phenolic group continued to increase until it resulted in a product complex. Consequently, a reaction energy diagram analysis was conducted by tracking the potential energy during the hydrogen transfer process from the phenolic group to the NOO˙ radical. The energy diagram indicates that the energy of the complex structure gradually decreases without forming a barrier, which suggests the absence of an activation energy for the reaction (see SI Table S4). Therefore, we conclude that all hydrogen transfer reactions involving the 1-OH atom are barrierless in all solvents, meaning that product formation is solely influenced by the diffusion rates of the two reactants.

**Table 3:** Activation Gibbs energy of reaction (Δ*G*^‡^) in kcal mol^−1^ at 298.15 K, quantum tunneling correction, and apparent rate constant for spontaneous NOO˙ scavenging sites. HA: neutral state, A^−^: anionic state, PE: pentyl ethanoate, W: water

Mechanism, sites	HA (PE)			HA (W)			A^−^ (W)		
	ΔG^‡^ (kcal mol^−1^)	*κ*	*k*_app_ (M^−1^ s^−1^)	Δ*G*^‡^ (kcal mol^−1^)	*κ*	*k*_app_ (M^−1^ s^−1^)	Δ*G*^‡^ (kcal mol^−1^)	*κ*	*k*_app_ (M^−1^ s^−1^)
**Hs**									
HAT, 1-OH	Barrierless	—	7.99 × 10^9^	Barrierless	—	7.75 × 10^9^			
SPLET							0.16	31.0^a^	7.78 × 10^9^

**HAs**									
HAT, 1-OH	Barrierless	—	8.15 × 10^9^	Barrierless	—	7.88 × 10^9^			
HAT, 1a-CH	21.93	1.8	1.97 × 10^−2^	14.07	6.5	4.12 × 10^4^	19.64^b^	13.0	2.74 × 10^9^
SPLET							0.05	31.4^a^	7.78 × 10^9^
RAF, C-2a				11.74	1.4	2.27 × 10^5^	10.86	1.4	1.00 × 10^6^
RAF, C-3a				9.19	1.3	1.55 × 10^7^	7.84	1.3	1.43 × 10^8^

**HPns**									
HAT, 1-OH	Barrierless	—	8.14 × 10^9^	Barrierless	—	7.88 × 10^9^			
HAT, 3a-CH	16.74	2.2	2.34 × 10^2^	13.31	10.8	3.73 × 10^5^	24.32^b^	9.4	2.73 × 10^9^
SET				6.81	30.8^a^	6.17 × 10^8^			
SPLET							0.01	30.3^a^	7.94 × 10^9^
RAF, C-2a	7.37	1.1	2.52 × 10^8^	3.44	1.0	2.54 × 10^9^	16.19^b^	1.1	2.07 × 10^9^

**HPs**									
HAT, 1-OH	Barrierless	—	8.14 × 10^9^	Barrierless	—	8.07 × 10^9^			
HAT, 1a-CH	22.31	1.6	9.36 × 10^−3^	15.68	10.0	4.22 × 10^3^	25.10^b^	2.0	2.73 × 10^9^
SET				8.23	33.0^a^	6.02 × 10^7^			
SPLET							0.02	31.2^a^	7.92 × 10^9^

a Nuclear reorganization energy (*λ*).

b Gibbs free energy of reaction relative to reactant complex.

As shown in Table 3, hydrogen transfer *via* the phenolic group exhibits the fastest scavenging rate in HAs and HPs compounds, both in polar and non-polar environments. In Hs and HPns compounds, this site remains the fastest scavenging pathway in non-polar solvents. However, in aqueous solvents, it is no longer the fastest mechanism, as SPLET becomes dominant. Nevertheless, the anionic species of each syringol derivative are present only in small fractions at physiological pH. As a result, molar fraction correction on the *k*_app_ values indicates that the contribution of the SPLET mechanism becomes negligible. Instead, hydrogen atom transfer (HAT) *via* the phenolic group emerges as the most dominant scavenging mechanism across all compounds. These corrected values, along with the overall scavenging rate constants, are summarized in Table 4.

**Table 4:** The values of *k*_app_, ^M^*f* at physiological pH, *k*_f_ at physiological pH, *k*_overall_, and *Γ* for each available site in the investigated syringol derivatives. HA: Neutral species, A^−^: anionic species, PE: pentyl ethanoate solvent, W: aqueous solvent

Mechanism, sites		HA (PE)		(W)							
				HA				A^−^			
		*k*_app_ (M^−1^ s^−1^)	*Γ* (%)	*k*_app_ (M^−1^ s^−1^)	^M^ *f* (%)	*k*_f_ (M^−1^ s^−1^)	*Γ* (%)	*k*_app_ (M^−1^ s^−1^)	^M^ *f* (%)	*k*_f_ (M^−1^ s^−1^)	*Γ* (%)
Hs	HAT, 1-OH	7.99 × 10^9^	100.0	7.75 × 10^9^	99.74	7.73 × 10^9^	99.7				
	SPLET							7.78 × 10^9^	0.26	2.04 × 10^7^	0.3
	***k***_**overall**_	**7.99× 10** ^**9**^		**7.75 × 10** ^**9**^							
HAs	HAT, 1-OH	8.15 × 10^9^	100.0	7.88 × 10^9^	99.77	7.86 × 10^9^	99.5				
	HAT, 1a-CH	1.97 × 10^−2^	∼0.0	4.12 × 10^4^	99.77	4.11 × 10^4^	∼0.0	2.74 × 10^9^	0.23	6.12 × 10^6^	0.1
	SPLET					2.27 × 10^5^		7.78 × 10^9^	0.23	1.74 × 10^7^	0.2
	RAF, C-2a			2.27 × 10^5^	99.77	1.55 × 10^7^	∼0.0	1.00 × 10^6^	0.23	2.24 × 10^6^	∼0.0
	RAF, C-3a			1.55 × 10^7^	99.77		0.2	1.43 × 10^8^	0.23	3.19 × 10^5^	∼0.0
	***k***_**overall**_	**8.15× 10** ^**9**^		**7.90 × 10** ^**9**^							
HPns	HAT, 1-OH	8.14 × 10^9^	97.0	7.88 × 10^9^	99.52	7.85 × 10^9^	71.0				
	HAT, 3′-CH	2.34 × 10^2^	∼0.0	3.73 × 10^5^	99.52	3.71 × 10^5^	∼0.0	2.73 × 10^9^	0.48	1.31 × 10^7^	0.1
	SET			6.17 × 10^8^	99.52	6.15 × 10^8^	5.6				
	SPLET							7.94 × 10^9^	0.48	3.80 × 10^7^	0.3
	RAF, C-2a	2.52 × 10^8^	3.0	2.54 × 10^9^	99.52	2.52 × 10^9^	22.8	2.07 × 10^9^	0.48	1.14 × 10^7^	0.1
	***k***_**overall**_	**8.39× 10** ^**9**^		**1.10 × 10** ^**10**^							
HPs	HAT, 1-OH	8.14 × 10^9^	100.0	8.07 × 10^9^	99.77	8.06 × 10^9^	99.0				
	HAT, 1′-CH	9.36 × 10^−3^	∼0.0	4.22 × 10^3^	99.77	4.21 × 10^3^	∼0.0	2.73 × 10^9^	0.23	6.37 × 10^6^	0.1
	SET			6.02 × 10^7^	99.77	5.99 × 10^7^	0.7				
	SPLET							7.92 × 10^9^	0.23	1.85 × 10^7^	0.2
	***k***_**overall**_	**8.14 × 10** ^**9**^		**8.14 × 10** ^**9**^							

Based on Table 4, the reactivity trend of NOO˙ radical scavenging is as follows: HPns > HPs > HAs > Hs, which is in good agreement with the energy gap values of each compound (see SI S3). HPns is the syringol derivative with the highest NOO˙ radical scavenging activity (*k*_overall_ = 1.10 × 10^10^ M^−1^ s^−1^ [water] & 8.39 × 10^9^ M^−1^ s^−1^ [pentyl ethanoate]). Compared to HAs, which also possesses an aliphatic double bond, HPns exhibits 39.24% higher activity in aqueous environments and 2.94% higher activity in pentyl ethanoate. The difference in the position of the aliphatic double bond appears to influence the high rate constant of HPns, as the RAF mechanism at C-2a provides a significant scavenging contribution of up to 22.8%. Meanwhile, the RAF mechanism in HAs possesses a higher activation energy and therefore contributes only 0.2% at its best *via* the C-3a atom. These results suggest that the presence of a conjugated double bond provides an additional dominant pathway, thereby enhancing antinitrosant activity against the NOO˙ radical. However, this argument should be examined from another perspective. A study conducted by Lu *et al.*,^26,27^ on different core structures, such as isorhapontigenin, piceatannol, and ferulic acid, presents a contrasting view. Therefore, further investigation is required to determine whether the rapid RAF mechanism facilitated by the conjugated double bond also depends on the core structure of the antioxidant compound.

According to the study by Prütz *et al.*,^58^ the initiation of lipid peroxidation by the NOO˙ radical in an aqueous environment has a rate constant of *k* = 2.00 × 10^5^–10^6^ M^−1^ s^−1^. This information indicates that the four syringol derivatives exhibit antinitrosant activity at least 3875 times higher. Additionally, their scavenging activity is at least 155 times higher than the oxidative damage of cysteine (*k* = 5 × 10^7^ M^−1^ s^−1^);^59^ 267 times higher than the oxidative damage of tyrosine (*k* = 2.90 × 10^7^ M^−1^ s^−1^); 15 500 times higher than the oxidative damage of tryptophan (*k* = 5.00 × 10^5^ M^−1^ s^−1^);^60^ and 7750 times higher than the oxidative damage of nucleic acids (*k* ≈ 10^6^ M^−1^ s^−1^)^61^ by the same radical. Furthermore, the four syringol derivatives demonstrate superior antinitrosant potential in aqueous environments compared to several antioxidant compounds, including ascorbic acid (*k* = 1.80 × 10^7^ M^−1^ s^−1^),^62^ trolox (*k* < 10^5^ M^−1^ s^−1^),^63^ resorcinol (*k* = 3.8 × 10^8^ M^−1^ s^−1^),^64^ and syringic acid (*k* = 1.98 × 10^8^ M^−1^ s^−1^).^28^ Therefore, we conclude that the four syringol derivatives are effective antinitrosants in aqueous environments.

Meanwhile, among antioxidant compounds with known activity in non-polar solvents, only sinapic acid has been reported. The four investigated syringol derivatives exhibit significantly higher antinitrosant potential than sinapic acid. Additionally, almost all of the studied compounds display greater scavenging activity in non-polar environments than in polar ones. Therefore, it can be concluded that the four syringol derivatives are also effective NOO˙ radical scavengers in non-polar environments.

## Conclusions

The QM-ORSA method has been successfully applied to evaluate the antinitrosant potential of syringol (Hs) and its derivatives against NO˙ and NOO˙ radicals. The findings of this study indicate that all four compounds are effective NOO˙ radical scavengers in both polar and non-polar environments. Among the possible mechanisms, the HAT pathway was found to be the most dominant for NOO˙ radical scavenging in all compounds. Among the four derivatives, 4-propenylsyringol exhibited the highest antinitrosant activity.

Furthermore, the results revealed that the presence of a conjugated double bond enhances the antinitrosant activity of syringol derivatives, particularly in the RAF mechanism. In addition, this study successfully identified the RAF attack site on the NOO˙ radical, which was found to be the nitrogen atom. Therefore, we recommend selecting this atom when modeling the RAF mechanism in antinitrosant studies using similar computational methods.

## Author contributions

Rahmanto Aryabraga Rusdipoetra: investigation, formal analysis, visualization, writing – original draft, data curation. Hery Suwito: validation, writing – review & editing. Ni Nyoman Tri Puspaningsih: validation, writing–review & editing. Kautsar Ul Haq: conceptualization, methodology, formal analysis, supervision, project administration.

## Conflicts of interest

The authors declare that they have no known competing financial interests or personal relationships that could have appeared to influence the work reported in this paper.

## Supplementary Material

RA-015-D5RA05587C-s001

## Data Availability

The data supporting this article have been included as part of the SI. Supplementary information: Relative Gibbs energy (in Kcal mol^−1^) of RAF mechanism on studied model. Relative enthalpy of reaction (Δ*H*°) in kcal mol^−1^ at 298.15 K for all possible NO˙ and NOO˙ scavenging sites. HOMO, LUMO, and energy gap in eV of syringol derivatives in studied environment. Potential energy scan of 1-OH HAT mechanism on studied model. Intrinsic reaction coordinate of HAT and RAF mechanism in water. Intrinsic reaction coordinate of HAT and RAF mechanism in pentyl ethanoate. Cartesian coordinate and thermochemical values of all optimized dtationary points in water. Cartesian coordinate and thermochemical values of all optimized stationary points in pentyl ethanoate. See DOI: https://doi.org/10.1039/d5ra05587c.
