# Supplementary material for: Theoretical investigation of the anti-nitrosant mechanism of syringol and its derivatives
Source: RSC Adv. 2025 Sep 17;15(41):33936–45. doi: 10.1039/d5ra05587c (PMC12442234; doi:10.1039/d5ra05587c)
Supplement: RA-015-D5RA05587C-s001 [file RA-015-D5RA05587C-s001.pdf]

Supplementary Information (SI) for RSC Advances.
This journal is © The Royal Society of Chemistry 2025

Suplementary Materials

Theoretical investigation of the anti-nitrosant mechanism of syringol and its derivatives revealed another possible dominant pathway

Rahmanto Aryabraga Rusdipoetra[a], Hery Suwito[b], Ni Nyoman Tripuspaningsih[b,c], and Kautsar Ul Haq[a,b]*

Author Affiliations
[a]*Bioinformatic Research Group. Research Centre of Bio-Molecule Engineering (BIOME), Airlangga University. Jl. Ir. H. Soekarno Mulyorejo, Surabaya, Indonesia*
[b]*Department of Chemistry, Faculty of Science and Technology. Airlangga University. Jl. Ir. H. Soekarno Mulyorejo, Surabaya, Indonesia*
[c]*Proteomic Research Group, Research Centre of Bio-Molecule Engineering (BIOME), Airlangga University. Jl. Ir. H. Soekarno Mulyorejo, Surabaya, Indonesia*

Author Emails
** Corresponding author: kautsar.ul.haq@fst.unair.ac.id*

Table of Contents

1. Table S1: Relative Gibbs Energy (in Kcal/mol) of RAF Mechanism on Studied Model. HA: neutral state, A^-: anionic state, PE: pentyl ethanoate, W: water. 2
2. Table S2: Relative Enthalpy of reaction (ΔH°) in kcal/mol at 298.15 K for all possible $NO^•$ and $NOO^•$ scavenging sites. HA: neutral state, A^-: anionic state, PE: pentyl ethanoate, W: water. 3
3. Table S3: HOMO, LUMO, and Energy Gap in eV of syringol derivatives in studied environment. 4
4. Table S4: Potential Energy Scan of 1-OH HAT Mechanism on Studied Model. HA: neutral state, A^-: anionic state, PE: pentyl ethanoate, W: water. 5
5. Figure S5: Intrinsic Reaction Coordinate of HAT and RAF Mechanism in Water 9
6. Figure S6: Intrinsic Reaction Coordinate of HAT and RAF Mechanism in Pentyl Ethanoate 13
7. Table S7: Cartesian Coordinate and Thermochemical Values of all Optimized Stationary Points in Water 15
8. Table S8: Cartesian Coordinate and Thermochemical Values of all Optimized Stationary Points in Pentyl Ethanoate 69

1. **Table S1: Relative Gibbs Energy (in Kcal/mol) of RAF Mechanism on Studied Model. HA: neutral state, A^-: anionic state, PE: pentyl ethanoate, W: water.**

Compound	Mechanism. Sites	Model 1 (C sp^2---$ONO^•$)			Model 2 (C sp^2---$NOO^•$)		
		HA (PE)	HA (W)	A^- (W)	HA (PE)	HA (W)	A^- (W)
Hs	RAF, C-1	14.73	13.19	-	12.65	11.24	-
	RAF, C-2	19.55	18.43	-	18.56	-	-
	RAF, C-3	21.51	19.74	17.43	18.18	15.68	-
	RAF, C-4	17.50	16.85	-	13.91	12.57	-
HAs	RAF, C-1	13.18	11.82	-	11.42	9.23	-
	RAF, C-2	19.49	16.69	-	17.54	-	-
	RAF, C-3	19.29	16.86	-	15.69	12.30	-
	RAF, C-4	15.72	14.33	-	13.58	10.85	-
	RAF, C-2a	6.32	2.73	2.47	2.68	-0.19	-2.13
	RAF, C-3a	5.81	2.02	2.33	0.36	-2.63	-3.07
HPns	RAF, C-1	10.71	8.28	-	9.04	6.55	-
	RAF, C-2	20.93	19.02	-	-	-	-
	RAF, C-3	16.60	14.33	11.95	13.53	10.04	5.68
	RAF, C-4	20.89	18.77	-	17.96	14.79	-
	RAF, C-1a	9.84	7.78	6.35	5.35	2.52	0.05
	RAF, C-2a	-1.14	-3.02	-7.85	-5.17	-7.86	-13.75
HPs	RAF, C-1	13.64	11.54	-	11.27	8.62	-
	RAF, C-2	19.40	17.71	-	-	-	-
	RAF, C-3	19.43	17.37	-	16.59	13.53	-
	RAF, C-4	16.90	15.06	-	14.59	12.04	-

2. **Table S2: Relative Enthalpy of reaction (ΔH°) in kcal/mol at 298.15 K for all possible $NO^•$ and $NOO^•$ scavenging sites. HA: neutral state, A^-: anionic state, PE: pentyl ethanoate, W: water.**

	NO•						NOO•					
Mechanism, Sites	HA (PE)	HA (W)	A⁻ (W)	HA (PE)	HA (W)	A⁻ (W)	HA (PE)	HA (W)	A⁻ (W)	HA (PE)	HA (W)	A⁻ (W)
	Hs			HPs			Hs			HPs		
HAT, 1-OH	71.78	65.13		70.04	63.11		-0.60	-6.44		-2.33	-8.46	
HAT, 1a-CH				77.79	72.76	68.64				5.42	1.19	-1.92
HAT, 2'-CH	89.26	84.61	82.85	87.72	83.21	82.82	16.88	11.04	11.28	16.78	12.97	11.253
SET		81.67			77.90			5.87			2.11	
SPLET			51.35			48.38			-24.45			-27.42
RAF, C-1	-	-	-	-	-	-	4.98	2.47	-	3.85	1.15	-
RAF, C-2	-	-	-	-	-	-	11.31	-	-	-	-	-
RAF, C-3	-	-	-	-	-	-	11.13	7.45	-	8.60	4.99	-
RAF, C-4	31.51	29.83	10.18	31.16	29.33	10.56	6.80	3.85	-	6.55	3.37	-
	HAs			HPns			HAs			HPns		
HAT, 1-OH	68.95	63.66		68.66	61.71		-3.42	-7.91		-3.71	-9.86	
HAT, 1a-CH	68.00	62.82	59.02				-4.37	-8.74	-12.54			
HAT, 3a-CH				72.87	67.90	65.10				0.50	-3.67	-6.46
HAT, 2'-CH	89.40	84.49	82.74	89.36	84.67	83.05	17.03	12.91	11.17	16.98	13.11	11.48
SET		79.14			75.03			3.35			-0.76	
SPLET			49.35			48.41			-26.44			-27.39
RAF, C-1	-	-	-	24.27	22.64	-	4.04	1.47	-	0.32	-2.11	-
RAF, C-2	-	-	-			-	10.44	-	-	-	-	-
RAF, C-3	-	-	-	28.35		-	9.09	5.07	-	4.70	1.48	-1.88
RAF, C-4	30.33	28.26	8.67	34.09	32.40	-	6.05	2.88	-	9.94	7.31	-
RAF, C-1a				20.90	18.42	17.91				-1.64	-5.00	-6.76
RAF, C-2a	20.57	17.71	17.04	11.60	9.07	3.20	-4.63	-8.73	-9.58	-13.33	-16.57	-22.37
RAF, C-3a	18.92	16.39	16.03				-6.86	-10.91	-11.47			

3. Table S3: HOMO, LUMO, and Energy Gap in eV of syringol derivatives in studied environment.

Compound	Solvent	HOMO	LUMO	Energy Gap

Hs	Water	-7.358	0.065	7.423
	Pentyl Ethanoate	-7.118	0.015	7.133
HAs	Water	-7.203	0.022	7.223
	Pentyl Ethanoate	-6.970	0.023	6.993
HPns	Water	-6.874	-0.005	6.869
	Pentyl Ethanoate	-6.696	-0.014	6.682
HPs	Water	-7.158	0.008	7.166
	Pentyl Ethanoate	-6.916	-0.003	6.913

4. Table S4: Potential Energy Scan of 1-OH HAT Mechanism on Studied Model. HA: neutral state, A^-: anionic state, PE: pentyl ethanoate, W: water.

2a. Hs HAT 1-OH in water

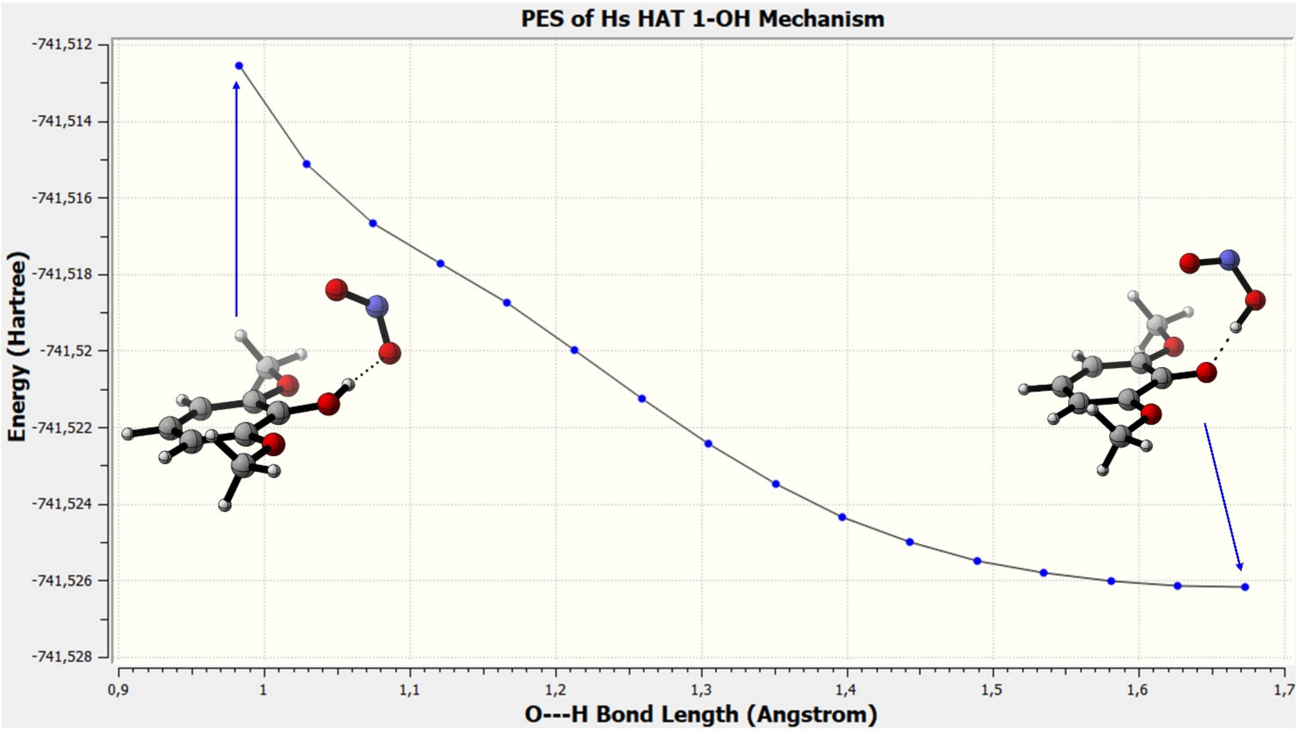

2b. Hs HAT 1-OH in pentyl ethanoate

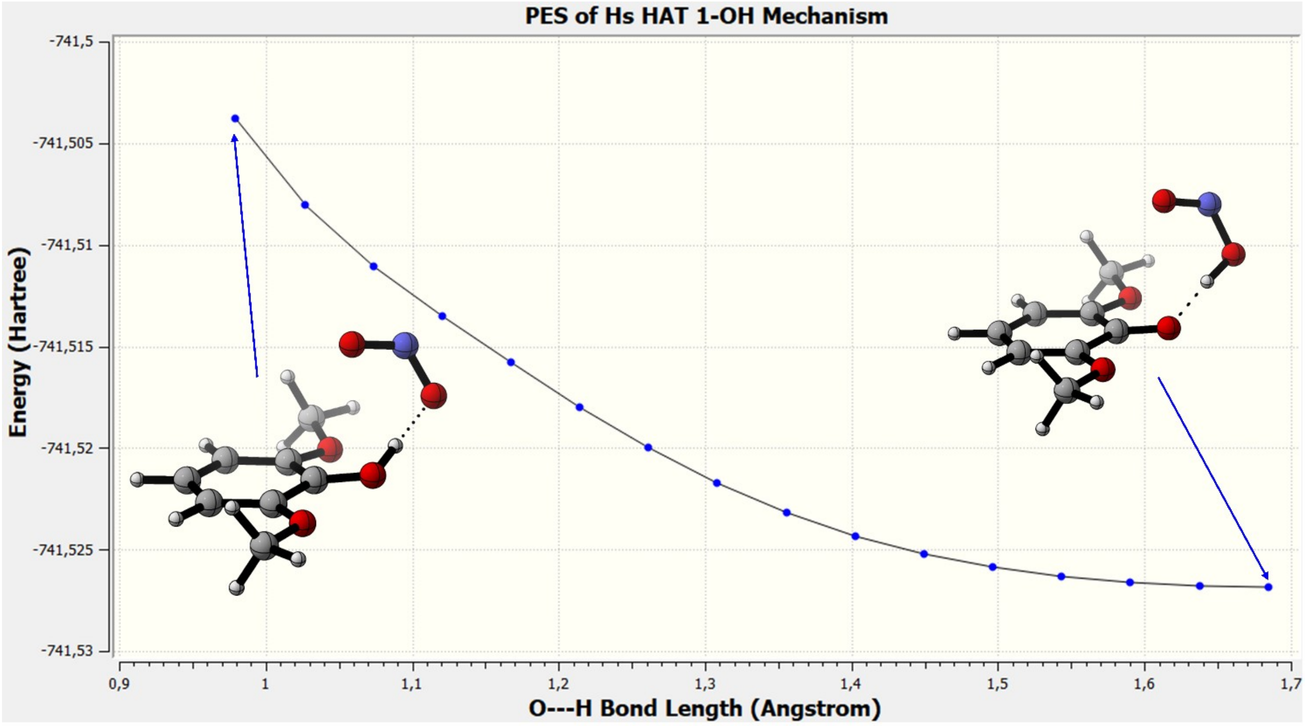

2c. HAs HAT 1-OH in water

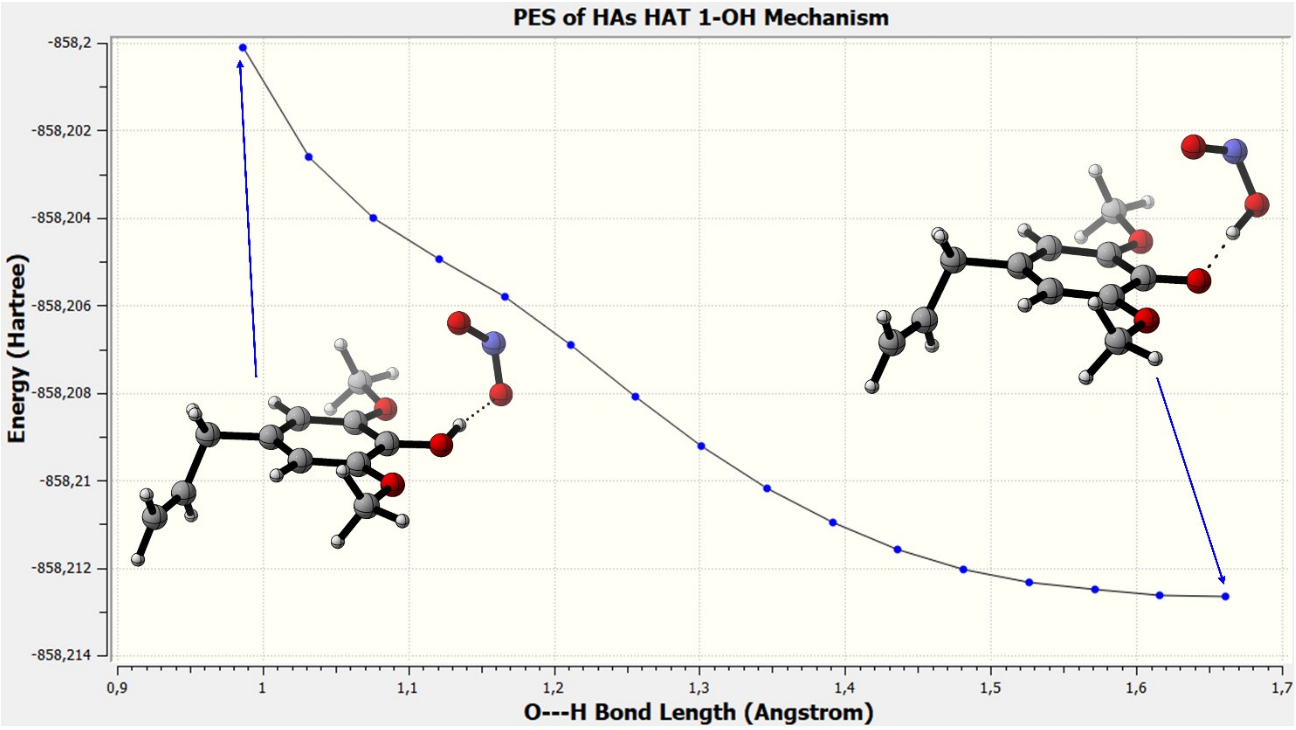

2d. HAs HAT 1-OH in pentyl ethanoate

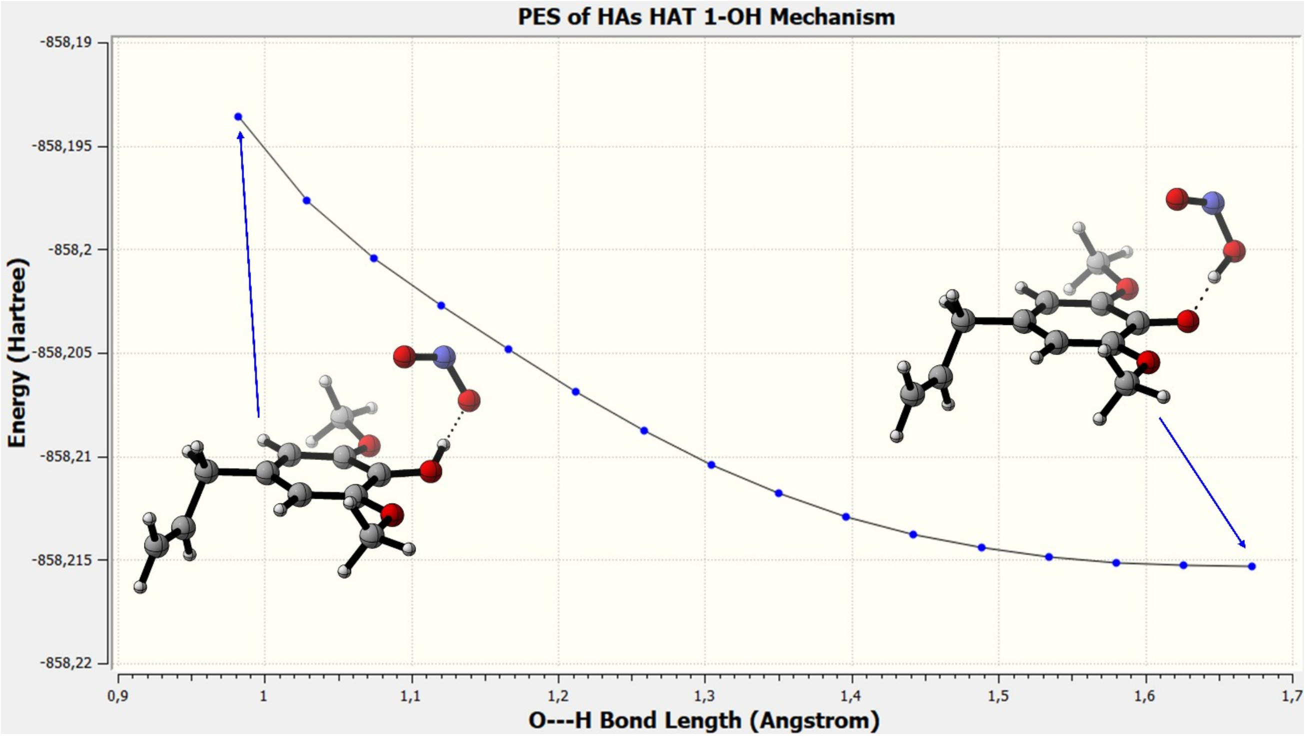

2e. HPns HAT 1-OH in water

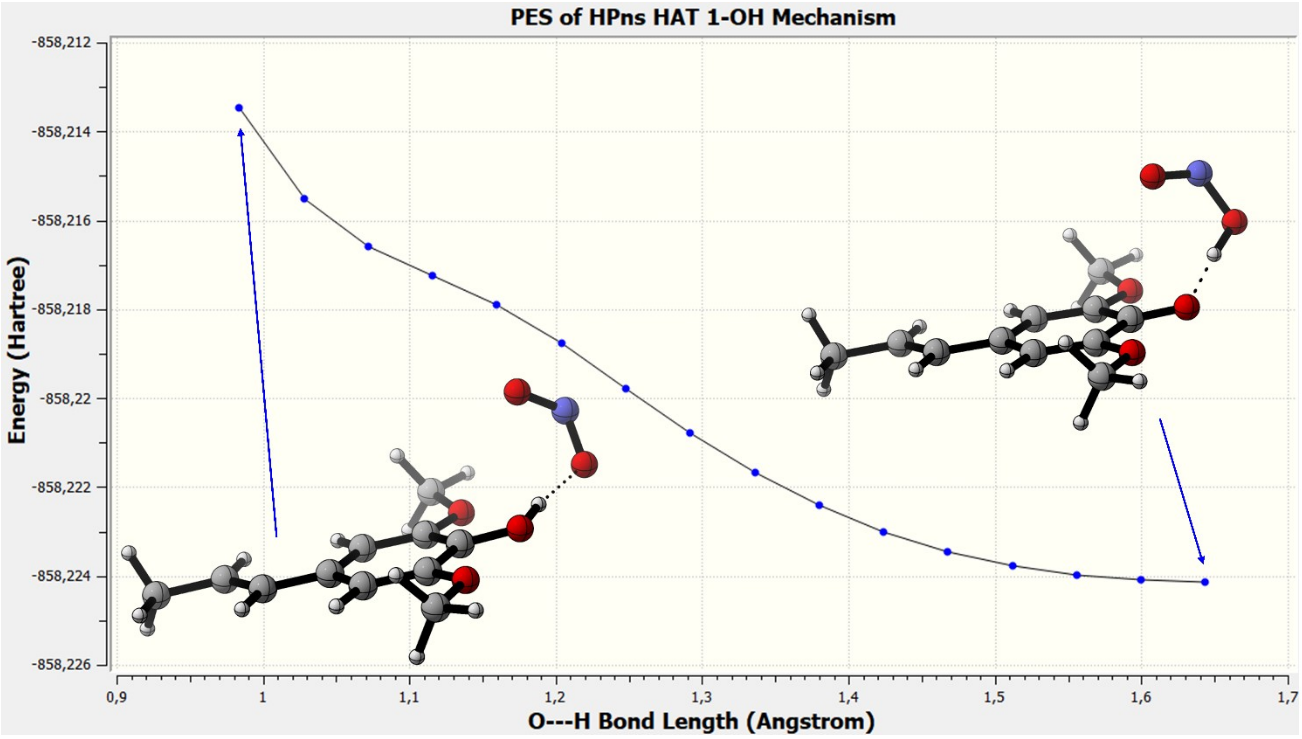

2f. HPns HAT 1-OH in pentyl ethanoate

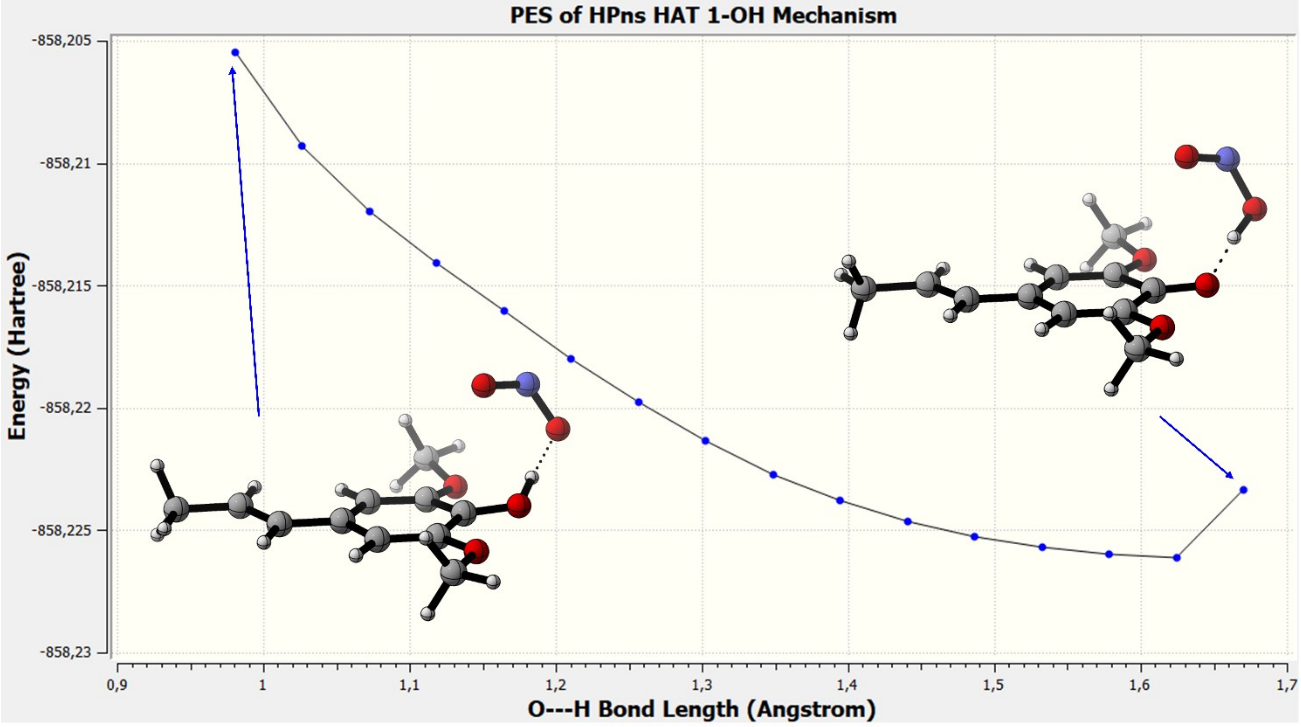

2g. HPs HAT 1-OH in water

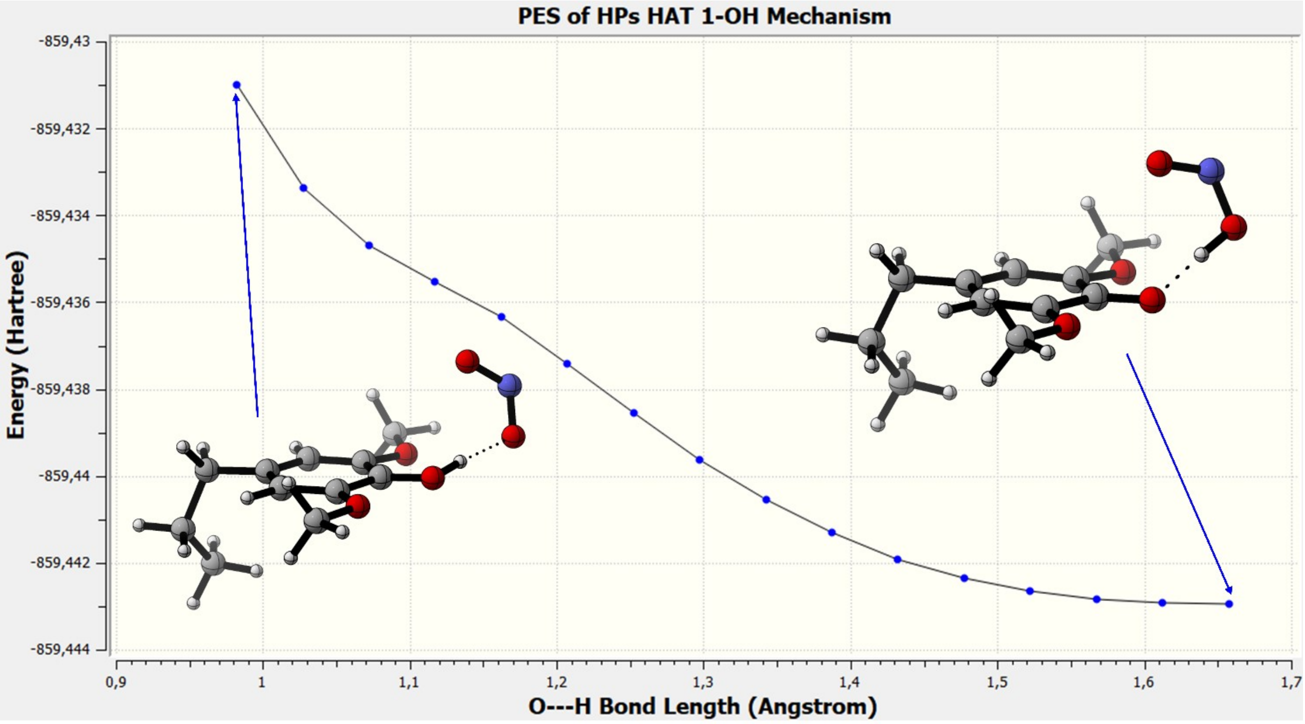

2h. HPs HAT 1-OH in pentyl ethanoate

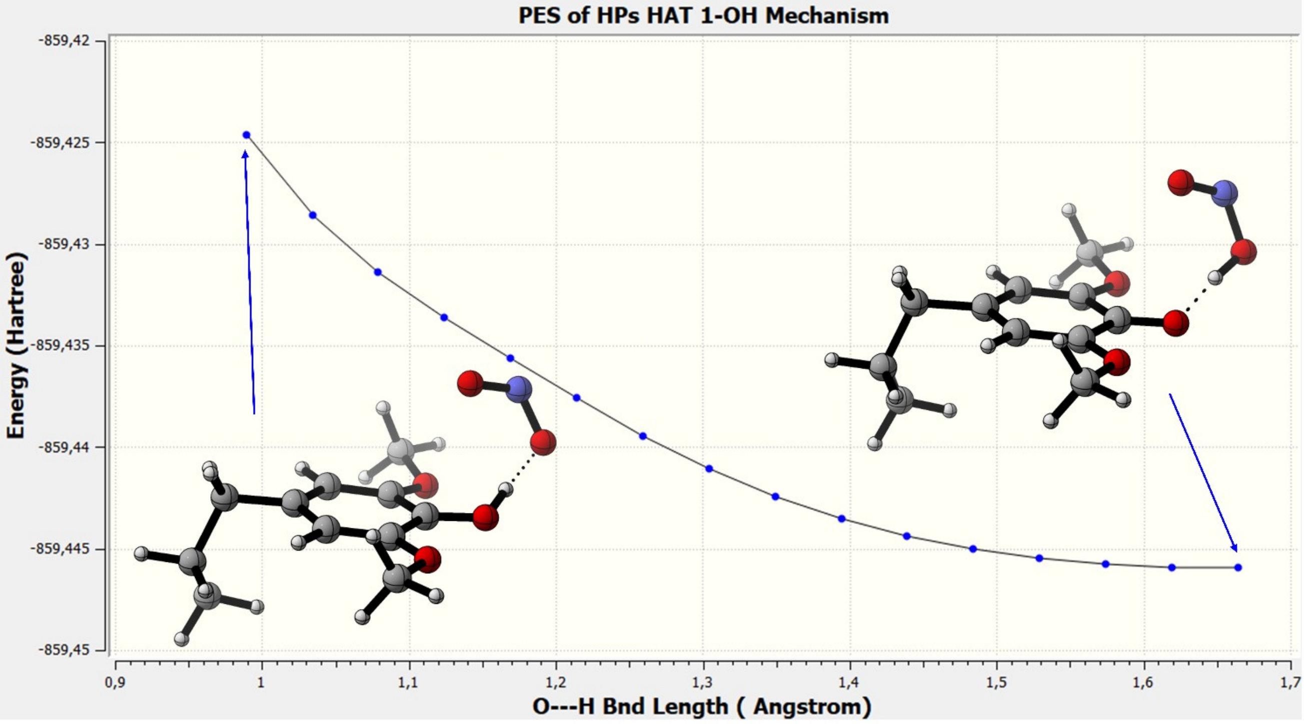

5. Figure S5: Intrinsic Reaction Coordinate of HAT and RAF Mechanism in Water

3a. HAs HAT 1a-CH···NOO•

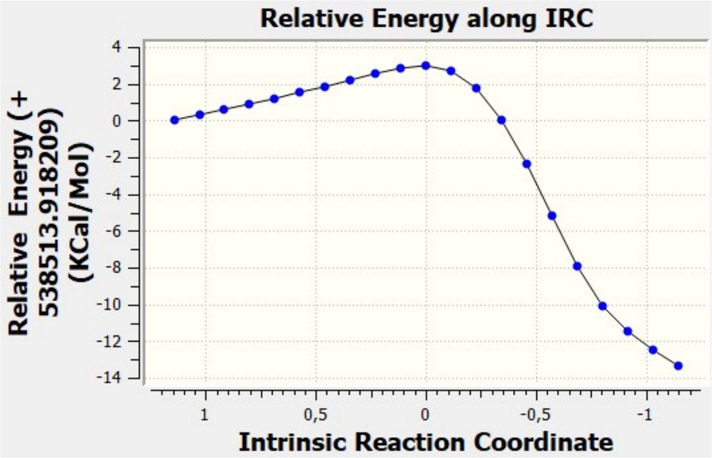

3b. Anionic HAs HAT 1a-CH···NOO•

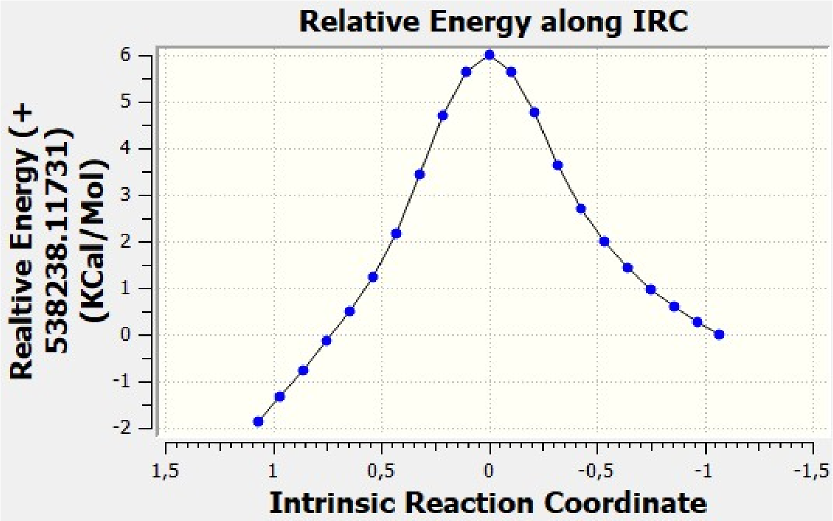

3c. HAs RAF C-2a···NOO•

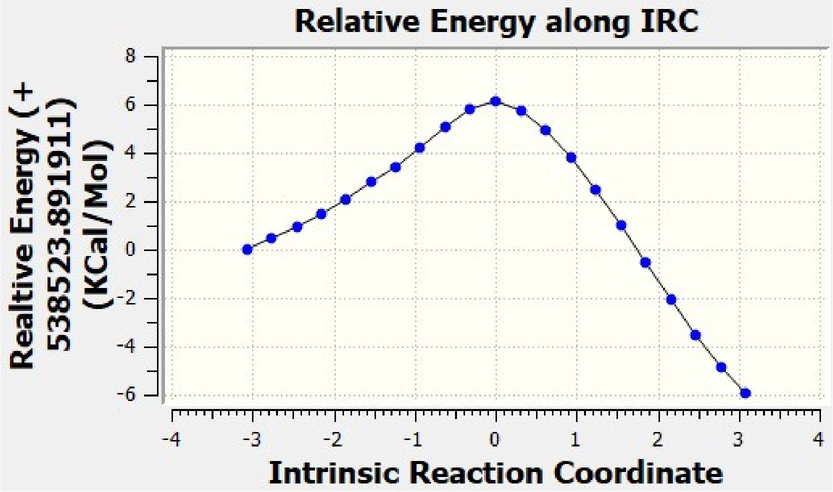

3d. Anionic HAs RAF C-2a···NOO•

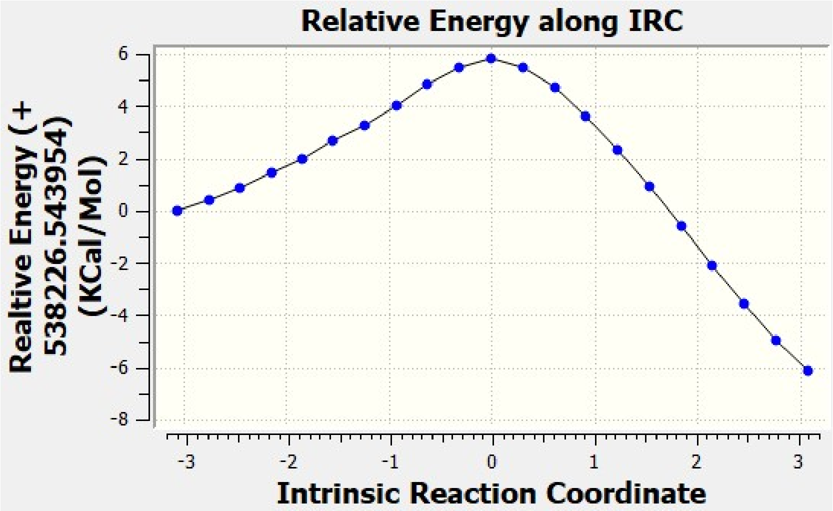

3e. HAs RAF C-3a···NOO•

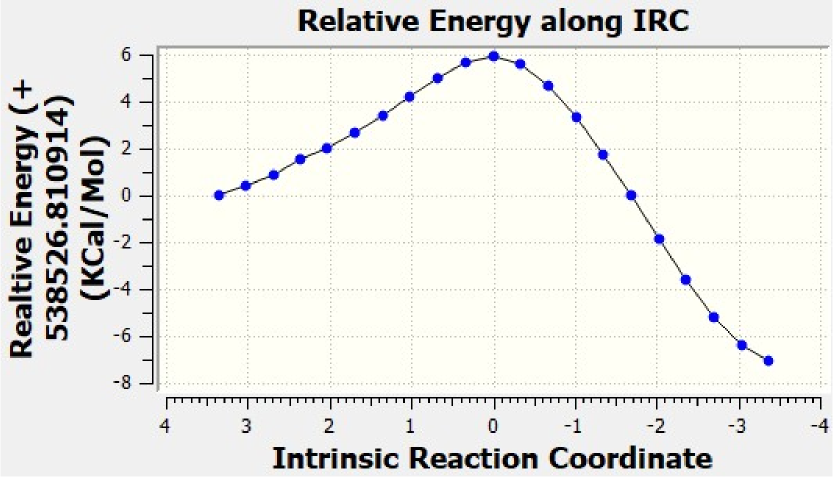

3f. Anionic HAs RAF C-3a···NOO•

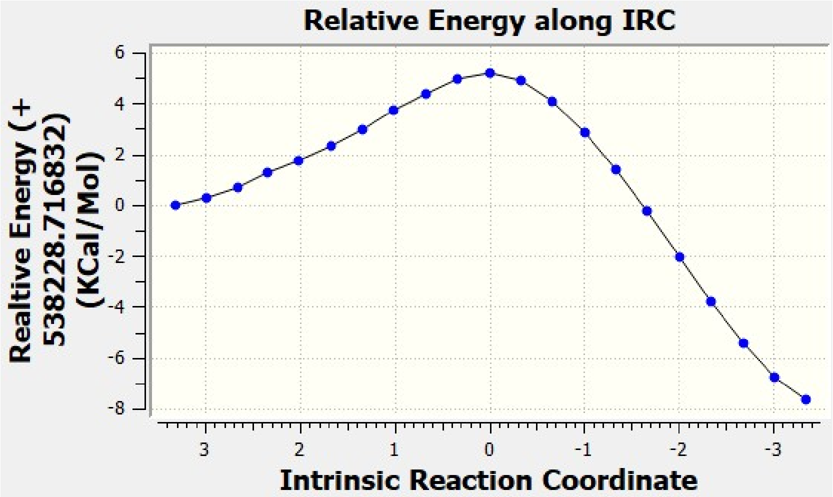

3g. HPns HAT 3a-CH···NOO•

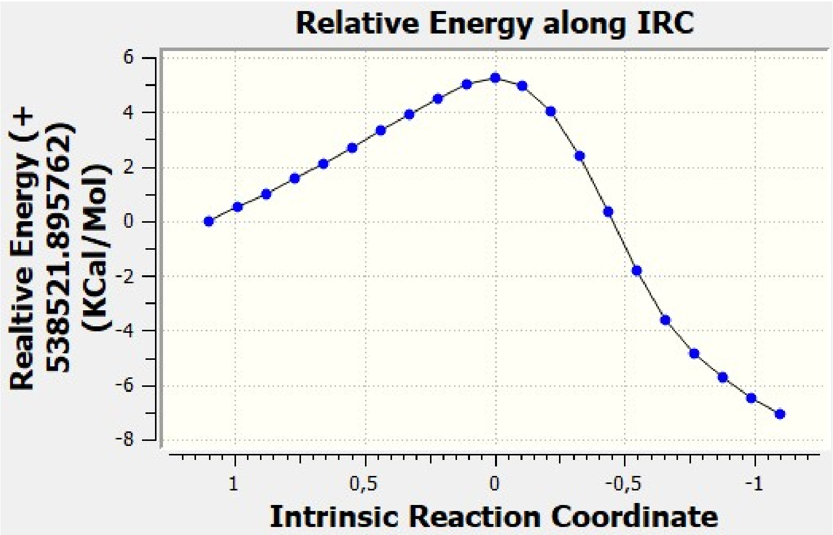

3h. Anionic HPns HAT 3a-CH···NOO•

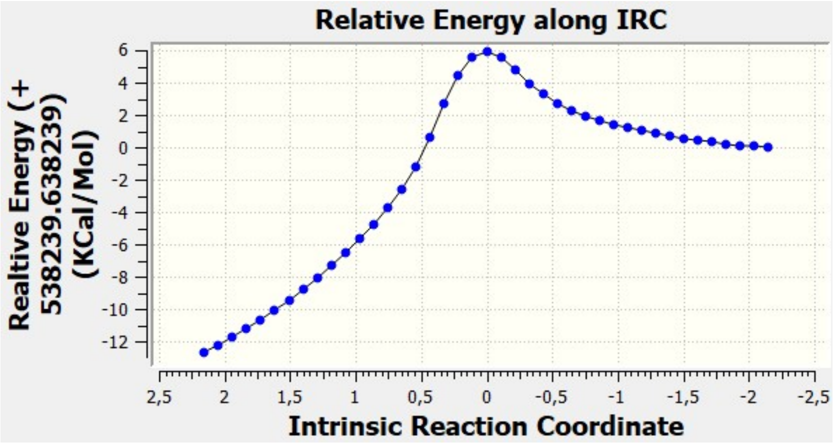

3i. HPns RAF C-2a···NOO•

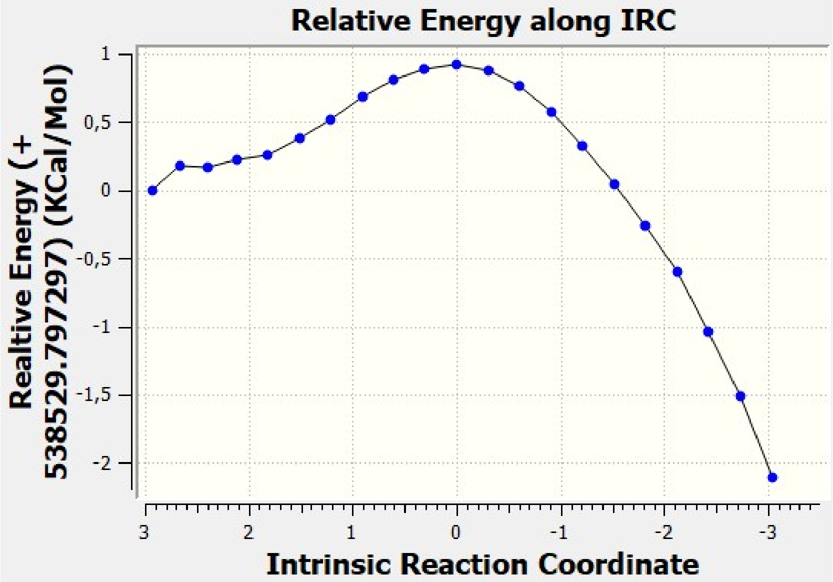

3j. Anionic HPns RAF C-2a···NOO•

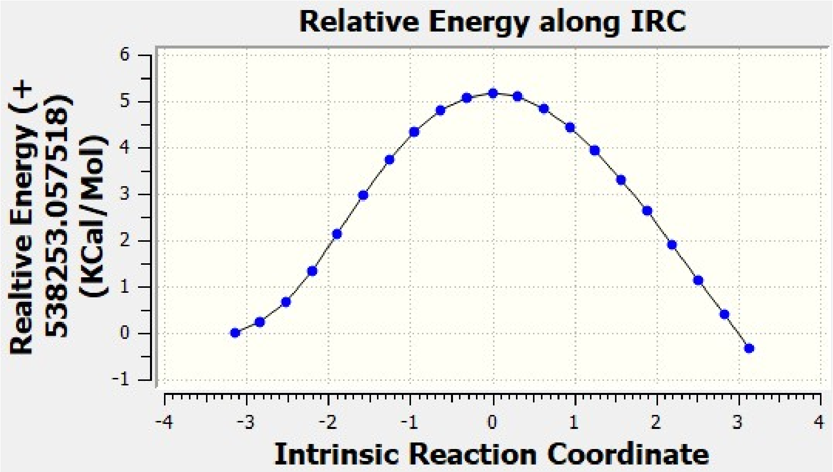

3k. HPs HAT 1a-CH···NOO$^{\bullet}$

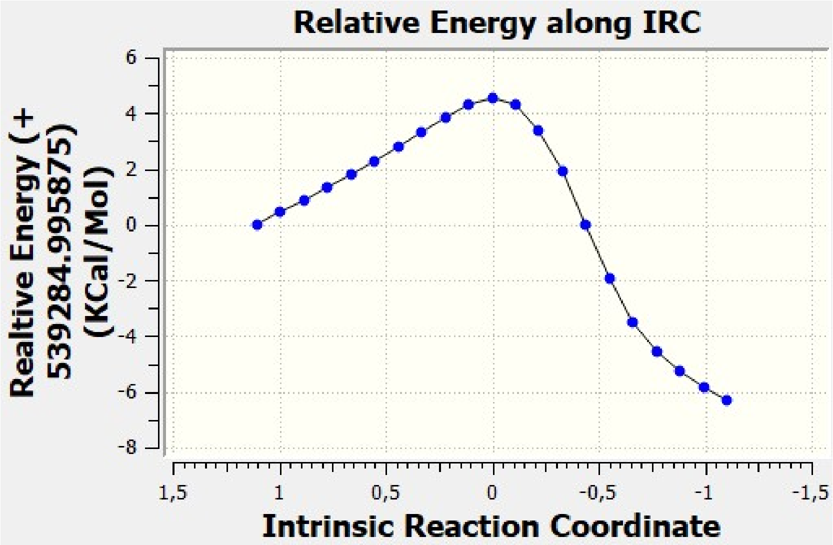

3l. Anionic HPs HAT 1a-CH···NOO$^{\bullet}$

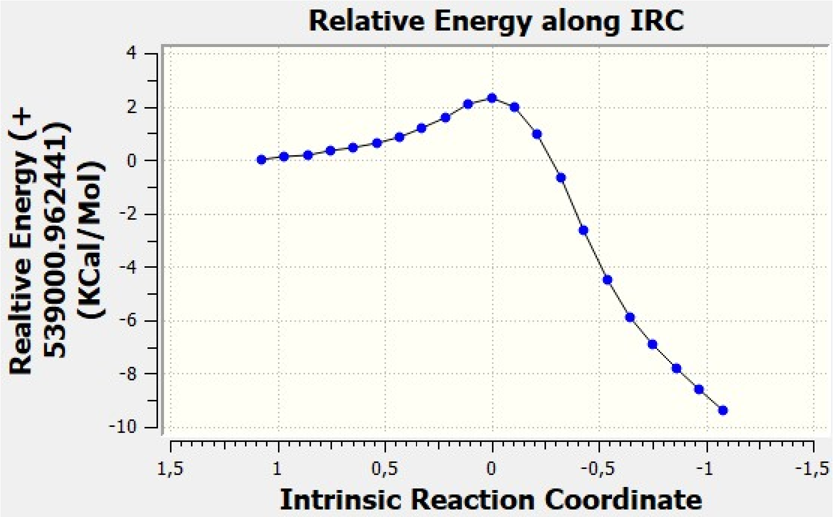

6. Figure S6: Intrinsic Reaction Coordinate of HAT and RAF Mechanism in Pentyl Ethanoate

4a. HAs HAT 1a-CH···NOO•

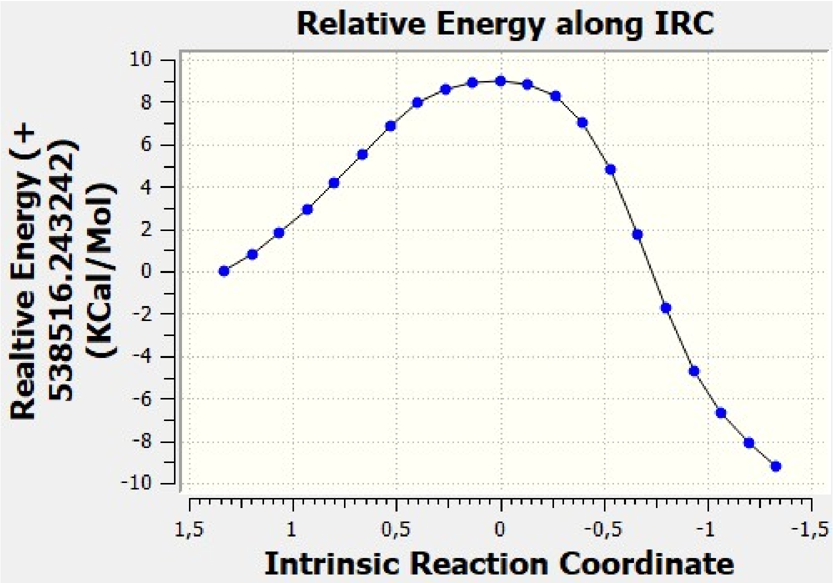

4b. HPns HAT 3a-CH···NOO•

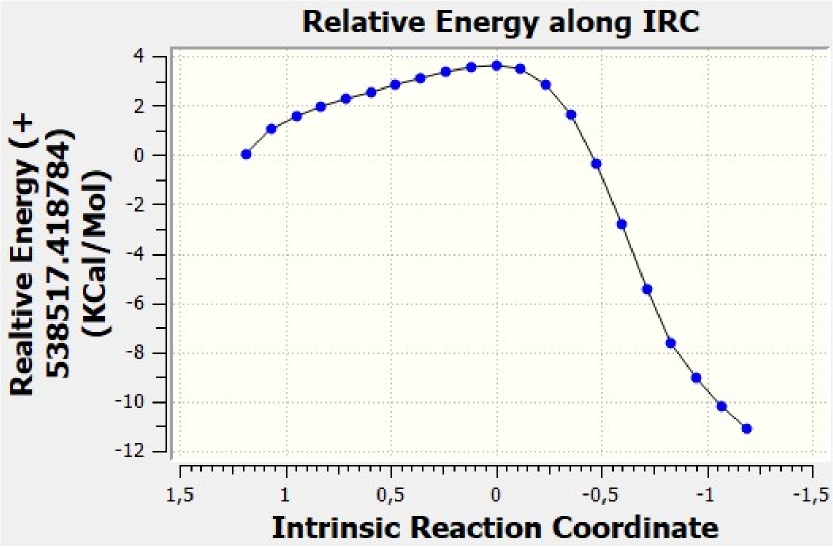

4c. HPns RAF C-2a···NOO•

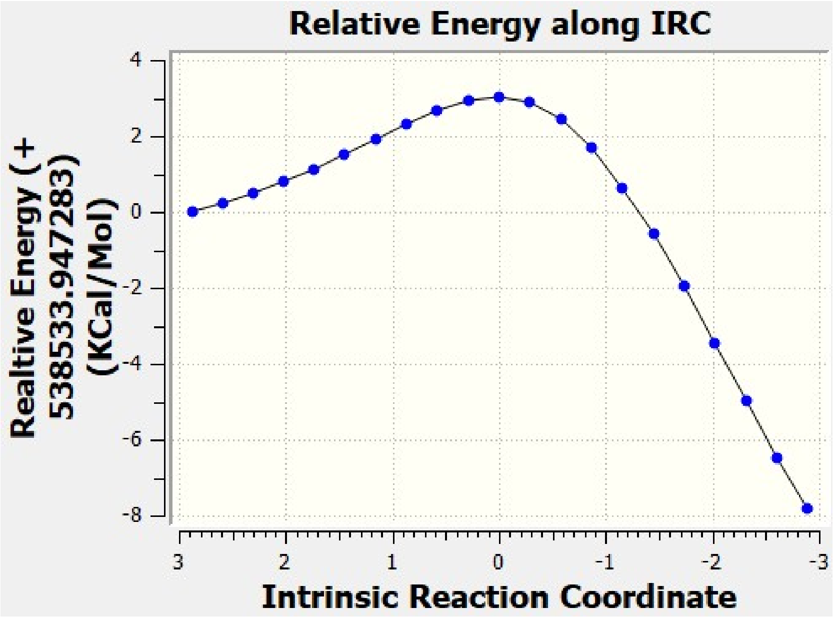

4d. HPs HAT 1a-CH···NOO•

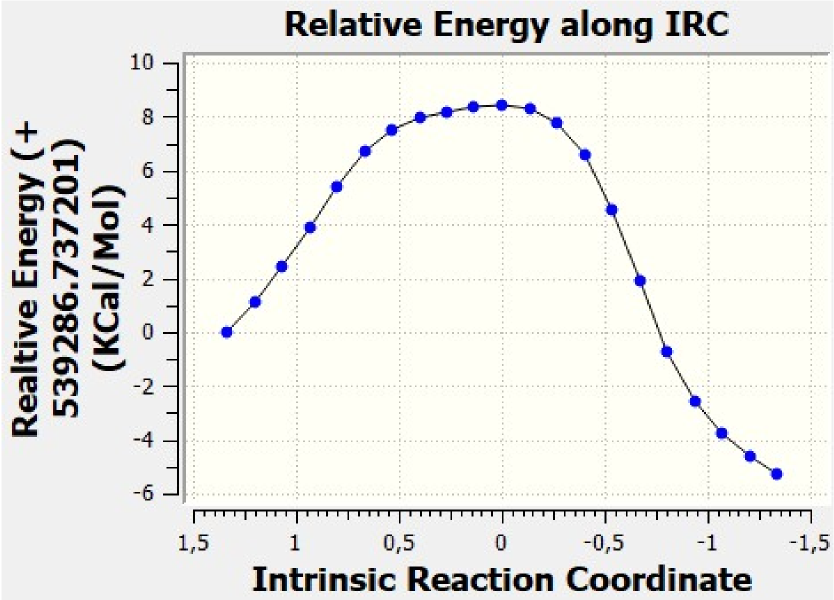

7. Table S7: Cartesian Coordinate and Thermochemical Values of all Optimized Stationary Points in Water

Name	Hs (Syringol)
Cartesian Coordinate:	Thermochemical Values:
O 2.32777200 -0.79878600 -0.00030400<br>O -2.31602600 -0.75685100 -0.00053900<br>O 0.00640000 -2.05358500 -0.00069000<br>C 0.02993100 2.08702600 -0.00031400<br>C 1.21856400 -0.00499500 -0.00043000<br>C -1.19980300 0.02960100 -0.00051800<br>C -1.19257300 1.42267800 -0.00037700<br>C 1.23431500 1.39010200 -0.00033700<br>C -0.00038900 -0.68418600 -0.00059500<br>C 3.59304500 -0.14409800 0.00143100<br>C -3.57965500 -0.09738700 0.00146900<br>H -2.11832600 1.98152900 -0.00029900<br>H 2.17090300 1.93013300 -0.00026200<br>H -0.91159800 -2.35969300 -0.00048700<br>H 3.71005500 0.47156700 0.89627900<br>H 3.71213200 0.47243900 -0.89253800<br>H 4.33798200 -0.93652600 0.00188600<br>H -3.69290200 0.51957400 -0.89267900<br>H -3.69063500 0.51832700 0.89676500<br>H -4.32832100 -0.88603700 0.00184200<br>H 0.04493000 3.17001800 -0.00021800	• Electronic Energy = -536.458220<br>• Zero Point Energy Correction = 0.170771<br>• Thermal Correction to Energy = 0.181536<br>• Thermal Correction to Enthalpy = 0.182480<br>• Thermal Correction to Free Energy = 0.133217

Name	Hs 1-OH radical
Cartesian Coordinate:	Thermochemical Values:
O 2.33973300 -0.78896300 0.00001200<br>O -2.33974500 -0.78895400 0.00000200<br>O 0.00000800 -2.03614600 0.00002000<br>C 0.00002600 2.02547300 0.00000000<br>C 1.24384100 -0.02265800 0.00001100<br>C -1.24384300 -0.02265200 0.00000500<br>C -1.23003800 1.35755800 -0.00000200<br>C 1.23008700 1.35755700 0.00000600<br>C -0.00004600 -0.78866100 0.00001300<br>C 3.60906400 -0.13154200 -0.00002000<br>C -3.60907400 -0.13153200 -0.00002700<br>H -2.14759700 1.92898000 -0.00000800<br>H 2.14765900 1.92894900 0.00000600<br>H 3.71712700 0.48342300 0.89542400<br>H 3.71709000 0.48340200 -0.89548300<br>H 4.35204100 -0.92458700 -0.00002500<br>H -3.71709400 0.48341700 -0.89548800<br>H -3.71712600 0.48343600 0.89541600<br>H -4.35205800 -0.92457000 -0.00003200<br>H -0.00010900 3.10879600 -0.00000400	• Electronic Energy = -535.822999<br>• Zero Point Energy Correction = 0.158473<br>• Thermal Correction to Energy = 0.168814<br>• Thermal Correction to Enthalpy = 0.169758<br>• Thermal Correction to Free Energy = 0.121775

Name	Hs 2’-CH radical
Cartesian Coordinate:	Thermochemical Values:
O 2.25929300 -0.82171500 0.03585900<br>O -2.39217500 -0.68813800 -0.17736500<br>O -0.07347700 -2.04162800 -0.06174500<br>C 0.00837500 2.10240300 -0.05729900<br>C 1.16542800 -0.01150400 -0.01289600<br>C -1.24110500 0.06621300 -0.10583400<br>C -1.22006400 1.45743300 -0.10790900<br>C 1.20025000 1.38102200 -0.01044000<br>C -0.06516400 -0.67572500 -0.06248100<br>C 3.53251900 -0.18766900 0.12374100<br>C -3.56017000 -0.13137700 0.27088100<br>H -2.14651300 2.01456300 -0.16299400<br>H 2.14526600 1.90522600 0.02587500<br>H -0.99209900 -2.34547300 -0.06857900<br>H 3.60002500 0.42025300 1.02891700<br>H 3.71838300 0.43248300 -0.75610400<br>H 4.26275800 -0.99241200 0.16604900<br>H -4.40765600 -0.78688300 0.13835000<br>H -3.49362500 0.57461800 1.08964400<br>H 0.04392500 3.18469300 -0.06172900	• Electronic Energy = -535.790297<br>• Zero Point Energy Correction = 0.156518<br>• Thermal Correction to Energy = 0.167151<br>• Thermal Correction to Enthalpy = 0.168096<br>• Thermal Correction to Free Energy = 0.120057

Name	Hs 2’-CH radical anion
Cartesian Coordinate:	Thermochemical Values:
O 2.27563900 -0.82497200 0.04723100<br>O -2.42620000 -0.71661100 -0.19425900<br>O -0.09368200 -2.08055300 -0.07791100<br>C -0.01970700 2.07590500 -0.06566100<br>C 1.14741500 -0.03760900 -0.01060800<br>C -1.24236600 0.01086100 -0.11493000<br>C -1.23321900 1.40228500 -0.12239100<br>C 1.17480000 1.35102000 -0.00776600<br>C -0.06986000 -0.78174400 -0.07014900<br>C 3.52601800 -0.15849800 0.12943200<br>C -3.56208400 -0.15177600 0.30300400<br>H -2.16867500 1.94582200 -0.18739600<br>H 2.11707800 1.88167600 0.03623600<br>H 3.58429900 0.45879700 1.03027800<br>H 3.69870500 0.46537200 -0.75193000<br>H 4.28315700 -0.93901100 0.17484300<br>H -4.43051000 -0.78383700 0.18384300<br>H -3.46061400 0.52722300 1.14187200<br>H 0.00452500 3.15837600 -0.07381500	• Electronic Energy = -535.319609<br>• Zero Point Energy Correction = 0.143812<br>• Thermal Correction to Energy = 0.154030<br>• Thermal Correction to Enthalpy = 0.154975<br>• Thermal Correction to Free Energy = 0.107528

Name	Hs radical cation
Cartesian Coordinate:	Thermochemical Values:
O -2.31362900 -0.78734400 0.00001500<br>O 2.30769900 -0.74772200 0.00005200<br>O -0.01179700 -1.98623500 0.00009500<br>C -0.03452100 2.06244800 0.00001500<br>C -1.26132000 0.00858400 0.00004200<br>C 1.24496100 0.04522700 0.00005700<br>C 1.21457800 1.41857700 0.00002500<br>C -1.25916800 1.38975800 0.00001900<br>C 0.00317000 -0.68803900 0.00007600<br>C -3.61477100 -0.17863800 -0.00015000<br>C 3.60489100 -0.13092800 -0.00013300<br>H 2.12412300 2.00205200 0.00000600<br>H -2.18231000 1.95067100 -0.00000100<br>H 0.89494100 -2.34912700 0.00009300<br>H -3.73704900 0.42896900 -0.89758600<br>H -3.73724200 0.42906500 0.89719500<br>H -4.31974000 -1.00432600 -0.00018000<br>H 3.72138000 0.47805000 0.89720900<br>H 3.72118100 0.47790700 -0.89759700<br>H 4.31696300 -0.95047400 -0.00014400<br>H -0.04735800 3.14569100 -0.00000300	• Electronic Energy = -536.248048<br>• Zero Point Energy Correction = 0.171067<br>• Thermal Correction to Energy = 0.181809<br>• Thermal Correction to Enthalpy = 0.182753<br>• Thermal Correction to Free Energy = 0.133557

Name	Hs RAF C-4···NO$^{\bullet}$ Product
Cartesian Coordinate:	Thermochemical Values:
O 2.30731700 -1.07036600 0.07953600<br>O -2.32708600 -1.03544100 0.04843200<br>O -0.01018000 -2.22992200 0.49908400<br>C 1.22671100 -0.26236100 -0.05459200<br>C -1.24270400 -0.22843800 -0.07515300<br>C -1.25716600 1.08601600 -0.41728200<br>C 1.26113500 1.05488000 -0.40439400<br>C -0.01663900 -0.92176300 0.16099900<br>C 3.58749800 -0.47176800 -0.10923600<br>C -3.60826700 -0.43546600 -0.13347400<br>H -2.18110700 1.61280500 -0.61179000<br>H 2.19396400 1.57075700 -0.58474800<br>H -0.92654200 -2.53605900 0.58042800<br>H 3.74227600 0.33623600 0.60942500<br>H 3.68689700 -0.08941100 -1.12755700<br>H 4.31374900 -1.26296100 0.05998000<br>H -3.70582700 -0.04626900 -1.14908000<br>H -3.75745600 0.36764000 0.59150800<br>H -4.33514400 -1.22675500 0.03140800<br>C 0.01042300 1.86188900 -0.43608400<br>H 0.02773400 2.61378500 -1.22761800<br>N 0.07179000 2.66972700 0.88191300<br>O 0.31963400 3.82259200 0.71470400	• Electronic Energy = -666.289616<br>• Zero Point Energy Correction = 0.177793<br>• Thermal Correction to Energy = 0.190804<br>• Thermal Correction to Enthalpy = 0.191748<br>• Thermal Correction to Free Energy = 0.137138

Name	Anionic Hs RAF C-4···NO$^{\bullet}$ Product
Cartesian Coordinate:	Thermochemical Values:
O 2.33093100 -1.07194800 0.01931200<br>O -2.37072100 -1.09588500 -0.02374100<br>O -0.01788500 -2.26082600 0.15524400<br>C 1.23496200 -0.27816300 -0.11769500<br>C -1.28464600 -0.28762100 -0.12712400<br>C -1.28627400 1.04144300 -0.29793800<br>C 1.22339400 1.04926300 -0.30615600<br>C -0.02012900 -1.05052700 -0.02045900<br>C 3.59288700 -0.41625600 -0.03354500<br>C -3.64118900 -0.45711000 -0.08737600<br>H -2.21496400 1.59731600 -0.35731200<br>H 2.14242000 1.62055400 -0.36072900<br>H 3.67938400 0.31745800 0.77217900<br>H 3.72991500 0.07864300 -0.99846700<br>H 4.34252800 -1.19387700 0.09243900<br>H -3.76864900 0.05141200 -1.04643500<br>H -3.75104800 0.26161500 0.72874700<br>H -4.38127000 -1.24740000 0.01323400<br>C -0.03598400 1.83538400 -0.41233000<br>H -0.04021600 2.32856500 -1.39650800<br>N -0.04559500 2.93602300 0.61045200<br>O 0.87886500 3.81469700 0.30746100	• Electronic Energy = -665.848538<br>• Zero Point Energy Correction = 0.166353<br>• Thermal Correction to Energy = 0.178770<br>• Thermal Correction to Enthalpy = 0.179714<br>• Thermal Correction to Free Energy = 0.126774

Name	Hs RAF C-1···NOO$^{\bullet}$ (Model 1) Product
Cartesian Coordinate:	Thermochemical Values:
O 2.34152300 0.03174700 0.72508300<br>O -2.33320400 0.02727500 0.68440200<br>O 0.01889500 0.21170600 2.02262000<br>C 0.02251200 -1.94729900 -1.34946900<br>C 1.25707400 -0.50592000 0.13436800<br>C -1.24354100 -0.50632000 0.09614100<br>C -1.21531300 -1.47235700 -0.86634000<br>C 1.24519000 -1.46963600 -0.83376300<br>C 3.61640000 -0.46810400 0.32251700<br>C -3.60595900 -0.49263200 0.29861400<br>H -2.13637100 -1.88709400 -1.25424700<br>H 2.17465500 -1.88006900 -1.20538000<br>H -0.86859000 0.45503700 2.32610700<br>H 3.77841300 -0.28182700 -0.74136900<br>H 3.68785000 -1.53765100 0.53142600<br>H 4.34960300 0.07646600 0.91176000<br>H -3.65778200 -1.56184000 0.51442200<br>H -3.77951100 -0.31443500 -0.76458200<br>H -4.34078800 0.04513600 0.89187400<br>H 0.03414700 -2.70789500 -2.11833300<br>C -0.00009900 0.13252600 0.63467700<br>O 0.00188900 1.58450700 0.23693500<br>N 0.00641400 1.73508800 -1.11185100<br>O 0.00288200 2.87314100 -1.41019000	• Electronic Energy = -741.497957<br>• Zero Point Energy Correction = 0.181154<br>• Thermal Correction to Energy = 0.195220<br>• Thermal Correction to Enthalpy = 0.196164<br>• Thermal Correction to Free Energy = 0.139762

Name	Hs RAF C-2···NOO$^{\bullet}$ (Model 1) Product
Cartesian Coordinate:	Thermochemical Values:
O -1.56910400 -1.28182100 -0.95205400<br>O 2.70547800 0.20208500 -0.73120700<br>O 0.36698000 0.27595900 -2.00007900<br>C 0.38269100 -0.87887800 1.96733900<br>C 1.60920200 -0.14613000 0.00307500<br>C 1.61812500 -0.53787300 1.33362800<br>C -0.80695200 -0.83014600 1.32942200<br>C 0.41655800 -0.08777800 -0.70753900<br>C -2.80210900 -1.82781300 -0.46730400<br>C 3.97344800 0.13243400 -0.08150000<br>H 2.53998800 -0.58855100 1.89500500<br>H -1.73397600 -1.06592000 1.83665700<br>H 1.26755900 0.44731400 -2.31872000<br>H -3.44424100 -1.04412000 -0.06156800<br>H -2.61509500 -2.59145000 0.28928300<br>H -3.28309000 -2.28137000 -1.33114500<br>H 4.17915400 -0.88805600 0.24914100	• Electronic Energy = -741.489314<br>• Zero Point Energy Correction = 0.181052<br>• Thermal Correction to Energy = 0.195160<br>• Thermal Correction to Enthalpy = 0.196104<br>• Thermal Correction to Free Energy = 0.139475

H 4.00641000 0.81624500 0.76954800<br>H 4.70596500 0.43477800 -0.82584000<br>H 0.41349100 -1.18347700 3.00693700<br>C -0.90542900 -0.40406700 -0.09506000<br>O -1.77435100 0.82557400 -0.18802200<br>N -1.28509000 1.84206100 0.55889900<br>O -1.97322200 2.79466400 0.48211800	

Name	Hs RAF C-3···NOO• (Model 1) Product
Cartesian Coordinate:	Thermochemical Values:
O 1.53412600 1.75398000 -0.22966700<br>O -2.88291800 0.27977300 -0.41281100<br>O -0.99859800 2.03486100 -1.04690000<br>C -0.07779900 -1.20896500 1.43046400<br>C 0.61176900 0.84325600 0.19600500<br>C -1.66494000 0.02189200 0.10888200<br>C -1.35143200 -1.04797900 0.96935700<br>C -0.66734600 0.97368500 -0.24985300<br>C 2.65884800 1.98894500 0.62399700<br>C -3.96275300 -0.58840000 -0.06241000<br>H -2.12821500 -1.73289400 1.28417200<br>H -1.94912800 1.99863600 -1.22452300<br>H 3.30701800 1.11145600 0.66981400<br>H 2.32580900 2.26609000 1.62667600<br>H 3.20184000 2.81750800 0.17533500<br>H -4.13174200 -0.56731700 1.01581300<br>H -3.75322300 -1.60711700 -0.39358200<br>H -4.83439600 -0.20047300 -0.58291400<br>H 0.16740500 -2.01401300 2.11133200<br>C 1.04079900 -0.33367200 0.99689100<br>O 2.05447700 -1.12642800 0.20610800<br>H 1.67286900 -0.04973000 1.84087200<br>N 1.50806200 -1.63131400 -0.92119600<br>O 2.29822100 -2.25412700 -1.53605800	• Electronic Energy = -741.487618<br>• Zero Point Energy Correction = 0.181609<br>• Thermal Correction to Energy = 0.195837<br>• Thermal Correction to Enthalpy = 0.196782<br>• Thermal Correction to Free Energy = 0.139866

Name	Anionic Hs RAF C-3···NOO• (Model 1) Product
Cartesian Coordinate:	Thermochemical Values:
O 1.62145700 1.69281800 -0.32738800<br>O -2.89440800 0.33275500 -0.44190800<br>O -0.96906200 2.00807900 -1.19124000<br>C -0.12334500 -1.09322400 1.48648300<br>C 0.61612200 0.86148300 0.14836700<br>C -1.66953300 0.10983700 0.08401500<br>C -1.39224000 -0.91447000 1.00549800<br>C -0.65143300 1.05526400 -0.36628800<br>C 2.62336900 2.06370700 0.61397800<br>C -3.96161600 -0.52898900 -0.05407500<br>H -2.18783300 -1.55790900 1.35946100<br>H 3.28633800 1.22466200 0.84360500<br>H 2.16910700 2.43639300 1.53678200<br>H 3.20526200 2.85789500 0.14867000<br>H -4.14591700 -0.45643700 1.02002400<br>H -3.74069200 -1.56341100 -0.32620700<br>H -4.83615400 -0.18382300 -0.60050300<br>H 0.09364100 -1.86613900 2.21280400<br>C 1.00279500 -0.27101000 1.00103700<br>O 2.01058900 -1.18552000 0.22066300<br>H 1.68938400 -0.00238800 1.80609400<br>N 1.44003300 -1.72335700 -0.85124200<br>O 2.19666400 -2.41324700 -1.45464200	• Electronic Energy = -741.016205<br>• Zero Point Energy Correction = 0.168615<br>• Thermal Correction to Energy = 0.182542<br>• Thermal Correction to Enthalpy = 0.183486<br>• Thermal Correction to Free Energy = 0.126858

Name	Hs RAF C-4···NOO• (Model 1) Product
Cartesian Coordinate:	Thermochemical Values:
O 2.27390300 -1.54404500 0.22238500<br>O -2.36525600 -1.36515500 0.20484500<br>O -0.07560800 -2.47109500 0.93081800<br>C 1.21482400 -0.78708600 -0.16317800<br>C -1.25624400 -0.68097800 -0.18010600<br>C -1.23484600 0.47762000 -0.88488000<br>C 1.28389300 0.37130700 -0.86924100<br>C -0.04838200 -1.32635000 0.22261600<br>C 3.56964600 -1.04738300 -0.10337300	• Electronic Energy = -741.492923<br>• Zero Point Energy Correction = 0.182109<br>• Thermal Correction to Energy

C -3.62811100 -0.78595300 -0.11739600<br>H -2.14260200 0.96646400 -1.21096600<br>H 2.22908100 0.78661900 -1.19010200<br>H -0.99924700 -2.71424500 1.10005200<br>H 3.73170600 -0.06880700 0.35455200<br>H 3.69400600 -0.97707300 -1.18640100<br>H 4.27553300 -1.76777700 0.30276100<br>H -3.74768200 -0.71092600 -1.20041100<br>H -3.72048500 0.20211900 0.33883300<br>H -4.37844300 -1.45726700 0.29254300<br>C 0.05674500 1.14778400 -1.18849400<br>O 0.10791500 2.48106900 -0.47923200<br>H 0.07919600 1.51173800 -2.21692200<br>N 0.09287100 2.32474400 0.85665400<br>O 0.13200600 3.36699900 1.41165700	= 0.196090<br>• Thermal Correction to Enthalpy<br>= 0.197034<br>• Thermal Correction to Free Energy<br>= 0.140561

Name	Hs RAF C-1···NOO• (Model 2) Product
Cartesian Coordinate:	Thermochemical Values:
O 2.33690900 -0.63167900 -0.13457500<br>O -2.32445300 -0.64547200 -0.12457000<br>O 0.03572800 -1.64251800 -1.01322300<br>C -0.00178900 2.20348500 0.14782200<br>C 1.25184600 0.15919200 -0.05523200<br>C -1.24710900 0.15818900 -0.03322800<br>C -1.23157300 1.51703200 0.09309600<br>C 1.22797200 1.51955500 0.06004900<br>C 3.61266400 0.01094300 -0.12160500<br>C -3.60808300 -0.01747100 -0.15460500<br>H -2.15941000 2.07244500 0.12872600<br>H 2.15250700 2.08095700 0.07850300<br>H -0.85777000 -1.99999600 -1.12959600<br>H 3.75004500 0.55898700 0.81280100<br>H 3.70230300 0.68896000 -0.97275500<br>H 4.34751700 -0.78587700 -0.19936000<br>H -3.67407800 0.65958600 -1.00863800<br>H -3.78095500 0.52847800 0.77479700<br>H -4.33097200 -0.82232500 -0.25632800<br>H -0.00163100 3.28081300 0.24245200<br>C 0.00406700 -0.65030100 -0.06141900<br>N -0.00142100 -1.45818700 1.38137200<br>O 0.24580100 -0.80449800 2.37003000<br>O -0.30901300 -2.62420500 1.37856000	• Electronic Energy<br>= -741.502664<br>• Zero Point Energy Correction<br>= 0.182708<br>• Thermal Correction to Energy<br>= 0.196472<br>• Thermal Correction to Enthalpy<br>= 0.197416<br>• Thermal Correction to Free Energy<br>= 0.141364

Name	Hs RAF C-3···NOO• (Model 2) Product
Cartesian Coordinate:	Thermochemical Values:
O 2.08035300 -1.19631000 0.07492600<br>O -2.54448500 -0.63634300 0.13435800<br>O -0.39540600 -2.18336300 0.28929200<br>C 0.02853000 1.89128000 -0.53577400<br>C 1.02637000 -0.34827600 -0.09381000<br>C -1.37141200 -0.00271700 -0.05214700<br>C -1.21747600 1.35862800 -0.37567400<br>C -0.23035700 -0.85121600 0.05091400<br>C 3.29344900 -0.87347400 -0.61428300<br>C -3.74826400 0.12885100 0.03131700<br>H -2.09010300 1.97829100 -0.53750600<br>H -1.34289800 -2.37471700 0.33933100<br>H 3.77460100 0.00502100 -0.17825700<br>H 3.09711500 -0.70892900 -1.67600100<br>H 3.94306100 -1.73639900 -0.48887500<br>H -3.85427300 0.53284400 -0.97687400<br>H -3.74579100 0.93595000 0.76615000<br>H -4.55761900 -0.56507500 0.24142600<br>H 0.16290900 2.92761000 -0.81604200<br>C 1.25131800 1.09935000 -0.26339100<br>H 2.07370100 1.35356000 -0.93298600<br>N 1.83819600 1.67945900 1.12545700<br>O 1.49097100 1.16024100 2.16107900<br>O 2.54755500 2.65818900 1.04660000	• Electronic Energy<br>= -741.495277<br>• Zero Point Energy Correction<br>= 0.183017<br>• Thermal Correction to Energy<br>= 0.197028<br>• Thermal Correction to Enthalpy<br>= 0.197972<br>• Thermal Correction to Free Energy<br>= 0.141061

Name	Hs RAF C-4···NOO• (Model 2) Product
Cartesian Coordinate:	Thermochemical Values:

O 2.31489200 -1.06811000 0.06864400<br>O -2.32071700 -1.04859800 0.05272500<br>O 0.00304000 -2.23627600 0.47256000<br>C 1.23241500 -0.26558400 -0.06619500<br>C -1.24056000 -0.24137300 -0.07718600<br>C -1.26116700 1.07642300 -0.40065700<br>C 1.26141900 1.05119100 -0.40353600<br>C -0.01077300 -0.93406000 0.13921300<br>C 3.59311100 -0.46065500 -0.10874900<br>C -3.60580200 -0.44708700 -0.10178600<br>H -2.18562000 1.60592000 -0.58364500<br>H 2.18744000 1.57576900 -0.59191900<br>H -0.91069100 -2.55136100 0.55803100<br>H 3.73835700 0.34344100 0.61616200<br>H 3.69523100 -0.07141500 -1.12419700<br>H 4.32258900 -1.24890500 0.05958300<br>H -3.71853500 -0.04657300 -1.11134100<br>H -3.74329300 0.34691000 0.63520300<br>H -4.32877600 -1.24131600 0.06521400<br>C 0.00209400 1.84015600 -0.45550400<br>H 0.01732700 2.58064500 -1.25562600<br>N 0.08515300 2.80366800 0.81441800<br>O -0.43357900 2.45538200 1.85052100<br>O 0.73460000 3.81977500 0.68257100	• Electronic Energy = -741.501498<br>• Zero Point Energy Correction = 0.183877<br>• Thermal Correction to Energy = 0.197502<br>• Thermal Correction to Enthalpy = 0.198446<br>• Thermal Correction to Free Energy = 0.142321

Name	HAs (4-allylsyringol)
Cartesian Coordinate:	Thermochemical Values:
O -0.15653000 2.73924500 0.03093200<br>O -2.81752100 -1.06494900 0.14164200<br>O -2.51545100 1.57916200 0.29042400<br>C 0.83213900 -0.80119300 -0.37078900<br>C 2.07074700 -1.64437700 -0.60106600<br>C -0.41750700 -1.39987300 -0.23180600<br>C 0.95754200 0.58675100 -0.28450400<br>C -0.16705400 1.37873600 -0.06549700<br>C -1.54093200 -0.60327900 -0.01014000<br>C -1.42157900 0.78198500 0.07325900<br>C 3.03604700 -1.53827800 0.54731600<br>C 1.10461000 3.38921500 -0.09790700<br>C -3.01955500 -2.47356300 0.06198600<br>C 4.26959800 -1.05226300 0.44665400<br>H 1.76639800 -2.68696600 -0.72886900<br>H 2.57046900 -1.32736500 -1.52104400<br>H -0.50965300 -2.47627600 -0.29889300<br>H 1.93658600 1.03819500 -0.38873800<br>H 2.66459600 -1.87432100 1.51390600<br>H -3.29419300 1.00926600 0.36099200<br>H 1.54352000 3.19358200 -1.07910700<br>H 1.79077100 3.06369700 0.68752500<br>H 0.90331400 4.45271400 0.00825600<br>H -2.46464300 -2.98762600 0.84998100<br>H -2.71539000 -2.85156000 -0.91659100<br>H -4.08651000 -2.63040400 0.20170600<br>H 4.92593500 -0.98841500 1.30776900<br>H 4.66049400 -0.70534400 -0.50591600	• Electronic Energy = -653.142002<br>• Zero Point Energy Correction = 0.232194<br>• Thermal Correction to Energy = 0.246727<br>• Thermal Correction to Enthalpy = 0.247671<br>• Thermal Correction to Free Energy = 0.190002

Name	Anionic HAs
Cartesian Coordinate:	Thermochemical Values:

O -0.13472800 2.77295800 0.03884500<br>O -2.86215800 -1.04630300 0.16212200<br>O -2.54182400 1.61710700 0.32146900<br>C 0.79125600 -0.78760000 -0.39077400<br>C 2.01599100 -1.64899400 -0.62839700<br>C -0.47232900 -1.35641100 -0.23816800<br>C 0.92347800 0.60011600 -0.29563200<br>C -0.19130000 1.39852600 -0.06077300<br>C -1.58441900 -0.54919400 0.00062100<br>C -1.49909200 0.86312700 0.09926000<br>C 2.96497200 -1.60277000 0.53742900<br>C 1.13927800 3.38203500 -0.09897100<br>C -3.03076700 -2.45110600 0.05816700<br>C 4.21050900 -1.13935200 0.47781600<br>H 1.69180700 -2.68146200 -0.79116600<br>H 2.54405500 -1.32309400 -1.53009800<br>H -0.57755800 -2.43226200 -0.30972600<br>H 1.90722000 1.04228900 -0.40620600<br>H 2.56801100 -1.95898300 1.48692400<br>H 1.56961100 3.17930100 -1.08376100<br>H 1.82790600 3.03648200 0.67729100<br>H 0.97678000 4.45262200 0.01164300<br>H -2.46035100 -2.97640600 0.82944600<br>H -2.72853700 -2.81339000 -0.92854600<br>H -4.09289200 -2.64037500 0.20294900<br>H 4.84984300 -1.11364200 1.35370300<br>H 4.62832300 -0.77144700 -0.45540700	• Electronic Energy = -652.667789<br>• Zero Point Energy Correction = 0.219778<br>• Thermal Correction to Energy = 0.233722<br>• Thermal Correction to Enthalpy = 0.234667<br>• Thermal Correction to Free Energy = 0.178021

Name	HAs 1-OH radical
Cartesian Coordinate:	Thermochemical Values:
O -0.01170600 2.76651200 0.05383400<br>O -2.86668100 -0.93657400 0.16933700<br>O -2.40902800 1.66967200 0.34722700<br>C 0.74913100 -0.79596400 -0.43072700<br>C 1.93690100 -1.69024700 -0.68561000<br>C -0.52775600 -1.35118900 -0.27057100<br>C 0.96695300 0.59143800 -0.32329200<br>C -0.08854500 1.43381100 -0.06401600<br>C -1.60471200 -0.52846700 -0.00412700<br>C -1.44198900 0.91603200 0.11120300<br>C 2.82462400 -1.75188200 0.53102400<br>C 1.27378400 3.37089600 -0.09829000<br>C -3.13368600 -2.33741100 0.07208300<br>C 4.07688300 -1.30777200 0.56072500<br>H 1.58465600 -2.69285600 -0.93945200<br>H 2.51103800 -1.30027400 -1.53068300<br>H -0.65119800 -2.42250800 -0.36054100<br>H 1.97350600 0.97191000 -0.44674500<br>H 2.37584500 -2.17835500 1.42578800<br>H 1.67097000 3.17274600 -1.09588800<br>H 1.96212800 2.99589300 0.66183200<br>H 1.11645000 4.43793500 0.03505100<br>H -2.57552900 -2.88395600 0.83468200<br>H -2.87365800 -2.70497400 -0.92241600<br>H -4.20144700 -2.44627600 0.24238300<br>H 4.67582100 -1.36739400 1.46287700<br>H 4.54121200 -0.87424100 -0.32048500	• Electronic Energy = -652.509369<br>• Zero Point Energy Correction = 0.220225<br>• Thermal Correction to Energy = 0.234252<br>• Thermal Correction to Enthalpy = 0.235196<br>• Thermal Correction to Free Energy = 0.177810

Name	HAs 1a-CH radical
Cartesian Coordinate:	Thermochemical Values:

O 2.51664300 1.68209500 0.00005200<br>O 0.85655500 -2.65370100 -0.00003300<br>O 2.88373300 -0.92968900 0.00002000<br>C -1.01707100 0.54277600 -0.00000300<br>C -2.33394900 1.11151300 -0.00002500<br>C -0.78996600 -0.85087400 0.00001000<br>C 0.09106100 1.41771400 0.00000100<br>C 1.38615300 0.92013700 0.00001700<br>C 0.50721100 -1.33487300 0.00000100<br>C 1.60232100 -0.46130200 -0.00000400<br>C -3.55078200 0.39946500 -0.00004100<br>C 2.34601200 3.09666900 -0.00001800<br>C -0.19710200 -3.61343900 0.00003200<br>C -4.77228800 1.00309200 0.00002300<br>H -2.39138600 2.19685100 -0.00006300<br>H -1.62156700 -1.54086500 0.00004600<br>H -0.08857100 2.48489800 0.00001400<br>H -3.52014500 -0.68683300 -0.00005300<br>H 2.85698200 -1.89724900 -0.00036500<br>H 1.80996100 3.42246500 0.89452800<br>H 1.81002400 3.42238200 -0.89463300<br>H 3.34909800 3.51680500 -0.00000200<br>H -0.81440700 -3.50652400 -0.89476900<br>H -0.81434500 -3.50647300 0.89486900<br>H 0.28659900 -4.58723200 0.00004200<br>H -5.68598800 0.42217100 0.00008700<br>H -4.86129900 2.08469400 0.00002200	• Electronic Energy = -652.509606<br>• Zero Point Energy Correction = 0.218858<br>• Thermal Correction to Energy = 0.233159<br>• Thermal Correction to Enthalpy = 0.234103<br>• Thermal Correction to Free Energy = 0.177222

Name	HAs 1a-CH radical anion
Cartesian Coordinate:	Thermochemical Values:
O 2.63188000 -1.55452900 -0.00023300<br>O 0.72163800 2.73325000 0.00053000<br>O 2.84946100 1.11097600 0.00015600<br>C -0.96166800 -0.58179700 -0.00010200<br>C -2.23355600 -1.20770000 -0.00023800<br>C -0.78485900 0.82929500 0.00015400<br>C 0.20674900 -1.39234400 -0.00021500<br>C 1.46379800 -0.82924700 -0.00012400<br>C 0.47777400 1.38061300 0.00025500<br>C 1.67395000 0.58794500 0.00009500<br>C -3.49417100 -0.55987400 -0.00025100<br>C 2.51600800 -2.96956500 -0.00044500<br>C -0.40591500 3.59635000 0.00071100<br>C -4.69205700 -1.20490700 -0.00033900<br>H -2.23954200 -2.29564000 -0.00035600<br>H -1.65339800 1.47344800 0.00028800<br>H 0.08181200 -2.46836600 -0.00039000<br>H -3.50928500 0.52775600 -0.00019500<br>H 1.99200800 -3.32059300 -0.89372200<br>H 1.99201100 -3.32086100 0.89272800<br>H 3.53327600 -3.35611700 -0.00050500<br>H -1.01770500 3.44280100 0.89392200<br>H -1.01767500 3.44321800 -0.89259200<br>H -0.01025500 4.61008100 0.00095600<br>H -5.62596700 -0.65649400 -0.00035600<br>H -4.74541900 -2.28942100 -0.00039800	• Electronic Energy = -652.041649<br>• Zero Point Energy Correction = 0.206686<br>• Thermal Correction to Energy = 0.220355<br>• Thermal Correction to Enthalpy = 0.221299<br>• Thermal Correction to Free Energy = 0.165761

Name	HAs 2’-CH radical
Cartesian Coordinate:	Thermochemical Values:
O 0.31486400 2.68663000 0.01093200<br>O 2.75431400 -1.26975800 -0.31975600<br>O 2.60090400 1.42027900 -0.34868300<br>C -0.84822400 -0.81097900 0.33669700<br>C -2.12615900 -1.59158700 0.56925900<br>C 0.36085100 -1.46622900 0.13871900<br>C -0.89770800 0.58652000 0.29475700<br>C 0.25655900 1.32671300 0.06434200<br>C 1.51325200 -0.71639100 -0.08719000<br>C 1.47877100 0.67080100 -0.12816100<br>C -3.10054000 -1.40771300 -0.56172300<br>C -0.90677000 3.39565000 0.19964700<br>C 3.01292300 -2.51676000 0.18351100<br>C -4.30011700 -0.84886200 -0.43292700<br>H -1.87602900 -2.65124100 0.67066300<br>H -2.59340100 -1.26680800 1.50341300	• Electronic Energy = -652.474220<br>• Zero Point Energy Correction = 0.217747<br>• Thermal Correction to Energy = 0.232295<br>• Thermal Correction to Enthalpy = 0.233239<br>• Thermal Correction to Free Energy

H 0.41278200 -2.54856900 0.14681500<br>H -1.84817600 1.08517600 0.44089100<br>H -2.76428200 -1.74817900 -1.53952300<br>H 3.35802400 0.82424800 -0.43567700<br>H -1.32678700 3.18799000 1.18658500<br>H -1.63045500 3.13310600 -0.57554300<br>H -0.65307700 4.45034600 0.12312700<br>H 3.99608500 -2.87401700 -0.08356900<br>H 2.52669000 -2.79035000 1.11187900<br>H -4.96404300 -0.72945800 -1.28220700<br>H -4.65500600 -0.49642300 0.53161700	= 0.174914

Name	HAs 2'-CH radical anion
Cartesian Coordinate:	Thermochemical Values:
O 0.27689000 2.71508600 0.00799400<br>O 2.81136800 -1.23835100 -0.33599400<br>O 2.62050300 1.46517000 -0.38466600<br>C -0.79891700 -0.81847100 0.33279600<br>C -2.05934100 -1.62741900 0.56509200<br>C 0.42951300 -1.43287800 0.12519500<br>C -0.86569900 0.58158000 0.29350800<br>C 0.27187600 1.33918500 0.05536800<br>C 1.56672900 -0.65927200 -0.10300600<br>C 1.55625500 0.75181300 -0.16000300<br>C -3.04564400 -1.45947400 -0.55784800<br>C -0.96250700 3.37516900 0.21506100<br>C 3.06506000 -2.46077600 0.20973600<br>C -4.25747000 -0.92786600 -0.42242100<br>H -1.78552700 -2.68307400 0.65402800<br>H -2.53555100 -1.32929300 1.50458300<br>H 0.50742900 -2.51498500 0.12833200<br>H -1.82524900 1.06241000 0.44751500<br>H -2.70599500 -1.78331500 -1.54050200<br>H -1.36811000 3.14955600 1.20526900<br>H -1.69110800 3.09382000 -0.55038400<br>H -0.75358400 4.44083100 0.14286200<br>H 4.04854500 -2.83363200 -0.03857800<br>H 2.58345900 -2.70214000 1.15040200<br>H -4.92625600 -0.81467000 -1.26887000<br>H -4.61726900 -0.59029800 0.54578700	• Electronic Energy<br>= -652.002817<br>• Zero Point Energy Correction<br>= 0.205291<br>• Thermal Correction to Energy<br>= 0.219311<br>• Thermal Correction to Enthalpy<br>= 0.220255<br>• Thermal Correction to Free Energy<br>= 0.162898

Name	HAs radical cation
Cartesian Coordinate:	Thermochemical Values:
O -0.32070900 2.76350500 0.08687300<br>O -2.64117900 -1.23551000 0.17361600<br>O -2.50265700 1.38141100 0.40957500<br>C 0.90234800 -0.63878700 -0.54171100<br>C 2.18403500 -1.36744600 -0.82848800<br>C -0.28342300 -1.37678500 -0.36274900<br>C 0.94641800 0.76206700 -0.39386600<br>C -0.19839400 1.45420400 -0.07776600<br>C -1.44154600 -0.71309500 -0.03829500<br>C -1.42382700 0.72069300 0.10617800<br>C 2.88742000 -1.65018900 0.47947200<br>C 0.85867300 3.56531400 -0.07280800<br>C -2.78082800 -2.66156800 0.07796400<br>C 4.04887300 -1.10351000 0.82072300<br>H 1.96125200 -2.30607000 -1.34071400<br>H 2.82499100 -0.75251600 -1.46265700<br>H -0.26728700 -2.45175800 -0.48430100<br>H 1.88888500 1.27539400 -0.53257000<br>H 2.37666000 -2.33012300 1.15720700<br>H -3.26346600 0.77838700 0.51172100<br>H 1.24648600 3.45641500 -1.08676400<br>H 1.61106500 3.26877900 0.65962700<br>H 0.54014600 4.58833000 0.10341900<br>H -2.13202300 -3.14316400 0.81067600<br>H -2.53056900 -2.98780300 -0.93231400<br>H -3.82399700 -2.86715000 0.29737400<br>H 4.51925100 -1.33108600 1.77098800<br>H 4.56647000 -0.41827200 0.15587200	• Electronic Energy<br>= -652.936122<br>• Zero Point Energy Correction<br>= 0.232918<br>• Thermal Correction to Energy<br>= 0.247273<br>• Thermal Correction to Enthalpy<br>= 0.248218<br>• Thermal Correction to Free Energy<br>= 0.190404

Name	HAs HAT 1a-CH···NOO$^{\bullet}$ Transition States Structure
Cartesian Coordinate:	Thermochemical Values:
O 1.70989400 2.58268800 0.46631900<br>O 2.63085900 -1.85450500 -0.48637500<br>O 3.34265600 0.55312400 0.32335700<br>C -0.57804200 -0.03679400 -0.77002600<br>C -1.95975500 -0.31386900 -1.07995700<br>C 0.33976300 -1.12405800 -0.82917000<br>C -0.14299200 1.23758700 -0.32219600<br>C 1.16842800 1.43114600 0.03814900<br>C 1.64754300 -0.93519200 -0.47146000<br>C 2.08522500 0.34406500 -0.02991700<br>C -2.93835900 0.75369800 -1.36851600<br>C 0.84277800 3.71422300 0.57435200<br>C 2.28374000 -3.17924900 -0.89820500<br>C -4.01641700 0.55719700 -2.13046200<br>H -2.09621700 -1.21219500 -1.68515300<br>H -0.01655100 -2.09030200 -1.16173600<br>H -0.84334800 2.05788700 -0.26874300<br>H -2.79385800 1.72292200 -0.90007000<br>H 3.85188400 -0.27093700 0.23579600<br>H 0.04085000 3.51099700 1.28681000<br>H 0.42580300 3.96756300 -0.40255800<br>H 1.46574600 4.52775400 0.93599600<br>H 1.91345400 -3.16865200 -1.92507700<br>H 1.52976300 -3.59552300 -0.22738600<br>H 3.20114200 -3.75799700 -0.83673600<br>H -4.73489700 1.35052100 -2.30084900<br>H -4.20424400 -0.40120700 -2.60549500<br>H -2.32038500 -0.74215800 0.04055600<br>O -2.74459400 -1.20657400 1.32962400<br>N -1.74495400 -0.97019500 2.07686500<br>O -1.88830800 -1.29971100 3.24220600	• Electronic Energy = -858.171761<br>• Zero Point Energy Correction = 0.237292<br>• Thermal Correction to Energy = 0.255518<br>• Thermal Correction to Enthalpy = 0.256462<br>• Thermal Correction to Free Energy = 0.187985

Name	HAs HAT 1a-CH···NOO$^{\bullet}$ Reactant Complex
Cartesian Coordinate:	Thermochemical Values:
O 0.01139700 2.94726300 -0.13295900<br>O 2.69877600 -0.82395200 -0.46154900<br>O 2.36740700 1.76728900 0.05999200<br>C -0.92262800 -0.49770200 -1.09505000<br>C -2.15102800 -1.31948900 -1.42419200<br>C 0.32608200 -1.10688200 -0.99862900<br>C -1.06554100 0.86227000 -0.81402400<br>C 0.04065500 1.61887500 -0.43536200<br>C 1.43173300 -0.34665400 -0.61838100<br>C 1.29465200 1.01235700 -0.33291200<br>C -2.87677000 -1.71010900 -0.16411300<br>C -1.25500200 3.59786700 -0.19169900<br>C 2.88645200 -2.23307900 -0.57399100<br>C -4.10988000 -1.31857900 0.14232100<br>H -2.82308400 -0.74762000 -2.06870600<br>H 0.42875500 -2.16350100 -1.21127500<br>H -2.04689700 1.31567400 -0.87977600<br>H -2.31777600 -2.33535400 0.53033900<br>H 3.14490100 1.19226800 0.09629100<br>H -1.95694300 3.14265300 0.51089800<br>H -1.66389900 3.55946100 -1.20401600<br>H -1.07139700 4.63196700 0.09050000<br>H 2.66127500 -2.57198300 -1.58750500<br>H 2.25730500 -2.76150600 0.14634800<br>H 3.93552000 -2.41278900 -0.35123500<br>H -4.58401800 -1.61518900 1.07163300<br>H -4.68391700 -0.68919400 -0.53212500<br>H -1.84449100 -2.21996100 -1.96389200<br>O -0.58662800 -0.59736100 2.10642900<br>N 0.58657100 -0.48996800 2.20009700<br>O 1.48230600 -1.26065700 2.27650000	• Electronic Energy = -858.199471<br>• Zero Point Energy Correction = 0.242431<br>• Thermal Correction to Energy = 0.261596<br>• Thermal Correction to Enthalpy = 0.262540<br>• Thermal Correction to Free Energy = 0.191426

Name	HAs HAT 1a-CH···NOO$^{\bullet}$ Product Complex
Cartesian Coordinate:	Thermochemical Values:

O 1.04974800 2.77510000 -0.11633200<br>O 2.55785900 -1.58455400 -0.63202800<br>O 3.00347600 1.00842700 -0.22560600<br>C -0.92049500 -0.30394500 -0.73554400<br>C -2.25833200 -0.80614100 -0.86626700<br>C 0.14591400 -1.22530400 -0.79153400<br>C -0.63670600 1.06050900 -0.51083800<br>C 0.67390100 1.48558300 -0.34893400<br>C 1.44997400 -0.78953500 -0.62056900<br>C 1.72579500 0.56526900 -0.40458200<br>C -3.44710300 -0.05294000 -0.78686700<br>C 0.01166000 3.74419600 -0.00383700<br>C 2.35270500 -2.98901800 -0.76742100<br>C -4.68886300 -0.60221900 -0.90560500<br>H -2.35738000 -1.87646700 -1.02832700<br>H -0.07065300 -2.27389600 -0.94986600<br>H -1.44150300 1.77922300 -0.45468000<br>H -3.37815700 1.01894300 -0.62174700<br>H 3.59942800 0.24663600 -0.26861100<br>H -0.65548600 3.50178100 0.82688600<br>H -0.55937000 3.81196700 -0.93271500<br>H 0.50945100 4.69147400 0.19009800<br>H 1.88134400 -3.21854600 -1.72558300<br>H 1.73704600 -3.36608000 0.05262600<br>H 3.34112400 -3.44017500 -0.72582300<br>H -5.58181400 0.00639100 -0.83843400<br>H -4.81452500 -1.66719800 -1.07252200<br>H -2.23388000 -0.96311800 2.26752300<br>O -1.32788700 -1.31539200 2.27438800<br>N -0.51608500 -0.22205100 2.37078900<br>O 0.61508800 -0.53549500 2.34178300	• Electronic Energy = -858.209994<br>• Zero Point Energy Correction = 0.241205<br>• Thermal Correction to Energy = 0.260307<br>• Thermal Correction to Enthalpy = 0.261251<br>• Thermal Correction to Free Energy = 0.191654

Name	Anionic HAs HAT 1a-CH···NOO$^{\bullet}$ Transition States Structure
Cartesian Coordinate:	Thermochemical Values:
O 1.53895500 2.71439800 -0.00893600<br>O 3.00862800 -1.72800600 0.22224600<br>O 3.41776000 0.89254700 0.46249900<br>C -0.31966800 -0.40233000 -0.72514800<br>C -1.62512400 -0.90734500 -1.05162600<br>C 0.71854700 -1.33595400 -0.44538600<br>C -0.05112900 0.98511800 -0.57028700<br>C 1.19065400 1.41531900 -0.17535000<br>C 1.96074300 -0.91185400 -0.05189300<br>C 2.27508300 0.49483000 0.10544800<br>C -2.66860900 -0.08195200 -1.66651500<br>C 0.53780600 3.69834600 -0.24973700<br>C 2.79298600 -3.13032800 0.10044600<br>C -3.71220300 -0.56836100 -2.35112400<br>H -1.63586800 -1.95290200 -1.36329700<br>H 0.49991900 -2.39015800 -0.55967900<br>H -0.83423300 1.70365900 -0.76479700<br>H -2.61404900 0.99399200 -1.51778700<br>H -0.30753800 3.56307600 0.42923100<br>H 0.19321800 3.65335100 -1.28556800<br>H 1.01192600 4.65862400 -0.06094500<br>H 2.52132100 -3.39135400 -0.92520500<br>H 2.01015900 -3.46179800 0.78684500<br>H 3.73693000 -3.60224300 0.36282900<br>H -4.47670100 0.08773100 -2.75053000<br>H -3.82479300 -1.63436600 -2.52763100<br>H -2.10710700 -1.06193500 0.23306400<br>O -2.57481200 -1.16273100 1.38569500<br>N -2.69849600 0.05279900 1.79549400<br>O -3.12530900 0.15026800 2.92125100	• Electronic Energy = -857.727456<br>• Zero Point Energy Correction = 0.224143<br>• Thermal Correction to Energy = 0.242184<br>• Thermal Correction to Enthalpy = 0.243128<br>• Thermal Correction to Free Energy = 0.174350

Name	Anionic HAs HAT 1a-CH···NOO$^{\bullet}$ Reactant Complex
Cartesian Coordinate:	Thermochemical Values:

O -0.05073400 2.64443400 -0.72701700<br>O 3.05041700 -0.73340000 0.20712900<br>O 2.35479400 1.82141900 0.04235600<br>C -0.38794800 -0.99614800 -1.04884400<br>C -1.43996900 -2.02831900 -1.36736600<br>C 0.87860600 -1.40905000 -0.60487500<br>C -0.73317100 0.36390600 -1.12335400<br>C 0.17610200 1.32391200 -0.74280200<br>C 1.81480600 -0.46649400 -0.23540300<br>C 1.51531500 0.96026500 -0.29167000<br>C -2.23116600 -2.32773300 -0.11759700<br>C -1.35768800 3.09205600 -1.09197700<br>C 3.42879000 -2.10549100 0.32884000<br>C -3.49218100 -1.95263600 0.06902900<br>H -2.10952700 -1.64779700 -2.14141900<br>H 1.09944000 -2.46762800 -0.55772200<br>H -1.73185600 0.63184400 -1.44468500<br>H -1.69284000 -2.84605600 0.67319900<br>H -2.10445200 2.66247200 -0.42078900<br>H -1.57783200 2.82041900 -2.12639200<br>H -1.33964300 4.17374600 -0.98742900<br>H 3.39536500 -2.59727300 -0.64533900<br>H 2.77083700 -2.61881800 1.03293700<br>H 4.44747900 -2.09876400 0.70736700<br>H -4.01204100 -2.16401800 0.99722100<br>H -4.04231100 -1.42235300 -0.70328700<br>H -0.95943700 -2.94005300 -1.73080500<br>O 0.00696800 0.73002800 2.28243100<br>N -1.09704300 0.15969500 2.21740400<br>O -2.04805200 0.87136800 1.84278000	• Electronic Energy<br>= -857.764752<br>• Zero Point Energy Correction<br>= 0.229590<br>• Thermal Correction to Energy<br>= 0.248168<br>• Thermal Correction to Enthalpy<br>= 0.249112<br>• Thermal Correction to Free Energy<br>= 0.180343

Name	Anionic HAs HAT 1a-CH···NOO$^{\bullet}$ Product Complex
Cartesian Coordinate:	Thermochemical Values:
O 0.87876600 2.70367600 -0.67875100<br>O 3.22450900 -1.20630400 0.42995700<br>O 3.09212600 1.44946000 0.12955600<br>C -0.30014900 -0.82014400 -0.70295700<br>C -1.44216800 -1.63040800 -0.91728200<br>C 0.90869700 -1.43213200 -0.26180700<br>C -0.30745400 0.59642300 -0.85179900<br>C 0.81662700 1.33786700 -0.57043400<br>C 2.02909200 -0.68108600 0.00763400<br>C 2.04998800 0.74809200 -0.12715900<br>C -2.73099900 -1.18908400 -1.32801800<br>C -0.30857000 3.37829000 -1.06949900<br>C 3.28643500 -2.61384600 0.60893100<br>C -3.79544000 -2.00953400 -1.52113400<br>H -1.31538000 -2.70023600 -0.76390900<br>H 0.92046800 -2.50848000 -0.14112500<br>H -1.20986600 1.09144300 -1.18237600<br>H -2.87773500 -0.12481400 -1.49819800<br>H -1.11566600 3.19462600 -0.35446600<br>H -0.62612200 3.06735600 -2.06843100<br>H -0.06400500 4.43851700 -1.07922200<br>H 3.09298900 -3.13757600 -0.33119000<br>H 2.57025400 -2.94417800 1.36620600<br>H 4.29826100 -2.83124700 0.94504000<br>H -4.75609300 -1.61941500 -1.83411200<br>H -3.71321700 -3.08121000 -1.36695700<br>H -1.51748400 -0.98061200 1.23036200<br>O -1.73100400 -0.49729400 2.05622300<br>N -2.42010100 0.59591100 1.64732800<br>O -2.71215400 1.28269000 2.55654300	• Electronic Energy<br>= -857.741404<br>• Zero Point Energy Correction<br>= 0.228669<br>• Thermal Correction to Energy<br>= 0.247296<br>• Thermal Correction to Enthalpy<br>= 0.248240<br>• Thermal Correction to Free Energy<br>= 0.178797

Name	HAs RAF C-4··· NO$^{\bullet}$ Product
Cartesian Coordinate:	Thermochemical Values:
O -1.41021800 2.43401200 0.05051500<br>O 2.84677400 0.61682600 -0.24039500<br>O 1.18555400 2.68299600 -0.24722500<br>C -0.95698900 -2.40406400 0.11290300<br>C 1.07475600 -0.93018000 0.33003700<br>C -1.22809000 0.06559000 0.49266400<br>C -0.70122500 1.28910600 0.21741900<br>C 1.55876900 0.31152900 0.06141500<br>C 0.70398600 1.45188800 0.03637000	• Electronic Energy<br>= -782.975256<br>• Zero Point Energy Correction<br>= 0.238568<br>• Thermal Correction to Energy

C -1.02775900 -2.32930000 -1.38357700<br>C -2.82449500 2.34421100 0.20584100<br>C 3.78852700 -0.45459800 -0.23886300<br>C -2.16193000 -2.34381500 -2.07747000<br>H -0.33132000 -3.25030800 0.41468400<br>H -1.95639300 -2.55720700 0.52860900<br>H 1.71447800 -1.80332600 0.33823100<br>H -2.29401100 -0.08051100 0.61020200<br>H -0.07764900 -2.25072300 -1.90848900<br>H 2.14174900 2.61903600 -0.39234300<br>H -3.07918700 2.00533000 1.21268100<br>H -3.24979500 1.66376100 -0.53542100<br>H -3.20585100 3.34984100 0.04632300<br>H 3.52133000 -1.19741100 -0.99349700<br>H 3.83191500 -0.92101700 0.74769700<br>H 4.74929100 -0.00779300 -0.48182200<br>H -2.16329900 -2.28581100 -3.16045600<br>H -3.12519300 -2.41797500 -1.58037800<br>C -0.35629400 -1.12708100 0.70827600<br>N -0.28209500 -1.24115600 2.26674300<br>O -0.65516800 -2.27947400 2.70870800	= 0.255343<br>• Thermal Correction to Enthalpy<br>= 0.256287<br>• Thermal Correction to Free Energy<br>= 0.192933

Name	Anionic HAs RAF C-4··· NO$^{\bullet}$ Product
Cartesian Coordinate:	Thermochemical Values:
O -1.53960800 2.31836000 -0.15901900<br>O 2.78618200 0.48442300 -0.40930700<br>O 1.01987700 2.40577300 -0.78999200<br>C -0.97695400 -2.31910800 0.10968300<br>C 1.04494200 -0.91645000 0.50494700<br>C -1.24521700 0.07301800 0.67195800<br>C -0.80621300 1.20608400 0.11002600<br>C 1.50390000 0.21964700 -0.03789400<br>C 0.60913300 1.36739600 -0.29092100<br>C -0.93739200 -2.14059200 -1.37725000<br>C -2.92496000 2.25089400 0.16038000<br>C 3.72977300 -0.56063600 -0.20275300<br>C -2.01271200 -1.94862700 -2.13708900<br>H -0.39874200 -3.20064200 0.39893500<br>H -2.00816900 -2.45743100 0.44813000<br>H 1.70539800 -1.75425800 0.69757700<br>H -2.28084600 -0.03726600 0.97603700<br>H 0.04672300 -2.15729800 -1.84222100<br>H -3.06636000 2.09985100 1.23364800<br>H -3.40666600 1.43996300 -0.39274400<br>H -3.35019800 3.20640900 -0.13730900<br>H 3.45344700 -1.44817000 -0.77822800<br>H 3.79632100 -0.81549700 0.85815300<br>H 4.68581100 -0.17717800 -0.55133200<br>H -1.93354500 -1.81620400 -3.21073800<br>H -3.00926800 -1.92149400 -1.70385700<br>C -0.37963400 -1.11672500 0.89746600<br>N -0.45152000 -1.42930000 2.38134600<br>O 0.22757300 -2.51031500 2.65988300	• Electronic Energy<br>= -782.533440<br>• Zero Point Energy Correction<br>= 0.227293<br>• Thermal Correction to Energy<br>= 0.243517<br>• Thermal Correction to Enthalpy<br>= 0.244462<br>• Thermal Correction to Free Energy<br>= 0.182194

Name	HAs RAF C-2a··· NO$^{\bullet}$ Product
Cartesian Coordinate:	Thermochemical Values:
O -1.73728300 2.41881600 0.15872400<br>O -2.02330000 -2.21780800 0.11650800<br>O -3.09862100 0.18619900 0.53266700<br>C 0.85243500 -0.06165200 -0.74179100<br>C 2.29625600 -0.13624600 -1.16373900<br>C 0.12234700 -1.23212100 -0.53644600<br>C 0.25835600 1.18186800 -0.51992800<br>C -1.06495800 1.25837400 -0.08951100<br>C -1.20201100 -1.15113100 -0.11244400<br>C -1.79790800 0.08879900 0.11505600<br>C -1.01358200 3.63503500 -0.00479100<br>C -1.46959600 -3.51970600 -0.05802700<br>C 3.14690900 1.09772700 0.90479100<br>H 2.48639500 -1.04785400 -1.73390300<br>H 2.55628100 0.71704800 -1.79207400<br>H 0.58636300 -2.19461700 -0.71165100<br>H 0.83456900 2.08284600 -0.68762900<br>H -3.44909500 -0.70890800 0.64348200	• Electronic Energy<br>= -782.992048<br>• Zero Point Energy Correction<br>= 0.238305<br>• Thermal Correction to Energy<br>= 0.255327<br>• Thermal Correction to Enthalpy<br>= 0.256271<br>• Thermal Correction to Free Energy<br>= 0.192220

H -0.68618200 3.75610300 -1.04005400<br>H -0.14992800 3.66450300 0.66410600<br>H -1.70704800 4.43134500 0.25542200<br>H -0.62724700 -3.67321900 0.62039700<br>H -1.14846500 -3.66619500 -1.09171100<br>H -2.26797900 -4.21792800 0.18163600<br>H 2.57660200 1.07278100 1.82151700<br>H 3.55137300 2.03741400 0.55492100<br>C 3.28972200 -0.09431700 0.04567300<br>H 4.28901700 -0.16133700 -0.39377600<br>N 3.09504300 -1.40071200 0.71963300<br>O 2.50053400 -1.34906800 1.75749500	

Name	Anionic HAs RAF C-2a·· $NO^{\bullet}$ Product
Cartesian Coordinate:	Thermochemical Values:
O -1.41021800 2.43401200 0.05051500<br>O 2.84677400 0.61682600 -0.24039500<br>O 1.18555400 2.68299600 -0.24722500<br>C -0.95698900 -2.40406400 0.11290300<br>C 1.07475600 -0.93018000 0.33003700<br>C -1.22809000 0.06559000 0.49266400<br>C -0.70122500 1.28910600 0.21741900<br>C 1.55876900 0.31152900 0.06141500<br>C 0.70398600 1.45188800 0.03637000<br>C -1.02775900 -2.32930000 -1.38357700<br>C -2.82449500 2.34421100 0.20584100<br>C 3.78852700 -0.45459800 -0.23886300<br>C -2.16193000 -2.34381500 -2.07747000<br>H -0.33132000 -3.25030800 0.41468400<br>H -1.95639300 -2.55720700 0.52860900<br>H 1.71447800 -1.80332600 0.33823100<br>H -2.29401100 -0.08051100 0.61020200<br>H -0.07764900 -2.25072300 -1.90848900<br>H 2.14174900 2.61903600 -0.39234300<br>H -3.07918700 2.00533000 1.21268100<br>H -3.24979500 1.66376100 -0.53542100<br>H -3.20585100 3.34984100 0.04632300<br>H 3.52133000 -1.19741100 -0.99349700<br>H 3.83191500 -0.92101700 0.74769700<br>H 4.74929100 -0.00779300 -0.48182200<br>H -2.16329900 -2.28581100 -3.16045600<br>H -3.12519300 -2.41797500 -1.58037800<br>C -0.35629400 -1.12708100 0.70827600<br>N -0.28209500 -1.24115600 2.26674300<br>O -0.65516800 -2.27947400 2.70870800	• Electronic Energy<br>= -782.518673<br>• Zero Point Energy Correction<br>= 0.225485<br>• Thermal Correction to Energy<br>= 0.242081<br>• Thermal Correction to Enthalpy<br>= 0.243025<br>• Thermal Correction to Free Energy<br>= 0.179521

Name	HAs RAF C-3a·· $NO^{\bullet}$ Product
Cartesian Coordinate:	Thermochemical Values:
O -0.64566700 2.94578300 -0.10654600<br>O -2.75727200 -1.13966300 0.54338800<br>O -2.73789400 1.52270400 0.65877400<br>C 0.62415900 -0.45887900 -0.77774400<br>C 1.85982400 -1.18738800 -1.25650100<br>C -0.48460600 -1.19925800 -0.36497600<br>C 0.60726100 0.93097400 -0.70201100<br>C -0.52498300 1.59188800 -0.21925200<br>C -1.60991600 -0.53728400 0.11533800<br>C -1.63526400 0.85720200 0.19017400<br>C 2.54463400 -1.95858700 -0.17387400<br>C 0.46706600 3.73479600 -0.51818300<br>C -2.80342700 -2.56367200 0.49889600<br>H 1.59863600 -1.87932900 -2.06270500<br>H 2.55766100 -0.45979400 -1.69437100<br>H -0.45942400 -2.28081400 -0.42032200<br>H 1.47747200 1.49218200 -1.01737300<br>H 3.15983500 -2.80653100 -0.45303700<br>H -3.41083400 0.86735200 0.89125100<br>H 0.68378500 3.57769400 -1.57743500<br>H 1.35082000 3.50260100 0.08071000<br>H 0.17466900 4.76933200 -0.35375000<br>H -2.02696400 -2.99347800 1.13563300<br>H -2.68553300 -2.92032600 -0.52670100<br>H -3.78473600 -2.84256300 0.87527400<br>C 2.70163200 -1.43823300 1.20235900<br>H 2.79173200 -2.22578100 1.95088300	• Electronic Energy<br>= -782.995212<br>• Zero Point Energy Correction<br>= 0.239644<br>• Thermal Correction to Energy<br>= 0.256389<br>• Thermal Correction to Enthalpy<br>= 0.257333<br>• Thermal Correction to Free Energy<br>= 0.192367

H 1.92540800 -0.71811400 1.46394200<br>N 3.99391600 -0.67057100 1.16532500<br>O 4.90901300 -1.24665100 1.68518800	

Name	Anionic HAs RAF C-3a·· NO$^{\bullet}$ Product
Cartesian Coordinate:	Thermochemical Values:
O -0.96427600 2.70802300 0.15421300<br>O -3.04918600 -1.50149500 0.18048300<br>O -3.15410100 1.17509600 0.42706200<br>C 0.49916800 -0.64218700 -0.43809300<br>C 1.82833500 -1.28531200 -0.75913700<br>C -0.65279900 -1.41042200 -0.27956500<br>C 0.41291000 0.74498400 -0.29249200<br>C -0.80707500 1.34653400 -0.00121200<br>C -1.87226700 -0.79970300 0.01156100<br>C -2.00970000 0.60434900 0.16072100<br>C 2.88367400 -1.03985700 0.26490200<br>C 0.19525800 3.51479400 0.01894600<br>C -2.98741600 -2.91319500 0.05291600<br>H 1.68663100 -2.36974100 -0.87786200<br>H 2.19893000 -0.93619500 -1.73232800<br>H -0.58752000 -2.48631900 -0.38751500<br>H 1.30866600 1.34341000 -0.41413700<br>H 2.60391500 -0.74575900 1.27023700<br>H 0.62437900 3.42455000 -0.98291000<br>H 0.95022900 3.24868500 0.76413300<br>H -0.12898600 4.54090600 0.18238600<br>H -2.31542800 -3.34810800 0.79816100<br>H -2.65900000 -3.20555500 -0.94845300<br>H -3.99927800 -3.27711300 0.22194100<br>C 4.32133600 -1.07713700 -0.07036500<br>H 4.51238700 -1.60210200 -1.00654900<br>H 4.95235800 -1.44497800 0.73882100<br>N 4.65346100 0.37091300 -0.31297300<br>O 5.23198300 0.88749700 0.60462800	• Electronic Energy = -782.520636<br>• Zero Point Energy Correction = 0.226579<br>• Thermal Correction to Energy = 0.242928<br>• Thermal Correction to Enthalpy = 0.243872<br>• Thermal Correction to Free Energy = 0.180041

Name	HAs RAF C-1···NOO$^{\bullet}$ (Model 1) Product
Cartesian Coordinate:	Thermochemical Values:
O 0.24264900 2.45835000 -0.72102400<br>O 1.92172000 -1.90724700 -0.67430100<br>O 1.68649800 0.52619100 -1.87445100<br>C -1.37241900 -0.66282200 0.44070000<br>C -2.69513500 -1.13179500 0.99085600<br>C -0.33104400 -1.57763100 0.17482900<br>C -1.20470900 0.71182200 0.13318800<br>C -0.03731700 1.18138700 -0.38717800<br>C 0.85534100 -1.14732700 -0.34646400<br>C -3.77867400 -1.02944000 -0.05173100<br>C -0.79348100 3.42037300 -0.53015700<br>C 1.79959100 -3.31863500 -0.49718600<br>C -4.82133600 -0.20886200 0.03356500<br>H -2.59734300 -2.16874800 1.32252100<br>H -2.97403500 -0.52270900 1.85640300<br>H -0.48773100 -2.62926500 0.38175000<br>H -2.03245400 1.38766400 0.31407000<br>H -3.65494400 -1.66116900 -0.92927700<br>H 2.33540400 -0.16901300 -2.05949900<br>H -1.06877200 3.47825400 0.52527700<br>H -1.66797800 3.16205700 -1.13147900<br>H -0.38250100 4.37095900 -0.86018700<br>H 0.97637200 -3.70536500 -1.10162400<br>H 1.63542000 -3.55421800 0.55635400<br>H 2.74173700 -3.74438300 -0.83223300<br>H -5.56745500 -0.15773800 -0.75198100<br>H -4.96042500 0.43523100 0.89740700<br>C 1.14501900 0.29545700 -0.61386200<br>O 2.26666000 0.73700500 0.29099700<br>N 1.93901800 0.60196200 1.59996900<br>O 2.82454300 0.94439500 2.29544800	• Electronic Energy = -858.183255<br>• Zero Point Energy Correction = 0.243009<br>• Thermal Correction to Energy = 0.260794<br>• Thermal Correction to Enthalpy = 0.261738<br>• Thermal Correction to Free Energy = 0.195888

Name	HAs RAF C-2···NOO$^{\bullet}$ (Model 1) Product

Cartesian Coordinate:	Thermochemical Values:
O -0.21463400 -1.84312100 -1.59358200<br>O -2.82663400 1.51619600 -0.07058200<br>O -2.56644200 -1.10422200 -0.46464800<br>C 0.89127000 1.16636600 -0.10293000<br>C 2.09683300 2.06610800 0.07380000<br>C -0.39939500 1.79961400 -0.01843900<br>C 1.00999900 -0.16527900 -0.30022800<br>C -1.54957200 1.04149600 -0.13555100<br>C -1.47351000 -0.33212500 -0.34646500<br>C 3.40092100 1.44960700 -0.33362500<br>C 1.00816700 -2.49738900 -1.95278700<br>C -2.98947500 2.91806700 0.13508200<br>C 4.42767200 1.26127100 0.48895900<br>H 1.92235400 2.97162100 -0.51816000<br>H 2.14413800 2.38310300 1.12103600<br>H -0.44917600 2.86879100 0.13992700<br>H 1.97874600 -0.64919000 -0.32701600<br>H 3.48556600 1.15081600 -1.37696600<br>H -3.35817400 -0.54829000 -0.38905000<br>H 1.44240100 -3.01342600 -1.09455300<br>H 1.72289800 -1.78198000 -2.36306400<br>H 0.74037300 -3.22371700 -2.71702700<br>H -2.53669400 3.48075000 -0.68413200<br>H -2.54666700 3.21981800 1.08680200<br>H -4.06224200 3.09358700 0.15493100<br>H 5.35778100 0.82393000 0.14276600<br>H 4.36847500 1.54381400 1.53635500<br>C -0.17529900 -1.05457800 -0.44158200<br>O -0.13981800 -2.10584600 0.63988400<br>N -0.13302500 -1.55390300 1.87421800<br>O -0.09800700 -2.38316400 2.71032900	• Electronic Energy<br>= -858.174261<br>• Zero Point Energy Correction<br>= 0.242188<br>• Thermal Correction to Energy<br>= 0.260235<br>• Thermal Correction to Enthalpy<br>= 0.261179<br>• Thermal Correction to Free Energy<br>= 0.194653

Name	HAs RAF C-3···NOO• (Model 1) Product
Cartesian Coordinate:	Thermochemical Values:
O -0.05404000 2.49528200 -0.86203400<br>O 3.20230000 -0.73948900 -0.03573800<br>O 2.50982900 1.72597500 -0.74684300<br>C -0.45535900 -1.13735500 0.06837500<br>C -1.55917200 -2.14325800 0.26747400<br>C 0.86896800 -1.47049100 0.14403900<br>C 0.22133700 1.19160600 -0.56245200<br>C 1.87165800 -0.51321800 -0.09604500<br>C 1.52538300 0.81663200 -0.47165300<br>C -2.31305900 -2.38088400 -1.01591000<br>C -1.29672700 2.76014900 -1.52050600<br>C 3.64587900 -2.05542700 0.29989400<br>C -3.56467000 -1.98531900 -1.22727200<br>H -1.12168100 -3.07937200 0.62297600<br>H -2.25733100 -1.78228800 1.03058400<br>H 1.14320700 -2.49287500 0.37528700<br>H -1.75930300 -2.88127300 -1.80774700<br>H 3.36690800 1.28268300 -0.67667300<br>H -2.14226200 2.56240700 -0.85847400<br>H -1.38198100 2.16242300 -2.43086500<br>H -1.27657500 3.81700200 -1.77636500<br>H 3.30062800 -2.77427700 -0.44555500<br>H 3.28541300 -2.33587300 1.29139800<br>H 4.73182700 -2.01088100 0.29812600<br>H -4.06221900 -2.15893500 -2.17531300<br>H -4.13112200 -1.47781100 -0.45102300<br>C -0.88113100 0.26777800 -0.19466500<br>O -1.61230900 0.82287300 1.00607900<br>H -1.70347700 0.29733300 -0.91528500<br>N -0.81054700 0.86037000 2.09168200<br>O -1.38288600 1.28884200 3.02972000	• Electronic Energy<br>= -858.174567<br>• Zero Point Energy Correction<br>= 0.242880<br>• Thermal Correction to Energy<br>= 0.261033<br>• Thermal Correction to Enthalpy<br>= 0.261977<br>• Thermal Correction to Free Energy<br>= 0.195221

Name	HAs RAF C-4···NOO• (Model 1) Product
Cartesian Coordinate:	Thermochemical Values:
O -1.04106800 2.68439000 0.37821300<br>O 2.87739900 0.42146700 -0.67090800<br>O 1.53040000 2.63724100 -0.15714700<br>C -1.31089300 -1.95177400 -0.87849100<br>C 0.92471600 -0.96076400 -0.30068900	• Electronic Energy<br>= -858.179909<br>• Zero Point Energy Correction

C -1.19235800 0.27568700 0.26718000<br>C -0.50972800 1.44699300 0.19870200<br>C 1.56775100 0.23106300 -0.35968700<br>C 0.88478800 1.45663800 -0.09924300<br>C -1.35409800 -1.40287900 -2.27383800<br>C -2.43216400 2.74565500 0.68202400<br>C 3.65366000 -0.74176800 -0.95053500<br>C -2.47544600 -1.06323800 -2.90148400<br>H -0.82431000 -2.93281600 -0.87431700<br>H -2.32377600 -2.06450900 -0.48331600<br>H 1.42815800 -1.89566100 -0.51206900<br>H -2.25563600 0.24566900 0.46804700<br>H -0.39726700 -1.28737100 -2.77895700<br>H 2.46074200 2.47432000 -0.37780900<br>H -2.64625900 2.21357600 1.61193200<br>H -3.02267800 2.32170600 -0.13363600<br>H -2.66602200 3.80122200 0.79748800<br>H 3.25253900 -1.26653500 -1.82047000<br>H 3.66716800 -1.40768400 -0.08489200<br>H 4.65911800 -0.38671600 -1.16214700<br>H -2.46138400 -0.67686000 -3.91469600<br>H -3.44371200 -1.16633600 -2.41968600<br>C -0.51663100 -1.03899200 0.07242400<br>O -0.62989900 -1.82871100 1.35988900<br>N -0.00038700 -1.21746300 2.37882500<br>O -0.09452500 -1.84307200 3.37677500	= 0.243004<br>• Thermal Correction to Energy<br>= 0.260723<br>• Thermal Correction to Enthalpy<br>= 0.261667<br>• Thermal Correction to Free Energy<br>= 0.196531

Name	HAs RAF C-2a···NOO• (Model 1) Product
Cartesian Coordinate:	Thermochemical Values:
O 2.57475800 -2.13753500 0.54285600<br>O 2.11740100 2.44363200 -0.07166900<br>O 3.49960800 0.32518600 0.77204000<br>C -0.23478300 -0.26272200 -0.95893900<br>C -1.60468000 -0.49422300 -1.54755000<br>C 0.26314100 1.03213800 -0.82968500<br>C 0.51299600 -1.35155800 -0.50849200<br>C 1.76266100 -1.14745100 0.07359700<br>C 1.51504300 1.23125300 -0.25066600<br>C 2.26598200 0.14768400 0.20281300<br>C 2.09637300 -3.47513100 0.43458700<br>C 1.40751200 3.59891600 -0.51082400<br>C -4.00579700 -1.05013100 -1.03440900<br>H -1.92896400 0.37439300 -2.12584700<br>H -1.59155400 -1.35798300 -2.21633900<br>H -0.32484200 1.87117100 -1.17915300<br>H 0.11495900 -2.35261500 -0.61792600<br>H 3.69781100 1.27215600 0.77554800<br>H 1.93563300 -3.74613100 -0.61146100<br>H 1.16936700 -3.60248300 0.99859800<br>H 2.87418200 -4.10448100 0.86090400<br>H 0.46095200 3.69590100 0.02562400<br>H 1.22403800 3.55193500 -1.58654900<br>H 2.04815600 4.44756500 -0.28323800<br>H -4.59559200 -0.22474300 -1.41372200<br>H -4.30071800 -2.06690700 -1.25394500<br>C -2.66191200 -0.78245700 -0.47383800<br>O -2.70178600 0.42431500 0.34799500<br>H -2.32212800 -1.60333000 0.16262200<br>N -3.31631200 0.19118900 1.53294000<br>O -3.38327400 1.17306700 2.17812000	• Electronic Energy<br>= -858.194974<br>• Zero Point Energy Correction<br>= 0.242347<br>• Thermal Correction to Energy<br>= 0.260589<br>• Thermal Correction to Enthalpy<br>= 0.261533<br>• Thermal Correction to Free Energy<br>= 0.193124

Name	Anionic HAs RAF C-2a···NOO• (Model 1) Product
Cartesian Coordinate:	Thermochemical Values:
O 2.63687900 -2.08092100 0.60914500<br>O 1.82781800 2.51215000 0.02826700<br>O 3.35651900 0.48740700 0.92457800<br>C -0.23280600 -0.38561200 -1.00665400<br>C -1.58311800 -0.69364600 -1.59860600<br>C 0.17811100 0.93747900 -0.83719300<br>C 0.57886500 -1.41299400 -0.51998800<br>C 1.77957400 -1.11764700 0.11892400<br>C 1.37222100 1.22655200 -0.18354700<br>C 2.23318200 0.21509600 0.32140100<br>C 2.23588100 -3.43523700 0.47245500<br>C 0.95643500 3.57202100 -0.33414200	• Electronic Energy<br>= -857.723287<br>• Zero Point Energy Correction<br>= 0.230012<br>• Thermal Correction to Energy<br>= 0.247380<br>• Thermal Correction to Enthalpy

C -4.02365700 -1.05158400 -1.08269500<br>H -1.56820200 -1.65124100 -2.12690700<br>H -1.88030800 0.07846600 -2.31438900<br>H -0.45882900 1.73507300 -1.20118800<br>H 0.25780500 -2.44051800 -0.64512600<br>H 2.12521500 -3.71004100 -0.58031400<br>H 1.29596600 -3.62329100 0.99920400<br>H 3.02783300 -4.03241200 0.92071300<br>H 0.00218000 3.49224500 0.19585900<br>H 0.77483500 3.58593600 -1.41239300<br>H 1.45971900 4.49187600 -0.04176500<br>H -4.18921700 -1.04539900 -2.15189700<br>H -4.85866600 -1.20342900 -0.41174900<br>C -2.67443900 -0.79361200 -0.53142900<br>O -2.74183300 0.48856600 0.18103000<br>H -2.40272400 -1.54039000 0.22208800<br>N -1.97903500 0.48930300 1.30521900<br>O -1.92907000 1.56420100 1.78492500	= 0.248325<br>• Thermal Correction to Free Energy<br>= 0.183246

Name	HAs RAF C-3a···NOO• (Model 1) Product
Cartesian Coordinate:	Thermochemical Values:
O 2.32300100 -1.94756900 0.07388500<br>O 1.33192900 2.58450600 -0.15084400<br>O 2.96305500 0.58137100 0.50235600<br>C -0.71074700 -0.29298500 -1.24102200<br>C -2.08811100 -0.61274900 -1.79366100<br>C -0.35738000 1.03834000 -1.01449600<br>C 0.16359900 -1.31821900 -0.89142200<br>C 1.40051800 -1.01923700 -0.31282600<br>C 0.87166300 1.33155100 -0.43379300<br>C 1.75469600 0.30647800 -0.08260900<br>C -3.17490400 -0.34043900 -0.80159600<br>C 1.98328100 -3.31864100 -0.11189100<br>C 0.43798900 3.67241100 -0.37267900<br>H -2.27059200 -0.03497400 -2.70225100<br>H -2.10919700 -1.67368000 -2.07689700<br>H -1.05102800 1.82923400 -1.27292100<br>H -0.12893200 -2.34653400 -1.06085600<br>H -4.05619400 0.21323200 -1.09752400<br>H 3.03131400 1.53924100 0.62087300<br>H 1.82608700 -3.54007300 -1.17012200<br>H 1.08841600 -3.57721700 0.45936800<br>H 2.83228100 -3.88818400 0.25916500<br>H -0.47138400 3.54695100 0.21989000<br>H 0.18543000 3.75401000 -1.43207500<br>H 0.96863400 4.56563300 -0.05170000<br>C -3.07056400 -0.82260800 0.59629900<br>O -2.26740100 0.12075100 1.38783500<br>H -2.57236800 -1.79346000 0.66528000<br>H -4.03895200 -0.86705900 1.09060800<br>N -1.12676200 -0.44291700 1.85559500<br>O -0.42638900 0.36467700 2.35153900	• Electronic Energy<br>= -858.199669<br>• Zero Point Energy Correction<br>= 0.243929<br>• Thermal Correction to Energy<br>= 0.261685<br>• Thermal Correction to Enthalpy<br>= 0.262629<br>• Thermal Correction to Free Energy<br>= 0.196681

Name	Anionic HAs RAF C-3a···NOO• (Model 1) Product
Cartesian Coordinate:	Thermochemical Values:
O -2.67793800 1.57299100 0.08280400<br>O -0.94642400 -2.78943800 -0.07768600<br>O -2.90426600 -1.06773400 0.55054500<br>C 0.58945200 0.38532300 -1.19973500<br>C 1.89421900 0.90680000 -1.78510800<br>C 0.43542400 -0.98032600 -0.94983500<br>C -0.44563000 1.25457900 -0.85898700<br>C -1.61720800 0.76609400 -0.28112200<br>C -0.73016000 -1.45890600 -0.36269400<br>C -1.81504100 -0.60949900 -0.00148200<br>C 3.07162500 0.63361200 -0.90382100<br>C -2.53346000 2.96805900 -0.13009900<br>C 0.12837100 -3.68513000 -0.31443600<br>H 2.06963000 0.46844000 -2.77085000<br>H 1.79279500 1.99067900 -1.92860800<br>H 1.24564800 -1.65588900 -1.19959800<br>H -0.32201400 2.31540900 -1.04151400<br>H 3.89222800 0.02153700 -1.25567100<br>H -2.40011000 3.19465100 -1.19176600<br>H -1.68787300 3.36835200 0.43673700	• Electronic Energy<br>= -857.725784<br>• Zero Point Energy Correction<br>= 0.231720<br>• Thermal Correction to Energy<br>= 0.248765<br>• Thermal Correction to Enthalpy<br>= 0.249709<br>• Thermal Correction to Free Energy<br>= 0.185517

H -3.45536400 3.42601300 0.22383900<br>H 1.01059500 -3.40064900 0.26665600<br>H 0.38697500 -3.72157500 -1.37624400<br>H -0.21785000 -4.66577900 0.00686800<br>C 3.09456500 1.11085600 0.49959700<br>O 2.34573000 0.19167000 1.36713200<br>H 2.62925800 2.09428100 0.60864100<br>H 4.10116000 1.13081100 0.91260600<br>N 1.15875800 0.72610400 1.76162400<br>O 0.48423100 -0.08220900 2.29019100	

Name	HAs RAF C-1···NOO• (Model 2) Product
Cartesian Coordinate:	Thermochemical Values:
O -0.26048500 2.63598400 -0.13188500<br>O -2.50234300 -1.45079000 -0.06959400<br>C 1.11812400 -0.77801800 -0.42544600<br>C 2.41321300 -1.52593600 -0.61315200<br>C -0.09450100 -1.51051000 -0.35493700<br>C 1.09841900 0.62947600 -0.35324500<br>C -0.08191300 1.30121900 -0.19903000<br>C -1.28780200 -0.87905900 -0.18356900<br>C 2.45002200 -2.23304600 -1.94307900<br>C 0.89666000 3.46030400 -0.28537400<br>C -2.55503100 -2.87673600 -0.12831400<br>C 2.49434700 -3.55425500 -2.08374600<br>H 3.24447700 -0.81962100 -0.54551800<br>H 2.53047200 -2.26434800 0.18671800<br>H -0.05557500 -2.59005100 -0.44122800<br>H 2.02937700 1.17616700 -0.43993800<br>H 2.41686200 -1.59352000 -2.82296700<br>H 1.36190300 3.27797400 -1.25623000<br>H 1.60863700 3.26159600 0.51801400<br>H 0.54191100 4.48564500 -0.22588500<br>H -1.96477600 -3.30962600 0.68246200<br>H -2.18489300 -3.23022700 -1.09291100<br>H -3.60260000 -3.14112300 -0.01099800<br>H 2.50840300 -4.01902900 -3.06358300<br>H 2.52184900 -4.21089600 -1.21860000<br>C -1.38215800 0.59685000 -0.04539200<br>O -2.42641500 1.12475000 -0.76965800<br>H -2.32436200 2.08749600 -0.82180800<br>N -1.80496800 0.86160400 1.53653000<br>O -1.19786500 0.23352900 2.37520800<br>O -2.63311600 1.70760900 1.76865100	• Electronic Energy = -858.188080<br>• Zero Point Energy Correction = 0.244062<br>• Thermal Correction to Energy = 0.261703<br>• Thermal Correction to Enthalpy = 0.262647<br>• Thermal Correction to Free Energy = 0.196584

Name	HAs RAF C-3···NOO• (Model 2) Product
Cartesian Coordinate:	Thermochemical Values:
O 0.00173600 2.69246300 -0.11278000<br>O 3.00044000 -0.87840300 -0.04983200<br>O 2.49329200 1.70942200 -0.32461800<br>C -0.66508500 -1.00219400 0.22951000<br>C -1.83499200 -1.94720300 0.32434800<br>C 0.62961000 -1.44411900 0.16221800<br>C 0.18502500 1.34110900 -0.04405200<br>C 1.69824400 -0.54327200 0.02432700<br>C 1.45237600 0.85555100 -0.11268200<br>C -2.80134900 -1.75493800 -0.81544500<br>C -1.20111900 3.14834500 -0.74487500<br>C 3.34638800 -2.26327000 0.03395500<br>C -4.03993400 -1.29495700 -0.66957500<br>H -1.44771700 -2.96900300 0.31691300<br>H -2.36761400 -1.80124900 1.27158900<br>H 0.82202900 -2.51013900 0.17700100<br>H -2.42232900 -1.99297700 -1.80728500<br>H 3.30888900 1.19292200 -0.39462500<br>H -2.07284900 2.97009500 -0.11108300<br>H -1.33177600 2.65861100 -1.71234400<br>H -1.07412200 4.21904700 -0.88734100<br>H 2.90394000 -2.81448200 -0.79757900<br>H 3.01232300 -2.67900800 0.98618600<br>H 4.43056200 -2.30064700 -0.02946400<br>H -4.69830700 -1.16086700 -1.52102600<br>H -4.43360600 -1.04327200 0.31154600	• Electronic Energy = -858.182800<br>• Zero Point Energy Correction = 0.244252<br>• Thermal Correction to Energy = 0.262190<br>• Thermal Correction to Enthalpy = 0.263134<br>• Thermal Correction to Free Energy = 0.196201

C -0.95415500 0.45909300 0.26942400<br>H -1.87563400 0.71087000 -0.26001500<br>N -1.41611600 0.76940200 1.78333900<br>O -0.56172700 0.98728200 2.61238300<br>O -2.60152500 0.68459900 2.02001800	

Name	HAs RAF C-4···NOO$^\bullet$ (Model 2) Product
Cartesian Coordinate:	Thermochemical Values:
O -1.42256700 2.41405800 -0.00482100<br>O 2.84859500 0.61870100 -0.25569900<br>O 1.16790800 2.66204600 -0.33952400<br>C -0.94182400 -2.39395400 0.11020900<br>C 1.09156100 -0.91898800 0.36972200<br>C -1.21693200 0.06792300 0.52165500<br>C -0.70470700 1.27986200 0.18646700<br>C 1.56342700 0.31287100 0.05091500<br>C 0.69666100 1.44308300 -0.02314200<br>C -1.04339400 -2.26868100 -1.38186100<br>C -2.83246800 2.32387300 0.19016700<br>C 3.80310300 -0.44044800 -0.19285100<br>C -2.19122100 -2.30737400 -2.05048900<br>H -0.29323200 -3.23388900 0.37082100<br>H -1.93137500 -2.56550600 0.54281700<br>H 1.73228000 -1.78995200 0.40587100<br>H -2.27994900 -0.08162400 0.65599100<br>H -0.10652300 -2.14028600 -1.91991400<br>H 2.12695900 2.60506900 -0.47415200<br>H -3.05893500 2.01718000 1.21381100<br>H -3.27300400 1.61810000 -0.51780000<br>H -3.22209000 3.32235400 0.00782100<br>H 3.55202000 -1.22149200 -0.91364800<br>H 3.84043100 -0.85772400 0.81564700<br>H 4.76092600 0.00602200 -0.44726400<br>H -2.21781300 -2.22227200 -3.13123200<br>H -3.13951600 -2.43122200 -1.53519800<br>C -0.34301300 -1.11188400 0.72221300<br>N -0.31066500 -1.42407100 2.30919200<br>O -0.74909500 -0.59818200 3.07732700<br>O 0.20367300 -2.46572600 2.65846400	• Electronic Energy = -858.186159<br>• Zero Point Energy Correction = 0.244523<br>• Thermal Correction to Energy = 0.262039<br>• Thermal Correction to Enthalpy = 0.262983<br>• Thermal Correction to Free Energy = 0.197250

Name	HAs RAF C-2a···NOO$^\bullet$ (Model 2) Product
Cartesian Coordinate:	Thermochemical Values:
O -1.74437900 2.39084400 0.17031200<br>O -1.98434000 -2.24729000 0.08835000<br>O -3.08454300 0.14309600 0.51961600<br>C 0.87143400 -0.05520500 -0.73824100<br>C 2.32608000 -0.12970900 -1.12791800<br>C 0.15222900 -1.23538400 -0.55069300<br>C 0.26694900 1.18108100 -0.50848500<br>C -1.05952300 1.24002700 -0.08414700<br>C -1.17343800 -1.17116000 -0.12785900<br>C -1.78159300 0.06131800 0.10770400<br>C -1.03155000 3.61609500 0.02541000<br>C -1.41094700 -3.54219000 -0.07693400<br>C 3.24724000 1.18134400 0.84645700<br>H 2.52599700 -1.04413500 -1.68750900<br>H 2.60786900 0.72219900 -1.74981800<br>H 0.62797800 -2.19109100 -0.73526700<br>H 0.83389000 2.08923800 -0.66576700<br>H -3.42690600 -0.75602000 0.62366200<br>H -0.69854300 3.75194500 -1.00619200<br>H -0.17288700 3.64619400 0.70065400<br>H -1.73469900 4.40239700 0.28986800<br>H -0.57131300 -3.68032700 0.60829500<br>H -1.08027500 -3.68811400 -1.10762700<br>H -2.20115900 -4.25057700 0.15977500<br>H 3.54974300 1.21119400 1.88366500<br>H 3.20405200 2.09975900 0.27794300<br>C 3.27474400 -0.08855200 0.09085500<br>H 4.28284600 -0.35750100 -0.24186400<br>N 2.87886200 -1.25921500 0.97719900<br>O 2.26279700 -1.04695700 1.99843100<br>O 3.14840000 -2.37166900 0.56933200	• Electronic Energy = -858.204753<br>• Zero Point Energy Correction = 0.244754<br>• Thermal Correction to Energy = 0.262122<br>• Thermal Correction to Enthalpy = 0.263066<br>• Thermal Correction to Free Energy = 0.198249

Name	Anionic HAs RAF C-2a···NOO$^{\bullet}$ (Model 2) Product
Cartesian Coordinate:	Thermochemical Values:
O -1.94720300 2.43129300 0.20851500<br>O -2.00206700 -2.26184000 0.08116900<br>O -3.23072200 0.09371100 0.48774500<br>C 0.78132400 0.07469700 -0.62733300<br>C 2.25179600 0.06494100 -0.95243600<br>C 0.08718700 -1.12560800 -0.46770900<br>C 0.11668200 1.28132500 -0.39809200<br>C -1.22328200 1.28199600 -0.02494300<br>C -1.25370400 -1.11607000 -0.09054800<br>C -1.97312400 0.08543400 0.14599100<br>C -1.26496300 3.66783200 0.07042800<br>C -1.33723400 -3.50268100 -0.09772100<br>C 4.59086700 0.10012000 -0.01087800<br>H 2.53575800 -0.86789900 -1.44656400<br>H 2.51341800 0.89155600 -1.61793700<br>H 0.60696600 -2.06085700 -0.64136100<br>H 0.65929900 2.21208100 -0.51591700<br>H -0.89460500 3.80250000 -0.94974900<br>H -0.43037600 3.73853700 0.77381800<br>H -1.99479800 4.44331500 0.29535000<br>H -0.50674400 -3.60954300 0.60622700<br>H -0.96427400 -3.60777100 -1.12040200<br>H -2.07915300 -4.27489400 0.09705700<br>H 5.32409900 0.56366200 0.63431600<br>H 4.91315900 -0.53022400 -0.82884600<br>C 3.14918700 0.23669500 0.28548000<br>H 2.91447800 1.15569700 0.82074000<br>N 2.71100500 -0.85838800 1.27067200<br>O 3.01354600 -2.00628200 1.01492500<br>O 2.03439700 -0.53319200 2.22221100	• Electronic Energy = -857.731732<br>• Zero Point Energy Correction = 0.231806<br>• Thermal Correction to Energy = 0.249035<br>• Thermal Correction to Enthalpy = 0.249980<br>• Thermal Correction to Free Energy = 0.184357

Name	HAs RAF C-3a···NOO$^{\bullet}$ (Model 2) Product
Cartesian Coordinate:	Thermochemical Values:
O -0.27301000 2.80866800 -0.11467600<br>O -2.77815400 -1.04727400 0.54625700<br>O -2.48090900 1.59213600 0.70143500<br>C 0.60560800 -0.69559100 -0.88917000<br>C 1.77998900 -1.52495100 -1.35264700<br>C -0.55463400 -1.32661000 -0.44136200<br>C 0.72571900 0.69050000 -0.80865100<br>C -0.30635600 1.45380900 -0.26171300<br>C -1.59122200 -0.55919900 0.08331900<br>C -1.47075000 0.82840600 0.17950600<br>C 2.66291700 -1.95793300 -0.22179300<br>C 0.94823500 3.46349900 -0.45037200<br>C -2.97154100 -2.45846300 0.49142300<br>H 1.43286500 -2.41167700 -1.89218500<br>H 2.37701300 -0.94470000 -2.06991700<br>H -0.63682400 -2.40489700 -0.49734600<br>H 1.63019800 1.16794100 -1.16466600<br>H 3.43167900 -2.69466200 -0.41383700<br>H -3.20279200 1.00130500 0.95944400<br>H 1.16324600 3.36273600 -1.51672000<br>H 1.77705500 3.05853100 0.13677200<br>H 0.80043900 4.51260800 -0.20475900<br>H -2.22246000 -2.97291600 1.09741000<br>H -2.92499500 -2.81283900 -0.54064500<br>H -3.96319000 -2.63983200 0.89901400<br>C 2.65401100 -1.27172400 1.08340500<br>H 3.31997400 -1.73473800 1.81239200<br>H 1.66208100 -1.14817600 1.52058300<br>N 3.17313000 0.16930700 0.98735700<br>O 3.90482100 0.46440800 0.06673600<br>O 2.81890900 0.94846000 1.84676600	• Electronic Energy = -858.208907<br>• Zero Point Energy Correction = 0.245560<br>• Thermal Correction to Energy = 0.262801<br>• Thermal Correction to Enthalpy = 0.263745<br>• Thermal Correction to Free Energy = 0.198514

Name	Anionic HAs RAF C-3a···NOO$^{\bullet}$ (Model 2) Product
Cartesian Coordinate:	Thermochemical Values:
O -0.25711000 2.83480700 -0.13067400<br>O -2.77659300 -1.06823000 0.56757000<br>O -2.49462100 1.59536500 0.71488500<br>C 0.59890700 -0.68341900 -0.90226400	• Electronic Energy = -857.735459

C 1.77228800 -1.51915000 -1.35611400<br>C -0.56451800 -1.29816300 -0.43524000<br>C 0.71073900 0.70414100 -0.81735900<br>C -0.31774400 1.46285900 -0.26311500<br>C -1.59662200 -0.53167300 0.09849900<br>C -1.52355800 0.88433000 0.21234100<br>C 2.64366800 -1.96741800 -0.22157600<br>C 0.97463300 3.45928000 -0.46105300<br>C -2.92338500 -2.47755600 0.49268300<br>H 1.43006300 -2.40195400 -1.90676500<br>H 2.38357900 -0.94146400 -2.06452700<br>H -0.64664100 -2.37761200 -0.48654400<br>H 1.61498500 1.18196400 -1.17631000<br>H 3.40782000 -2.71200100 -0.40409300<br>H 1.19915400 3.35463200 -1.52623400<br>H 1.79675900 3.04063800 0.12803300<br>H 0.85410000 4.51398600 -0.22035000<br>H -2.15875400 -2.98605100 1.08671700<br>H -2.87126700 -2.82504900 -0.54292700<br>H -3.90720900 -2.70165300 0.90087300<br>C 2.62597500 -1.28190400 1.08384200<br>H 3.28899800 -1.74173300 1.81769100<br>H 1.63036600 -1.15991200 1.51275700<br>N 3.14073200 0.16057200 0.98978300<br>O 3.88482900 0.45564900 0.07876100<br>O 2.77427000 0.94154800 1.84262600	• Zero Point Energy Correction = 0.232975<br>• Thermal Correction to Energy = 0.249671<br>• Thermal Correction to Enthalpy = 0.250616<br>• Thermal Correction to Free Energy = 0.186592

Name	HAs RAF C-2a···NOO$^{\bullet}$ Transition States Structure
Cartesian Coordinate:	Thermochemical Values:
O -1.82553000 2.51400800 -0.01495300<br>O -1.86503300 -2.12588900 0.25066500<br>O -3.06980300 0.24513300 0.51228600<br>C 0.88862800 0.11806400 -0.75737900<br>C 2.34531300 0.07846400 -1.14474600<br>C 0.21606100 -1.07289400 -0.48618700<br>C 0.23465800 1.34057900 -0.61050200<br>C -1.09406200 1.37626000 -0.18692800<br>C -1.10646000 -1.03291500 -0.05339900<br>C -1.76580800 0.18702600 0.09666200<br>C -1.17457600 3.75242200 -0.28421200<br>C -1.21845000 -3.39646300 0.21295900<br>C 3.01910100 0.73934600 1.22021300<br>H 2.56011700 -0.80971700 -1.74404500<br>H 2.59534300 0.94311400 -1.76824100<br>H 0.71607900 -2.02320100 -0.62643200<br>H 0.76925800 2.25753600 -0.82377800<br>H -3.38335300 -0.65843700 0.65899200<br>H -0.85128900 3.80240200 -1.32652800<br>H -0.31734400 3.89227800 0.37863800<br>H -1.91538300 4.52534900 -0.09315400<br>H -0.36051800 -3.40979800 0.89033400<br>H -0.89583700 -3.63478200 -0.80304300<br>H -1.96103700 -4.11943000 0.54186700<br>H 3.79325400 0.85758200 1.96820500<br>H 2.01998000 1.08255100 1.46389900<br>C 3.29074500 0.10453700 0.03479700<br>H 4.33009400 -0.10909100 -0.20059400<br>N 3.02578900 -1.92425900 0.66206400<br>O 2.61012000 -2.16344600 1.76122000<br>O 3.29495500 -2.71469600 -0.19947500	• Electronic Energy = -858.182684<br>• Zero Point Energy Correction = 0.242043<br>• Thermal Correction to Energy = 0.259597<br>• Thermal Correction to Enthalpy = 0.260541<br>• Thermal Correction to Free Energy = 0.195192

Name	HAs RAF C-2a···NOO$^{\bullet}$ Reactant Complex
Cartesian Coordinate:	Thermochemical Values:
O -2.05018200 2.68968600 0.12693400<br>O -1.96118500 -1.95609200 0.09944300<br>O -3.17847000 0.35656900 0.64543400<br>C 0.62609700 0.42087600 -1.04039000<br>C 2.03969400 0.45675100 -1.56150700<br>C 0.00670200 -0.80137600 -0.78967900<br>C -0.03900000 1.61332100 -0.75068600<br>C -1.31633300 1.58540600 -0.19417300<br>C -1.26586700 -0.82569900 -0.22123000<br>C -1.92904800 0.36162400 0.08089000<br>C -1.45542800 3.95931100 -0.12592200<br>C -1.31188400 -3.20627000 -0.11705300	• Electronic Energy = -858.198929<br>• Zero Point Energy Correction = 0.242622<br>• Thermal Correction to Energy = 0.261591<br>• Thermal Correction to Enthalpy

C 2.86068700 1.03535600 0.76987500<br>H 2.28469300 -0.50188300 -2.02984700<br>H 2.13393400 1.21209000 -2.34980900<br>H 0.52219800 -1.72402400 -1.02453500<br>H 0.45262200 2.55730800 -0.94985600<br>H -3.43981400 -0.56304800 0.79441900<br>H -1.24523400 4.08440100 -1.19077600<br>H -0.53538500 4.07886600 0.45116000<br>H -2.18642500 4.69888400 0.19265500<br>H -0.38710500 -3.26593500 0.46270100<br>H -1.09657700 -3.35066100 -1.17820900<br>H -2.00948000 -3.96791300 0.22333700<br>H 3.68657600 1.22444800 1.44706200<br>H 1.85760800 1.10135500 1.18124000<br>C 3.08301600 0.74675500 -0.50952400<br>H 4.10915600 0.70683200 -0.86921500<br>N 1.67906400 -1.61507400 1.83923300<br>O 0.58848400 -1.31289500 2.18041100<br>O 2.10987300 -2.31194300 0.98703800	= 0.262535<br>• Thermal Correction to Free Energy = 0.192431

Name	Anionic HAs RAF C-2a···NOO• Transition States Structure
Cartesian Coordinate:	Thermochemical Values:
O -1.81953300 2.54895100 -0.01441100<br>O -1.85509200 -2.13986500 0.28279300<br>O -3.06986700 0.24186400 0.56959100<br>C 0.87800300 0.12609800 -0.76520300<br>C 2.33604300 0.07992100 -1.14405900<br>C 0.19101000 -1.05436600 -0.47617600<br>C 0.21720000 1.34589800 -0.61465500<br>C -1.10485700 1.38063400 -0.17778100<br>C -1.12587400 -1.01141100 -0.02824600<br>C -1.83664700 0.20627100 0.14636700<br>C -1.15133000 3.76564500 -0.30810200<br>C -1.17761500 -3.38522100 0.21148700<br>C 3.00102700 0.70684900 1.23053400<br>H 2.55484000 -0.80413300 -1.74937500<br>H 2.59841300 0.94613900 -1.76174900<br>H 0.68982300 -2.00641900 -0.61464700<br>H 0.75071600 2.26288700 -0.83573600<br>H -0.83373900 3.79940900 -1.35405100<br>H -0.28255500 3.90586300 0.34145400<br>H -1.87265500 4.55962600 -0.12363900<br>H -0.30494300 -3.39640200 0.87204500<br>H -0.86220800 -3.60638900 -0.81211800<br>H -1.89178600 -4.13822200 0.53958600<br>H 3.76879900 0.81752700 1.98638500<br>H 1.99753800 1.04075000 1.46976400<br>C 3.28166700 0.09404900 0.03628900<br>H 4.32314200 -0.11757800 -0.19343700<br>N 3.01299900 -1.95112700 0.64418800<br>O 2.60613600 -2.20548200 1.74366400<br>O 3.26970700 -2.73169600 -0.23054700	• Electronic Energy = -857.709277<br>• Zero Point Energy Correction = 0.229336<br>• Thermal Correction to Energy = 0.246425<br>• Thermal Correction to Enthalpy = 0.247370<br>• Thermal Correction to Free Energy = 0.182615

Name	Anionic HAs RAF C-2a···NOO• Reactant Complex
Cartesian Coordinate:	Thermochemical Values:
O -2.08318700 2.73860900 0.09152200<br>O -1.99204400 -1.95924500 0.16395000<br>O -3.23929800 0.37801400 0.64823600<br>C 0.59534100 0.42219900 -0.99687400<br>C 2.01663700 0.43682600 -1.49873900<br>C -0.04470800 -0.78484000 -0.71957200<br>C -0.07777100 1.61701700 -0.73096500<br>C -1.35993500 1.59746800 -0.18943800<br>C -1.32155200 -0.79717900 -0.16125300<br>C -2.04097600 0.39037400 0.12957300<br>C -1.46303600 3.98407300 -0.18659200<br>C -1.32042900 -3.18496500 -0.08056300<br>C 2.82881600 1.03686700 0.82909700<br>H 2.26149400 -0.53518900 -1.94183200<br>H 2.13332200 1.16950600 -2.30632400<br>H 0.46793400 -1.71395000 -0.93778500	• Electronic Energy = -857.724699<br>• Zero Point Energy Correction = 0.230250<br>• Thermal Correction to Energy = 0.248627<br>• Thermal Correction to Enthalpy = 0.249571<br>• Thermal Correction to Free Energy = 0.180640

H 0.41743100 2.55778700 -0.94215600<br>H -1.23238900 4.08158700 -1.25122600<br>H -0.54652800 4.10802100 0.39734800<br>H -2.18075900 4.75068200 0.09977700<br>H -0.38516600 -3.24398100 0.48501200<br>H -1.10947800 -3.31478800 -1.14593000<br>H -1.99485300 -3.97171000 0.25286100<br>H 3.64968100 1.23595800 1.50978600<br>H 1.82216200 1.09417200 1.23271700<br>C 3.05779700 0.74134200 -0.44809700<br>H 4.08662500 0.70510500 -0.80223600<br>N 1.96096300 -1.76173500 1.62631700<br>O 0.92079600 -1.49114500 2.11742900<br>O 2.28482800 -2.41717800 0.69664900	

Name	HAs RAF C-3a···NOO• Transition States Structure
Cartesian Coordinate:	Thermochemical Values:
O 0.00062900 2.78813300 -0.33617600<br>O -3.08860600 -0.59613900 0.42615100<br>O -2.31475000 1.94493700 0.62026400<br>C 0.21190700 -0.79224400 -1.21366000<br>C 1.22610500 -1.81033700 -1.70541500<br>C -1.01537400 -1.22665100 -0.71317600<br>C 0.58187500 0.54774200 -1.11829900<br>C -0.26853300 1.46202700 -0.49748600<br>C -1.86485000 -0.30809900 -0.10362200<br>C -1.49306900 1.03283400 0.01558800<br>C 1.87893500 -2.37996100 -0.48117800<br>C 1.26286000 3.25522600 -0.80578600<br>C -3.51970800 -1.95416000 0.37632200<br>H 0.73258300 -2.59797600 -2.27615400<br>H 1.96716800 -1.31691400 -2.33686300<br>H -1.28571700 -2.27300400 -0.78081400<br>H 1.54486900 0.86356200 -1.49924800<br>H 1.40316000 -3.23607600 -0.01193000<br>H -3.10361500 1.48104900 0.93510200<br>H 1.35185600 3.11218600 -1.88523500<br>H 2.08041400 2.74321100 -0.29264900<br>H 1.29127700 4.31733900 -0.57366600<br>H -2.82934200 -2.59644400 0.92767400<br>H -3.60099900 -2.29366600 -0.65860100<br>H -4.49908500 -1.97382700 0.84825500<br>C 2.92570700 -1.76676700 0.15382900<br>H 3.47638900 -0.97558000 -0.34567600<br>H 3.40859100 -2.24975900 0.99602900<br>O 0.94790900 -0.62561000 1.93661800<br>N 1.98991700 -0.33789600 1.41922700<br>O 2.56572500 0.71380800 1.48003900	• Electronic Energy = -858.187622<br>• Zero Point Energy Correction = 0.242492<br>• Thermal Correction to Energy = 0.260002<br>• Thermal Correction to Enthalpy = 0.260946<br>• Thermal Correction to Free Energy = 0.196063

Name	HAs RAF C-3a···NOO• Reactant Complex
Cartesian Coordinate:	Thermochemical Values:
O 0.01596800 2.73314600 -0.30117800<br>O -3.14461700 -0.60868600 0.35606400<br>O -2.34984100 1.92591900 0.56512500<br>C 0.21583100 -0.85680300 -1.15756500<br>C 1.21773600 -1.87327800 -1.66352100<br>C -1.03696900 -1.26897900 -0.70557700<br>C 0.59809900 0.47955000 -1.04814400<br>C -0.26476500 1.40882900 -0.46763000<br>C -1.89695700 -0.33693400 -0.12950100<br>C -1.51548800 0.99918200 -0.00441200<br>C 2.03437100 -2.38929500 -0.50717600<br>C 1.26873400 3.19854800 -0.79610800<br>C -3.59230000 -1.96020200 0.28973400<br>H 0.68978500 -2.70129100 -2.14386800<br>H 1.87854200 -1.41055700 -2.40001400<br>H -1.32497300 -2.30923800 -0.79142600<br>H 1.57831200 0.78215200 -1.39525200<br>H 1.50232800 -3.01099200 0.21125900<br>H -3.15581400 1.47197900 0.84911000<br>H 1.34417200 3.03304800 -1.87346300<br>H 2.09767800 2.70435900 -0.28463600<br>H 1.29272700 4.26554700 -0.58659000	• Electronic Energy = -858.200221<br>• Zero Point Energy Correction = 0.242509<br>• Thermal Correction to Energy = 0.261553<br>• Thermal Correction to Enthalpy = 0.262497<br>• Thermal Correction to Free Energy = 0.193212

H -2.93085500 -2.61310800 0.86376600<br>H -3.64293900 -2.29845800 -0.74756500<br>H -4.58771500 -1.96691400 0.72745300<br>C 3.31090500 -2.08158200 -0.29701200<br>H 3.85930700 -1.45084600 -0.99163300<br>H 3.84622200 -2.44737700 0.57296500<br>O 0.75637600 -0.53709000 1.93253100<br>N 1.88402200 -0.18963400 1.88087800<br>O 2.43975700 0.73088600 1.38818900	

Name	Anionic HAs RAF C-3a···NOO• Transition States Structure
Cartesian Coordinate:	Thermochemical Values:
O -0.21059400 2.79131300 -0.09742700<br>O -2.83090400 -1.05396300 0.55359700<br>O -2.48235600 1.60320200 0.72196200<br>C 0.56843600 -0.74276900 -0.87716300<br>C 1.72735000 -1.58543300 -1.34199000<br>C -0.61375000 -1.33155400 -0.42866000<br>C 0.71400300 0.64198900 -0.78257700<br>C -0.30050000 1.42183200 -0.23658700<br>C -1.63263500 -0.54239000 0.10074100<br>C -1.52562500 0.86924100 0.22473900<br>C 2.72416600 -1.90255000 -0.27003900<br>C 1.02082100 3.39671500 -0.46228700<br>C -3.00978900 -2.45886700 0.46808700<br>H 1.37861500 -2.53335100 -1.77108400<br>H 2.27009700 -1.08224900 -2.15245000<br>H -0.72347400 -2.40782400 -0.49023500<br>H 1.63364100 1.09812900 -1.13035500<br>H 3.63688700 -2.39731000 -0.59046300<br>H 1.22115100 3.27334400 -1.53038400<br>H 1.85171000 2.97948900 0.11536600<br>H 0.91664900 4.45609200 -0.23478300<br>H -2.26310000 -2.98902700 1.06606700<br>H -2.95581700 -2.80130700 -0.56916300<br>H -4.00252800 -2.66259100 0.86528800<br>C 2.59440500 -1.54600800 1.04362300<br>H 3.32619700 -1.88229300 1.76942000<br>H 1.64354700 -1.18982700 1.42536400<br>N 3.26774500 0.48340100 1.07651000<br>O 3.98451000 0.87442100 0.19798800<br>O 2.76498400 1.13497200 1.94881200	• Electronic Energy = -857.713726<br>• Zero Point Energy Correction = 0.229427<br>• Thermal Correction to Energy = 0.246546<br>• Thermal Correction to Enthalpy = 0.247490<br>• Thermal Correction to Free Energy = 0.182250

Name	Anionic HAs RAF C-3a···NOO• Reactant Complex
Cartesian Coordinate:	Thermochemical Values:
O -0.64123700 2.69316900 -0.27115100<br>O -3.15356500 -1.20773900 0.46611700<br>O -2.87579300 1.45841100 0.59310100<br>C 0.22796800 -0.83326100 -0.99835300<br>C 1.38262300 -1.66613500 -1.49428800<br>C -0.93973700 -1.44251100 -0.53256000<br>C 0.33180100 0.55542100 -0.93287600<br>C -0.69969200 1.31851700 -0.38839100<br>C -1.97327200 -0.67295200 -0.00713200<br>C -1.90278200 0.74381000 0.09467000<br>C 2.19146600 -2.34233700 -0.41327200<br>C 0.55784800 3.32741600 -0.68836500<br>C -3.30112800 -2.61696600 0.39522300<br>H 1.02947000 -2.44412600 -2.18185600<br>H 2.06426600 -1.03909100 -2.08035400<br>H -1.02366800 -2.52225200 -0.57687600<br>H 1.22882800 1.03359100 -1.30720600<br>H 3.04836500 -2.90930300 -0.77322000<br>H 0.73141400 3.18193400 -1.75843600<br>H 1.41938900 2.95332700 -0.12598000<br>H 0.42577300 4.38872600 -0.48541000<br>H -2.53709700 -3.12461400 0.99083900<br>H -3.24889100 -2.96768000 -0.63935600<br>H -4.28523200 -2.83951800 0.80373400<br>C 1.95391500 -2.30096600 0.89552400<br>H 2.60128500 -2.81911700 1.59497200<br>H 1.11021900 -1.75576200 1.30805200<br>N 2.89589900 0.57887200 1.07258700	• Electronic Energy = -857.724674<br>• Zero Point Energy Correction = 0.230066<br>• Thermal Correction to Energy = 0.248466<br>• Thermal Correction to Enthalpy = 0.249410<br>• Thermal Correction to Free Energy = 0.180582

O 3.35512700 0.80504300 0.00640300<br>O 1.95361200 0.97942800 1.66251900	

Name	HPns (4-propenylsyringol)
Cartesian Coordinate:	Thermochemical Values:
O 2.44300500 -1.83699700 -0.00017200<br>O 1.10112600 2.60810300 0.00005000<br>O 3.00603800 0.74589300 -0.00029000<br>C -0.98840700 -0.44098600 0.00030800<br>C 1.37486900 -0.98840700 -0.00002500<br>C 0.66224900 1.31544300 0.00008300<br>C -0.66958100 0.92274200 0.00030900<br>C 0.03948200 -1.38912900 0.00018100<br>C 1.69039700 0.36813100 -0.00009100<br>C -2.37967400 -0.93114000 0.00043900<br>C -3.49248700 -0.19234900 -0.00046200<br>C 2.16468400 -3.23432400 -0.00002100<br>C 0.11527900 3.63699900 0.00005900<br>C -4.87467300 -0.76612300 -0.00023600<br>H -1.45105100 1.67019700 0.00054500<br>H -0.21600800 -2.44073100 0.00022300<br>H -2.47981900 -2.01543500 0.00130200<br>H -3.42570100 0.89355300 -0.00148400<br>H 3.04674100 1.71278600 -0.00019800<br>H 1.60534000 -3.51881700 -0.89437300<br>H 1.60562300 -3.51867800 0.89455200<br>H 3.13279000 -3.72983600 -0.00013700<br>H -0.50793100 3.57336100 0.89488400<br>H -0.50810700 3.57319500 -0.89463400<br>H 0.66412100 4.57569400 -0.00008100<br>H -5.43460200 -0.42874800 0.87704700<br>H -5.43423300 -0.43032900 -0.87836000<br>H -4.85134200 -1.85734100 0.00075000	• Electronic Energy<br>= -653.149950<br>• Zero Point Energy Correction<br>= 0.231740<br>• Thermal Correction to Energy<br>= 0.246588<br>• Thermal Correction to Enthalpy<br>= 0.247532<br>• Thermal Correction to Free Energy<br>= 0.189452

Name	Anionic HPns
Cartesian Coordinate:	Thermochemical Values:
O -2.52812400 -1.76955300 -0.00001900<br>O -1.03444900 2.67941000 -0.00000800<br>O -3.01215100 0.87395800 0.00003100<br>C 0.94291800 -0.45414000 -0.00006700<br>C -1.43875900 -0.92229900 -0.00002800<br>C -0.67120200 1.35019700 -0.00001500<br>C 0.64645600 0.91697200 -0.00005700<br>C -0.12079200 -1.36527400 -0.00006300<br>C -1.78130400 0.45569000 0.00000200<br>C 2.31793500 -0.97618700 -0.00007800<br>C 3.45954300 -0.27788300 0.00016900<br>C -2.26455500 -3.16377000 -0.00011000<br>C 0.00916900 3.64059000 0.00009200<br>C 4.82218200 -0.90167800 0.00011600<br>H 1.45215200 1.63944500 -0.00010100<br>H 0.10343200 -2.42527000 -0.00008300<br>H 2.38847300 -2.06397900 -0.00030800<br>H 3.43112900 0.80999700 0.00043300<br>H -1.70691600 -3.46032900 0.89284900<br>H -1.70695000 -3.46021800 -0.89312700<br>H -3.23554400 -3.65564100 -0.00012200<br>H 0.63347500 3.54557100 -0.89292200<br>H 0.63343200 3.54542500 0.89312000<br>H -0.47660500 4.61458000 0.00015700<br>H 5.39734100 -0.59067800 -0.87749100<br>H 5.39724900 -0.59102300 0.87790400<br>H 4.75757900 -1.99170600 -0.00010300	• Electronic Energy<br>= -652.677373<br>• Zero Point Energy Correction<br>= 0.219459<br>• Thermal Correction to Energy<br>= 0.233619<br>• Thermal Correction to Enthalpy<br>= 0.234563<br>• Thermal Correction to Free Energy<br>= 0.178254

Name	HPns 1-OH radical
Cartesian Coordinate:	Thermochemical Values:

O -2.58209000 -1.66655900 -0.00003100<br>O -0.90789400 2.70243800 0.00003900<br>O -2.90869900 0.96643400 0.00007300<br>C 0.91201800 -0.49912300 -0.00008400<br>C -1.47164900 -0.91352600 -0.00004100<br>C -0.58024900 1.39959600 0.00000400<br>C 0.69624100 0.90147200 -0.00005300<br>C -0.18293500 -1.39162600 -0.00009800<br>C -1.74571800 0.51837800 0.00001900<br>C 2.24701400 -1.07382600 -0.00010500<br>C 3.40442600 -0.39010000 0.00014200<br>C -2.41947300 -3.08550000 -0.00011500<br>C 0.15753500 3.65220600 0.00008800<br>C 4.74852900 -1.03466300 0.00012000<br>H 1.54342000 1.57255200 -0.00009000<br>H 0.01795300 -2.45514200 -0.00014000<br>H 2.28691700 -2.16076100 -0.00030900<br>H 3.39434000 0.69687200 0.00038500<br>H -1.88327700 -3.40706800 0.89519700<br>H -1.88332000 -3.40696600 -0.89548900<br>H -3.42483200 -3.49831100 -0.00011500<br>H 0.77231500 3.53539500 -0.89503700<br>H 0.77234500 3.53526500 0.89517500<br>H -0.31704600 4.62998400 0.00016500<br>H 5.32177800 -0.71849500 -0.87673400<br>H 5.32164800 -0.71883500 0.87718100<br>H 4.67278900 -2.12272900 -0.00009700	• Electronic Energy<br>= -652.520688<br>• Zero Point Energy Correction<br>= 0.220319<br>• Thermal Correction to Energy<br>= 0.234377<br>• Thermal Correction to Enthalpy<br>= 0.235321<br>• Thermal Correction to Free Energy<br>= 0.178986

Name	HPns 3a-CH radical
Cartesian Coordinate:	Thermochemical Values:
O 2.51667600 -1.68205200 -0.00011700<br>O 0.85650000 2.65374300 0.00003700<br>O 2.88372400 0.92970900 -0.00026300<br>C -1.01704000 -0.54277600 0.00021800<br>C 1.38616100 -0.92009900 -0.00000900<br>C 0.50720900 1.33488200 0.00006000<br>C -0.78996200 0.85088500 0.00023400<br>C 0.09109600 -1.41771500 0.00015000<br>C 1.60235100 0.46135200 -0.00009200<br>C -2.33387500 -1.11150900 0.00025300<br>C -3.55076300 -0.39943800 -0.00029200<br>C 2.34600100 -3.09661600 0.00001200<br>C -0.19727000 3.61333800 0.00009000<br>C -4.77219500 -1.00322100 -0.00020900<br>H -1.62156700 1.54086700 0.00050800<br>H -0.08852700 -2.48489900 0.00017500<br>H -2.39145400 -2.19682600 0.00065400<br>H -3.52017100 0.68685400 -0.00094400<br>H 2.85703200 1.89727100 -0.00025000<br>H 1.80987900 -3.42242400 -0.89448800<br>H 1.81005600 -3.42228100 0.89467000<br>H 3.34907400 -3.51678400 -0.00005300<br>H -0.81443300 3.50638800 0.89498700<br>H -0.81464600 3.50625200 -0.89464300<br>H 0.28629400 4.58720100 -0.00004000<br>H -5.68601300 -0.42247300 -0.00071300<br>H -4.86101000 -2.08484200 0.00038800	• Electronic Energy<br>= -652.509606<br>• Zero Point Energy Correction<br>= 0.218860<br>• Thermal Correction to Energy<br>= 0.233160<br>• Thermal Correction to Enthalpy<br>= 0.234104<br>• Thermal Correction to Free Energy<br>= 0.177225

Name	HPns 3a-CH radical anion
Cartesian Coordinate:	Thermochemical Values:
O 2.63188400 -1.55452400 0.00007800<br>O 0.72163300 2.73325100 -0.00007100<br>O 2.84945700 1.11098100 0.00001000<br>C -0.96166700 -0.58180100 0.00001100<br>C 1.46380000 -0.82924500 0.00004700<br>C 0.47777100 1.38061200 -0.00003300<br>C -0.78486000 0.82929300 -0.00003200<br>C 0.20675200 -1.39234500 0.00005100<br>C 1.67394800 0.58794700 0.00000800<br>C -2.23355400 -1.20770500 0.00001600<br>C -3.49416900 -0.55987900 -0.00005000<br>C 2.51601500 -2.96956000 0.00011600<br>C -0.40592100 3.59634900 -0.00011400<br>C -4.69205600 -1.20491100 -0.00003000<br>H -1.65340100 1.47344400 -0.00005800	• Electronic Energy<br>= -652.041649<br>• Zero Point Energy Correction<br>= 0.206686<br>• Thermal Correction to Energy<br>= 0.220355<br>• Thermal Correction to Enthalpy<br>= 0.221299<br>• Thermal Correction to Free Energy

H 0.08181600 -2.46836700 0.00008200<br>H -2.23953900 -2.29564500 0.00007300<br>H -3.50928300 0.52775200 -0.00012900<br>H 1.99202300 -3.32074700 -0.89310300<br>H 1.99201000 -3.32069800 0.89334600<br>H 3.53328300 -3.35611100 0.00013300<br>H -1.01770800 3.44303500 0.89314000<br>H -1.01768400 3.44297800 -0.89337400<br>H -0.01026300 4.61008000 -0.00014100<br>H -5.62596500 -0.65649600 -0.00009100<br>H -4.74541900 -2.28942400 0.00004900	= 0.165761

Name	HPns 2’-CH radical
Cartesian Coordinate:	Thermochemical Values:
O -2.40115800 -1.83745000 0.05064200<br>O -1.11460200 2.63264700 -0.18217100<br>O -3.01061400 0.73019500 -0.03797500<br>C 1.00814000 -0.38655200 -0.04580500<br>C -1.34894500 -0.97338100 0.00281000<br>C -0.67297700 1.33011000 -0.09957700<br>C 0.66569100 0.96706800 -0.10745400<br>C -0.00849100 -1.34902700 0.00416500<br>C -1.69041000 0.38005400 -0.04450100<br>C 2.40696500 -0.85407800 -0.04098900<br>C 3.50316100 -0.09294100 0.00976100<br>C -2.09946200 -3.22986900 0.08721500<br>C -0.27760700 3.63291100 0.23458400<br>C 4.89738400 -0.63572500 0.01243000<br>H 1.42275500 1.73730200 -0.18165500<br>H 0.26379500 -2.39574900 0.04460500<br>H 2.52741800 -1.93556900 -0.07534600<br>H 3.41022900 0.99013600 0.05561800<br>H -3.07871600 1.69553700 -0.03137400<br>H -1.52302900 -3.47849800 0.98130100<br>H -1.54797900 -3.52999800 -0.80690700<br>H -3.05908300 -3.74062300 0.11553600<br>H -0.70409300 4.61413400 0.09039500<br>H 0.40227700 3.40815000 1.04723800<br>H 5.46470600 -0.25024500 -0.83992100<br>H 5.43311100 -0.32293900 0.91358800<br>H 4.89890700 -1.72619900 -0.03288800	• Electronic Energy<br>= -652.509606<br>• Zero Point Energy Correction<br>= 0.218860<br>• Thermal Correction to Energy<br>= 0.233160<br>• Thermal Correction to Enthalpy<br>= 0.234104<br>• Thermal Correction to Free Energy<br>= 0.177225

Name	HPns 2’-CH radical anion
Cartesian Coordinate:	Thermochemical Values:
O -2.40115800 -1.83745000 0.05064200<br>O -1.11460200 2.63264700 -0.18217100<br>O -3.01061400 0.73019500 -0.03797500<br>C 1.00814000 -0.38655200 -0.04580500<br>C -1.34894500 -0.97338100 0.00281000<br>C -0.67297700 1.33011000 -0.09957700<br>C 0.66569100 0.96706800 -0.10745400<br>C -0.00849100 -1.34902700 0.00416500<br>C -1.69041000 0.38005400 -0.04450100<br>C 2.40696500 -0.85407800 -0.04098900<br>C 3.50316100 -0.09294100 0.00976100<br>C -2.09946200 -3.22986900 0.08721500<br>C -0.27760700 3.63291100 0.23458400<br>C 4.89738400 -0.63572500 0.01243000<br>H 1.42275500 1.73730200 -0.18165500<br>H 0.26379500 -2.39574900 0.04460500<br>H 2.52741800 -1.93556900 -0.07534600<br>H 3.41022900 0.99013600 0.05561800<br>H -3.07871600 1.69553700 -0.03137400<br>H -1.52302900 -3.47849800 0.98130100<br>H -1.54797900 -3.52999800 -0.80690700<br>H -3.05908300 -3.74062300 0.11553600<br>H -0.70409300 4.61413400 0.09039500<br>H 0.40227700 3.40815000 1.04723800<br>H 5.46470600 -0.25024500 -0.83992100<br>H 5.43311100 -0.32293900 0.91358800<br>H 4.89890700 -1.72619900 -0.03288800	• Electronic Energy<br>= -652.509606<br>• Zero Point Energy Correction<br>= 0.218860<br>• Thermal Correction to Energy<br>= 0.233160<br>• Thermal Correction to Enthalpy<br>= 0.234104<br>• Thermal Correction to Free Energy<br>= 0.177225

Name	HPns HAT 3a-CH···NOO• Transition State
Cartesian Coordinate:	Thermochemical Values:
O 3.23078800 -1.86112300 0.47615000<br>O 2.01429800 2.59070800 0.03970200<br>O 3.74828000 0.70376200 0.65914300<br>C 0.00415000 -0.43368900 -0.65937200<br>C 2.22707400 -1.02438700 0.12152700<br>C 1.56792400 1.32025200 -0.11409100<br>C 0.31959800 0.94548100 -0.55133800<br>C 0.97740700 -1.40655400 -0.31789000<br>C 2.53201900 0.34638800 0.22821300<br>C -1.26384300 -0.89979900 -1.09759100<br>C -2.33795200 -0.10992300 -1.48961200<br>C 2.97184300 -3.26103700 0.37590900<br>C 1.11432300 3.64838000 -0.29237000<br>H -0.41154300 1.69869900 -0.80747700<br>H 0.71704100 -2.45294400 -0.40751000<br>H -1.40196000 -1.97775500 -1.12669200<br>H -2.23335600 0.97128700 -1.47545800<br>H 3.80567700 1.67223400 0.68769100<br>H 2.74114900 -3.53480600 -0.65605300<br>H 2.14727900 -3.54681300 1.03270100<br>H 3.88661700 -3.75461000 0.69399800<br>H 0.22681600 3.60557600 0.34243900<br>H 0.82917300 3.58791600 -1.34475200<br>H 1.65895700 4.57048400 -0.10697800<br>H -4.16717500 -0.60628200 -0.66989400<br>O -4.72546700 -0.57513300 0.58506900<br>C -3.60328000 -0.63972900 -1.82316600<br>H -4.26471400 -0.00072900 -2.40369400<br>H -3.64314600 -1.70086600 -2.06459000<br>N -3.92070100 0.16532500 1.23750300<br>O -4.20558600 0.31666400 2.41023800	• Electronic Energy<br>= -858.180841<br>• Zero Point Energy Correction<br>= 0.236719<br>• Thermal Correction to Energy<br>= 0.254866<br>• Thermal Correction to Enthalpy<br>= 0.255810<br>• Thermal Correction to Free Energy<br>= 0.187349

Name	HPns HAT 3a-CH··· NOO• Reactant Complex
Cartesian Coordinate:	Thermochemical Values:
O 3.28886000 -1.49045400 0.45188100<br>O 1.36465300 2.65035100 -0.31087500<br>O 3.40040900 1.11155900 0.31794400<br>C -0.16426100 -0.69266300 -0.57050600<br>C 2.15740000 -0.87798500 0.09768300<br>C 1.11280700 1.33518600 -0.32557800<br>C -0.06983600 0.72637100 -0.63310300<br>C 0.95915100 -1.47657600 -0.19907300<br>C 2.25350500 0.54820500 0.03570900<br>C -1.38488100 -1.38766100 -0.85802200<br>C -2.54713100 -0.80666400 -1.24667100<br>C 3.26279800 -2.92012200 0.53232300<br>C 0.27578500 3.53349000 -0.60465900<br>H -0.93005700 1.31990300 -0.90504400<br>H 0.85042200 -2.55195900 -0.15798700<br>H -1.36036900 -2.46728600 -0.73921000<br>H -2.60153800 0.26944200 -1.38244900<br>H 3.33540000 2.08066200 0.23193400<br>H 3.01774000 -3.34632300 -0.44229400<br>H 2.53637900 -3.24086400 1.28107500<br>H 4.26505500 -3.21423000 0.83036300<br>H -0.53258600 3.37814700 0.11276300<br>H -0.07913200 3.36246700 -1.62243900<br>H 0.67793900 4.53793800 -0.50940400<br>H -4.59478500 -1.17784100 -0.86022300<br>O -3.47651000 -0.62847100 1.85840600<br>C -3.79548600 -1.56265100 -1.50258400<br>H -4.12501300 -1.39736800 -2.53377500<br>H -3.67022700 -2.63091800 -1.32709500<br>N -3.61423300 0.55220400 1.48934000<br>O -2.55850000 1.19441700 1.33830500	• Electronic Energy<br>= -858.204801<br>• Zero Point Energy Correction<br>= 0.242067<br>• Thermal Correction to Energy<br>= 0.261330<br>• Thermal Correction to Enthalpy<br>= 0.262274<br>• Thermal Correction to Free Energy<br>= 0.190803

Name	HPns HAT 3a-CH··· NOO• Product Complex
Cartesian Coordinate:	Thermochemical Values:

O 2.46152200 -1.73328200 -0.64820000<br>O 1.25154100 2.70797900 -0.04493400<br>O 3.08546000 0.78517900 -0.17276000<br>C -0.92106900 -0.21142900 -0.74194900<br>C 1.42019600 -0.85555600 -0.61961500<br>C 0.77523700 1.45571900 -0.30452000<br>C -0.55823300 1.12869800 -0.48597700<br>C 0.08896400 -1.19383700 -0.81380500<br>C 1.77179800 0.47478800 -0.36605300<br>C -2.28548200 -0.63134700 -0.88647200<br>C -3.42849300 0.18687300 -0.78211600<br>C 2.14484900 -3.10732700 -0.85369200<br>C 0.29921000 3.76331500 0.05788000<br>H -1.31426000 1.89828600 -0.42019500<br>H -0.19607400 -2.22135100 -0.99792800<br>H -2.44686500 -1.68895700 -1.07926300<br>H -3.29669000 1.24712800 -0.58385600<br>H 3.15344300 1.73514900 -0.00021000<br>H 1.66891000 -3.25484100 -1.82590500<br>H 1.49123100 -3.47549500 -0.05912100<br>H 3.09335800 -3.63846700 -0.82532500<br>H -0.40159100 3.57009300 0.87323200<br>H -0.24417700 3.88381200 -0.88193700<br>H 0.87226300 4.66271000 0.27013700<br>H -2.28792900 -1.28047200 2.10291100<br>O -1.35006800 -1.52692700 2.17383000<br>C -4.70020600 -0.28521300 -0.91494200<br>H -5.55605800 0.37202500 -0.82629800<br>H -4.88792200 -1.33542400 -1.11402000<br>N -0.67660200 -0.34879100 2.32132000<br>O 0.48253800 -0.53054500 2.36532600	• Electronic Energy = -858.210064<br>• Zero Point Energy Correction = 0.241061<br>• Thermal Correction to Energy = 0.260223<br>• Thermal Correction to Enthalpy = 0.261167<br>• Thermal Correction to Free Energy = 0.191312

Name	Anionic HPns HAT 3a-CH··· NOO• Transition State
Cartesian Coordinate:	Thermochemical Values:
O 3.31659400 -1.80975800 0.50691000<br>O 1.98082000 2.66191400 0.05663000<br>O 3.77473500 0.80826600 0.69243000<br>C 0.07170600 -0.43335700 -0.65698000<br>C 2.30472100 -0.97369700 0.14523700<br>C 1.60236100 1.36295100 -0.08984800<br>C 0.37398200 0.95472700 -0.53491700<br>C 1.07607100 -1.38029700 -0.30429200<br>C 2.64511400 0.42569500 0.27945900<br>C -1.17912100 -0.91713400 -1.11444200<br>C -2.26370600 -0.14874700 -1.51213300<br>C 3.07388200 -3.20734900 0.39700800<br>C 1.02178200 3.66074600 -0.27044700<br>H -0.37621800 1.68804600 -0.79557600<br>H 0.83884400 -2.43249700 -0.39969700<br>H -1.29306100 -1.99855900 -1.15119900<br>H -2.16152000 0.93431800 -1.49247200<br>H 2.85533200 -3.48223700 -0.63801300<br>H 2.24465700 -3.50912400 1.04182400<br>H 3.98889400 -3.69754700 0.72168800<br>H 0.13578500 3.56987500 0.36305500<br>H 0.73287100 3.59171100 -1.32223300<br>H 1.50864800 4.61561200 -0.08558200<br>H -4.12804300 -0.66928300 -0.56810600<br>O -4.62304100 -0.72272100 0.54014300<br>C -3.53099300 -0.65801500 -1.85121700<br>H -4.20512800 -0.00619100 -2.40057200<br>H -3.60074700 -1.71775100 -2.09355100<br>N -4.02857300 0.19889700 1.22231500<br>O -4.38849500 0.26332600 2.37134400	• Electronic Energy = -857.729951<br>• Zero Point Energy Correction = 0.224409<br>• Thermal Correction to Energy = 0.242177<br>• Thermal Correction to Enthalpy = 0.243121<br>• Thermal Correction to Free Energy = 0.175222

Name	Anionic HPns HAT 3a-CH··· NOO• Reactant Complex
Cartesian Coordinate:	Thermochemical Values:
O 3.31957200 -1.48922600 0.42468500<br>O 1.38007000 2.72037500 -0.24245500<br>O 3.44449200 1.16161700 0.35308100<br>C -0.11883200 -0.62649500 -0.57823700<br>C 2.19831300 -0.83325000 0.08543700<br>C 1.17335700 1.39230100 -0.28069900	• Electronic Energy = -857.773357<br>• Zero Point Energy Correction

C -0.01263100 0.78638000 -0.60053900<br>C 0.99573500 -1.42068700 -0.22474500<br>C 2.35903300 0.61611500 0.07273000<br>C -1.36207400 -1.30851800 -0.88823800<br>C -2.49329300 -0.72989200 -1.32794100<br>C 3.26485000 -2.91549400 0.46065400<br>C 0.26310400 3.56559900 -0.51807300<br>H -0.87792700 1.38233300 -0.85307100<br>H 0.87990300 -2.49667300 -0.20962400<br>H -1.35726200 -2.38494900 -0.73276200<br>H -2.52413500 0.34154500 -1.50732100<br>H 3.01175200 -3.31157500 -0.52505800<br>H 2.53336200 -3.25040900 1.19892300<br>H 4.26047300 -3.24265500 0.74879400<br>H -0.54487900 3.37218300 0.19167300<br>H -0.09066700 3.41042600 -1.53968000<br>H 0.62565900 4.58366900 -0.40185900<br>H -4.55562700 -1.07407500 -0.93225800<br>O -3.28453500 -0.79552400 1.91865100<br>C -3.76114900 -1.47308700 -1.57210700<br>H -4.09578100 -1.33879300 -2.60516400<br>H -3.64786300 -2.53889900 -1.36899800<br>N -3.65472700 0.32653300 1.52666000<br>O -2.74364800 1.14579600 1.30608000	= 0.229475<br>• Thermal Correction to Energy<br>= 0.248249<br>• Thermal Correction to Enthalpy<br>= 0.249193<br>• Thermal Correction to Free Energy<br>= 0.179869

Name	Anionic HPns HAT 3a-CH··· NOO• Product Complex
Cartesian Coordinate:	Thermochemical Values:
O 2.98292500 -1.86608500 0.22802700<br>O 1.73201900 2.65308700 0.04046400<br>O 3.52210900 0.73373700 0.54291800<br>C -0.26316600 -0.36673500 -0.80399300<br>C 1.97998200 -0.97683500 -0.06908300<br>C 1.33220400 1.35676400 -0.16733500<br>C 0.07938300 1.00172800 -0.61271400<br>C 0.73075300 -1.34332400 -0.51368200<br>C 2.35672700 0.39342200 0.12859700<br>C -1.53565300 -0.79688200 -1.24675500<br>C -2.64200500 0.02775400 -1.57029900<br>C 2.69645500 -3.24623700 0.05690500<br>C 0.77612900 3.67425700 -0.20503900<br>H -0.65508100 1.76822000 -0.81776100<br>H 0.47693000 -2.38697600 -0.65332100<br>H -1.68861200 -1.87193600 -1.31759600<br>H -2.50746900 1.10646500 -1.53266800<br>H 2.44856400 -3.46877100 -0.98454000<br>H 1.87251700 -3.55919100 0.70399900<br>H 3.60240600 -3.77990000 0.33731500<br>H -0.09685500 3.55781400 0.44311800<br>H 0.45837300 3.67128300 -1.25118600<br>H 1.27453300 4.61482700 0.02083400<br>H -3.87379800 -0.62320800 0.33402500<br>O -3.68227000 -0.80516400 1.27949600<br>C -3.88231200 -0.43840700 -1.89246400<br>H -4.69200200 0.23968400 -2.13239800<br>H -4.08111900 -1.50472800 -1.95553200<br>N -2.77305100 0.13353000 1.63747700<br>O -2.41703600 -0.01698900 2.74891400	• Electronic Energy<br>= -857.741195<br>• Zero Point Energy Correction<br>= 0.228719<br>• Thermal Correction to Energy<br>= 0.247309<br>• Thermal Correction to Enthalpy<br>= 0.248253<br>• Thermal Correction to Free Energy<br>= 0.178177

Name	HPns RAF C-1···NO• Product
Cartesian Coordinate:	Thermochemical Values:
O -1.82579600 -2.23230500 0.16786800<br>O -1.02955200 2.36468800 0.32795400<br>O -2.46531500 0.19398900 1.10175000<br>C 1.40336800 -0.41009500 0.04058400<br>C -0.86057500 -1.28892400 0.15404000<br>C -0.43166900 1.16048900 0.20356100<br>C 0.90289100 0.92247700 0.11522500<br>C 0.47687000 -1.49892600 0.08228400<br>C 2.81008000 -0.70829700 -0.05352400<br>C 3.82436500 0.17941700 -0.13798900<br>C -1.40319200 -3.59470900 0.14081600	• Electronic Energy<br>= -782.992654<br>• Zero Point Energy Correction<br>= 0.238750<br>• Thermal Correction to Energy<br>= 0.255702<br>• Thermal Correction to Enthalpy

C -0.19018800 3.51924600 0.32334600<br>C 5.26244800 -0.21249000 -0.22963900<br>H 1.59488100 1.75389100 0.14554500<br>H 0.87045700 -2.50738500 0.05139500<br>H 3.06058400 -1.76762700 -0.06081200<br>H 3.61423000 1.24584800 -0.14320900<br>H -2.83044600 1.08952300 1.05009000<br>H -0.78509000 -3.81727600 1.01330900<br>H -0.84584000 -3.80196500 -0.77544400<br>H -2.31201300 -4.19035500 0.16686900<br>H 0.36948100 3.57615400 -0.61282200<br>H 0.49758400 3.49191200 1.17123700<br>H -0.85707600 4.37285500 0.41243200<br>H 5.71449400 0.18099100 -1.14555200<br>H 5.83284900 0.20860000 0.60433100<br>H 5.38138500 -1.29724200 -0.22024000<br>C -1.46177900 0.07401800 0.13737300<br>N -2.08368400 0.27536600 -1.28791400<br>O -3.01876300 1.00878800 -1.25984600	= 0.256647<br>• Thermal Correction to Free Energy<br>= 0.193988

Name	HPns RAF C-4···NO• Product
Cartesian Coordinate:	Thermochemical Values:
O 2.51802100 -1.84841500 -0.35399300<br>O 1.30366200 2.62615000 -0.23153500<br>O 3.11940300 0.70495100 -0.42805000<br>C 1.47886100 -1.00720600 -0.11914100<br>C 0.82319000 1.36758200 -0.06374900<br>C -0.47888200 1.03581100 0.16396700<br>C 0.19702700 -1.38326000 0.13132100<br>C 1.83187000 0.37369800 -0.18541500<br>C -2.18465200 -0.80443600 -0.16257300<br>C -2.90332500 -0.11489300 -1.04429500<br>C 2.24064300 -3.24527300 -0.29682200<br>C 0.37390700 3.70235900 -0.11817600<br>C -4.19547900 -0.59102100 -1.63060100<br>H -1.24679500 1.79115400 0.26357300<br>H -0.08856600 -2.42588600 0.17415900<br>H -2.53222900 -1.78154800 0.17051200<br>H -2.54441800 0.85439800 -1.38372600<br>H 3.19036200 1.67033800 -0.47819100<br>H 1.50732100 -3.52308200 -1.05721900<br>H 1.87372400 -3.52363100 0.69386300<br>H 3.18553800 -3.74516900 -0.49548900<br>H -0.07854800 3.71088300 0.87572200<br>H -0.40118900 3.61828200 -0.88291400<br>H 0.94989300 4.61129400 -0.27242500<br>H -4.99846800 0.12010000 -1.41749300<br>H -4.11942000 -0.66474700 -2.71914900<br>H -4.47817900 -1.56716900 -1.23301800<br>C -0.86670800 -0.37842900 0.41917600<br>N -0.98176700 -0.34047700 2.00502100<br>O -2.09675500 -0.29412000 2.40914000	• Electronic Energy<br>= -782.976417<br>• Zero Point Energy Correction<br>= 0.237878<br>• Thermal Correction to Energy<br>= 0.255024<br>• Thermal Correction to Enthalpy<br>= 0.255968<br>• Thermal Correction to Free Energy<br>= 0.192149

Name	HPns RAF C-1a···NO• Product
Cartesian Coordinate:	Thermochemical Values:
O 1.80317800 2.55435800 0.03995200<br>O 2.64476600 -2.01050600 -0.07681900<br>O 3.48076700 0.51922900 -0.00738000<br>C -0.59689500 -0.24854100 -0.05558200<br>C 1.23837600 1.31522500 0.00063800<br>C 1.66588500 -1.06094800 -0.05745400<br>C 0.29691000 -1.31710300 -0.06805700<br>C -0.13219800 1.06682300 -0.01892400<br>C 2.14027500 0.24974300 -0.02185100<br>C -2.89228200 0.22135100 -1.00556700<br>C 0.91962900 3.67226400 0.05808700<br>C 2.23216500 -3.37506500 -0.09413400<br>C -4.25776800 -0.24490800 -1.33386000<br>H -0.07594700 -2.33282800 -0.09002500<br>H -0.83298700 1.89127500 -0.00030000<br>H -2.55852000 1.20471800 -1.31404300<br>H 3.96047500 -0.32116200 -0.01772700<br>H 0.28362400 3.65012900 0.94611000<br>H 0.30165200 3.69276000 -0.84263500<br>H 1.55664200 4.55324500 0.08546000	• Electronic Energy<br>= -782.999202<br>• Zero Point Energy Correction<br>= 0.238539<br>• Thermal Correction to Energy<br>= 0.255526<br>• Thermal Correction to Enthalpy<br>= 0.256470<br>• Thermal Correction to Free Energy<br>= 0.193159

H 1.64732500 -3.58840900 -0.99155600<br>H 1.64807500 -3.61084100 0.79818200<br>H 3.14660300 -3.96322900 -0.10203600<br>H -4.89534700 -0.23922700 -0.43499200<br>H -4.72969700 0.38846300 -2.08398900<br>H -4.24576500 -1.28064000 -1.68855800<br>C -2.06876900 -0.53332900 -0.05183500<br>H -2.28050000 -1.60377500 -0.08523500<br>N -2.67550100 -0.01351800 1.27168100<br>O -3.61234800 -0.66544900 1.64559800	

Name	Anionic HPns RAF C-1a···$NO^{\bullet}$ Product
Cartesian Coordinate:	Thermochemical Values:
O 1.87339100 2.55348200 0.07139400<br>O 2.63322200 -2.07899900 -0.09032100<br>O 3.53797800 0.44843200 0.00440400<br>C -0.56699000 -0.22129300 -0.08678400<br>C 1.30609600 1.29939300 0.00817400<br>C 1.69581200 -1.06819400 -0.07136300<br>C 0.32381300 -1.29602400 -0.09889600<br>C -0.06622000 1.08228400 -0.02830100<br>C 2.25477200 0.23797200 -0.01940000<br>C -2.88302700 0.34896700 -0.95192100<br>C 0.99222700 3.66538600 0.10624400<br>C 2.14940900 -3.41202000 -0.14222700<br>C -4.25328300 -0.08975100 -1.30138100<br>H -0.06855600 -2.30494700 -0.13579700<br>H -0.75084000 1.92102300 -0.00348300<br>H -2.56094200 1.36016900 -1.16905100<br>H 0.34702800 3.63020400 0.98859600<br>H 0.37542500 3.70844200 -0.79581400<br>H 1.62400500 4.55028700 0.15502000<br>H 1.56482500 -3.58438200 -1.05026400<br>H 1.53883400 -3.64454100 0.73474400<br>H 3.02902400 -4.05301300 -0.15100300<br>H -4.87202400 -0.18436800 -0.39436900<br>H -4.74488300 0.61436300 -1.97171100<br>H -4.24320900 -1.08245300 -1.76352200<br>C -2.03946700 -0.49022800 -0.08970700<br>H -2.25987300 -1.55221200 -0.21973600<br>N -2.62937600 -0.10157500 1.28470200<br>O -3.55109500 -0.79872800 1.61912900	• Electronic Energy<br>= -782.527225<br>• Zero Point Energy Correction<br>= 0.225734<br>• Thermal Correction to Energy<br>= 0.242337<br>• Thermal Correction to Enthalpy<br>= 0.243281<br>• Thermal Correction to Free Energy<br>= 0.179813

Name	HPns RAF C-2a···$NO^{\bullet}$ Product
Cartesian Coordinate:	Thermochemical Values:
O -2.95600600 -1.86026300 0.11512100<br>O -1.67134700 2.59848900 -0.00691100<br>O -3.53026500 0.70957100 0.17334100<br>C 0.45950900 -0.43013300 -0.20231000<br>C -1.89540500 -1.01173900 0.01730500<br>C -1.21092000 1.31631900 -0.04847400<br>C 0.11495400 0.94571400 -0.17232300<br>C -0.57428200 -1.39950900 -0.10217100<br>C -2.22438000 0.35160000 0.04818200<br>C 1.79123500 -0.88138500 -0.33467200<br>C -2.67435400 -3.25651200 0.07072800<br>C -0.70870300 3.64582700 -0.10270700<br>C 4.11950400 -0.62968300 -1.23698500<br>H 0.88045400 1.70556000 -0.24097200<br>H -0.30526100 -2.44734200 -0.12369200<br>H 1.97104000 -1.95197900 -0.30333200<br>H -3.58503500 1.67613000 0.20019900<br>H -2.03237400 -3.54866000 0.90497300<br>H -2.19988500 -3.52514800 -0.87610600<br>H -3.63632600 -3.75651000 0.15498800<br>H -0.17226400 3.58833000 -1.05244100<br>H -0.00310000 3.59616700 0.72962900<br>H -1.27280000 4.57395900 -0.05318300<br>H 3.80976900 -0.66519600 -2.28236800<br>H 5.02877900 -0.03041100 -1.16232400<br>H 4.34082000 -1.64588500 -0.90638100	• Electronic Energy<br>= -783.014810<br>• Zero Point Energy Correction<br>= 0.239616<br>• Thermal Correction to Energy<br>= 0.256222<br>• Thermal Correction to Enthalpy<br>= 0.257166<br>• Thermal Correction to Free Energy<br>= 0.194333

C 3.00299800 -0.02280900 -0.41536700<br>H 2.75656000 0.98517300 -0.75399600<br>N 3.36239200 0.16935700 1.04448500<br>O 4.36786100 -0.38602500 1.38424600	

Name	Anionic HPns RAF C-2a···NO• Product
Cartesian Coordinate:	Thermochemical Values:
O -2.85562500 -1.96486400 0.02244500<br>O -1.80235200 2.60793500 0.08544400<br>O -3.54686300 0.60289400 0.16487400<br>C 0.47049400 -0.31442700 -0.20642600<br>C -1.84703500 -1.04323600 -0.03193300<br>C -1.29605300 1.34159200 -0.00138800<br>C 0.03462900 1.04409300 -0.12720300<br>C -0.51855400 -1.34650500 -0.14642200<br>C -2.31887000 0.32048300 0.05109500<br>C 1.81057300 -0.67871500 -0.34723600<br>C -2.48092600 -3.33398300 -0.05250300<br>C -0.87239000 3.68231300 0.04558900<br>C 4.10936400 -0.15005200 -1.28526400<br>H 0.76623300 1.83933500 -0.16114600<br>H -0.18311300 -2.37485600 -0.20025900<br>H 2.04365900 -1.74004800 -0.38844000<br>H -1.83145500 -3.60627900 0.78362600<br>H -1.97334500 -3.54786300 -0.99690900<br>H -3.40622600 -3.90336600 0.00253200<br>H -0.32053800 3.68563600 -0.89808700<br>H -0.17086200 3.62173400 0.88191700<br>H -1.46138500 4.59320000 0.12764700<br>H 3.81828100 0.11629200 -2.30300000<br>H 5.02076100 0.39636600 -1.03070400<br>H 4.32344400 -1.21884400 -1.25630400<br>C 2.98909800 0.21453800 -0.33735700<br>H 2.71624200 1.26529900 -0.41887200<br>N 3.24360900 -0.05000500 1.10828700<br>O 4.13872400 -0.89511300 1.31902200	• Electronic Energy = -782.551841<br>• Zero Point Energy Correction = 0.227651<br>• Thermal Correction to Energy = 0.243511<br>• Thermal Correction to Enthalpy = 0.244455<br>• Thermal Correction to Free Energy = 0.183680

Name	HPns RAF C-1···NOO• (Model 1) Product
Cartesian Coordinate:	Thermochemical Values:
O 1.85256500 -2.09459500 -0.57474600<br>O 0.77715400 2.45543700 -0.62012800<br>O 2.19879300 0.35786700 -1.59505400<br>C -1.45263400 -0.46511700 -0.13757500<br>C 0.84120200 -1.20836300 -0.45075200<br>C 0.25702200 1.21946700 -0.45474900<br>C -1.04365900 0.90112300 -0.26434800<br>C -0.47192600 -1.49373200 -0.25587900<br>C -2.82243400 -0.84442300 0.08854000<br>C -3.87975300 -0.01701400 0.24281900<br>C 1.51553700 -3.47813200 -0.49220500<br>C -0.13515200 3.55253600 -0.62883900<br>C -5.27523000 -0.49401600 0.47650200<br>H -1.78503900 1.68781800 -0.22104500<br>H -0.80019600 -2.52353500 -0.18641600<br>H -3.00521900 -1.91629800 0.14103500<br>H -3.73881600 1.05983700 0.20148700<br>H 2.37993800 1.30381900 -1.70244100<br>H 0.82174800 -3.74934300 -1.29094900<br>H 1.07299200 -3.70432900 0.48066600<br>H 2.44992800 -4.02052100 -0.61155000<br>H -0.65249100 3.62204200 0.33045100<br>H -0.85932500 3.43584200 -1.43806100<br>H 0.46799000 4.44183000 -0.79221900<br>H -5.66523100 -0.10369200 1.42187300<br>H -5.94564100 -0.13225100 -0.30949700<br>H -5.32465100 -1.58384100 0.50264000<br>C 1.35240300 0.19611400 -0.50348800<br>O 2.27467500 0.42606300 0.65285500<br>N 1.63657300 0.28679300 1.84548200<br>O 2.37378000 0.47578000 2.74151000	• Electronic Energy = -858.197536<br>• Zero Point Energy Correction = 0.242716<br>• Thermal Correction to Energy = 0.260702<br>• Thermal Correction to Enthalpy = 0.261646<br>• Thermal Correction to Free Energy = 0.196026

Name	HPns RAF C-2···NOO• (Model 1) Product

Cartesian Coordinate:	Thermochemical Values:
O -1.92310600 2.53760500 -0.02179000<br>O -1.27040900 -1.75121200 -1.43608700<br>O -2.89949100 0.11633200 -0.37140900<br>C 1.23212500 0.55917600 -0.32562200<br>C -1.01555000 1.53006500 -0.15605000<br>C 0.73364100 -0.69090200 -0.50284200<br>C 0.35984800 1.69047900 -0.14793700<br>C -1.56495600 0.26339600 -0.33092500<br>C 2.68367200 0.83208500 -0.30472200<br>C 3.61910400 -0.03659100 0.08477200<br>C -1.40508400 3.85814100 0.12269200<br>C -0.54922800 -2.94078900 -1.78045500<br>C 5.08518200 0.25462400 0.09694700<br>H 1.38396300 -1.54332000 -0.65551500<br>H 0.80287000 2.66851500 -0.01850700<br>H 2.98037700 1.83595300 -0.60239800<br>H 3.31614400 -1.02351600 0.42976900<br>H -3.12407000 -0.80530400 -0.57381800<br>H -0.81292600 4.13855700 -0.75146600<br>H -0.79749400 3.93830800 1.02692400<br>H -2.27220600 4.50919300 0.20383400<br>H -0.27480700 -3.50248200 -0.88610000<br>H 0.34091500 -2.69323500 -2.36061800<br>H -1.22894200 -3.53240600 -2.38953600<br>H 5.49390800 0.13503000 1.10433300<br>H 5.62060300 -0.45070200 -0.54556200<br>H 5.29263200 1.26854300 -0.24893400<br>C -0.72088300 -0.96087500 -0.42963300<br>O -1.01537600 -1.81725200 0.79920200<br>N -0.58384000 -1.22794400 1.93408500<br>O -0.81428200 -1.89802000 2.87654000	• Electronic Energy<br>= -858.179509<br>• Zero Point Energy Correction<br>= 0241881<br>• Thermal Correction to Energy<br>= 0.260072<br>• Thermal Correction to Enthalpy<br>= 0.261017<br>• Thermal Correction to Free Energy<br>= 0.195110

Name	HPns RAF C-3···NOO$^{\bullet}$ (Model 1) Product
Cartesian Coordinate:	Thermochemical Values:
O 2.91264100 -1.65109800 0.12745200<br>O 0.93202400 2.52100500 -0.51828200<br>O 3.11147200 0.93234600 -0.23271800<br>C -0.67069800 -0.89277300 -0.33625000<br>C 1.73895800 -1.01668900 -0.06920700<br>C 0.75240000 1.16582800 -0.44114400<br>C 0.49128000 -1.62601800 -0.12656400<br>C 1.85346300 0.40322500 -0.25574300<br>C -1.94434800 -1.55865700 -0.45255600<br>C -3.13981700 -0.96909300 -0.65492900<br>C 2.87562800 -3.06629300 0.31140600<br>C 0.05813200 3.23211200 -1.40275500<br>C -4.42420300 -1.72236500 -0.78050200<br>H 0.40833200 -2.70168300 -0.03118300<br>H -1.91192700 -2.64344000 -0.36672800<br>H -3.20515500 0.11334200 -0.73642600<br>H 3.03759600 1.88926600 -0.36100800<br>H 2.46896800 -3.55630400 -0.57554100<br>H 2.27859200 -3.32144700 1.18937700<br>H 3.90776600 -3.37166400 0.46292600<br>H -0.96106700 3.24783800 -1.01105700<br>H 0.07408200 2.78150300 -2.39761100<br>H 0.44369600 4.24784700 -1.45118200<br>H -5.13873100 -1.40142400 -0.01616900<br>H -4.89516000 -1.52789400 -1.74910600<br>H -4.26626600 -2.79755000 -0.68013400<br>C -0.62314900 0.60290700 -0.41236800<br>O -1.37433500 1.22018800 0.72450600<br>H -1.22480600 0.96931200 -1.24842800<br>N -0.84675000 0.84966200 1.91511000<br>O -1.44361900 1.32900300 2.81031300	• Electronic Energy<br>= -858.188996<br>• Zero Point Energy Correction<br>= 0.243366<br>• Thermal Correction to Energy<br>= 0.261278<br>• Thermal Correction to Enthalpy<br>= 0.262222<br>• Thermal Correction to Free Energy<br>= 0.197133

Name	Anionic HPns RAF C-3···NOO$^{\bullet}$ (Model 1) Product
Cartesian Coordinate:	Thermochemical Values:
O 2.94224700 -1.60724100 0.10620400<br>O 0.85814000 2.59155000 -0.48680000<br>O 3.11571300 1.02247600 -0.20633100<br>C -0.63635600 -0.86966000 -0.35870400<br>C 1.77165100 -0.95809300 -0.08685800	• Electronic Energy<br>= -857.717743<br>• Zero Point Energy Correction

C 0.76498400 1.20464100 -0.43021600<br>C 0.54048000 -1.59001200 -0.16383000<br>C 1.92957300 0.49765100 -0.24252900<br>C -1.89987000 -1.54718600 -0.48922700<br>C -3.10806000 -0.97186600 -0.66675300<br>C 2.90374300 -3.02242200 0.26326400<br>C 0.07307700 3.22104900 -1.49533400<br>C -4.38170900 -1.74274700 -0.80480900<br>H 0.47162700 -2.66921100 -0.09459200<br>H -1.85329500 -2.63407200 -0.43507000<br>H -3.19024300 0.11128700 -0.71713600<br>H 2.50385300 -3.49985700 -0.63415300<br>H 2.30065200 -3.29768900 1.13157600<br>H 3.93430100 -3.33377300 0.41695300<br>H -0.98861800 3.22331900 -1.23176100<br>H 0.21099300 2.72324400 -2.45972200<br>H 0.42150200 4.25072900 -1.56396500<br>H -5.09515200 -1.46190600 -0.02361100<br>H -4.86693000 -1.52734400 -1.76202600<br>H -4.20393700 -2.81784900 -0.74112500<br>C -0.58860100 0.62156100 -0.39522000<br>O -1.35918800 1.19191100 0.80280800<br>H -1.23665900 1.01467200 -1.18275700<br>N -0.82439300 0.78467400 1.95599100<br>O -1.41326300 1.22233200 2.88746300	= 0.230150<br>• Thermal Correction to Energy<br>= 0.247841<br>• Thermal Correction to Enthalpy<br>= 0.248785<br>• Thermal Correction to Free Energy<br>= 0.183465

Name	HPns RAF C-4···NOO$^{\bullet}$ (Model 1) Product
Cartesian Coordinate:	Thermochemical Values:
O 2.47921100 -1.62019800 -0.92114500<br>O 1.06864000 2.75141000 -0.23885500<br>O 2.92053400 0.96754000 -0.85341200<br>C 1.42302700 -0.86199500 -0.52754200<br>C 0.65637600 1.45841900 -0.17126400<br>C -0.58646400 1.03444700 0.16466800<br>C 0.19707700 -1.33241300 -0.18144200<br>C 1.69110300 0.53727000 -0.51357300<br>C -2.19241800 -0.81560600 -0.43699700<br>C -2.89264400 -0.04390200 -1.26325900<br>C 2.28113400 -3.03145100 -0.94837000<br>C 0.11251200 3.75606100 0.09398600<br>C -4.13793300 -0.48402600 -1.96543700<br>H -1.37591200 1.72782300 0.42130300<br>H -0.03521200 -2.38907300 -0.19780700<br>H -2.50439700 -1.83950100 -0.23893000<br>H -2.55238000 0.96956200 -1.46400100<br>H 2.94033600 1.93591900 -0.80136400<br>H 1.49291400 -3.29517700 -1.65731200<br>H 2.02621800 -3.40259200 0.04702100<br>H 3.22704200 -3.45891500 -1.27188000<br>H -0.22633100 3.63229000 1.12490500<br>H -0.73902500 3.71199000 -0.58863400<br>H 0.62765500 4.70694900 -0.01640900<br>H -4.97379300 0.17298600 -1.70987200<br>H -4.00730100 -0.42279100 -3.04956100<br>H -4.40396500 -1.50890300 -1.70233500<br>C -0.90437400 -0.41753600 0.24219600<br>O -1.24775500 -0.74093100 1.69866000<br>N -0.19597900 -0.55001700 2.51165400<br>O -0.47167600 -0.79357700 3.63544000	• Electronic Energy<br>= -858.180464<br>• Zero Point Energy Correction<br>= 0.242272<br>• Thermal Correction to Energy<br>= 0.260318<br>• Thermal Correction to Enthalpy<br>= 0.261263<br>• Thermal Correction to Free Energy<br>= 0.195674

Name	HPns RAF C-1a···NOO$^{\bullet}$ (Model 1) Product
Cartesian Coordinate:	Thermochemical Values:
O 3.05664600 -1.77155900 0.17477800<br>O 1.63664000 2.63496300 -0.16939800<br>O 3.55726900 0.81947200 0.13617000<br>C -0.36573600 -0.46065300 -0.27515100<br>C 1.97829700 -0.95359400 0.02139600<br>C 1.22549600 1.33518100 -0.16043100<br>C -0.09005300 0.90733500 -0.30135400<br>C 0.66010600 -1.38903000 -0.11255500<br>C 2.26090100 0.41123900 0.00142400<br>C -2.60757900 -0.33478400 -1.44933100<br>C 2.81137300 -3.17558500 0.18275400<br>C 0.63644200 3.63759300 -0.33491000<br>C -3.99302500 -0.81810800 -1.68697900	• Electronic Energy<br>= -858.196673<br>• Zero Point Energy Correction<br>= 0.242447<br>• Thermal Correction to Energy<br>= 0.260817<br>• Thermal Correction to Enthalpy<br>= 0.261761

H -0.88833900 1.62674800 -0.42821500<br>H 0.42597300 -2.44548000 -0.09362700<br>H -2.23281700 0.53838000 -1.96638200<br>H 3.57848700 1.78641900 0.09673000<br>H 2.36434300 -3.49587600 -0.76124100<br>H 2.16065300 -3.45058900 1.01595400<br>H 3.78396400 -3.64604900 0.30638300<br>H -0.08621000 3.59750300 0.48326600<br>H 0.12521600 3.51539900 -1.29238900<br>H 1.16241000 4.58895800 -0.31615600<br>H -4.70837600 -0.33748300 -1.00483200<br>H -4.32129300 -0.59602000 -2.70366200<br>H -4.06820200 -1.89611400 -1.51618500<br>C -1.78212200 -0.96154300 -0.39103300<br>O -2.46192200 -0.86549600 0.93892400<br>H -1.77904900 -2.04838800 -0.50926100<br>N -2.81220800 0.40231800 1.23797400<br>O -3.31812100 0.46487800 2.30212800	• Thermal Correction to Free Energy<br>= 0.194364

Name	Anionic HPns RAF C-1a···NOO$^{\bullet}$ (Model 1) Product
Cartesian Coordinate:	Thermochemical Values:
O 3.09539900 -1.74962000 0.15197900<br>O 1.62513800 2.69918900 -0.12034200<br>O 3.57738200 0.88838900 0.16392600<br>C -0.33201800 -0.43321700 -0.31766700<br>C 2.01608600 -0.90631600 0.00092100<br>C 1.26126700 1.37117700 -0.14156600<br>C -0.04681700 0.93626700 -0.30752000<br>C 0.70884300 -1.35074600 -0.15738600<br>C 2.35720700 0.47435200 0.01782300<br>C -2.59462200 -0.26201500 -1.44776900<br>C 2.83365400 -3.14447100 0.13994600<br>C 0.59038900 3.65772800 -0.27742700<br>C -3.97659400 -0.75306600 -1.69276800<br>H -0.85007500 1.65281400 -0.42470400<br>H 0.48144400 -2.40999800 -0.16227900<br>H -2.25122300 0.65587300 -1.90580400<br>H 2.39264500 -3.45450000 -0.81161700<br>H 2.16827800 -3.42776600 0.96028100<br>H 3.79680100 -3.63451900 0.26933000<br>H -0.14605300 3.57797400 0.52725300<br>H 0.08982200 3.54252700 -1.24296300<br>H 1.07175800 4.63279800 -0.23286900<br>H -4.68904000 -0.32105700 -0.97576800<br>H -4.32405800 -0.48128700 -2.69108600<br>H -4.03395200 -1.83977200 -1.58021400<br>C -1.73596800 -0.93829100 -0.44929900<br>O -2.42192700 -0.93782100 0.90153000<br>H -1.72906100 -2.01744900 -0.62538700<br>N -2.76438300 0.29774900 1.28904700<br>O -3.26538700 0.29132700 2.36150300	• Electronic Energy<br>= -857.725682<br>• Zero Point Energy Correction<br>= 0.229928<br>• Thermal Correction to Energy<br>= 0.247734<br>• Thermal Correction to Enthalpy<br>= 0.248679<br>• Thermal Correction to Free Energy<br>= 0.182478

Name	HPns RAF C-2a···NOO$^{\bullet}$ (Model 1) Product
Cartesian Coordinate:	Thermochemical Values:
O -3.28940000 -1.61875300 0.31328900<br>O -1.54685000 2.65879700 -0.15004600<br>O -3.57196500 1.00232800 0.29500400<br>C 0.20716500 -0.59785600 -0.41744600<br>C -2.15813000 -0.89958400 0.07012000<br>C -1.23431000 1.33208400 -0.17667000<br>C 0.02298500 0.80887700 -0.41419300<br>C -0.90730500 -1.43994200 -0.16511500<br>C -2.33131600 0.49193600 0.06497200<br>C 1.46865900 -1.18526800 -0.65958000<br>C -3.16793900 -3.03853000 0.30329700<br>C -0.49315700 3.58595300 -0.40018600<br>C 3.87000100 -1.26133100 -1.39513400<br>H 0.86108600 1.46815700 -0.59467500<br>H -0.75832400 -2.51165600 -0.16623400<br>H 1.55135500 -2.26630500 -0.63502100<br>H -3.52290100 1.96752200 0.23239600<br>H -2.47893800 -3.37398800 1.08192800<br>H -2.82611800 -3.39031900 -0.67292700<br>H -4.16489000 -3.42354300 0.50440700<br>H -0.07229900 3.42855000 -1.39576300	• Electronic Energy<br>= -858.216356<br>• Zero Point Energy Correction<br>= 0.243706<br>• Thermal Correction to Energy<br>= 0.261362<br>• Thermal Correction to Enthalpy<br>= 0.262306<br>• Thermal Correction to Free Energy<br>= 0.196840

H 0.28924800 3.49096100 0.35603800<br>H -0.94459000 4.57334100 -0.34181900<br>H 3.61445300 -1.74723500 -2.33858700<br>H 4.75820600 -0.64684000 -1.54693600<br>H 4.08818200 -2.03436800 -0.65539600<br>C 2.70395800 -0.40449200 -0.94872200<br>O 3.10658700 0.41223900 0.22031400<br>H 2.50958400 0.38225800 -1.68060500<br>N 3.26860700 -0.34434000 1.32403800<br>O 3.59313200 0.31323400 2.24854700	

Name	Anionic HPns RAF C-2a···NOO• (Model 1) Product
Cartesian Coordinate:	Thermochemical Values:
O -3.35873000 -1.57396700 0.31164500<br>O -1.52718600 2.72441600 -0.11901500<br>O -3.59506700 1.08814000 0.30375100<br>C 0.15741100 -0.58139100 -0.41780800<br>C -2.21960000 -0.84786500 0.07124900<br>C -1.27212000 1.37717600 -0.15626300<br>C -0.03053900 0.83695000 -0.39608300<br>C -0.98781700 -1.40334400 -0.16540100<br>C -2.43926500 0.57414100 0.08677100<br>C 1.39843800 -1.17089900 -0.66157800<br>C -3.23608700 -2.98891200 0.31119100<br>C -0.43351400 3.60077100 -0.34946400<br>C 3.77503400 -1.25189100 -1.47492800<br>H 0.82001800 1.48418500 -0.56394000<br>H -0.85375900 -2.47798000 -0.17366500<br>H 1.48210200 -2.25247400 -0.62513000<br>H -2.54546500 -3.32258600 1.09040700<br>H -2.89327800 -3.35195300 -0.66147100<br>H -4.23138500 -3.37845800 0.51547000<br>H -0.00822700 3.44340600 -1.34441000<br>H 0.34411000 3.46515000 0.40704600<br>H -0.83569400 4.60953000 -0.28096900<br>H 3.49598200 -1.67297200 -2.44254500<br>H 4.67590300 -0.64994800 -1.60316100<br>H 3.98825900 -2.07492500 -0.78945100<br>C 2.63225100 -0.40294100 -0.95647900<br>O 3.11136900 0.36728900 0.24073400<br>H 2.44272400 0.43052300 -1.63590400<br>N 3.33425800 -0.43109400 1.28885400<br>O 3.70883200 0.18354600 2.23019800	• Electronic Energy = -857.750923<br>• Zero Point Energy Correction = 0.231463<br>• Thermal Correction to Energy = 0.248619<br>• Thermal Correction to Enthalpy = 0.249563<br>• Thermal Correction to Free Energy = 0.185090

Name	HPns RAF C-1···NOO• (Model 2) Product
Cartesian Coordinate:	Thermochemical Values:
O 1.72564400 -2.16639300 -0.40975900<br>O 0.84427700 2.40999100 -0.40332400<br>O 2.20593700 0.27812400 -1.40369400<br>C -1.53385200 -0.41923800 -0.10406800<br>C 0.73748300 -1.25068200 -0.33560500<br>C 0.25578300 1.19773100 -0.31134700<br>C -1.06269600 0.93005700 -0.17192600<br>C -0.59241700 -1.48542900 -0.19986300<br>C -2.92713500 -0.74583300 0.05125000<br>C -3.95463200 0.12326800 0.17091300<br>C 1.34223800 -3.53941800 -0.33505700<br>C -0.01116000 3.55366100 -0.39768000<br>C -5.37818600 -0.29646000 0.32996200<br>H -1.76881200 1.74857800 -0.12834300<br>H -0.96234100 -2.50252000 -0.16029500<br>H -3.15524000 -1.80980500 0.07516100<br>H -3.76679000 1.19357600 0.15487400<br>H 2.39146800 1.22151200 -1.52930900<br>H 0.68152200 -3.79394100 -1.16638800<br>H 0.84442800 -3.74190600 0.61588300<br>H 2.26430500 -4.11059100 -0.40325800<br>H -0.56108700 3.60806300 0.54413200<br>H -0.70692500 3.50740500 -1.23820100<br>H 0.64276500 4.41562300 -0.49912600<br>H -5.79621200 0.09887100 1.26109300<br>H -5.99274800 0.10509700 -0.48192500<br>H -5.47542900 -1.38324500 0.33698600<br>C 1.29431800 0.12797900 -0.38308300<br>N 2.16036500 0.30007700 0.99405300	• Electronic Energy = -858.202059<br>• Zero Point Energy Correction = 0.244231<br>• Thermal Correction to Energy = 0.261896<br>• Thermal Correction to Enthalpy = 0.262840<br>• Thermal Correction to Free Energy = 0.197799

O 1.59829300 0.00579400 2.02490300<br>O 3.27835700 0.74360000 0.91429400	

Name	HPns RAF C-3···NOO• (Model 2) Product
Cartesian Coordinate:	Thermochemical Values:
O 3.02537500 -1.52405000 0.10163200<br>O 0.78275400 2.56225500 -0.23429000<br>O 3.06632300 1.08387300 -0.06803900<br>C -0.59529400 -0.94938000 -0.29388000<br>C 1.81832000 -0.94835300 -0.04001300<br>C 0.69981800 1.19533200 -0.23844200<br>C 0.60953000 -1.62470300 -0.15858700<br>C 1.84536400 0.48795900 -0.10939400<br>C -1.83550600 -1.67071600 -0.43516300<br>C -3.06269300 -1.12252700 -0.53332100<br>C 3.07513500 -2.95063800 0.16066800<br>C 0.11518800 3.22843800 -1.31817100<br>C -4.31931400 -1.91929100 -0.66468900<br>H 0.59346900 -2.70754800 -0.16678800<br>H -1.74792600 -2.75535300 -0.44524000<br>H -3.18279700 -0.04086500 -0.52871700<br>H 2.93614900 2.04208300 -0.12814500<br>H 2.69795500 -3.38374800 -0.76769100<br>H 2.49509300 -3.31609000 1.01048400<br>H 4.12418800 -3.20375500 0.28934900<br>H -0.96737800 3.19452200 -1.18387300<br>H 0.39453100 2.77012500 -2.26916500<br>H 0.45259600 4.26205200 -1.28938700<br>H -5.00466900 -1.69853000 0.15933000<br>H -4.84656600 -1.66032200 -1.58792100<br>H -4.11200900 -2.99051700 -0.66891100<br>C -0.63222500 0.53933300 -0.23932800<br>H -1.31070100 0.97842000 -0.97440900<br>N -1.32084000 0.99582700 1.11494300<br>O -1.11746700 0.34346500 2.11355500<br>O -1.95523600 2.02770900 1.08319300	• Electronic Energy<br>= -858.196853<br>• Zero Point Energy Correction<br>= 0.244680<br>• Thermal Correction to Energy<br>= 0.262417<br>• Thermal Correction to Enthalpy<br>= 0.263362<br>• Thermal Correction to Free Energy<br>= 0.198154

Name	Anionic HPns RAF C-3···NOO• (Model 2) Product
Cartesian Coordinate:	Thermochemical Values:
O 2.87254600 -1.72532100 0.15891800<br>O 0.85580700 2.56625400 -0.19347900<br>O 3.11087300 0.91856800 0.05676500<br>C -0.67602800 -0.85823200 -0.31648800<br>C 1.72434700 -1.03496500 0.00164600<br>C 0.77427200 1.17769800 -0.16021400<br>C 0.48124700 -1.62169900 -0.17022500<br>C 1.91925600 0.42949700 -0.01659800<br>C -1.95870200 -1.48483100 -0.49578400<br>C -3.14726300 -0.85549800 -0.61257100<br>C 2.80196000 -3.14904500 0.16838200<br>C 1.05337900 3.06794200 -1.51769900<br>C -4.45019500 -1.56835100 -0.78248600<br>H 0.38475400 -2.70031900 -0.20793600<br>H -1.94844100 -2.57327800 -0.52290400<br>H -3.19618900 0.23139700 -0.59516600<br>H 2.42048200 -3.52121800 -0.78495300<br>H 2.16684400 -3.49697000 0.98612500<br>H 3.82115800 -3.49680400 0.31854200<br>H 0.22216400 2.77399100 -2.16563100<br>H 1.99237600 2.68991500 -1.93042300<br>H 1.09352100 4.15490800 -1.44912500<br>H -5.13999700 -1.31690100 0.02916900<br>H -4.94049700 -1.26727900 -1.71348700<br>H -4.31139700 -2.65079800 -0.79686100<br>C -0.58718900 0.62345700 -0.21781400<br>H -1.21966400 1.15263200 -0.93619000<br>N -1.30869100 1.06880500 1.15051300<br>O -1.09806700 0.42415800 2.15635700<br>O -1.96550800 2.08924600 1.12573300	• Electronic Energy<br>= -857.729004<br>• Zero Point Energy Correction<br>= 0.231298<br>• Thermal Correction to Energy<br>= 0.248812<br>• Thermal Correction to Enthalpy<br>= 0.249756<br>• Thermal Correction to Free Energy<br>= 0.184729

Name	HPns RAF C-4⋯NOO$^{\bullet}$ (Model 2) Product
Cartesian Coordinate:	Thermochemical Values:
O 2.50955000 -1.85437200 -0.56342900<br>O 1.46127800 2.64464500 -0.12866600<br>O 3.19910800 0.67442200 -0.44092600<br>C 1.49864300 -0.98988000 -0.30040100<br>C 0.92564200 1.39959800 -0.07788300<br>C -0.38677600 1.10458600 0.09938000<br>C 0.19568500 -1.33081900 -0.11967000<br>C 1.90235900 0.37593800 -0.25696700<br>C -2.13376700 -0.61630100 -0.52287600<br>C -2.79184400 0.22643800 -1.31421000<br>C 2.17444900 -3.23950000 -0.61595500<br>C 0.57219300 3.74593400 0.05467300<br>C -4.04829200 -0.12739800 -2.04352600<br>H -1.12521500 1.88238700 0.22797700<br>H -0.13730500 -2.35733300 -0.17959300<br>H -2.49391100 -1.63240100 -0.37754800<br>H -2.40833800 1.23188500 -1.46867700<br>H 3.31039200 1.63800800 -0.41950800<br>H 1.46039300 -3.43114100 -1.41998700<br>H 1.75958400 -3.57003800 0.33904000<br>H 3.10586600 -3.76317600 -0.81641000<br>H 0.09203500 3.68763500 1.03377400<br>H -0.18283400 3.76012300 -0.73441100<br>H 1.18856800 4.63925300 -0.00493500<br>H -4.85659900 0.55220400 -1.75986600<br>H -3.90584500 -0.01711800 -3.12217200<br>H -4.35862000 -1.15162500 -1.83245300<br>C -0.83515400 -0.30153800 0.18261400<br>N -1.24941400 -0.56728100 1.76083500<br>O -1.32309200 0.37484900 2.51563900<br>O -1.48948300 -1.71205100 2.07961500	• Electronic Energy<br>= -858.186966<br>• Zero Point Energy Correction<br>= 0.243963<br>• Thermal Correction to Energy<br>= 0.261815<br>• Thermal Correction to Enthalpy<br>= 0.262759<br>• Thermal Correction to Free Energy<br>= 0.195829

Name	HPns RAF C-1a⋯NOO$^{\bullet}$ (Model 2) Product
Cartesian Coordinate:	Thermochemical Values:
O 1.73123100 2.64598100 -0.01535900<br>O 2.90402400 -1.84590200 -0.06883300<br>O 3.54758100 0.73575800 0.01990600<br>C -0.44703800 -0.32244000 -0.19865000<br>C 1.25883300 1.37128500 -0.07096100<br>C 1.85946500 -0.97171400 -0.09613100<br>C 0.51457900 -1.32733000 -0.16411900<br>C -0.08722900 1.02432100 -0.14882000<br>C 2.23424000 0.37107300 -0.04817600<br>C -2.67886400 -0.06291900 -1.35798400<br>C 0.76864900 3.69745800 -0.03202500<br>C 2.59599300 -3.23760200 -0.11721300<br>C -4.04309400 -0.54483900 -1.69064600<br>H 0.21392100 -2.36623400 -0.19576900<br>H -0.85264800 1.79035800 -0.16514600<br>H -2.29658600 0.85057800 -1.79464500<br>H 4.08815700 -0.06726200 0.02349500<br>H 0.10049600 3.62398400 0.82918400<br>H 0.18841300 3.67661100 -0.95740300<br>H 1.33866300 4.62178600 0.02313800<br>H 2.07085100 -3.48227600 -1.04315100<br>H 1.99053100 -3.52649100 0.74463000<br>H 3.55154800 -3.75527900 -0.08695500<br>H -4.79050600 -0.10480600 -1.01481800<br>H -4.32447000 -0.26681100 -2.70732400<br>H -4.11550700 -1.63080200 -1.58428400<br>C -1.90395100 -0.70353600 -0.27783900<br>H -2.01737200 -1.78942000 -0.28481500<br>N -2.51244900 -0.33789900 1.11986500<br>O -3.17552200 0.67104300 1.21799800<br>O -2.25579500 -1.07827700 2.04506200	• Electronic Energy<br>= -858.207066<br>• Zero Point Energy Correction<br>= 0.244352<br>• Thermal Correction to Energy<br>= 0.262287<br>• Thermal Correction to Enthalpy<br>= 0.263232<br>• Thermal Correction to Free Energy<br>= 0.196384

Name	Anionic HPns RAF C-1a⋯NOO$^{\bullet}$ (Model 2) Product
Cartesian Coordinate:	Thermochemical Values:

O 1.80647000 2.65404900 0.06809800<br>O 2.88985900 -1.91130300 -0.11829700<br>O 3.59896000 0.66605300 0.08615600<br>C -0.42123500 -0.27509300 -0.27589900<br>C 1.32538100 1.36900800 -0.04319300<br>C 1.88275100 -0.97189200 -0.13481400<br>C 0.53567700 -1.29101900 -0.25038500<br>C -0.02200500 1.06058200 -0.16487700<br>C 2.34359600 0.37057300 -0.02384500<br>C -2.69965100 0.14861800 -1.30602700<br>C 0.85260700 3.70540300 0.06129400<br>C 2.50418800 -3.27445500 -0.20859500<br>C -4.09440700 -0.26580000 -1.60492000<br>H 0.21261200 -2.32193100 -0.32632300<br>H -0.76907700 1.84512000 -0.16322600<br>H -2.32522800 1.10270200 -1.65297200<br>H 0.16041500 3.61429200 0.90312200<br>H 0.28861500 3.71922100 -0.87556300<br>H 1.41992100 4.62919400 0.15661400<br>H 1.98387200 -3.47540900 -1.14931900<br>H 1.86374600 -3.55834200 0.63115400<br>H 3.42553500 -3.85263300 -0.17375300<br>H -4.78536400 0.08575300 -0.82488100<br>H -4.43932900 0.15086500 -2.55226600<br>H -4.18337700 -1.35541000 -1.64200700<br>C -1.87576200 -0.63375200 -0.36362700<br>H -1.99927200 -1.70834500 -0.51516200<br>N -2.44587700 -0.47000400 1.09598200<br>O -3.02413600 0.55705900 1.38224800<br>O -2.23600000 -1.37061900 1.88204900	• Electronic Energy = -857.737014<br>• Zero Point Energy Correction = 0.231577<br>• Thermal Correction to Energy = 0.249051<br>• Thermal Correction to Enthalpy = 0.249995<br>• Thermal Correction to Free Energy = 0.183769

Name	HPns RAF C-2a···NOO• (Model 2) Product
Cartesian Coordinate:	Thermochemical Values:
O -3.23983000 -1.73904900 0.23091700<br>O -1.69193500 2.62058700 -0.13578400<br>O -3.64818100 0.86308500 0.20962600<br>C 0.23744600 -0.54163100 -0.30627600<br>C -2.13503800 -0.96498400 0.04953900<br>C -1.31161700 1.31245300 -0.14520700<br>C -0.01977000 0.85526800 -0.31597700<br>C -0.84902100 -1.43977500 -0.11764900<br>C -2.37689500 0.41748900 0.04046500<br>C 1.52579100 -1.08528100 -0.47829600<br>C -3.04829600 -3.15137900 0.22548500<br>C -0.67061700 3.60183900 -0.30099800<br>C 3.84993600 -1.05432600 -1.42359200<br>H 0.78651300 1.56170600 -0.45186900<br>H -0.64683500 -2.50263300 -0.10971100<br>H 1.64438400 -2.16264700 -0.42808000<br>H -3.64243500 1.83124300 0.17595800<br>H -2.38514300 -3.45514500 1.03867200<br>H -2.63960300 -3.48193400 -0.73225600<br>H -4.03428000 -3.58528000 0.37386600<br>H -0.17828700 3.48368500 -1.26877900<br>H 0.06410200 3.52935000 0.50419700<br>H -1.17306600 4.56495100 -0.25720300<br>H 3.50128100 -1.20218400 -2.44680800<br>H 4.78200400 -0.48878500 -1.45087000<br>H 4.02725900 -2.02937500 -0.96975100<br>C 2.77333700 -0.29700200 -0.67634900<br>H 2.58760800 0.68002900 -1.11883600<br>N 3.28379500 0.06093100 0.73268800<br>O 4.01352900 -0.72210500 1.30433800<br>O 2.87496800 1.09179200 1.22912700	• Electronic Energy = -858.226136<br>• Zero Point Energy Correction = 0.245643<br>• Thermal Correction to Energy = 0.262926<br>• Thermal Correction to Enthalpy = 0.263870<br>• Thermal Correction to Free Energy = 0.198905

Name	Anionic HPns RAF C-2a···NOO• (Model 2) Product
Cartesian Coordinate:	Thermochemical Values:
O -3.30525200 -1.67087600 0.22437800<br>O -1.61736000 2.69101300 -0.12377600<br>O -3.63675100 0.97879500 0.19983100<br>C 0.20579800 -0.54620300 -0.31695100<br>C -2.18380200 -0.90510300 0.04212100<br>C -1.30870800 1.35650000 -0.14189500<br>C -0.03767500 0.86459000 -0.31375900	• Electronic Energy = -857.763059<br>• Zero Point Energy Correction = 0.233519

C -0.92136800 -1.41154400 -0.12416300<br>C -2.45688100 0.50952000 0.04350900<br>C 1.46778100 -1.10927500 -0.50295300<br>C -3.13086600 -3.08084300 0.23613100<br>C -0.54648000 3.60959300 -0.29014300<br>C 3.81396900 -1.10842100 -1.41026800<br>H 0.79068300 1.54634200 -0.44461500<br>H -0.74535400 -2.47999600 -0.11948700<br>H 1.57164300 -2.18869000 -0.44920100<br>H -2.46971000 -3.38637500 1.05145900<br>H -2.72523400 -3.43261600 -0.71621600<br>H -4.12047400 -3.50601800 0.39035900<br>H -0.06217100 3.47465100 -1.26100900<br>H 0.19308500 3.49713800 0.50740100<br>H -0.98995600 4.60184700 -0.23902900<br>H 3.49590900 -1.23821300 -2.44583100<br>H 4.75829300 -0.56206300 -1.40229600<br>H 3.96103200 -2.09281100 -0.96507000<br>C 2.72583900 -0.34379500 -0.68657200<br>H 2.56688000 0.63943400 -1.12759700<br>N 3.22426000 0.00994200 0.73299600<br>O 3.91109700 -0.79486600 1.33519700<br>O 2.82825600 1.05189100 1.22434800	• Thermal Correction to Energy = 0.250207<br>• Thermal Correction to Enthalpy = 0.251151<br>• Thermal Correction to Free Energy = 0.187822

Name	HPns RAF C-2a ···NOO$^{\bullet}$ Transition States Structure
Cartesian Coordinate:	Thermochemical Values:
O -3.27039100 -1.59256300 0.37542600<br>O -1.52245700 2.65987600 -0.20177900<br>O -3.50113200 1.01217200 0.33827200<br>C 0.17545800 -0.60359100 -0.55053000<br>C -2.15348500 -0.90533500 0.06464800<br>C -1.20940200 1.34790000 -0.24966800<br>C 0.01419500 0.81035000 -0.54938900<br>C -0.92541200 -1.44507500 -0.23209300<br>C -2.30972800 0.50297600 0.05505600<br>C 1.41704700 -1.22383000 -0.83858000<br>C -3.16769800 -3.01734000 0.40345100<br>C -0.48055600 3.59472600 -0.49067100<br>C 3.83712100 -1.27087000 -1.51284600<br>H 0.85092800 1.45647200 -0.77336900<br>H -0.77388600 -2.51636600 -0.23386000<br>H 1.45842800 -2.30529100 -0.74030800<br>H -3.46497700 1.98212400 0.28267500<br>H -2.44011400 -3.33197800 1.15445600<br>H -2.88266800 -3.39791100 -0.57957200<br>H -4.15729900 -3.37888000 0.66984200<br>H -0.10873100 3.44340300 -1.50607300<br>H 0.33234300 3.48806100 0.23044200<br>H -0.93270700 4.57866600 -0.40082200<br>H 3.97009100 -1.26403800 -2.60114400<br>H 4.69269900 -0.74168600 -1.08777400<br>H 3.82774800 -2.30391500 -1.16536900<br>C 2.57599000 -0.56463700 -1.18435200<br>H 2.55100800 0.49921200 -1.38837000<br>O 4.41387400 0.18186800 1.44464000<br>N 3.23732400 0.11886700 1.07629900<br>O 2.35194100 0.18969800 1.93281500	• Electronic Energy = -858.200356<br>• Zero Point Energy Correction = 0.241451<br>• Thermal Correction to Energy = 0.259874<br>• Thermal Correction to Enthalpy = 0.260819<br>• Thermal Correction to Free Energy = 0.191131

Name	HPns RAF C-2a ···NOO$^{\bullet}$ Reactant Complex
Cartesian Coordinate:	Thermochemical Values:
O -2.79446700 -1.59719200 0.74467600<br>O -1.07880100 2.60872900 -0.13362300<br>O -2.96744500 1.00015600 0.74294900<br>C 0.42746500 -0.68707700 -0.79644500<br>C -1.75001000 -0.94780500 0.23034300<br>C -0.81789400 1.30229500 -0.25504000<br>C 0.30756900 0.73041200 -0.77582500<br>C -0.61035600 -1.50820300 -0.29069600<br>C -1.87573200 0.47778400 0.25133100<br>C 1.59828300 -1.34854100 -1.31016100<br>C -2.73827600 -3.02848400 0.75775400<br>C -0.05503500 3.52849300 -0.52983500<br>C 3.88005500 -1.46121200 -2.32394000	• Electronic Energy = -858.207498<br>• Zero Point Energy Correction = 0.241971<br>• Thermal Correction to Energy = 0.261210<br>• Thermal Correction to Enthalpy = 0.262155

H 1.10692600 1.35231500 -1.15154400<br>H -0.48268400 -2.58193800 -0.31697100<br>H 1.58203000 -2.43464300 -1.28113200<br>H -2.91807200 1.97343500 0.72601200<br>H -1.89254700 -3.36502900 1.35991100<br>H -2.65666000 -3.40955600 -0.26170300<br>H -3.67242600 -3.35293000 1.20709500<br>H 0.14977100 3.42408200 -1.59678100<br>H 0.85037700 3.34824700 0.05304800<br>H -0.45052400 4.51761700 -0.31759800<br>H 4.06152900 -1.18499900 -3.36761000<br>H 4.77268700 -1.15875700 -1.76704900<br>H 3.75710000 -2.54167100 -2.25150200<br>C 2.69416800 -0.73301500 -1.80919000<br>H 2.74169600 0.35153500 -1.84793400<br>O 1.18228000 0.20042100 2.35397300<br>N 0.52349000 1.20642500 2.67195100<br>O -0.70993400 1.04321000 2.72961100	• Thermal Correction to Free Energy<br>= 0.191863

Name	Anionic HPns RAF C-2a ···NOO$^{\bullet}$ Transition States Structure
Cartesian Coordinate:	Thermochemical Values:
O -3.47658400 -1.35184000 0.29453400<br>O -1.27569300 2.75792300 -0.21534800<br>O -3.47086300 1.31125900 0.18468900<br>C 0.10723900 -0.67549200 -0.39960700<br>C -2.28357600 -0.74275700 0.05125700<br>C -1.13219900 1.40548100 -0.22021100<br>C 0.05408500 0.74992700 -0.41887700<br>C -1.09736200 -1.39726400 -0.14463900<br>C -2.37711200 0.70196400 0.01573000<br>C 1.30819600 -1.39195100 -0.59226500<br>C -3.47889000 -2.77356000 0.35120000<br>C -0.10492500 3.53866000 -0.42648600<br>C 3.72688000 -1.67058300 -1.21657400<br>H 0.96190200 1.31449700 -0.58260100<br>H -1.04565800 -2.47849200 -0.11703200<br>H 1.27625800 -2.46792100 -0.44406100<br>H -2.83297900 -3.13026500 1.15747800<br>H -3.15171700 -3.20025900 -0.60024700<br>H -4.50798900 -3.06440900 0.54952000<br>H 0.33173300 3.32609200 -1.40559400<br>H 0.63345100 3.34921800 0.35677700<br>H -0.42581700 4.57699500 -0.38507600<br>H 3.66961800 -1.93132400 -2.27751200<br>H 4.66653300 -1.14053900 -1.05638700<br>H 3.72615900 -2.59412500 -0.63532000<br>C 2.54915100 -0.81098700 -0.86421000<br>H 2.57135400 0.18716300 -1.29394900<br>O 4.17362800 0.72213400 0.84885400<br>N 3.20852800 -0.03429600 0.85869500<br>O 2.55432900 -0.20287400 1.87617400	• Electronic Energy<br>= -857.752569<br>• Zero Point Energy Correction<br>= 0.230727<br>• Thermal Correction to Energy<br>= 0.247928<br>• Thermal Correction to Enthalpy<br>= 0.248872<br>• Thermal Correction to Free Energy<br>= 0.183538

Name	Anionic HPns RAF C-2a ···NOO$^{\bullet}$ Reactant Complex
Cartesian Coordinate:	Thermochemical Values:
O -3.56823500 -0.86900700 0.39032000<br>O -0.67073400 2.67513400 -0.58808500<br>O -3.07383500 1.70533600 -0.02504600<br>C 0.03892300 -0.93084500 -0.36112500<br>C -2.30464500 -0.53021100 0.08693100<br>C -0.77074300 1.34245800 -0.44273400<br>C 0.26479800 0.45327200 -0.56221900<br>C -1.25064100 -1.40379700 -0.02574100<br>C -2.12564500 0.90154600 -0.12235100<br>C 1.10740000 -1.90733200 -0.47655700<br>C -3.84338800 -2.25101900 0.62028100<br>C 0.62770000 3.21167600 -0.84466300<br>C 3.42867700 -2.69152300 -0.99027900<br>H 1.26285200 0.80453700 -0.78520300<br>H -1.38710700 -2.46597400 0.13112600<br>H 0.84540000 -2.92566100 -0.19826200<br>H -3.25894700 -2.62034500 1.46559600<br>H -3.62222100 -2.83739600 -0.27402200<br>H -4.90442300 -2.30771100 0.84903200	• Electronic Energy<br>= -857.773936<br>• Zero Point Energy Correction<br>= 0.229298<br>• Thermal Correction to Energy<br>= 0.248161<br>• Thermal Correction to Enthalpy<br>= 0.249105<br>• Thermal Correction to Free Energy<br>= 0.179102

H 1.01746300 2.83348000 -1.79242300<br>H 1.31069100 2.95737200 -0.03084700<br>H 0.49674800 4.28928600 -0.90114300<br>H 3.80157300 -2.76920000 -2.01606700<br>H 4.28242100 -2.40153900 -0.36940900<br>H 3.07189800 -3.67012000 -0.66664900<br>C 2.35848000 -1.65815600 -0.90221400<br>H 2.63860800 -0.65365100 -1.20721700<br>O 3.49792400 1.22406900 0.61194500<br>N 3.20259600 0.33570900 1.43318500<br>O 2.17005200 0.53953700 2.09676900	

Name	HPs (4-propylsyringol)
Cartesian Coordinate:	Thermochemical Values:
O -2.07370400 -1.99362800 0.17122200<br>O -1.24964700 2.57750200 0.08729400<br>O -2.87716100 0.53157400 0.47834200<br>C 2.50460500 -0.48779300 -1.00770400<br>C 1.06304500 -0.21514600 -0.65064600<br>C 3.32243200 -0.99891500 0.18784800<br>C 0.61003900 1.09282700 -0.48292700<br>C 0.19100500 -1.28482500 -0.43740700<br>C -1.12557300 -1.03744500 -0.06027200<br>C -0.71100200 1.33750200 -0.10298600<br>C -1.58161200 0.27078300 0.10917300<br>C 3.37512400 -0.00064900 1.34013300<br>C -1.67403600 -3.35535500 0.04151000<br>C -0.38684700 3.69534800 -0.09923200<br>H 2.54566000 -1.22868000 -1.81118500<br>H 2.96091700 0.43149600 -1.38622900<br>H 2.89525400 -1.94510100 0.53566500<br>H 4.33721100 -1.21823000 -0.15648700<br>H 1.29494300 1.91518900 -0.64574600<br>H 0.54450100 -2.30026300 -0.56814300<br>H 3.76991000 0.96208300 1.00138800<br>H 2.38064800 0.17570700 1.75829400<br>H 4.01712000 -0.36337800 2.14591600<br>H -3.33549400 -0.31303300 0.58973800<br>H -0.87221300 -3.58990500 0.74519800<br>H -1.34788400 -3.56538400 -0.97961700<br>H -2.55473300 -3.94879500 0.27550000<br>H -0.01635100 3.73342000 -1.12624900<br>H 0.45371100 3.65755100 0.59779700<br>H -0.99218200 4.57574600 0.10434800	• Electronic Energy = -654.371190<br>• Zero Point Energy Correction = 0.255725<br>• Thermal Correction to Energy = 0.270721<br>• Thermal Correction to Enthalpy = 0.271665<br>• Thermal Correction to Free Energy = 0.213305

Name	HPs 1-OH radical
Cartesian Coordinate:	Thermochemical Values:
O -1.94283900 -2.14075900 0.19222100<br>O -1.41223200 2.50748500 0.10671400<br>O -2.87434600 0.32666900 0.49245700<br>C 2.46474600 -0.32571600 -1.02240800<br>C 1.02208000 -0.13628800 -0.65066700<br>C 3.30474700 -0.81876800 0.16961600<br>C 0.50294200 1.15722600 -0.48143000<br>C 0.22479300 -1.27619600 -0.42719300<br>C -1.08812100 -1.13468800 -0.04209300<br>C -0.80955900 1.32794500 -0.09141500<br>C -1.68212400 0.18459400 0.14693900<br>C 3.29886500 0.15921500 1.33986000<br>C -1.45408500 -3.47354200 0.03350900<br>C -0.63575200 3.69020600 -0.09232200<br>H 2.53148600 -1.05846800 -1.83175700<br>H 2.87424800 0.61949000 -1.38688600<br>H 2.92874700 -1.79353900 0.49485600<br>H 4.32832600 -0.97366100 -0.18130500<br>H 1.14876600 2.00764700 -0.65813900<br>H 0.66300800 -2.25625700 -0.56796500<br>H 3.63827100 1.14896300 1.02031900<br>H 2.29585800 0.26958800 1.76085700<br>H 3.96039900 -0.18305100 2.13845700<br>H -0.63072400 -3.66161900 0.72548900<br>H -1.12635700 -3.63837500 -0.99494500<br>H -2.29214300 -4.12522800 0.26624900<br>H -0.28768000 3.74681500 -1.12550700<br>H 0.21446100 3.70546700 0.59263000	• Electronic Energy = -653.739532<br>• Zero Point Energy Correction = 0.243961<br>• Thermal Correction to Energy = 0.258349<br>• Thermal Correction to Enthalpy = 0.259293<br>• Thermal Correction to Free Energy = 0.201722

H -1.30251100 4.52114100 0.12213600	

Name	HPs 1a-CH radical
Cartesian Coordinate:	Thermochemical Values:
O 2.93105400 -0.94313000 0.11945800<br>O 0.06439800 2.70863900 -0.00679300<br>O 2.48438700 1.68149800 0.18952700<br>C -1.85748900 -1.79459800 -0.39427300<br>C -0.76829900 -0.90525600 -0.24772400<br>C -3.28774600 -1.36993600 -0.47424900<br>C -0.95145900 0.50236700 -0.20674100<br>C 0.55001100 -1.42365000 -0.13721500<br>C 1.62321400 -0.56558300 0.00516800<br>C 0.13693800 1.34717600 -0.06177100<br>C 1.43115700 0.82272600 0.04502500<br>C -3.88408300 -1.06844600 0.91135100<br>C 3.21202900 -2.33983900 0.09486100<br>C -1.22952600 3.29531100 -0.10738100<br>H -1.63470600 -2.85670100 -0.38842700<br>H -3.38923000 -0.48133600 -1.10562800<br>H -3.87140000 -2.16288400 -0.94720400<br>H -1.94794700 0.91412300 -0.28454600<br>H 0.69635300 -2.49564500 -0.16781800<br>H -3.83215400 -1.95145000 1.55253500<br>H -3.33498000 -0.26107900 1.40175400<br>H -4.93123300 -0.76894100 0.82530800<br>H 3.29659600 1.15932200 0.25472300<br>H 2.89498000 -2.77951700 -0.85347500<br>H 2.71579100 -2.84658800 0.92553100<br>H 4.29058700 -2.42977900 0.20029400<br>H -1.87047500 2.96495000 0.71362900<br>H -1.69484400 3.04803000 -1.06449000<br>H -1.07452800 4.36980100 -0.04201800	• Electronic Energy = -653.722812<br>• Zero Point Energy Correction = 0.242068<br>• Thermal Correction to Energy = 0.257001<br>• Thermal Correction to Enthalpy = 0.257945<br>• Thermal Correction to Free Energy = 0.199714

Name	HPs 1a-CH radical anion
Cartesian Coordinate:	Thermochemical Values:
O 0.01566100 -2.73090500 -0.00468600<br>O -3.00310800 0.86105000 0.13152100<br>O -2.47301200 -1.76224300 0.18637100<br>C 1.75447200 1.84129000 -0.38729600<br>C 0.70901000 0.92374100 -0.23925100<br>C 3.20292600 1.46571600 -0.47865300<br>C -0.64362200 1.37779100 -0.12404800<br>C 0.92787000 -0.48865700 -0.19786500<br>C -0.12821800 -1.36304800 -0.05608700<br>C -1.68290100 0.48815900 0.01585700<br>C -1.48960600 -0.93262300 0.05662100<br>C 3.83256600 1.16147500 0.89090500<br>C 1.33250800 -3.25061900 -0.10511200<br>C -3.28765300 2.25100300 0.09173400<br>H 1.49985500 2.89731900 -0.38436100<br>H 3.33280800 0.59110400 -1.12612100<br>H 3.75830400 2.28253500 -0.94701200<br>H -0.82622300 2.44515700 -0.15247700<br>H 1.93907000 -0.86554500 -0.27472300<br>H 3.76525900 2.03316100 1.54662700<br>H 3.31366200 0.33265900 1.37919200	• Electronic Energy = -653.252928<br>• Zero Point Energy Correction = 0.229688<br>• Thermal Correction to Energy = 0.244053<br>• Thermal Correction to Enthalpy = 0.244998<br>• Thermal Correction to Free Energy = 0.187821

H 4.88722200 0.89179700 0.78903300<br>H 1.95995000 -2.89451200 0.71684600<br>H 1.79188100 -2.97811900 -1.05929200<br>H 1.23559300 -4.33299300 -0.04501400<br>H -2.97314400 2.68975600 -0.85928200<br>H -2.79736000 2.77676900 0.91566500<br>H -4.36730700 2.34233300 0.19445200	

Name	HPs 2'-CH_3 radical
Cartesian Coordinate:	Thermochemical Values:
O -1.90124300 -2.23028100 -0.02653200<br>O -1.48363200 2.40866500 0.13430100<br>O -2.93474900 0.21937900 0.40728600<br>C 2.53843900 -0.26925000 -1.02720900<br>C 1.07455900 -0.14113900 -0.68175500<br>C 3.38323500 -0.74554000 0.16400600<br>C 0.50234400 1.11462500 -0.46405500<br>C 0.29473700 -1.28656000 -0.53523500<br>C -1.04033700 -1.16205200 -0.16829300<br>C -0.83866900 1.23089800 -0.10186800<br>C -1.62070300 0.08306500 0.04948600<br>C 3.33685100 0.21683600 1.34663900<br>C -1.36964600 -3.44977000 0.29780500<br>C -0.71649100 3.60362600 0.01817900<br>H 2.65489100 -0.97637500 -1.85320300<br>H 2.91387900 0.69950400 -1.36930900<br>H 3.03387800 -1.73507600 0.47636800<br>H 4.41691300 -0.86672900 -0.17235200<br>H 1.11464500 2.00010700 -0.57836500<br>H 0.71566100 -2.26882500 -0.71906200<br>H 3.65635400 1.21868500 1.04402900<br>H 2.32539500 0.29822400 1.75324800<br>H 3.99473400 -0.11774000 2.15176300<br>H -3.31913200 -0.66206200 0.51317100<br>H -0.47849900 -3.45688500 0.91361500<br>H -2.11807600 -4.22607000 0.35186800<br>H -0.33547900 3.72269400 -0.99875300<br>H 0.11381900 3.60171100 0.72839800<br>H -1.39791100 4.41830900 0.25195100	• Electronic Energy = -653.703418<br>• Zero Point Energy Correction = 0.241423<br>• Thermal Correction to Energy = 0.256386<br>• Thermal Correction to Enthalpy = 0.257330<br>• Thermal Correction to Free Energy = 0.198585

Name	HPs 2'-CH_3 radical anion
Cartesian Coordinate:	Thermochemical Values:
O -1.97814800 -2.22944800 -0.01447900<br>O -1.45333400 2.44870400 0.14454400<br>O -2.98593500 0.25229700 0.39710000<br>C 2.48955000 -0.34696200 -1.03282700<br>C 1.03089100 -0.18293300 -0.67680700<br>C 3.35392300 -0.79837900 0.15422200<br>C 0.48041000 1.08447300 -0.45000400<br>C 0.21033900 -1.29726800 -0.52268100<br>C -1.12131300 -1.13988100 -0.14918500<br>C -0.85457300 1.22899700 -0.08825600<br>C -1.73065200 0.11684800 0.07409800<br>C 3.35293000 0.20406300 1.30406000<br>C -1.43918100 -3.42946100 0.34087900<br>C -0.63899600 3.60446600 0.02153400<br>H 2.58947600 -1.07876400 -1.84029900<br>H 2.87699800 0.60467900 -1.41086100<br>H 2.99253200 -1.76943600 0.50908400<br>H 4.37862400 -0.95233800 -0.19784700<br>H 1.11482600 1.95578700 -0.56339000<br>H 0.60109300 -2.29287700 -0.70889400<br>H 3.68917200 1.18736100 0.96081000	• Electronic Energy = -653.231223<br>• Zero Point Energy Correction = 0.228891<br>• Thermal Correction to Energy = 0.243336<br>• Thermal Correction to Enthalpy = 0.244281<br>• Thermal Correction to Free Energy = 0.186297

H 2.35106400 0.32328900 1.72449100<br>H 4.01820100 -0.11688800 2.10889500<br>H -0.55750600 -3.41787000 0.97157700<br>H -2.17675600 -4.21655300 0.40754200<br>H -0.25133800 3.70711100 -0.99585800<br>H 0.19513100 3.57579600 0.72832500<br>H -1.28057000 4.45298700 0.25191200	

Name	HPs HAT 1a-CH···NOO• Transition State
Cartesian Coordinate:	Thermochemical Values:
O -1.39458100 2.54702900 0.87236900<br>O -2.82391800 -1.61435300 -0.58556400<br>O -3.25685200 0.70978600 0.53341800<br>C 1.90605400 -0.51696200 -1.09790700<br>C 0.57385900 -0.14042400 -0.72837900<br>C 2.98816800 0.52431500 -1.31068600<br>C -0.47464000 -1.09095400 -0.89411600<br>C 0.29837400 1.11438700 -0.11848900<br>C -0.97731500 1.40173600 0.29503700<br>C -1.75020100 -0.80508600 -0.47656400<br>C -2.01776600 0.44894700 0.13039400<br>C 2.74516900 1.35903200 -2.57120800<br>C -0.41780700 3.56782800 1.08953100<br>C -2.61425200 -2.89067800 -1.19148500<br>H 1.93773000 -1.35314300 -1.80000000<br>H 3.06520100 1.17771300 -0.43747200<br>H 3.94680100 0.00665400 -1.39345700<br>H -0.23946500 -2.04048000 -1.35686500<br>H 1.09242600 1.83629600 0.01158300<br>H 2.68486500 0.71578400 -3.45263000<br>H 1.80743400 1.91537400 -2.49431700<br>H 3.55592900 2.07431100 -2.72614000<br>H -3.29103500 1.59211200 0.93948900<br>H 0.00820000 3.89069800 0.13750400<br>H 0.36930700 3.20236200 1.75195800<br>H -0.94954000 4.39080400 1.55923100<br>H -1.89608500 -3.47518400 -0.61250600<br>H -2.26143200 -2.77158300 -2.21822100<br>H -3.58345900 -3.38238700 -1.18640500<br>H 2.26271700 -1.10653000 -0.02060200<br>O 2.71178800 -1.65072200 1.15819100<br>N 1.76391600 -1.41350300 1.97918400<br>O 1.96170500 -1.81813100 3.10756200	• Electronic Energy = -859.398113<br>• Zero Point Energy Correction = 0.260706<br>• Thermal Correction to Energy = 0.279389<br>• Thermal Correction to Enthalpy = 0.280333<br>• Thermal Correction to Free Energy = 0.211010

Name	HPs HAT 1a-CH···NOO• Reactant Complex
Cartesian Coordinate:	Thermochemical Values:
O -0.10292900 -2.56271200 0.96052800<br>O -3.50695800 0.23338700 -0.52667200<br>O -2.67252200 -2.10961400 0.37707900<br>C 1.11564200 2.05851000 -0.36735900<br>C 0.10367100 0.95520200 -0.15583100<br>C 1.86379000 2.45223700 0.91216600<br>C -1.24314000 1.15945400 -0.45073000<br>C 0.52106100 -0.28130700 0.34143300<br>C -0.40134000 -1.30762400 0.51084400<br>C -2.17102600 0.13118300 -0.26520500<br>C -1.75014400 -1.10743400 0.21313500<br>C 0.92879400 2.98648900 1.99275200<br>C 1.27409000 -2.86383500 1.17365000<br>C -3.97697900 1.47882500 -1.03340200<br>H 0.60241400 2.93737700 -0.76851600<br>H 2.41789700 1.58823000 1.29264100<br>H 2.60808400 3.21240000 0.65788300	• Electronic Energy = -859.424692<br>• Zero Point Energy Correction = 0.266070<br>• Thermal Correction to Energy = 0.285766<br>• Thermal Correction to Enthalpy = 0.286711<br>• Thermal Correction to Free Energy = 0.214930

H -1.56363600 2.12157800 -0.82909200<br>H 1.56093000 -0.43862900 0.59741600<br>H 0.36558400 3.84940700 1.62513800<br>H 0.20784200 2.22493100 2.30253700<br>H 1.48543500 3.29989000 2.87867700<br>H -2.20874200 -2.89675800 0.69516300<br>H 1.68479900 -2.24720600 1.97629000<br>H 1.84657200 -2.70996400 0.25486500<br>H 1.31047800 -3.91209900 1.46058400<br>H -3.49894000 1.71576000 -1.98683300<br>H -3.79550500 2.28343500 -0.31696200<br>H -5.04739000 1.35482800 -1.18118700<br>H 1.83988600 1.73893000 -1.12491000<br>O 2.39142300 -0.83410800 -2.07879000<br>N 3.40901000 -0.71233600 -1.49107800<br>O 3.73282500 -0.11519700 -0.52275200	

Name	HPs HAT 1a-CH···NOO• Product Complex
Cartesian Coordinate:	Thermochemical Values:
O -0.34318000 2.72106800 -0.47350200<br>O -3.01837300 -1.07594200 -0.49596500<br>O -2.73131700 1.54814800 -0.46920100<br>C 1.82878600 -1.68292800 -0.42031900<br>C 0.69401600 -0.84191200 -0.49390300<br>C 3.23834700 -1.18946400 -0.37667100<br>C -0.60467300 -1.41771700 -0.50530700<br>C 0.81227600 0.57160700 -0.50266500<br>C -0.32658700 1.35627600 -0.49095600<br>C -1.73300300 -0.61708900 -0.50166700<br>C -1.60366400 0.77791800 -0.48941000<br>C 3.77598600 -0.82607800 -1.77147400<br>C 0.91351200 3.38827600 -0.39251000<br>C -3.19958700 -2.48847300 -0.48371200<br>H 1.65565800 -2.75417600 -0.43998900<br>H 3.31359700 -0.31111100 0.27353200<br>H 3.87540100 -1.96251000 0.05934500<br>H -0.69329200 -2.49640700 -0.49618400<br>H 1.78967900 1.03357100 -0.49162400<br>H 3.75026100 -1.69570800 -2.43217000<br>H 3.17121200 -0.03762000 -2.22579100<br>H 4.80833200 -0.47411700 -1.70847100<br>H -2.46371600 2.47777400 -0.43671100<br>H 1.52504800 3.16624000 -1.26985400<br>H 1.44413600 3.09621200 0.51697500<br>H 0.68602800 4.45120100 -0.36234400<br>H -2.75635000 -2.92829200 0.41294200<br>H -2.76189700 -2.94364800 -1.37531100<br>H -4.27492300 -2.65119600 -0.47912200<br>H 2.08392000 -1.01517100 2.54891800<br>O 1.57094600 -0.18951400 2.57296200<br>N 0.26295600 -0.57541600 2.50981100<br>O -0.44885500 0.35753800 2.47930000	• Electronic Energy = -859.423876<br>• Zero Point Energy Correction = 0.264519<br>• Thermal Correction to Energy = 0.284186<br>• Thermal Correction to Enthalpy = 0.285131<br>• Thermal Correction to Free Energy = 0.214759

Name	Anionic HPs HAT 1a-CH···NOO• Transition State
Cartesian Coordinate:	Thermochemical Values:
O 1.29453900 2.69367600 -0.65030200<br>O 3.07472700 -1.58528700 0.05879300<br>O 3.29633000 0.94339400 -0.75723000<br>C -1.61748900 -0.81726800 1.16821200<br>C -0.35514000 -0.32724800 0.73293300<br>C -2.73569000 0.11674600 1.58456400<br>C 0.75784100 -1.21947900 0.65412300<br>C -0.17628000 1.01344400 0.28142400<br>C 1.03437100 1.43147500 -0.21079400<br>C 1.96468800 -0.80163200 0.16029700<br>C 2.17869700 0.55229300 -0.30377100<br>C -2.45746900 0.83079000 2.91198100<br>C 0.23054300 3.63637500 -0.59387200<br>C 2.95810300 -2.93512700 0.49237400<br>H -1.56530000 -1.75182700 1.73031900<br>H -2.92297100 0.86081900 0.80341300<br>H -3.65869300 -0.46341400 1.67449100<br>H 0.61841000 -2.23698300 0.99800900<br>H -1.00935000 1.70092000 0.33728000<br>H -2.30369400 0.10329800 3.71338400	• Electronic Energy = -858.948996<br>• Zero Point Energy Correction = 0.247988<br>• Thermal Correction to Energy = 0.266335<br>• Thermal Correction to Enthalpy = 0.267279<br>• Thermal Correction to Free Energy = 0.199073

H -1.55623400 1.44555900 2.83975200<br>H -3.29017300 1.47917000 3.19685200<br>H -0.10751300 3.77891400 0.43573000<br>H -0.60781400 3.31354700 -1.21646200<br>H 0.63575000 4.56923700 -0.97942100<br>H 2.20393900 -3.46735000 -0.09284600<br>H 2.70144200 -2.98115800 1.55375600<br>H 3.93400200 -3.38772100 0.33123100<br>H -2.10036500 -1.35383700 -0.07159100<br>O -2.58583000 -1.74917300 -1.08536000<br>N -2.57659700 -0.73167400 -1.89051200<br>O -3.03330500 -0.97382000 -2.97654300	

Name	Anionic HPs HAT 1a-CH···NOO• Reactant Complex
Cartesian Coordinate:	Thermochemical Values:
O -0.04154000 2.66735800 0.59259700<br>O 3.68361400 -0.01396700 -0.32982300<br>O 2.58244800 2.30254000 0.34913900<br>C -0.80950000 -2.04645100 -0.56316500<br>C 0.11581000 -0.88310200 -0.34583100<br>C -1.51355100 -2.47664800 0.73498500<br>C 1.50413500 -1.04645600 -0.47323400<br>C -0.44008500 0.35836900 0.02193100<br>C 0.37731300 1.43953400 0.25236500<br>C 2.34756500 0.02121300 -0.24118700<br>C 1.82719800 1.32974400 0.13667500<br>C -0.52938600 -2.92398900 1.81102000<br>C -1.45193900 2.86763900 0.71105100<br>C 4.29273300 -1.25434900 -0.69306400<br>H -0.24613400 -2.89025000 -0.96921400<br>H -2.12454500 -1.64826500 1.10596400<br>H -2.20043700 -3.29226700 0.49336600<br>H 1.89532300 -2.01599500 -0.75294000<br>H -1.51566400 0.44302700 0.11568000<br>H 0.10590000 -3.73514600 1.44325500<br>H 0.12281000 -2.10110300 2.11707000<br>H -1.05424100 -3.28190300 2.69925000<br>H -1.86236700 2.22971900 1.49696400<br>H -1.94764200 2.65379900 -0.23852500<br>H -1.58078500 3.91439100 0.97411500<br>H 3.95534500 -1.56792500 -1.68289500<br>H 4.05880600 -2.02338100 0.04571400<br>H 5.36273700 -1.06463100 -0.70725400<br>H -1.56626100 -1.75972100 -1.30054800<br>O -3.34459300 0.37978600 -1.76098700<br>N -3.89451100 -0.24149500 -0.83349700<br>O -3.93780600 0.35391900 0.25972500	• Electronic Energy<br>= -858.992883<br>• Zero Point Energy Correction<br>= 0.253181<br>• Thermal Correction to Energy<br>= 0.272314<br>• Thermal Correction to Enthalpy<br>= 0.273258<br>• Thermal Correction to Free Energy<br>= 0.202953

Name	Anionic HPs HAT 1a-CH···NOO• Product Complex
Cartesian Coordinate:	Thermochemical Values:
O -0.75913600 2.82369100 0.43367200<br>O -3.20040800 -1.13285400 -0.20907000<br>O -3.03835900 1.43065800 0.53753300<br>C 1.56457800 -1.14571000 -1.26465300<br>C 0.41186600 -0.46999800 -0.84930300<br>C 2.91135200 -0.50033900 -1.42625200<br>C -0.84202600 -1.16043100 -0.77167300<br>C 0.43317600 0.90030800 -0.43666000<br>C -0.70880700 1.51530900 0.02138700<br>C -1.97110200 -0.53020500 -0.31196300<br>C -1.97578500 0.84472800 0.11144200<br>C 3.03635300 0.30419600 -2.72988800<br>C 0.45266900 3.56318800 0.39777000<br>C -3.29081200 -2.49159300 -0.61150500<br>H 1.44371500 -2.16513800 -1.62363200<br>H 3.12344900 0.16291200 -0.57980200<br>H 3.68307900 -1.27459500 -1.41241700<br>H -0.86992000 -2.19889300 -1.07852900<br>H 1.36454300 1.44804000 -0.48775300<br>H 2.87241800 -0.34177500 -3.59597800<br>H 2.29332900 1.10512100 -2.76188500<br>H 4.02849000 0.75385800 -2.82225300<br>H 0.83770600 3.63654900 -0.62303400	• Electronic Energy<br>= -858.954122<br>• Zero Point Energy Correction<br>= 0.251515<br>• Thermal Correction to Energy<br>= 0.270756<br>• Thermal Correction to Enthalpy<br>= 0.271700<br>• Thermal Correction to Free Energy<br>= 0.200386

H 1.20910500 3.10684100 1.04225400<br>H 0.21064600 4.55761000 0.76755200<br>H -2.64186400 -3.12557900 -0.00113300<br>H -3.02686400 -2.60631300 -1.66635000<br>H -4.32869300 -2.78255500 -0.46216100<br>H 1.64911400 -1.72823700 0.65104500<br>O 1.74826100 -1.95783200 1.61171700<br>N 1.94201600 -0.78013600 2.23801300<br>O 2.05325000 -0.92161300 3.40311900	

Name	HPs RAF C-4···NO$^{\bullet}$ Product
Cartesian Coordinate:	Thermochemical Values:
O -2.93114300 -0.27018800 0.01218900<br>O 0.50258500 2.84908700 0.09012000<br>O -2.05231600 2.22232300 0.12447400<br>C 1.64845500 -1.89461000 0.23105700<br>C 1.29984600 -2.12243300 1.70478400<br>C 1.15294300 0.54331000 -0.24494300<br>C -0.69836300 -1.15300800 -0.30061700<br>C -1.58546800 -0.14108300 -0.13003900<br>C 0.22496500 1.53142600 -0.09098200<br>C -1.15917200 1.22108300 -0.05313500<br>C 1.34289100 -0.87157600 2.57993900<br>C -3.46791600 -1.58983900 -0.04329400<br>C 1.87769100 3.22456400 0.08235000<br>H 1.56485400 -2.84795000 -0.30058100<br>H 2.68856900 -1.56764600 0.14217000<br>H 0.30808300 -2.57934500 1.77562500<br>H 2.00756600 -2.85958400 2.09491200<br>H 2.21377600 0.75716100 -0.25303300<br>H -1.01895000 -2.18587900 -0.34841300<br>H 2.29131100 -0.33905200 2.46093300<br>H 0.53648800 -0.17817600 2.32891600<br>H 1.23697200 -1.13992700 3.63376100<br>H -2.94279300 1.84152100 0.16562700<br>H -3.05754100 -2.20190900 0.76292400<br>H -3.25029900 -2.04940800 -1.00995500<br>H -4.54221400 -1.48138500 0.08332100<br>H 2.33949700 2.97477700 -0.87571400<br>H 2.41570900 2.73123000 0.89519200<br>H 1.89310000 4.30163400 0.23050700<br>C 0.75520100 -0.87688800 -0.48162200<br>N 0.99445800 -0.94524000 -2.03556200<br>O 1.98190200 -1.52435400 -2.35906400	• Electronic Energy<br>= -784.202827<br>• Zero Point Energy Correction<br>= 0.262321<br>• Thermal Correction to Energy<br>= 0.279430<br>• Thermal Correction to Enthalpy<br>= 0.280374<br>• Thermal Correction to Free Energy<br>= 0.217087

Name	Anionic HPs RAF C-4···NO$^{\bullet}$ Product
Cartesian Coordinate:	Thermochemical Values:
O -2.82055900 0.97737700 -0.15710700<br>O 1.67822000 2.34097000 0.05903200<br>O -0.92364500 2.78446800 0.12658700<br>C 0.53785900 -2.30919500 0.45597100<br>C 0.02081400 -2.15145400 1.88811200<br>C 1.24759100 -0.01144000 -0.28993600<br>C -1.13932300 -0.72791500 -0.45585700<br>C -1.53799200 0.53170500 -0.23544300<br>C 0.86430100 1.26035600 -0.11034500<br>C -0.56146700 1.62844800 -0.05269300<br>C 0.61923700 -0.98535600 2.67338200<br>C -3.83861500 -0.00344500 -0.32062500<br>C 3.07654400 2.08086500 0.03529500<br>H 0.05694700 -3.18437400 0.00536600<br>H 1.61275300 -2.50813800 0.47168200<br>H -1.07036300 -2.05999600 1.87806400<br>H 0.24414500 -3.08507600 2.41376900<br>H 2.29714300 -0.27984200 -0.33243700<br>H -1.86164800 -1.52344800 -0.60778100<br>H 1.70980800 -0.96764000 2.58022000<br>H 0.23913200 -0.02376200 2.32314400<br>H 0.37371000 -1.07245400 3.73455400<br>H -3.75712600 -0.77461600 0.45018900<br>H -3.77547500 -0.46328800 -1.31022400<br>H -4.78443500 0.52325600 -0.21772100<br>H 3.37476900 1.66010100 -0.92868000<br>H 3.35395500 1.39296900 0.83866100<br>H 3.56538800 3.04062700 0.18601300	• Electronic Energy<br>= -783.759072<br>• Zero Point Energy Correction<br>= 0.251167<br>• Thermal Correction to Energy<br>= 0.267650<br>• Thermal Correction to Enthalpy<br>= 0.268595<br>• Thermal Correction to Free Energy<br>= 0.206821

C 0.29116700 -1.13168000 -0.51747200<br>N 0.52790200 -1.62911900 -1.94757800<br>O 1.72164400 -2.14729400 -2.06277600	

Name	HPs RAF C-1···NOO• (Model 1) Product
Cartesian Coordinate:	Thermochemical Values:
O 1.17026400 -2.28147500 -0.76250000<br>O 0.96061300 2.38819000 -0.70739000<br>O 1.62157900 0.11514400 -1.94498100<br>C -2.75589800 -0.15456000 1.37343400<br>C -1.41845100 -0.07427700 0.69611300<br>C -3.87636700 -0.59203800 0.41307100<br>C -0.85840100 1.17527000 0.34672700<br>C -0.75168200 -1.27094300 0.32598600<br>C 0.44169700 -1.23021800 -0.32673300<br>C 0.33480200 1.25880000 -0.31358400<br>C -4.06452800 0.37526800 -0.75122100<br>C 0.61520900 -3.58221100 -0.57199600<br>C 0.30219900 3.62382200 -0.43357300<br>H -2.70118600 -0.86861400 2.20115500<br>H -3.00771500 0.82256300 1.79514100<br>H -3.65169500 -1.59361400 0.03279000<br>H -4.80545800 -0.67109500 0.98452500<br>H -1.40144700 2.07700900 0.60212200<br>H -1.21335600 -2.22271000 0.56214900<br>H -4.25641000 1.38876000 -0.38622200<br>H -3.17410500 0.41179500 -1.38468300<br>H -4.90825200 0.07709400 -1.37740700<br>H 1.98180800 -0.75349300 -2.17907400<br>H -0.34847200 -3.65999900 -1.07999900<br>H 0.49620500 -3.79078100 0.49334400<br>H 1.32597300 -4.27813500 -1.01004200<br>H 0.17731800 3.75848300 0.64325000<br>H -0.67029400 3.65466000 -0.92995800<br>H 0.94863000 4.40121600 -0.83278700<br>C 1.14742900 0.05041900 -0.63905400<br>O 2.43271800 0.09586400 0.15641200<br>N 2.19807600 0.06628000 1.48966100<br>O 3.20556100 0.09564100 2.09884100	• Electronic Energy<br>= -859.412746<br>• Zero Point Energy Correction<br>= 0.266469<br>• Thermal Correction to Energy<br>= 0.284736<br>• Thermal Correction to Enthalpy<br>= 0.285680<br>• Thermal Correction to Free Energy<br>= 0.219047

Name	HPs RAF C-2···NOO• (Model 1) Product
Cartesian Coordinate:	Thermochemical Values:
O 1.64633100 -0.95515300 -1.62668400<br>O -0.49260500 2.87960100 -0.38779100<br>O 1.70541200 1.73365400 -1.28350200<br>C -2.20228400 -1.49183700 1.25109500<br>C -1.10147900 -0.66336200 0.63316400<br>C -3.14093400 -2.09979600 0.19735100<br>C -1.31442200 0.74923900 0.47417100<br>C 0.04038500 -1.25076200 0.20420800<br>C -0.36955800 1.53978700 -0.16345700<br>C 0.80248100 0.96781200 -0.64384500<br>C -3.85564500 -1.05617200 -0.65608300<br>C 1.92884200 -2.35928300 -1.67591200<br>C -1.69936100 3.49852800 0.05093100<br>H -1.75719300 -2.29733700 1.83949100<br>H -2.78497900 -0.86520800 1.93285100<br>H -2.56076300 -2.77024000 -0.44477400<br>H -3.87983500 -2.71691100 0.71619600<br>H -2.23323600 1.17992300 0.84829800<br>H 0.21591700 -2.31036100 0.35281900<br>H -4.41016500 -0.35290600 -0.02758300<br>H -3.14941000 -0.48011500 -1.25977700<br>H -4.56543800 -1.53088600 -1.33709600<br>H 2.42843100 1.18218000 -1.62001800<br>H 1.00352300 -2.93309200 -1.74626800<br>H 2.50232800 -2.67186500 -0.80183300<br>H 2.52148900 -2.51030900 -2.57537400<br>H -1.80805500 3.41187200 1.13451100<br>H -2.56477600 3.05538300 -0.44756100<br>H -1.61253800 4.54657200 -0.22595200<br>C 1.13245100 -0.46907200 -0.42518300<br>O 2.36945200 -0.52507700 0.45882600<br>N 2.11978900 -0.05166400 1.69837300	• Electronic Energy<br>= -859.402293<br>• Zero Point Energy Correction<br>= 0.265927<br>• Thermal Correction to Energy<br>= 0.284346<br>• Thermal Correction to Enthalpy<br>= 0.285290<br>• Thermal Correction to Free Energy<br>= 0.218418

O 3.08307500 -0.10371700 2.37600400	

Name	HPs RAF C-3···NOO$^{\bullet}$ (Model 1) Product
Cartesian Coordinate:	Thermochemical Values:
O 0.04392100 2.22735500 -1.51391600<br>O -3.14769100 -0.70015200 0.22060500<br>O -2.52453000 1.55112000 -0.98671900<br>C 1.61704100 -2.03971600 0.58324700<br>C 0.51909300 -1.07946500 0.21658700<br>C 2.64997100 -2.26258600 -0.53090400<br>C -0.80350600 -1.38584900 0.39080400<br>C -0.19301600 1.11541600 -0.74688700<br>C -1.82678000 -0.47755800 0.05499000<br>C -1.49257700 0.76695800 -0.55374500<br>C 2.01249300 -2.72202500 -1.83811500<br>C 1.07541600 3.11686300 -1.06820600<br>C -3.54462700 -1.93474400 0.81865700<br>H 2.13743800 -1.66978200 1.47548500<br>H 1.16007500 -2.99581500 0.85286500<br>H 3.22269100 -1.34460300 -0.69578700<br>H 3.36554700 -3.01156700 -0.18200600<br>H -1.06317500 -2.35928100 0.78904300<br>H 1.41655600 -3.62596400 -1.68079000<br>H 1.34880900 -1.95440200 -2.24708900<br>H 2.77147600 -2.94383600 -2.59130300<br>H -2.14525000 2.35070600 -1.37996300<br>H 2.05295900 2.63323200 -1.11191400<br>H 0.87088900 3.45634300 -0.05025700<br>H 1.05618300 3.96192000 -1.75325000<br>H -3.11743400 -2.03021600 1.81891400<br>H -3.23897100 -2.77811900 0.19623200<br>H -4.62919800 -1.89652300 0.88132800<br>C 0.92851600 0.27412800 -0.26593700<br>O 1.69897300 0.96862400 0.83625000<br>H 1.71868700 0.22654000 -1.02132900<br>N 0.93553400 1.13788700 1.93351000<br>O 1.53830800 1.67650600 2.79406800	• Electronic Energy = -859.403488<br>• Zero Point Energy Correction = 0.266753<br>• Thermal Correction to Energy = 0.285105<br>• Thermal Correction to Enthalpy = 0.286049<br>• Thermal Correction to Free Energy = 0.219082

Name	HPs RAF C-4···NOO$^{\bullet}$ (Model 1) Product
Cartesian Coordinate:	Thermochemical Values:
O 2.43051500 1.63004600 0.03619600<br>O -2.19672200 1.86978000 0.39001900<br>O 0.18818100 2.97364400 0.43977400<br>C -0.18483300 -2.21110800 -1.26138200<br>C 0.08711100 -1.64616600 -2.65724000<br>C -1.25771600 -0.32969100 0.03955000<br>C 1.24612800 -0.47314200 -0.14740300<br>C 1.30371600 0.86851200 0.03750500<br>C -1.15449100 1.00980100 0.23265000<br>C 0.12204000 1.64102700 0.24881000<br>C -0.81224100 -0.48791400 -3.08544900<br>C 3.66871200 0.95345100 -0.16910900<br>C -3.50283900 1.30089200 0.36193800<br>H 0.53091400 -3.01466000 -1.06420300<br>H -1.18946600 -2.64314800 -1.21495400<br>H 1.13429400 -1.33528800 -2.72142600<br>H -0.03246800 -2.47341200 -3.36221400<br>H -2.21826000 -0.82596400 -0.00566400<br>H 2.13456800 -1.06613300 -0.32401700<br>H -1.86806000 -0.72733900 -2.92745300<br>H -0.58853300 0.42424800 -2.52759100<br>H -0.67040300 -0.27248200 -4.14707200<br>H 1.11766600 3.24874600 0.41123400<br>H 3.67737700 0.46402800 -1.14559200<br>H 3.83561000 0.21716400 0.62017200<br>H 4.43871300 1.71998900 -0.13102100<br>H -3.62280700 0.57225200 1.16721300<br>H -3.69347000 0.82358100 -0.60253400<br>H -4.19264800 2.12886500 0.50675200<br>C -0.05484200 -1.19746200 -0.11419700<br>O -0.03231600 -2.15059500 1.08054500<br>N 0.08019900 -1.49406300 2.24278200	• Electronic Energy = -859.407782<br>• Zero Point Energy Correction = 0.266349<br>• Thermal Correction to Energy = 0.284568<br>• Thermal Correction to Enthalpy = 0.285512<br>• Thermal Correction to Free Energy = 0.219698

O 0.09548200 -2.24177200 3.16032400	

Name	HPs RAF C-1···NOO$^{\bullet}$ (Model 2) Product
Cartesian Coordinate:	Thermochemical Values:
O 1.24082800 -2.25672300 -0.51202900<br>O 0.96699000 2.39681000 -0.50228700<br>O 1.80000100 0.13995100 -1.65160800<br>C -2.91446700 -0.18214200 1.19987600<br>C -1.51857600 -0.08449300 0.65668600<br>C -3.92519100 -0.65123900 0.13800700<br>C -0.94712500 1.17132100 0.35317800<br>C -0.79895100 -1.27138000 0.37169800<br>C 0.44783500 -1.21786200 -0.17410500<br>C 0.30460200 1.26685300 -0.18511500<br>C -4.01626000 0.30164100 -1.04946500<br>C 0.70699400 -3.56989300 -0.33518100<br>C 0.29183200 3.63292500 -0.26708700<br>H -2.92786500 -0.88679300 2.03707900<br>H -3.22382400 0.79431500 1.58247500<br>H -3.64410600 -1.65132300 -0.20683300<br>H -4.90402600 -0.74422400 0.61653600<br>H -1.52365700 2.06739800 0.54725200<br>H -1.26286100 -2.22916400 0.57634700<br>H -4.26569800 1.31367100 -0.71672900<br>H -3.06796800 0.35247800 -1.59133700<br>H -4.78629900 -0.02121000 -1.75343900<br>H 2.16558900 -0.73249100 -1.86342500<br>H -0.20051400 -3.69039200 -0.93050400<br>H 0.49263300 -3.75035400 0.72012700<br>H 1.47662900 -4.25385800 -0.68265700<br>H 0.06373300 3.74320300 0.79531000<br>H -0.62680700 3.68039000 -0.85554700<br>H 0.97900700 4.41262200 -0.58484500<br>C 1.13919800 0.06962400 -0.44669300<br>N 2.35786000 0.12282800 0.68654300<br>O 2.03350900 0.37820100 1.82496000<br>O 3.47513000 -0.16526400 0.33266300	• Electronic Energy<br>= -859.417692<br>• Zero Point Energy Correction<br>= 0.267398<br>• Thermal Correction to Energy<br>= 0.285624<br>• Thermal Correction to Enthalpy<br>= 0.286568<br>• Thermal Correction to Free Energy<br>= 0.219344

Name	HPs RAF C-3···NOO$^{\bullet}$ (Model 2) Product
Cartesian Coordinate:	Thermochemical Values:
O -0.02193800 2.34256700 -1.26438200<br>O -3.17868300 -0.78169800 0.18447100<br>O -2.59717100 1.55220700 -0.86757200<br>C 1.60428700 -2.03982700 0.51916500<br>C 0.49402300 -1.06766200 0.23010800<br>C 2.67424600 -2.11112500 -0.57914900<br>C -0.82580000 -1.41437200 0.36014700<br>C -0.26292100 1.16915700 -0.59638600<br>C -1.86575300 -0.51500400 0.06505200<br>C -1.55474400 0.77569500 -0.46120300<br>C 2.08473000 -2.41779300 -1.95210300<br>C 0.87699600 3.25932800 -0.62683400<br>C -3.55709400 -2.06082000 0.69763500<br>H 2.08867400 -1.77783000 1.46861900<br>H 1.15668100 -3.02777900 0.65588800<br>H 3.23673900 -1.17308700 -0.61246600<br>H 3.39008800 -2.88828500 -0.29964800<br>H -1.06655100 -2.42480900 0.66711400<br>H 1.49749600 -3.34041600 -1.92335100<br>H 1.42347800 -1.61418600 -2.28996000<br>H 2.87079600 -2.54003400 -2.70015100<br>H -2.23806100 2.39310900 -1.18756200<br>H 1.88134800 2.83364300 -0.55244600<br>H 0.50219000 3.53302700 0.36175000<br>H 0.91378700 4.13873400 -1.26571100<br>H -3.14352500 -2.20749800 1.69728400<br>H -3.22060100 -2.85520400 0.02879700<br>H -4.64297800 -2.04971700 0.74247300<br>C 0.85894100 0.33717700 -0.12627400<br>H 1.73401500 0.41032100 -0.77708700	• Electronic Energy<br>= -859.412451<br>• Zero Point Energy Correction<br>= 0.268596<br>• Thermal Correction to Energy<br>= 0.286488<br>• Thermal Correction to Enthalpy<br>= 0.287432<br>• Thermal Correction to Free Energy<br>= 0.221921

N 1.45579300 0.93867500 1.23646800<br>O 0.68628300 1.41245800 2.04245000<br>O 2.64455800 0.79081200 1.42381300	

Name	HPs RAF C-4···NOO• (Model 2) Product
Cartesian Coordinate:	Thermochemical Values:
O -2.96965200 -0.14772200 0.02135300<br>O 0.52856000 2.89962000 -0.10361400<br>O -2.03315700 2.32003900 -0.14564800<br>C 1.52536800 -1.78264100 0.79100900<br>C 1.03735500 -1.77460300 2.24251900<br>C 1.14023900 0.56422700 -0.09199100<br>C -0.74688800 -1.09666900 -0.04721100<br>C -1.61743200 -0.05724000 -0.05301700<br>C 0.23263900 1.57536600 -0.12183100<br>C -1.16290200 1.29401100 -0.12338900<br>C 1.04087400 -0.40699100 2.92239600<br>C -3.53200100 -1.45783200 0.07508300<br>C 1.91097200 3.24694400 -0.06809000<br>H 1.45769400 -2.80448400 0.40567300<br>H 2.57358500 -1.47849800 0.74697000<br>H 0.03177100 -2.20244200 2.28955000<br>H 1.68987700 -2.45495500 2.79655900<br>H 2.20476700 0.75237000 -0.05484500<br>H -1.08487600 -2.12245400 0.01915800<br>H 2.00854600 0.08977500 2.80551400<br>H 0.27343800 0.25138600 2.50826600<br>H 0.84433300 -0.51631700 3.99139100<br>H -2.93654700 1.96713400 -0.12871200<br>H -3.19138900 -1.98119000 0.97124800<br>H -3.25936400 -2.02529700 -0.81733200<br>H -4.60939400 -1.31866600 0.11299800<br>H 2.42666100 2.86129500 -0.95057400<br>H 2.38069000 2.85774100 0.83841900<br>H 1.94491800 4.33366200 -0.06475000<br>C 0.71302200 -0.86357100 -0.13873200<br>N 1.17369000 -1.31395900 -1.63016500<br>O 2.36320900 -1.45619000 -1.82398600<br>O 0.33378800 -1.42291600 -2.49545900	• Electronic Energy = -859.414837<br>• Zero Point Energy Correction = 0.268566<br>• Thermal Correction to Energy = 0.286306<br>• Thermal Correction to Enthalpy = 0.287251<br>• Thermal Correction to Free Energy = 0.221933

Name	NO•
Cartesian Coordinate:	Thermochemical Values:
N 0.00000000 0.00000000 -0.60685700<br>O 0.00000000 0.00000000 0.53100000	• Electronic Energy = -129.877717<br>• Zero Point Energy Correction = 0.004739<br>• Thermal Correction to Energy = 0.007100<br>• Thermal Correction to Enthalpy = 0.008044<br>• Thermal Correction to Free Energy = -0.015232

Name	NO^-
Cartesian Coordinate:	Thermochemical Values:
N 0.00000000 0.00000000 -0.66124100<br>O 0.00000000 0.00000000 0.57858600	• Electronic Energy = -129.956799<br>• Zero Point Energy Correction = 0.003517<br>• Thermal Correction to Energy

	= 0.005881<br>• Thermal Correction to Enthalpy = 0.006826<br>• Thermal Correction to Free Energy = -0.015962

Name	HNO
Cartesian Coordinate:	Thermochemical Values:
N 0.05697000 0.72324400 0.00000000<br>O 0.05697000 -0.52002700 0.00000000<br>H -0.85454700 -0.90249400 0.00000000	• Electronic Energy = -130.406762<br>• Zero Point Energy Correction = 0.014590<br>• Thermal Correction to Energy = 0.017434<br>• Thermal Correction to Enthalpy = 0.018378<br>• Thermal Correction to Free Energy = -0.006633

Name	$NOO^{\bullet}$
Cartesian Coordinate:	Thermochemical Values:
N 0.00000000 0.31673800 0.00000000<br>O 1.08981600 -0.13857300 0.00000000<br>O -1.08981600 -0.13857200 0.00000000	• Electronic Energy = -205.046342<br>• Zero Point Energy Correction = 0.009033<br>• Thermal Correction to Energy = 0.011958<br>• Thermal Correction to Enthalpy = 0.012902<br>• Thermal Correction to Free Energy = -0.014940

Name	NOO^{-}
Cartesian Coordinate:	Thermochemical Values:
N 0.00000000 0.00000000 0.46099100<br>O 0.00000000 1.05349200 -0.20168400<br>O 0.00000000 -1.05349200 -0.20168400	• Electronic Energy = -205.246497<br>• Zero Point Energy Correction = 0.008105<br>• Thermal Correction to Energy = 0.011025<br>• Thermal Correction to Enthalpy = 0.011969<br>• Thermal Correction to Free Energy = -0.014875

Name	HNO_2
Cartesian Coordinate:	Thermochemical Values:
O 1.10583200 -0.10951400 0.00000000<br>N 0.00000000 -0.50140600 0.00000000<br>O -0.88499800 0.53275000 0.00000000<br>H -1.76666600 0.12395100 0.00000000	• Electronic Energy = -205.691242<br>• Zero Point Energy Correction = 0.020962<br>• Thermal Correction to Energy = 0.024097<br>• Thermal Correction to Enthalpy = 0.025041<br>• Thermal Correction to Free Energy = -0.002934

8. Table S8: Cartesian Coordinate and Thermochemical Values of all Optimized Stationary Points in Pentyl Ethanoate

Name	Hs (Syringol)
Cartesian Coordinate:	Thermochemical Values:
O 2.32429700 -0.80127400 0.00000600<br>O -2.30867500 -0.74877400 0.00000100<br>O -0.01796800 -2.04366700 0.00000600<br>C 0.03883300 2.08885500 -0.00000400<br>C 1.22296400 -0.01347800 0.00000300<br>C -1.19544700 0.03754800 0.00000000<br>C -1.18608100 1.43022400 -0.00000300<br>C 1.23782600 1.38343700 -0.00000100<br>C -0.00148600 -0.68745800 0.00000300<br>C 3.58656800 -0.15619000 0.00000000<br>C -3.57287500 -0.10209000 -0.00000800<br>H -2.10976200 1.99274800 -0.00000500<br>H 2.17558700 1.92162600 -0.00000100<br>H -0.94300200 -2.32245100 0.00000500<br>H 3.71649900 0.46018500 0.89450600<br>H 3.71649500 0.46017800 -0.89451100<br>H 4.32919400 -0.95170900 0.00000100<br>H -3.69534200 0.51363400 -0.89511700<br>H -3.69535200 0.51364300 0.89509300<br>H -4.31782700 -0.89510000 -0.00000800<br>H 0.06046800 3.17189500 -0.00000600	• Electronic Energy = -536.458159<br>• Zero Point Energy Correction = 0.171508<br>• Thermal Correction to Energy = 0.182095<br>• Thermal Correction to Enthalpy = 0.183039<br>• Thermal Correction to Free Energy = 0.135374

Name	Hs 1-OH radical

Cartesian Coordinate:	Thermochemical Values:
O 2.34345100 -0.78333000 0.00000200<br>O -2.34348200 -0.78330300 -0.00000100<br>O -0.00004100 -2.03932500 0.00000100<br>C 0.00004700 2.02493000 -0.00000300<br>C 1.24544400 -0.02817600 0.00000000<br>C -1.24544200 -0.02815400 -0.00000100<br>C -1.22775800 1.35464600 -0.00000300<br>C 1.22783700 1.35465500 -0.00000200<br>C -0.00005200 -0.80355700 0.00000000<br>C 3.60436300 -0.12503200 0.00000700<br>C -3.60436900 -0.12497100 -0.00000200<br>H -2.14446200 1.92794700 -0.00000300<br>H 2.14452800 1.92798000 -0.00000200<br>H 3.72048300 0.49143600 0.89531300<br>H 3.72049000 0.49143600 -0.89529900<br>H 4.35229100 -0.91448900 0.00000900<br>H -3.72048200 0.49151400 -0.89530100<br>H -3.72048400 0.49151400 0.89529600<br>H -4.35232700 -0.91440000 -0.00000300<br>H 0.00010800 3.10869300 -0.00000400	• Electronic Energy = -535.820165<br>• Zero Point Energy Correction = 0.158915<br>• Thermal Correction to Energy = 0.169139<br>• Thermal Correction to Enthalpy = 0.170083<br>• Thermal Correction to Free Energy = 0.122555

Name	Hs 2’-CH_3 radical
Cartesian Coordinate:	Thermochemical Values:
O 2.25525600 -0.82307800 0.04049100<br>O -2.38626600 -0.67989700 -0.17941400<br>O -0.09483600 -2.03283700 -0.06425200<br>C 0.01477500 2.10336800 -0.05772600<br>C 1.16920300 -0.01921900 -0.00975900<br>C -1.23736000 0.07239000 -0.10721700<br>C -1.21513700 1.46361900 -0.11149100<br>C 1.20218100 1.37477900 -0.00693000<br>C -0.06664100 -0.67889900 -0.06275800<br>C 3.52554900 -0.19750900 0.12104100<br>C -3.54836700 -0.13588600 0.27736700<br>H -2.14054400 2.02192100 -0.17425600<br>H 2.14793700 1.89775300 0.03102700<br>H -1.01856500 -2.31329000 -0.09362500<br>H 3.60835500 0.41217600 1.02557800<br>H 3.71822500 0.42118800 -0.76021400<br>H 4.25422400 -1.00465900 0.16118300<br>H -4.39899100 -0.78569200 0.13508100<br>H -3.48515200 0.55560300 1.10951300<br>H 0.05606300 3.18563600 -0.06405000	• Electronic Energy = -535.790941<br>• Zero Point Energy Correction = 0.157207<br>• Thermal Correction to Energy = 0.167783<br>• Thermal Correction to Enthalpy = 0.168727<br>• Thermal Correction to Free Energy = 0.120722

Name	Hs RAF C-4···NO• Product
Cartesian Coordinate:	Thermochemical Values:
O 2.30885000 -1.07096200 0.07884200<br>O -2.31425200 -1.02555100 0.05864900<br>O -0.02798600 -2.21361200 0.52013600<br>C 1.23697900 -0.26690900 -0.05598800<br>C -1.23329800 -0.21914100 -0.07503500<br>C -1.24739600 1.09159900 -0.43272100<br>C 1.26977200 1.04853700 -0.41674500<br>C -0.01222500 -0.91772300 0.17001400<br>C 3.58614400 -0.48278600 -0.11644300<br>C -3.59636300 -0.43963800 -0.13108000<br>H -2.17036000 1.61967600 -0.62894500<br>H 2.20409700 1.55977300 -0.60288800<br>H -0.95258600 -2.49390800 0.58150600<br>H 3.75363800 0.33039400 0.59549000<br>H 3.69185100 -0.10722700 -1.13818000<br>H 4.31076300 -1.27550400 0.05647600<br>H -3.69894500 -0.06247300 -1.15166900<br>H -3.75703000 0.37035600 0.58491000<br>H -4.31984300 -1.23327100 0.04091900<br>C 0.02380300 1.86231900 -0.44327600<br>H 0.04519500 2.62977800 -1.22144000<br>N 0.06127000 2.65368800 0.89847200<br>O 0.21893400 3.82193100 0.75540500	• Electronic Energy = -666.287210<br>• Zero Point Energy Correction = 0.178261<br>• Thermal Correction to Energy = 0.191267<br>• Thermal Correction to Enthalpy = 0.192211<br>• Thermal Correction to Free Energy = 0.137495

Name	Hs RAF C-1···NOO• (Model 1) Product

Cartesian Coordinate:	Thermochemical Values:
O 2.34015100 0.01152900 0.74711600<br>O -2.32842600 -0.00622600 0.70844600<br>O -0.00208300 0.19363600 2.02158600<br>C 0.03098700 -1.88963900 -1.40116000<br>C 1.26115900 -0.49910200 0.14165100<br>C -1.24072500 -0.50452500 0.09028400<br>C -1.21036400 -1.43630100 -0.90383600<br>C 1.24870200 -1.43155500 -0.85976100<br>C 3.61050900 -0.48328300 0.34567500<br>C -3.59899400 -0.50930200 0.31121100<br>H -2.12888500 -1.84428400 -1.30527300<br>H 2.17883800 -1.83094200 -1.24182800<br>H -0.88822000 0.46291100 2.30047800<br>H 3.79404400 -0.26768400 -0.71047800<br>H 3.67880800 -1.55996600 0.52317100<br>H 4.34274700 0.03705100 0.95862900<br>H -3.65295600 -1.58780500 0.48051200<br>H -3.78635200 -0.28567300 -0.74202900<br>H -4.33311400 -0.00059200 0.93149000<br>H 0.04742900 -2.62081000 -2.19822100<br>C -0.00062000 0.13717200 0.64496800<br>O -0.00431300 1.58279900 0.24940700<br>N 0.01467200 1.72530200 -1.10260300<br>O -0.00019900 2.85824800 -1.41310800	• Electronic Energy = -741.498206<br>• Zero Point Energy Correction = 0.182064<br>• Thermal Correction to Energy = 0.195969<br>• Thermal Correction to Enthalpy = 0.196913<br>• Thermal Correction to Free Energy = 0.140914

Name	Hs RAF C-2···NOO$^{\bullet}$ (Model 1) Product
Cartesian Coordinate:	Thermochemical Values:
O -1.56679300 -1.23280900 -1.00945400<br>O 2.68856000 0.21462700 -0.72510200<br>O 0.37444600 0.43876900 -1.94803400<br>C 0.37642600 -1.00701700 1.91661000<br>C 1.60120800 -0.17230400 -0.00215000<br>C 1.61589400 -0.65014500 1.29768200<br>C -0.81359100 -0.90117700 1.28813000<br>C 0.40521300 -0.04080900 -0.70172200<br>C -2.80881100 -1.78584800 -0.57867000<br>C 3.95935600 0.13496400 -0.09258500<br>H 2.54007500 -0.75106500 1.84845700<br>H -1.74023200 -1.15124500 1.78986400<br>H 1.28383800 0.63798800 -2.21789800<br>H -3.46632400 -1.01592800 -0.16866200<br>H -2.65238000 -2.57853400 0.15685900<br>H -3.26871700 -2.21057700 -1.46919500<br>H 4.19233000 -0.89765100 0.18036500<br>H 3.99081600 0.77292400 0.79455800<br>H 4.68243600 0.49062500 -0.82335500<br>H 0.40509000 -1.37085300 2.93744500<br>C -0.92214300 -0.39451700 -0.11395900<br>O -1.79043700 0.82591700 -0.12130500<br>N -1.23970000 1.82555200 0.61559400<br>O -1.92707000 2.77806800 0.62669400	• Electronic Energy = -741.489904<br>• Zero Point Energy Correction = 0.181864<br>• Thermal Correction to Energy = 0.195883<br>• Thermal Correction to Enthalpy = 0.196827<br>• Thermal Correction to Free Energy = 0.140289

Name	Hs RAF C-3···NOO$^{\bullet}$ (Model 1) Product
Cartesian Coordinate:	Thermochemical Values:
O 1.53755600 1.74254700 -0.22296000<br>O -2.88021100 0.29812400 -0.39229000<br>O -1.01085700 2.03603200 -1.02145400<br>C -0.07684300 -1.21610900 1.43102600<br>C 0.61523700 0.84800400 0.20319700<br>C -1.66204500 0.02973300 0.12485400<br>C -1.35153000 -1.04767700 0.97485900<br>C -0.66831400 0.98462900 -0.23586900<br>C 2.68151600 1.95106700 0.59982200<br>C -3.95274800 -0.58903500 -0.09088700<br>H -2.12815000 -1.73537400 1.28447700<br>H -1.95386400 1.96434000 -1.21695200<br>H 3.31233800 1.05964700 0.64105400<br>H 2.38565200 2.25077300 1.60943400<br>H 3.23829200 2.76146400 0.13367200<br>H -4.15086900 -0.60293200 0.98355400<br>H -3.72790500 -1.59741100 -0.44603900<br>H -4.82076000 -0.19956100 -0.61775900<br>H 0.16904700 -2.03016500 2.10135500	• Electronic Energy = -741.487518<br>• Zero Point Energy Correction = 0.182619<br>• Thermal Correction to Energy = 0.196687<br>• Thermal Correction to Enthalpy = 0.197631<br>• Thermal Correction to Free Energy = 0.141039

C 1.04330300 -0.34069700 0.99586500<br>O 2.05178300 -1.12209400 0.21098100<br>H 1.66806700 -0.05578000 1.84649900<br>N 1.49353300 -1.61665100 -0.92894200<br>O 2.27447500 -2.23185200 -1.55351600	

Name	Hs RAF C-4···NOO• (Model 1) Product
Cartesian Coordinate:	Thermochemical Values:
O 2.26693500 -1.54437500 0.22701500<br>O -2.35909700 -1.35235200 0.19990800<br>O -0.10414400 -2.45422000 0.93502400<br>C 1.21832500 -0.78902800 -0.15728700<br>C -1.25325900 -0.66765000 -0.18564700<br>C -1.22867600 0.48744400 -0.89483700<br>C 1.28780700 0.36793000 -0.86816400<br>C -0.05135500 -1.31856400 0.22822500<br>C 3.56123700 -1.06717300 -0.10662000<br>C -3.62376000 -0.78771200 -0.12284100<br>H -2.13482100 0.97843100 -1.22296200<br>H 2.23543100 0.77968700 -1.18694200<br>H -1.03660700 -2.66944500 1.08521200<br>H 3.74393600 -0.08693200 0.34270100<br>H 3.68798900 -1.00599500 -1.19132700<br>H 4.26251600 -1.79221100 0.30095400<br>H -3.74923400 -0.72047800 -1.20651500<br>H -3.72814000 0.20253300 0.32779600<br>H -4.37031600 -1.46174300 0.29088800<br>C 0.06738600 1.15922600 -1.18749600<br>O 0.12050700 2.48149100 -0.48403500<br>H 0.09599700 1.51472700 -2.21985800<br>N 0.09311100 2.31079700 0.86006700<br>O 0.13520400 3.34183300 1.42553800	• Electronic Energy = -741.494211<br>• Zero Point Energy Correction = 0.182931<br>• Thermal Correction to Energy = 0.196847<br>• Thermal Correction to Enthalpy = 0.197791<br>• Thermal Correction to Free Energy = 0.141332

Name	Hs RAF C-1···NOO• (Model 2) Product
Cartesian Coordinate:	Thermochemical Values:
O 2.33462500 -0.62169400 -0.10397000<br>O -2.32440200 -0.63139000 -0.09949000<br>O 0.02102000 -1.58253400 -1.08600300<br>C 0.00106000 2.22086600 0.08933100<br>C 1.25337300 0.16642300 -0.06560400<br>C -1.24810300 0.17395900 -0.03599700<br>C -1.23102900 1.53380100 0.05877700<br>C 1.22706300 1.53050100 0.01991800<br>C 3.60786800 0.01149100 -0.05864300<br>C -3.60824100 -0.01446400 -0.09485200<br>H -2.15711800 2.09256500 0.09306600<br>H 2.15178900 2.09151500 0.04047400<br>H -0.81576500 -2.06847400 -1.06516100<br>H 3.72451800 0.57304800 0.87201000<br>H 3.73701600 0.67638800 -0.91668500<br>H 4.34229200 -0.78925700 -0.10018100<br>H -3.71248500 0.65391200 -0.95334200<br>H -3.76103100 0.54005300 0.83419100<br>H -4.33137300 -0.82338600 -0.16488200<br>H 0.00387000 3.30018100 0.16010300<br>C 0.00199600 -0.64506100 -0.08646900<br>N -0.00646400 -1.49392800 1.30613400<br>O 0.23804200 -0.87971100 2.31708500<br>O -0.30941900 -2.65871000 1.24326200	• Electronic Energy = -741.503187<br>• Zero Point Energy Correction = 0.183515<br>• Thermal Correction to Energy = 0.197183<br>• Thermal Correction to Enthalpy = 0.198127<br>• Thermal Correction to Free Energy = 0.142587

Name	Hs RAF C-2···NOO• (Model 2) Product
Cartesian Coordinate:	Thermochemical Values:
O 2.03413500 -0.64040500 -0.75281900<br>O -2.46049700 -0.68376500 -0.18837300<br>O -0.19286400 -2.01396600 -0.04929900<br>C -0.02672800 2.09374500 0.20751300<br>C -1.34115900 0.07427000 -0.04450000<br>C -1.29571300 1.45504300 0.05541800<br>C 1.14094100 1.41474500 0.24678700<br>C -0.17357600 -0.67999000 0.00384500<br>C 3.24473600 0.05341900 -1.05519700<br>C -3.71187700 -0.00772600 -0.21845900	• Electronic Energy = -741.493063<br>• Zero Point Energy Correction = 0.183430<br>• Thermal Correction to Energy = 0.197157

H -2.19658500 2.05110500 0.02433800<br>H 2.08531900 1.92673800 0.37940200<br>H -1.11531800 -2.30533300 -0.11278400<br>H 3.80508100 0.30005800 -0.15210300<br>H 3.03765200 0.95656500 -1.63359700<br>H 3.83008000 -0.63697900 -1.65918700<br>H -3.76082000 0.67748900 -1.06867300<br>H -3.87800000 0.53858800 0.71344500<br>H -4.46887700 -0.78088600 -0.32873200<br>H -0.00839500 3.17448300 0.28996400<br>C 1.17366200 -0.07101700 0.14964900<br>N 1.69140000 -0.59202900 1.62437600<br>O 2.79541600 -1.06982800 1.69940700<br>O 0.93151700 -0.42567800 2.54935700	• Thermal Correction to Enthalpy = 0.198101<br>• Thermal Correction to Free Energy = 0.141878

Name	Hs RAF C-3···NOO• (Model 2) Product
Cartesian Coordinate:	Thermochemical Values:
O 2.06035900 -1.20237700 0.05138000<br>O -2.55422500 -0.64569800 0.11216700<br>O -0.42766500 -2.18451600 0.25243200<br>C 0.01823500 1.89451800 -0.51408300<br>C 1.01411600 -0.35967500 -0.10724800<br>C -1.38095200 -0.00502800 -0.05984300<br>C -1.22816100 1.36173700 -0.35368300<br>C -0.24647500 -0.86168600 0.03192100<br>C 3.29547700 -0.84715400 -0.56207800<br>C -3.75808500 0.11279800 0.03197200<br>H -2.09967600 1.98948000 -0.48940100<br>H -1.37751200 -2.35002800 0.31239200<br>H 3.74485600 0.03262800 -0.09213200<br>H 3.16473300 -0.67329300 -1.63395000<br>H 3.95664500 -1.69814100 -0.41282800<br>H -3.87248900 0.54748100 -0.96371800<br>H -3.76615900 0.89822000 0.79123900<br>H -4.56664200 -0.58919500 0.22073700<br>H 0.15136500 2.93701200 -0.77271200<br>C 1.24180200 1.09097600 -0.28030500<br>H 2.03792600 1.33473800 -0.98611900<br>N 1.89531700 1.66598500 1.07486500<br>O 1.54389200 1.19020700 2.12521000<br>O 2.66336600 2.59346800 0.94301600	• Electronic Energy = -741.493960<br>• Zero Point Energy Correction = 0.183874<br>• Thermal Correction to Energy = 0.197767<br>• Thermal Correction to Enthalpy = 0.198711<br>• Thermal Correction to Free Energy = 0.142171

Name	Hs RAF C-4···NOO• (Model 2) Product
Cartesian Coordinate:	Thermochemical Values:
O 2.31496900 -1.07119200 0.06066600<br>O -2.31134700 -1.05095700 0.04763800<br>O -0.01667500 -2.24231800 0.44471100<br>C 1.23805000 -0.27466200 -0.06803300<br>C -1.23487800 -0.24083300 -0.08229200<br>C -1.25705400 1.07897900 -0.39373100<br>C 1.26328500 1.04769200 -0.38912300<br>C -0.00908200 -0.94312500 0.12628400<br>C 3.58936700 -0.46531300 -0.10309500<br>C -3.59833600 -0.45839200 -0.08969900<br>H -2.18365300 1.61031400 -0.56037200<br>H 2.19114100 1.57245100 -0.56754500<br>H -0.93946000 -2.52986200 0.51119800<br>H 3.73854400 0.33198200 0.63038900<br>H 3.70362500 -0.06471200 -1.11432500<br>H 4.31928200 -1.25494800 0.06049800<br>H -3.72625600 -0.05061700 -1.09549400<br>H -3.73814100 0.32872500 0.65529400<br>H -4.31742300 -1.25703300 0.07662400<br>C 0.00718600 1.84381000 -0.44603600<br>H 0.02739200 2.57159000 -1.25895400<br>N 0.08387700 2.82480500 0.80526400<br>O -0.44748500 2.49418100 1.83729800<br>O 0.73123200 3.83739800 0.64733800	• Electronic Energy = -741.501379<br>• Zero Point Energy Correction = 0.184671<br>• Thermal Correction to Energy = 0.198280<br>• Thermal Correction to Enthalpy = 0.199224<br>• Thermal Correction to Free Energy = 0.142782

Name	HAs (4-allylsyringol)

Cartesian Coordinate:	Thermochemical Values:
O 2.84769700 -1.09059500 0.11156700<br>O 0.18658800 2.70079300 0.00630500<br>O 2.54842800 1.54869200 0.19867000<br>C -0.81697300 -0.84022200 -0.27384900<br>C -2.04502300 -1.70951400 -0.46888000<br>C -0.95454800 0.54714300 -0.21846400<br>C 0.44603300 -1.41737200 -0.16412700<br>C 1.58328300 -0.61907000 -0.00541000<br>C 0.17762500 1.33980800 -0.06224800<br>C 1.44818100 0.76862200 0.04379600<br>C -3.11860700 -1.42343800 0.54358200<br>C 3.02749900 -2.49583800 0.06899500<br>C -1.06270700 3.37211800 -0.06248800<br>C -4.34021800 -0.99452800 0.24262400<br>H -2.45022600 -1.55957100 -1.47490000<br>H -1.74388800 -2.75878400 -0.39355600<br>H -1.93838100 0.99353700 -0.29556900<br>H 0.53958900 -2.49480200 -0.20571100<br>H -2.84184600 -1.57322900 1.58580400<br>H 2.25054600 2.46739600 0.21889300<br>H 2.68962100 -2.90995700 -0.88562200<br>H 2.49964700 -2.98817600 0.89122800<br>H 4.09737900 -2.66406400 0.17619200<br>H -1.71369400 3.06828400 0.76176400<br>H -1.55752400 3.17711300 -1.01765700<br>H -0.84005200 4.43385100 0.02141100<br>H -5.07881300 -0.79935800 1.01260600<br>H -4.64133000 -0.82960000 -0.78839800	• Electronic Energy = -653.144244<br>• Zero Point Energy Correction = 0.232857<br>• Thermal Correction to Energy = 0.247249<br>• Thermal Correction to Enthalpy = 0.248193<br>• Thermal Correction to Free Energy = 0.191127

Name	HAs 1-OH radical
Cartesian Coordinate:	Thermochemical Values:
O 2.89894000 -0.91786700 0.14312200<br>O -0.01085700 2.75268400 0.03685700<br>O 2.41940100 1.69525600 0.28807600<br>C -0.73077700 -0.82436800 -0.36123000<br>C -1.91009600 -1.73997300 -0.59539400<br>C -0.95900600 0.56090600 -0.27319900<br>C 0.55728300 -1.35677300 -0.22275900<br>C 1.63581900 -0.51781200 -0.00166100<br>C 0.09246500 1.42577400 -0.05734600<br>C 1.46552100 0.93344600 0.09302600<br>C -2.89554300 -1.67842400 0.54183200<br>C 3.17151800 -2.31159000 0.06964600<br>C -1.30491800 3.32720600 -0.08970500<br>C -4.15856500 -1.28528300 0.41647500<br>H -2.41417700 -1.45421800 -1.52425900<br>H -1.54798500 -2.76476200 -0.71476300<br>H -1.97336900 0.92685700 -0.37418900<br>H 0.69098300 -2.42833200 -0.29516900<br>H -2.51342300 -1.96987600 1.51826700<br>H 2.89111800 -2.71167900 -0.90837000<br>H 2.64357700 -2.85290000 0.85917600<br>H 4.24494800 -2.41309200 0.21212300<br>H -1.97269900 2.95838100 0.69345100<br>H -1.73127700 3.11174400 -1.07312900<br>H -1.16704200 4.40020400 0.02189200<br>H -4.82902300 -1.25616000 1.26846400<br>H -4.56370900 -0.98540000 -0.54604400	• Electronic Energy = -652.508243<br>• Zero Point Energy Correction = 0.220403<br>• Thermal Correction to Energy = 0.234407<br>• Thermal Correction to Enthalpy = 0.235352<br>• Thermal Correction to Free Energy = 0.178201

Name	HAs 1a-CH radical
Cartesian Coordinate:	Thermochemical Values:
O -2.48793300 -1.71611600 0.00034900<br>O -0.89905200 2.63490700 -0.00013900<br>O -2.88167200 0.90862400 0.00018400<br>C 1.02576700 -0.52705500 -0.00012800<br>C 2.34764000 -1.08064100 -0.00022800<br>C 0.77897800 0.86305900 -0.00019900<br>C -0.07409400 -1.41281700 0.00004700<br>C -1.37971400 -0.93947200 0.00015600<br>C -0.52487300 1.32559400 -0.00009800<br>C -1.61310600 0.44168400 0.00007500<br>C 3.56136100 -0.36297900 -0.00036200<br>C -2.29886600 -3.12142400 0.00077500	• Electronic Energy = -652.511563<br>• Zero Point Energy Correction = 0.219345<br>• Thermal Correction to Energy = 0.233597<br>• Thermal Correction to Enthalpy

C 0.12815700 3.61619700 -0.00018000<br>C 4.78747100 -0.95546400 -0.00045600<br>H 2.41559600 -2.16546700 -0.00018900<br>H 1.59948700 1.56555900 -0.00033100<br>H 0.12175200 -2.47707900 0.00009600<br>H 3.52534500 0.72320700 -0.00038900<br>H -2.83740900 1.87428100 0.00013200<br>H -1.75995200 -3.44737400 0.89534700<br>H -1.76016000 -3.44796700 -0.89370400<br>H -3.29588300 -3.55741600 0.00102800<br>H 0.74938600 3.52813300 -0.89546600<br>H 0.74948600 3.52809700 0.89503200<br>H -0.37573800 4.58033900 -0.00013500<br>H 5.69687300 -0.36768900 -0.00055100<br>H 4.88814200 -2.03603300 -0.00043800	= 0.234541<br>• Thermal Correction to Free Energy<br>= 0.177689

Name	HAs 2'-CH_3 radical
Cartesian Coordinate:	Thermochemical Values:
O -2.76277700 -1.20945100 -0.07758200<br>O -0.27884600 2.71687600 -0.18078600<br>O -2.61054300 1.42911200 -0.27784800<br>C 0.89013500 -0.75363700 0.24993900<br>C 2.16101100 -1.55573800 0.45743700<br>C 0.95296100 0.63203300 0.13252600<br>C -0.34700700 -1.39700500 0.18094500<br>C -1.52491100 -0.67032800 0.00159300<br>C -0.22498900 1.35069600 -0.03494200<br>C -1.46570700 0.72352500 -0.10647800<br>C 3.22227900 -1.22858800 -0.55546800<br>C -2.87027400 -2.61873600 0.03722500<br>C 0.73412900 3.46879600 0.33172600<br>C 4.42260600 -0.74372600 -0.25468100<br>H 2.55414500 -1.37282000 1.46275000<br>H 1.91456200 -2.62008200 0.39546600<br>H 1.90713200 1.14630600 0.14949700<br>H -0.38404500 -2.47527300 0.26748900<br>H 2.95525500 -1.39899600 -1.59697300<br>H -2.37987900 2.36561100 -0.32530200<br>H -2.49876300 -2.96594400 1.00582700<br>H -2.32859600 -3.12372000 -0.76799600<br>H -3.93140900 -2.84516700 -0.04465800<br>H 0.62067200 4.52205300 0.12228500<br>H 1.22052400 3.10992800 1.23113100<br>H 5.15377400 -0.52167300 -1.02436800<br>H 4.71255500 -0.55626300 0.77564700	• Electronic Energy<br>= -652.476898<br>• Zero Point Energy Correction<br>= 0.218667<br>• Thermal Correction to Energy<br>= 0.233034<br>• Thermal Correction to Enthalpy<br>= 0.233978<br>• Thermal Correction to Free Energy<br>= 0.176472

Name	HAs HAT 1a-CH···NOO$^\bullet$ Transition States Structure
Cartesian Coordinate:	Thermochemical Values:
O 1.48771200 2.69238800 0.42360700<br>O 2.62774800 -1.64496500 -0.69576800<br>O 3.24811100 0.76859200 0.09314500<br>C -0.71336400 -0.05801700 -0.67386100<br>C -2.10582700 -0.42408100 -0.90152500<br>C 0.26669200 -1.07172600 -0.84316600<br>C -0.32628500 1.22771600 -0.23352000<br>C 1.00015200 1.51260800 0.01770400<br>C 1.58525000 -0.79405400 -0.58632800<br>C 1.97289200 0.49657200 -0.15575600<br>C -3.15660100 0.61591300 -1.01497300<br>C 0.55462200 3.74013000 0.66581500<br>C 2.33940200 -2.99639700 -1.04739600<br>C -4.17171100 0.54156300 -1.87435100<br>H -2.21212900 -1.22073100 -1.64203300<br>H -0.04641300 -2.05709000 -1.16443500<br>H -1.07531300 1.99522200 -0.10581100<br>H -3.11771100 1.44689200 -0.31558200<br>H 3.77019700 -0.03570700 -0.05546800<br>H -0.15145200 3.45521700 1.45028000<br>H 0.01512600 3.99891000 -0.24921500<br>H 1.14493500 4.59173600 0.99537200<br>H 1.89240800 -3.04444400 -2.04325400<br>H 1.67033500 -3.44689400 -0.31038800<br>H 3.29408200 -3.51648200 -1.04678800	• Electronic Energy<br>= -858.165938<br>• Zero Point Energy Correction<br>= 0.238247<br>• Thermal Correction to Energy<br>= 0.256281<br>• Thermal Correction to Enthalpy<br>= 0.257225<br>• Thermal Correction to Free Energy<br>= 0.189801

H -4.94395700 1.30200700 -1.90005500<br>H -4.26031100 -0.28192000 -2.57686000<br>H -2.34453600 -1.00639600 0.12983100<br>O -2.39155900 -1.62315900 1.49941200<br>N -1.29777000 -1.18681000 1.94682500<br>O -0.97478700 -1.51960800 3.06594900	

Name	HAs HAT 1a-CH···NOO• Reactant Complex
Cartesian Coordinate:	Thermochemical Values:
O 0.31931900 2.92850300 0.25252100<br>O 2.89050400 -0.82713800 -0.60990600<br>O 2.66684000 1.69728300 0.11860300<br>C -0.77138800 -0.42066900 -0.85266700<br>C -2.02487700 -1.19109200 -1.22587300<br>C 0.47324400 -1.04864700 -0.92257900<br>C -0.85255700 0.91651200 -0.46357600<br>C 0.30091400 1.63449900 -0.14240700<br>C 1.62190700 -0.33140500 -0.60012100<br>C 1.54720700 1.00691500 -0.21003400<br>C -3.20763300 -0.86861900 -0.35729700<br>C -0.92673600 3.59685700 0.35737900<br>C 3.05792900 -2.19982100 -0.93255000<br>C -4.35135800 -0.36044600 -0.80445100<br>H -2.28221100 -0.98943000 -2.27130600<br>H 0.53512300 -2.08917500 -1.21617400<br>H -1.82355500 1.39135000 -0.40743600<br>H -3.09260700 -1.06944800 0.70692700<br>H 3.41436900 1.08977800 0.04388600<br>H -1.57614900 3.10983600 1.09085900<br>H -1.43472700 3.64215200 -0.61056600<br>H -0.69718400 4.60662300 0.69208300<br>H 2.71192900 -2.40627300 -1.94882700<br>H 2.52206100 -2.83442700 -0.22084800<br>H 4.12554300 -2.39834200 -0.86528000<br>H -5.17915700 -0.15124000 -0.13552200<br>H -4.49676500 -0.14191700 -1.85886300<br>H -1.80486000 -2.26135100 -1.15258700<br>O -0.70217000 -2.49108500 1.80734900<br>N -0.55573200 -1.32119000 1.92922700<br>O 0.29655600 -0.63935200 2.37820000	• Electronic Energy = -858.203671<br>• Zero Point Energy Correction = 0.243239<br>• Thermal Correction to Energy = 0.262340<br>• Thermal Correction to Enthalpy = 0.263284<br>• Thermal Correction to Free Energy = 0.192763

Name	HAs HAT 1a-CH···NOO• Product Complex
Cartesian Coordinate:	Thermochemical Values:
O 0.93874900 2.55035200 -0.87314200<br>O 2.76798100 -1.62319900 -0.06649000<br>O 3.00355900 0.97795400 -0.36872700<br>C -0.77407200 -0.72724700 -0.68573100<br>C -2.06487300 -1.35011400 -0.71847100<br>C 0.35293700 -1.53661700 -0.42262700<br>C -0.59286100 0.66480800 -0.85327600<br>C 0.66773900 1.23327600 -0.74217100<br>C 1.60710000 -0.96147300 -0.32081800<br>C 1.78020600 0.41984000 -0.47497000<br>C -3.30906200 -0.70224900 -0.88407100<br>C -0.15729600 3.42483800 -1.08756600<br>C 2.69949600 -3.02985800 0.12197000<br>C -4.50212800 -1.35836200 -0.89407000<br>H -2.08274200 -2.43057700 -0.59795600<br>H 0.21765400 -2.60242300 -0.28943100<br>H -1.44510700 1.29635800 -1.05961300<br>H -3.32347000 0.37779600 -1.00440800<br>H 3.63238800 0.27137600 -0.16779300<br>H -0.86754000 3.37566700 -0.25652100<br>H -0.66946200 3.19391400 -2.02619300<br>H 0.26524800 4.42588400 -1.14321700<br>H 2.31576200 -3.52307600 -0.77496200<br>H 2.07073400 -3.27574600 0.98184400<br>H 3.71916900 -3.35894100 0.31025400<br>H -5.43604700 -0.82510600 -1.01894400<br>H -4.54977700 -2.43633900 -0.77823800<br>H -1.94552900 -0.40607800 1.46665000<br>O -1.84202100 0.25438000 2.17434700<br>N -0.48173500 0.37349700 2.32684100	• Electronic Energy = -858.209816<br>• Zero Point Energy Correction = 0.241370<br>• Thermal Correction to Energy = 0.260626<br>• Thermal Correction to Enthalpy = 0.261570<br>• Thermal Correction to Free Energy = 0.190785

O -0.21479900 1.19573300 3.11419200	

Name	HAs RAF C-4···NO• Product
Cartesian Coordinate:	Thermochemical Values:
O -1.38332300 2.45911800 0.07796600<br>O 2.86069000 0.64252800 -0.19393600<br>O 1.23772400 2.69917200 -0.11458800<br>C -0.96688600 -2.40567300 0.09233700<br>C 1.07408000 -0.92739400 0.26453900<br>C -1.22060400 0.07860100 0.42690700<br>C -0.68683300 1.31415200 0.21813000<br>C 1.56916500 0.32469600 0.06872600<br>C 0.72648200 1.47343600 0.08663200<br>C -1.10588000 -2.35775100 -1.40036100<br>C -2.79575000 2.37236900 0.18833200<br>C 3.80830600 -0.41810900 -0.21153100<br>C -2.25956200 -2.48083700 -2.04856900<br>H -0.32381800 -3.24401300 0.38115200<br>H -1.94406300 -2.57035400 0.55465200<br>H 1.70943400 -1.80385900 0.24979900<br>H -2.28962100 -0.06976000 0.50580500<br>H -0.18808500 -2.20566500 -1.96535400<br>H 2.19453200 2.60682900 -0.22807600<br>H -3.08785200 1.99521000 1.17257500<br>H -3.20951900 1.72787100 -0.59252500<br>H -3.16930500 3.38609100 0.06026400<br>H 3.57443800 -1.12990500 -1.00729400<br>H 3.82975900 -0.92956600 0.75414300<br>H 4.77408700 0.04306700 -0.40552100<br>H -2.30708700 -2.44010300 -3.13131000<br>H -3.19493100 -2.62828900 -1.51570200<br>C -0.35524900 -1.11558100 0.64667500<br>N -0.25253600 -1.20366300 2.22506900<br>O -0.58762500 -2.24024300 2.69044500	• Electronic Energy = -782.974534<br>• Zero Point Energy Correction =0.238900<br>• Thermal Correction to Energy = 0.255771<br>• Thermal Correction to Enthalpy = 0.256715<br>• Thermal Correction to Free Energy = 0.192840

Name	HAs RAF C-2a···NO• Product
Cartesian Coordinate:	Thermochemical Values:
O -1.76673400 2.43528600 0.12568700<br>O -2.00649500 -2.19296700 0.12821100<br>O -3.09975000 0.17387300 0.52534900<br>C 0.84141500 -0.02206000 -0.76475200<br>C 2.28978300 -0.08544200 -1.17998000<br>C 0.12568700 -1.19897100 -0.54944700<br>C 0.23205600 1.21342600 -0.54655700<br>C -1.09274700 1.28523800 -0.11171200<br>C -1.19584500 -1.12574800 -0.11647000<br>C -1.81148800 0.10710200 0.10506700<br>C -1.06054800 3.65188600 -0.04810900<br>C -1.44736400 -3.49124500 -0.01319100<br>C 3.14743500 1.00308800 0.96769900<br>H 2.47423300 -0.96830100 -1.79699900<br>H 2.55823100 0.79543600 -1.76621700<br>H 0.60072500 -2.15772600 -0.71631100<br>H 0.79956000 2.11911300 -0.71873400<br>H -3.41773900 -0.73249800 0.62905000<br>H -0.73337100 3.77594700 -1.08478100<br>H -0.19537700 3.70542100 0.61983900<br>H -1.76196300 4.44440700 0.20523300<br>H -0.60023400 -3.62557800 0.66498200<br>H -1.13000300 -3.66847200 -1.04433600<br>H -2.23859400 -4.19070600 0.24836700<br>H 2.56734100 0.93032200 1.87616200<br>H 3.58088500 1.95435400 0.69013300<br>C 3.26144300 -0.13871100 0.03622900<br>H 4.27205900 -0.21839100 -0.38588000<br>N 3.04858600 -1.48767600 0.63979400<br>O 2.75780800 -1.49436800 1.79622700	• Electronic Energy = -782.990018<br>• Zero Point Energy Correction = 0.238763<br>• Thermal Correction to Energy = 0.255709<br>• Thermal Correction to Enthalpy = 0.256653<br>• Thermal Correction to Free Energy = 0.192856

Name	HAs RAF C-3a···NO• Product
Cartesian Coordinate:	Thermochemical Values:
O -0.50346500 2.85038800 -0.00996000<br>O -2.84180600 -1.11899500 0.48447800<br>O -2.68424900 1.50358200 0.68916300<br>C 0.58162300 -0.59008200 -0.78669400<br>C 1.79796200 -1.35768700 -1.25017100<br>C -0.56679600 -1.28326000 -0.40655600<br>C 0.63354600 0.79633100 -0.66552200<br>C -0.46198100 1.50700000 -0.16631000<br>C -1.65883000 -0.57362400 0.08599900<br>C -1.61601900 0.81701700 0.21035000<br>C 2.60480600 -1.93059800 -0.12520500<br>C 0.66200100 3.58306600 -0.35183500<br>C -2.97995700 -2.52969100 0.39935200<br>H 1.49942500 -2.17276300 -1.91719600<br>H 2.43449600 -0.69400000 -1.85341900<br>H -0.59923500 -2.36225800 -0.49551700<br>H 1.53850800 1.31682900 -0.95203200<br>H 3.32685700 -2.70442200 -0.36317200<br>H -3.37579600 0.86003700 0.89120600<br>H 0.90237700 3.47164200 -1.41321300<br>H 1.51781900 3.27001000 0.25372500<br>H 0.43197600 4.62571200 -0.14152900<br>H -2.24080900 -3.03182200 1.02937800<br>H -2.87867000 -2.86897600 -0.63504100<br>H -3.98063100 -2.75755900 0.76043500<br>C 2.75118800 -1.24383300 1.17802800<br>H 3.13858500 -1.89348300 1.96435600<br>H 1.83490800 -0.73353400 1.48458600<br>N 3.76178900 -0.14440500 0.92154400<br>O 4.83138200 -0.34502800 1.41312600	• Electronic Energy<br>= -782.993544<br>• Zero Point Energy Correction<br>= 0.239858<br>• Thermal Correction to Energy<br>= 0.256606<br>• Thermal Correction to Enthalpy<br>= 0.257550<br>• Thermal Correction to Free Energy<br>= 0.193159

Name	HAs RAF C-1···NOO• (Model 1) Product
Cartesian Coordinate:	Thermochemical Values:
O -2.05039300 -1.83682400 -0.70987600<br>O -0.19776500 2.44865900 -0.71841700<br>O -1.84061400 0.61126600 -1.77092100<br>C 1.33187600 -0.75708900 0.30130600<br>C 2.63632100 -1.30584200 0.82378200<br>C 1.21610500 0.63540600 0.05125400<br>C 0.24179200 -1.61093600 0.03347900<br>C -0.95431900 -1.12242300 -0.42084000<br>C 0.04540300 1.16273900 -0.39479500<br>C 3.78312700 -1.02093700 -0.11011300<br>C -1.97219100 -3.24714200 -0.55658700<br>C 0.88179900 3.36826300 -0.60497100<br>C 4.84498900 -0.28759800 0.20817300<br>H 2.85736200 -0.86909800 1.80436000<br>H 2.53596100 -2.38619100 0.96232200<br>H 2.08280800 1.26799600 0.20431700<br>H 0.36432700 -2.67493900 0.19425800<br>H 3.69779100 -1.44308600 -1.10977900<br>H -1.82855300 1.56978700 -1.89981400<br>H -1.76713500 -3.51245500 0.48434300<br>H -1.19849700 -3.66495100 -1.20653000<br>H -2.94522600 -3.63528400 -0.84889000<br>H 1.71095600 3.06722100 -1.25055000<br>H 1.22066500 3.43376700 0.43210300<br>H 0.49347900 4.33182800 -0.92632100<br>H 5.64263900 -0.10621500 -0.50405200<br>H 4.95234300 0.14979900 1.19709700<br>C -1.20006900 0.34617900 -0.57997900<br>O -2.20682700 0.78600100 0.44386700<br>N -1.75505700 0.57167000 1.70636900<br>O -2.53721500 0.92919900 2.50763400	• Electronic Energy<br>= -858.185707<br>• Zero Point Energy Correction<br>= 0.243406<br>• Thermal Correction to Energy<br>= 0.261232<br>• Thermal Correction to Enthalpy<br>= 0.262176<br>• Thermal Correction to Free Energy<br>= 0.195623

Name	HAs RAF C-2···NOO• (Model 1) Product
Cartesian Coordinate:	Thermochemical Values:
O 2.78408900 1.57491700 -0.16289900<br>O 0.24362600 -1.99544900 -1.44551300<br>O 2.58361100 -1.02288700 -0.67367700<br>C -0.91824300 1.12232800 -0.12739900	• Electronic Energy<br>= -858.176662

C -2.13895100 2.00428400 0.04555700<br>C -1.02167600 -0.20886500 -0.33567600<br>C 0.36399100 1.76904900 -0.02568400<br>C 1.53678500 1.05261900 -0.19227100<br>C 1.47137200 -0.31876700 -0.44615000<br>C -3.41962600 1.40122200 -0.44906500<br>C 2.89576600 2.97300300 0.05309900<br>C -0.92098000 -2.79270900 -1.66223100<br>C -4.47205500 1.14126000 0.31900000<br>H -2.23347700 2.26152900 1.10626600<br>H -1.95326800 2.94317400 -0.48838100<br>H -1.98533500 -0.70236000 -0.37205400<br>H 0.39021800 2.83469400 0.15892400<br>H -3.46231200 1.17554000 -1.51326600<br>H 2.32672500 -1.91402300 -0.95613700<br>H 2.47985500 3.25592600 1.02433100<br>H 2.39615700 3.53516200 -0.74129500<br>H 3.96112000 3.19317300 0.03820200<br>H -1.71000400 -2.20147100 -2.13158400<br>H -1.27850500 -3.22809700 -0.72719200<br>H -0.61676800 -3.58882700 -2.33898300<br>H -5.38194600 0.71661100 -0.09116300<br>H -4.45727400 1.34867800 1.38536500<br>C 0.18361500 -1.07240000 -0.40813600<br>O 0.22090200 -1.98336400 0.80074000<br>N 0.22353600 -1.26650900 1.95745900<br>O 0.24278100 -1.97200500 2.89616200	• Zero Point Energy Correction = 0.243274<br>• Thermal Correction to Energy = 0.261072<br>• Thermal Correction to Enthalpy = 0.262016<br>• Thermal Correction to Free Energy = 0.196622

Name	HAs RAF C-3···NOO$^{\bullet}$ (Model 1) Product
Cartesian Coordinate:	Thermochemical Values:
O -3.19934700 -0.82418900 -0.04233600<br>O -0.03180900 2.50128400 -0.79150000<br>O -2.56760400 1.65317400 -0.70233700<br>C 0.46710100 -1.13453600 0.04935600<br>C 1.58232200 -2.13157700 0.23626200<br>C -0.85398100 -1.48587300 0.11924400<br>C -1.88681700 -0.55350300 -0.10563200<br>C -0.25309600 1.17835200 -0.53388200<br>C -1.55337600 0.78919700 -0.45535300<br>C 2.34983000 -2.33886600 -1.04464100<br>C -3.59161000 -2.14170200 0.32055200<br>C 1.15637600 2.82950600 -1.50926800<br>C 3.60237200 -1.93788600 -1.23750800<br>H 2.27177100 -1.78217000 1.01286500<br>H 1.15545500 -3.08057700 0.57172800<br>H -1.10432700 -2.51699300 0.33888400<br>H 1.80425100 -2.82151200 -1.85337300<br>H -2.17131400 2.51354800 -0.89508100<br>H -3.21098600 -2.40225500 1.31169300<br>H -3.24353200 -2.87027000 -0.41647700<br>H -4.67914900 -2.13406900 0.33770000<br>H 1.20566700 2.27418900 -2.45016500<br>H 2.04544000 2.62902100 -0.90648800<br>H 1.09798000 3.89577700 -1.71846000<br>H 4.10789300 -2.09097200 -2.18496600<br>H 4.16431400 -1.44792200 -0.44665300<br>C 0.87790300 0.28033200 -0.19514500<br>O 1.62109900 0.82738900 0.98529300<br>H 1.68013500 0.32708400 -0.93932400<br>N 0.82220400 0.84876500 2.08755300<br>O 1.39501500 1.27298000 3.02104700	• Electronic Energy = -858.177667<br>• Zero Point Energy Correction = 0.244340<br>• Thermal Correction to Energy = 0.262147<br>• Thermal Correction to Enthalpy = 0.263091<br>• Thermal Correction to Free Energy = 0.197318

Name	HAs RAF C-4···NOO$^{\bullet}$ (Model 1) Product
Cartesian Coordinate:	Thermochemical Values:
O -2.83652700 0.55127200 -0.79732600<br>O 1.15486700 2.61997200 0.32432000<br>O -1.38523500 2.69963300 -0.29948100<br>C 1.20588100 -2.05919700 -0.78125200<br>C 1.19451700 0.20020300 0.30525600<br>C -0.97748000 -0.90485400 -0.31342300<br>C -1.55798300 0.31803200 -0.43507200<br>C 0.58074400 1.39983100 0.16883500<br>C -0.79935900 1.50080700 -0.18245200<br>C 1.36462400 -1.53763600 -2.17864500<br>C -3.65655000 -0.58297600 -1.03088300	• Electronic Energy = -858.183006<br>• Zero Point Energy Correction = 0.243638<br>• Thermal Correction to Energy = 0.261348

C 2.52918300 2.65446400 0.68733100<br>C 2.53751500 -1.34740100 -2.77314200<br>H 2.18498000 -2.25254400 -0.33464800<br>H 0.64605100 -3.00080800 -0.79065700<br>H 2.24587300 0.11740800 0.55054000<br>H -1.52979000 -1.81498200 -0.51001900<br>H 0.44687400 -1.30746400 -2.71628400<br>H -0.72109300 3.37594300 -0.10002100<br>H -3.72438200 -1.20691900 -0.13505000<br>H -3.27473900 -1.17715500 -1.86633400<br>H -4.64163300 -0.19485300 -1.28096700<br>H 3.14189600 2.18151800 -0.08456000<br>H 2.68526000 2.15503300 1.64701600<br>H 2.79315200 3.70617500 0.77264000<br>H 2.60232200 -0.97466500 -3.78956200<br>H 3.47212200 -1.56331500 -2.26282800<br>C 0.44033600 -1.07495800 0.12390500<br>O 0.45268000 -1.83016100 1.42232900<br>N -0.17667800 -1.13583600 2.40128000<br>O -0.16812500 -1.72701600 3.41878600	• Thermal Correction to Enthalpy = 0.262292<br>• Thermal Correction to Free Energy = 0.196954

Name	HAs RAF C-2a···NOO• (Model 1) Product
Cartesian Coordinate:	Thermochemical Values:
O 1.51962900 2.69065700 0.21501300<br>O 3.07604600 -1.60505200 -0.55713300<br>O 3.39752300 1.01013600 -0.62254100<br>C -0.16356700 -0.53199700 0.85096900<br>C -1.47566600 -1.08843100 1.34678400<br>C 0.83087400 -1.39982500 0.40062300<br>C 0.03924000 0.84638100 0.80165100<br>C 1.23375400 1.37056400 0.30430600<br>C 2.02243100 -0.87509000 -0.09350700<br>C 2.23289000 0.50362000 -0.14503400<br>C 0.51564500 3.60374900 0.62672400<br>C 2.94701500 -3.01864300 -0.56203600<br>C -3.78222300 -1.86128700 0.68525300<br>H -1.33011400 -2.09104100 1.75589700<br>H -1.88927500 -0.46474700 2.14420400<br>H 0.67440500 -2.47073500 0.44306600<br>H -0.74595900 1.50643300 1.14499900<br>H 3.95306100 0.26390100 -0.88285900<br>H 0.28297700 3.48424400 1.68904700<br>H -0.39603800 3.48251200 0.03413200<br>H 0.92740400 4.59686000 0.45795200<br>H 2.11384200 -3.33136600 -1.19748300<br>H 2.80477600 -3.40046300 0.45263400<br>H 3.87901300 -3.40610300 -0.96822400<br>H -3.89328800 -2.93289500 0.58969600<br>H -4.49560300 -1.30193500 1.27811600<br>C -2.53208800 -1.20342400 0.23607300<br>H -2.09898100 -1.72418500 -0.62303200<br>O -2.81689200 0.16178600 -0.16965800<br>N -3.34595800 0.19398600 -1.42601900<br>O -3.62309900 1.28721100 -1.74653800	• Electronic Energy = -858.196367<br>• Zero Point Energy Correction = 0.243273<br>• Thermal Correction to Energy = 0.261272<br>• Thermal Correction to Enthalpy = 0.262217<br>• Thermal Correction to Free Energy = 0.195336

Name	HAs RAF C-3a···NOO• (Model 1) Product
Cartesian Coordinate:	Thermochemical Values:
O -0.79821600 -2.74903300 0.26859300<br>O -2.72215300 1.43906700 -0.21476400<br>O -2.79583800 -1.18185600 -0.50558100<br>C 0.54933000 0.54651500 1.24115700<br>C 1.81434100 1.15918600 1.81517400<br>C -0.50287100 1.35435900 0.82194600<br>C 0.47630800 -0.83750400 1.07511600<br>C -0.64297800 -1.42276800 0.48528700<br>C -1.62605000 0.76697000 0.23843700<br>C -1.70569000 -0.61352700 0.06795600<br>C 2.98376500 1.01987700 0.89117400<br>C 0.30275900 -3.58920100 0.57150100<br>C -2.70103800 2.85688000 -0.14761800<br>H 1.62571200 2.22339000 2.01418600<br>H 2.05170400 0.69894500 2.77753500<br>H -0.44139600 2.42846600 0.94361700<br>H 1.31489300 -1.44893800 1.38463900<br>H 3.93355900 0.65991500 1.26602900	• Electronic Energy = -858.199864<br>• Zero Point Energy Correction = 0.244779<br>• Thermal Correction to Energy = 0.262384<br>• Thermal Correction to Enthalpy = 0.263328<br>• Thermal Correction to Free Energy = 0.198034

H -3.39785400 -0.46827700 -0.75359800<br>H 0.53385700 -3.57129000 1.64073100<br>H 1.18800200 -3.29890200 -0.00259600<br>H -0.00025500 -4.59475400 0.28618100<br>H -1.87294400 3.26172600 -0.73601100<br>H -2.62343200 3.19768000 0.88838100<br>H -3.64568700 3.19364900 -0.56969900<br>C 2.86412500 1.34328700 -0.55245800<br>O 2.45758500 0.16570400 -1.31532900<br>H 3.81914400 1.65019500 -0.97898700<br>H 2.11621900 2.11973100 -0.73815200<br>N 1.21492100 0.32364800 -1.86479200<br>O 0.86137600 -0.65157200 -2.40976300	

Name	HAs RAF C-1···NOO$^{\bullet}$ (Model 2) Product
Cartesian Coordinate:	Thermochemical Values:
O -0.17162600 2.70397800 0.03252600<br>O -2.48113000 -1.33505300 -0.18849200<br>C 1.15615600 -0.70965000 -0.42521100<br>C 2.45104800 -1.45953500 -0.61302300<br>C -0.06310100 -1.43474500 -0.38960600<br>C 1.14201200 0.69493500 -0.30503500<br>C -0.02879700 1.38230700 -0.13485000<br>C -1.24935300 -0.79059800 -0.22880900<br>C 2.49217100 -2.18123100 -1.93479600<br>C 1.01069300 3.49453600 0.04354100<br>C -2.57897400 -2.74711200 -0.34330100<br>C 2.58593200 -3.50054000 -2.06506800<br>H 3.28285900 -0.75196800 -0.55454600<br>H 2.57937300 -2.18739800 0.19574100<br>H -0.03507900 -2.51131700 -0.51200200<br>H 2.08244600 1.23079000 -0.33981200<br>H 2.42061100 -1.55364500 -2.82119200<br>H 1.53871900 3.41179900 -0.91014400<br>H 1.66800000 3.19111600 0.86295300<br>H 0.68380400 4.52062600 0.19423000<br>H -2.04318000 -3.25655800 0.46155100<br>H -2.17847100 -3.05376900 -1.31286100<br>H -3.63875500 -2.98375800 -0.28867800<br>H 2.60427500 -3.97349700 -3.04093700<br>H 2.65391400 -4.14972700 -1.19633300<br>C -1.34598800 0.69142100 -0.06890500<br>O -2.27856400 1.28857500 -0.87712600<br>H -3.10625200 0.79184500 -0.80712500<br>N -1.89312400 0.90696600 1.45488500<br>O -1.11101900 0.68355200 2.34803200<br>O -3.04928800 1.21891600 1.59113700	• Electronic Energy<br>= -858.190855<br>• Zero Point Energy Correction<br>= 0.244877<br>• Thermal Correction to Energy<br>= 0.262434<br>• Thermal Correction to Enthalpy<br>= 0.263378<br>• Thermal Correction to Free Energy<br>= 0.197957

Name	HAs RAF C-2···NOO$^{\bullet}$ (Model 2) Product
Cartesian Coordinate:	Thermochemical Values:
O 0.12694500 1.96202200 -1.27711900<br>O 2.86244200 -1.46080300 -0.15124500<br>C -0.85762300 -1.23176100 -0.06445500<br>C -2.04108100 -2.16815200 0.07968800<br>C 0.44903000 -1.83366000 -0.06873600<br>C -1.01806800 0.10752700 -0.15736300<br>C 1.57226500 -1.03325100 -0.14771100<br>C 1.45479700 0.35156100 -0.23549000<br>C -3.35557900 -1.57825500 -0.33625100<br>C -1.13878200 2.45722900 -1.71350900<br>C 3.09083600 -2.86005200 -0.03508200<br>C -4.38834300 -1.39896100 0.47983600<br>H -1.83815200 -3.06021100 -0.52343200<br>H -2.09486100 -2.50039900 1.12219100<br>H 0.53343300 -2.91036000 -0.00378000<br>H -2.00427000 0.55397300 -0.12413200<br>H -3.44012400 -1.28789200 -1.38217900<br>H -1.72925400 2.84390800 -0.88130900<br>H -1.69477600 1.68161400 -2.24537800<br>H -0.91031100 3.27303300 -2.39656500<br>H 2.65126400 -3.39507800 -0.88074200<br>H 2.68140100 -3.24119600 0.90383500<br>H 4.17070100 -2.99028500 -0.04263600<br>H -5.32343500 -0.97812900 0.12643400<br>H -4.33108800 -1.67053200 1.53022500	• Electronic Energy<br>= -858.180449<br>• Zero Point Energy Correction<br>= 0.244600<br>• Thermal Correction to Energy<br>= 0.262226<br>• Thermal Correction to Enthalpy<br>= 0.263171<br>• Thermal Correction to Free Energy<br>= 0.197312

C 0.13889500 1.03739000 -0.26362100<br>N 0.11979400 1.92135300 1.12484500<br>O 0.34400900 1.31687500 2.14747400<br>O -0.13981200 3.09585600 1.04713900<br>O 2.52297200 1.15242700 -0.27550900<br>H 3.32038300 0.60337200 -0.22619800	

Name	HAs RAF C-3···NOO• (Model 2) Product
Cartesian Coordinate:	Thermochemical Values:
O 0.01199300 2.68734400 -0.08773600<br>O 2.99480800 -0.88678000 -0.11276300<br>O 2.50411600 1.68260400 -0.35269300<br>C -0.66341000 -0.99895800 0.25269800<br>C -1.84013800 -1.92963000 0.39806200<br>C 0.62694600 -1.44742100 0.16266900<br>C 0.19369200 1.34496300 -0.04572100<br>C 1.69327500 -0.55043700 -0.00902300<br>C 1.45882200 0.85115400 -0.13518200<br>C -2.76804700 -1.83933900 -0.78664200<br>C -1.20881700 3.16218600 -0.65221200<br>C 3.35048400 -2.26320400 -0.01795500<br>C -3.99098900 -1.32155200 -0.73754000<br>H -1.46484400 -2.95124000 0.50033100<br>H -2.40233500 -1.69406400 1.31002200<br>H 0.81562600 -2.51380200 0.19713000<br>H -2.36921800 -2.20277100 -1.73175300<br>H 3.30608300 1.14636500 -0.40231800<br>H -2.06307300 2.95237400 -0.00245500<br>H -1.37565900 2.72613000 -1.64124500<br>H -1.09415600 4.24009300 -0.74682300<br>H 2.89479800 -2.83423800 -0.83030900<br>H 3.04606000 -2.67141700 0.94859100<br>H 4.43363700 -2.29885500 -0.10710300<br>H -4.61666300 -1.26265100 -1.62168600<br>H -4.40519600 -0.94398300 0.19332900<br>C -0.95291500 0.46137000 0.25749800<br>H -1.85576600 0.69799800 -0.31132500<br>N -1.46589100 0.82015300 1.73860700<br>O -0.64863900 0.85982500 2.62425900<br>O -2.65968700 0.97659200 1.87742100	• Electronic Energy = -858.183502<br>• Zero Point Energy Correction = 0.245341<br>• Thermal Correction to Energy = 0.263119<br>• Thermal Correction to Enthalpy = 0.264063<br>• Thermal Correction to Free Energy = 0.197402

Name	HAs RAF C-4···NOO• (Model 2) Product
Cartesian Coordinate:	Thermochemical Values:
O -1.40760400 2.41852600 -0.02800100<br>O 2.85060700 0.61635500 -0.28100300<br>O 1.19351500 2.63968600 -0.42008200<br>C -0.93604700 -2.37222500 0.10810400<br>C 1.10248600 -0.90997600 0.39601900<br>C -1.19838100 0.08949600 0.54765000<br>C -0.69234800 1.29417900 0.17355100<br>C 1.56873200 0.31201000 0.03757700<br>C 0.70724900 1.44468900 -0.06462000<br>C -1.06774300 -2.22115500 -1.37832600<br>C -2.80742300 2.34227000 0.19872200<br>C 3.81525400 -0.42569200 -0.18841800<br>C -2.21406200 -2.34452600 -2.03820100<br>H -0.27734600 -3.21276500 0.34640600<br>H -1.91653700 -2.56652200 0.55398300<br>H 1.74828800 -1.77466400 0.46075800<br>H -2.25900200 -0.04875200 0.70419000<br>H -0.15221100 -1.99930200 -1.92306500<br>H 2.14797700 2.54643200 -0.55715200<br>H -3.01999700 2.05607600 1.23257400<br>H -3.27507100 1.63028600 -0.48736500<br>H -3.19603100 3.34041500 0.00852500<br>H 3.57632400 -1.23304700 -0.88532600<br>H 3.86333400 -0.81387000 0.83205800<br>H 4.76919200 0.02166300 -0.45800400<br>H -2.25875700 -2.23789500 -3.11645800<br>H -3.14460400 -2.56089800 -1.52089200<br>C -0.33237400 -1.09803200 0.74918900<br>N -0.35342100 -1.47207100 2.31213900<br>O -1.10763800 -0.87549500 3.04272900<br>O 0.38286600 -2.36732700 2.66425100	• Electronic Energy = -858.188048<br>• Zero Point Energy Correction = 0.245382<br>• Thermal Correction to Energy = 0.262824<br>• Thermal Correction to Enthalpy = 0.263769<br>• Thermal Correction to Free Energy = 0.198600

Name	HAs RAF C-2a···NOO• (Model 2) Product
Cartesian Coordinate:	Thermochemical Values:
O -1.96537000 2.41430400 0.16165400<br>O -2.01088000 -2.21729300 0.06984200<br>O -3.22090700 0.09435600 0.44561400<br>C 0.78741800 0.08299100 -0.62016600<br>C 2.26106500 0.08735900 -0.94387100<br>C 0.11185600 -1.12627200 -0.46973100<br>C 0.11892200 1.28991000 -0.41368300<br>C -1.23001500 1.29925500 -0.05537900<br>C -1.23352100 -1.11427100 -0.10923800<br>C -1.91013500 0.08792300 0.09883600<br>C -1.30595900 3.66364100 0.04497000<br>C -1.38547200 -3.48815000 -0.04453500<br>C 4.59158600 0.15729000 0.01157900<br>H 2.54645500 -0.82568000 -1.47145300<br>H 2.51193800 0.93614200 -1.58495400<br>H 0.63397500 -2.06125400 -0.62918600<br>H 0.65735900 2.22123900 -0.53747700<br>H -3.50523800 -0.82595300 0.52149800<br>H -0.92184100 3.81560900 -0.96813200<br>H -0.48742300 3.74735800 0.76626300<br>H -2.05709100 4.42032700 0.26236800<br>H -0.57722800 -3.58981400 0.68481600<br>H -0.99662800 -3.64034500 -1.05486300<br>H -2.15911200 -4.22475400 0.16134500<br>H 5.30070700 0.64203000 0.66853400<br>H 4.95086000 -0.47229300 -0.79182100<br>C 3.14203100 0.21737200 0.30450400<br>H 2.87283400 1.09324500 0.89314600<br>N 2.77462800 -0.95392900 1.23686200<br>O 2.96859200 -2.07677700 0.82263600<br>O 2.31117300 -0.68714400 2.32146900	• Electronic Energy = -858.205203<br>• Zero Point Energy Correction = 0.245426<br>• Thermal Correction to Energy = 0.262954<br>• Thermal Correction to Enthalpy = 0.263899<br>• Thermal Correction to Free Energy = 0.198378

Name	HAs RAF C-3a···NOO• (Model 2) Product
Cartesian Coordinate:	Thermochemical Values:
O -0.92047200 2.64783700 0.30694500<br>O -3.06378700 -1.45520700 0.04884900<br>O -3.12086500 1.15828000 0.40100900<br>C 0.52533900 -0.68708300 -0.32660600<br>C 1.87084300 -1.33013900 -0.58746400<br>C -0.63484200 -1.45420100 -0.27473900<br>C 0.46136200 0.69342400 -0.13177700<br>C -0.76125100 1.31855000 0.11013300<br>C -1.85642100 -0.82688200 -0.03002500<br>C -1.93010400 0.55210100 0.16250100<br>C 2.85151400 -1.09343100 0.51362700<br>C 0.24599900 3.45435000 0.26332200<br>C -3.08882000 -2.86252600 -0.13554600<br>H 1.72872900 -2.41023300 -0.73394400<br>H 2.28586800 -0.95208000 -1.53021700<br>H -0.58320300 -2.52520400 -0.42379100<br>H 1.37732700 1.26879000 -0.17066700<br>H 2.50074600 -1.02232600 1.53518800<br>H -3.80111500 0.47242400 0.39738700<br>H 0.73154900 3.39606500 -0.71531700<br>H 0.95727900 3.16456700 1.04259100<br>H -0.08891600 4.47445800 0.44055300<br>H -2.49237100 -3.36633200 0.62990400<br>H -2.72214200 -3.13205200 -1.12970500<br>H -4.13048600 -3.16152900 -0.03946400<br>C 4.28976600 -0.94788500 0.23716300<br>H 4.64449700 -1.56920300 -0.58805600<br>H 4.92488900 -1.11241600 1.10774300<br>N 4.67210400 0.47517600 -0.21575600<br>O 3.83226800 1.34642500 -0.18288100<br>O 5.81669100 0.63243100 -0.57627600	• Electronic Energy = -858.207178<br>• Zero Point Energy Correction = 0.245887<br>• Thermal Correction to Energy = 0.263418<br>• Thermal Correction to Enthalpy = 0.264362<br>• Thermal Correction to Free Energy = 0.196658

Name	HPns (4-propenylsyringol)
Cartesian Coordinate:	Thermochemical Values:

O -2.41840400 -1.86018800 -0.02928400<br>O -1.13034200 2.58925800 0.00184100<br>O -2.99663200 0.73535100 -0.06397100<br>C 0.99305900 -0.43113900 0.06215100<br>C -1.37007200 -1.00315500 -0.00366400<br>C -0.67271600 1.30582900 0.00975600<br>C 0.66274900 0.92885300 0.05453000<br>C -0.02825600 -1.38564000 0.04108700<br>C -1.69627500 0.35321600 -0.02322700<br>C 2.38864900 -0.90609600 0.09140800<br>C 3.48968300 -0.17453200 -0.09526900<br>C -2.12900900 -3.24768100 -0.00153200<br>C -0.16929300 3.63388600 0.02809400<br>C 4.88092100 -0.72540900 -0.05046100<br>H 1.43781100 1.68179200 0.09698300<br>H 0.23801000 -2.43457900 0.05373400<br>H 2.50451100 -1.97384700 0.27141800<br>H 3.40241400 0.89054100 -0.30067300<br>H -3.01478400 1.70133300 -0.06500000<br>H -1.59684400 -3.52302200 0.91392200<br>H -1.54035100 -3.54709200 -0.87374100<br>H -3.09175600 -3.75449900 -0.02511700<br>H 0.48183400 3.58568900 -0.84895400<br>H 0.43149500 3.58762400 0.94039600<br>H -0.73383500 4.56381100 0.01143600<br>H 5.40013500 -0.55188600 -0.99801900<br>H 5.47086800 -0.23142800 0.72786600<br>H 4.87686400 -1.79859500 0.14981200	• Electronic Energy = -653.152044<br>• Zero Point Energy Correction = 0.232584<br>• Thermal Correction to Energy = 0.247290<br>• Thermal Correction to Enthalpy = 0.248234<br>• Thermal Correction to Free Energy = 0.189859

Name	HPns 1-OH radical
Cartesian Coordinate:	Thermochemical Values:
O 2.56234400 -1.69343500 -0.00004000<br>O 0.92673000 2.69846800 -0.00010700<br>O 2.92244000 0.94428600 -0.00018800<br>C -0.91379300 -0.48609500 0.00015100<br>C 1.47098500 -0.92375400 0.00000600<br>C 0.59950200 1.40253400 -0.00001700<br>C -0.68208800 0.91033900 0.00012100<br>C 0.17340400 -1.38559200 0.00011500<br>C 1.76865300 0.51182200 -0.00007300<br>C -2.25679800 -1.04982500 0.00018700<br>C -3.41389300 -0.36901900 -0.00013600<br>C 2.38067300 -3.10281600 0.00001700<br>C -0.12878200 3.64894200 0.00014800<br>C -4.76090500 -1.01299400 -0.00015600<br>H -1.52417000 1.58761600 0.00019500<br>H -0.03955900 -2.44690900 0.00014700<br>H -2.30153800 -2.13711600 0.00046300<br>H -3.40530800 0.71821100 -0.00044200<br>H 1.84185600 -3.42586900 -0.89499100<br>H 1.84197800 -3.42581200 0.89511900<br>H 3.37968500 -3.53278200 -0.00004100<br>H -0.74846400 3.54313600 0.89516500<br>H -0.74877100 3.54329300 -0.89467600<br>H 0.34860200 4.62617800 0.00014900<br>H -5.33636800 -0.70164500 0.87728100<br>H -5.33586100 -0.70255600 -0.87825500<br>H -4.68593600 -2.10156100 0.00040000	• Electronic Energy = -652.519011<br>• Zero Point Energy Correction = 0.220157<br>• Thermal Correction to Energy = 0.234335<br>• Thermal Correction to Enthalpy = 0.235279<br>• Thermal Correction to Free Energy = 0.178404

Name	HPns 3a-CH radical
Cartesian Coordinate:	Thermochemical Values:

O 2.48793300 -1.71611500 -0.00004300<br>O 0.89905000 2.63490700 0.00015900<br>O 2.88167200 0.90862500 0.00009700<br>C -1.02576700 -0.52705500 -0.00007200<br>C 1.37971400 -0.93947100 -0.00003400<br>C 0.52487200 1.32559500 0.00006200<br>C -0.77897800 0.86305800 0.00000200<br>C 0.07409400 -1.41281700 -0.00008200<br>C 1.61310600 0.44168500 0.00003800<br>C -2.34764000 -1.08064200 -0.00012600<br>C -3.56136100 -0.36298000 -0.00021100<br>C 2.29886700 -3.12142400 -0.00008800<br>C -0.12815900 3.61619700 0.00039300<br>C -4.78747000 -0.95546500 -0.00021600<br>H -1.59948800 1.56555700 0.00002200<br>H -0.12175100 -2.47708000 -0.00013300<br>H -2.41559600 -2.16546800 -0.00010000<br>H -3.52534500 0.72320700 -0.00028500<br>H 2.83740900 1.87428200 0.00016400<br>H 1.76007600 -3.44764600 -0.89463300<br>H 1.76004000 -3.44769600 0.89441800<br>H 3.29588400 -3.55741500 -0.00008000<br>H -0.74939900 3.52793500 0.89565100<br>H -0.74947600 3.52829500 -0.89484800<br>H 0.37573600 4.58034000 0.00056200<br>H -5.69687300 -0.36769100 -0.00029400<br>H -4.88814200 -2.03603400 -0.00014000	• Electronic Energy<br>= -652.511563<br>• Zero Point Energy Correction<br>= 0.219345<br>• Thermal Correction to Energy<br>= 0.233597<br>• Thermal Correction to Enthalpy<br>= 0.234541<br>• Thermal Correction to Free Energy<br>= 0.177689

Name	HPns 2'-CH_3 radical
Cartesian Coordinate:	Thermochemical Values:
O -2.38611900 -1.84848000 0.06272100<br>O -1.13036500 2.62055200 -0.17981600<br>O -3.00257900 0.73037100 -0.02024100<br>C 1.00964800 -0.38137900 -0.08023800<br>C -1.34951600 -0.98198300 0.00125800<br>C -0.67960300 1.32359600 -0.10671900<br>C 0.66132200 0.96948700 -0.13787300<br>C -0.00452800 -1.34587200 -0.01790900<br>C -1.69639600 0.37314800 -0.04038300<br>C 2.41061800 -0.84073000 -0.09412800<br>C 3.49930600 -0.08451300 0.06279600<br>C -2.07913700 -3.23249000 0.10669500<br>C -0.30555500 3.62112400 0.23645900<br>C 4.89960200 -0.61190200 0.03478000<br>H 1.41260100 1.74244100 -0.23853800<br>H 0.27433700 -2.39111600 0.01809300<br>H 2.54109900 -1.91245100 -0.23522900<br>H 3.39290800 0.98574300 0.22812500<br>H -3.04989100 1.69462700 -0.04798000<br>H -1.49037900 -3.47857300 0.99528000<br>H -1.53976900 -3.54607100 -0.79193800<br>H -3.03558100 -3.74932600 0.15252500<br>H -0.73364400 4.60231000 0.09464900<br>H 0.37605500 3.40242400 1.05022100<br>H 5.47814300 -0.13587800 -0.76298500<br>H 5.41737400 -0.39298900 0.97365000<br>H 4.91469300 -1.69160900 -0.12560900	• Electronic Energy<br>= -652.484834<br>• Zero Point Energy Correction<br>= 0.218614<br>• Thermal Correction to Energy<br>= 0.233140<br>• Thermal Correction to Enthalpy<br>= 0.234084<br>• Thermal Correction to Free Energy<br>= 0.176708

Name	HPns HAT 3a-CH···NOO• Transition States Structure
Cartesian Coordinate:	Thermochemical Values:
O 3.41565600 -1.55499300 0.51615900<br>O 1.63465400 2.64400900 -0.25436100<br>O 3.61539900 1.07137200 0.39769000<br>C -0.01880600 -0.64734200 -0.52662200<br>C 2.30626000 -0.88657900 0.15389200<br>C 1.34163700 1.32342500 -0.26735300<br>C 0.12652300 0.76125200 -0.57823800<br>C 1.08336900 -1.45277800 -0.14827500<br>C 2.44261100 0.51561400 0.09531100<br>C -1.24912400 -1.29594700 -0.82519100<br>C -2.38727000 -0.69145500 -1.33273300<br>C 3.32092500 -2.97049700 0.60989400<br>C 0.57750800 3.55810500 -0.53346900	• Electronic Energy<br>= -858.176340<br>• Zero Point Energy Correction<br>= 0.238933<br>• Thermal Correction to Energy<br>= 0.256638<br>• Thermal Correction to Enthalpy<br>= 0.257582

H -0.71565700 1.38965800 -0.83071600<br>H 0.94816800 -2.52548500 -0.10525300<br>H -1.29217700 -2.36654900 -0.64089300<br>H -2.35627800 0.36388200 -1.58395100<br>H 3.52470500 2.03397100 0.32633000<br>H 3.06512500 -3.40974500 -0.35843800<br>H 2.58021000 -3.26311500 1.35917400<br>H 4.30571500 -3.31485500 0.91725800<br>H -0.22951600 3.44306000 0.19417700<br>H 0.19655800 3.40618100 -1.54630300<br>H 1.01052300 4.55188600 -0.44944500<br>H -4.17887200 -1.05552000 -0.41938200<br>O -4.58726300 -0.52444400 0.86759300<br>C -3.63226000 -1.36639900 -1.48223500<br>H -4.30968400 -0.95365500 -2.22827500<br>H -3.58942100 -2.45480100 -1.46845500<br>N -3.66776300 0.33158500 1.02108400<br>O -3.67260800 0.95775500 2.06000600	• Thermal Correction to Free Energy<br>= 0.190892

Name	HPns HAT 3a-CH···NOO• Reactant Complex
Cartesian Coordinate:	Thermochemical Values:
O 2.72673900 -1.74683900 -0.07001200<br>O 1.12904300 2.56338500 -0.64387100<br>O 3.10405000 0.87910700 -0.19911200<br>C -0.74055300 -0.62163000 -0.68687400<br>C 1.63083100 -0.98742000 -0.29972300<br>C 0.77411100 1.24918300 -0.59878000<br>C -0.51383300 0.75840000 -0.76769700<br>C 0.33748200 -1.48385100 -0.46477300<br>C 1.85195000 0.38979000 -0.36308800<br>C -2.09075400 -1.20114500 -0.80276900<br>C -3.24606300 -0.54150000 -0.69031900<br>C 2.54107500 -3.14942700 0.02344600<br>C 0.09133200 3.52034200 -0.79987000<br>H -1.33386800 1.43441200 -0.96843900<br>H 0.15076200 -2.54829700 -0.40606700<br>H -2.11986200 -2.27714900 -0.96748100<br>H -3.23622200 0.52849900 -0.49221700<br>H 3.04937500 1.84230000 -0.25348100<br>H 2.14672400 -3.55987400 -0.91083200<br>H 1.87207800 -3.40518500 0.85044000<br>H 3.52681200 -3.56944500 0.21375200<br>H -0.62728500 3.44982200 0.02183500<br>H -0.42232400 3.38659700 -1.75549200<br>H 0.57363700 4.49533100 -0.78003000<br>H -5.17679500 -1.02702300 0.10807300<br>O 0.15410000 0.19216700 2.48994300<br>C -4.59511500 -1.17908500 -0.80625600<br>H -5.16869100 -0.73079500 -1.62349700<br>H -4.51397400 -2.25200000 -0.99083800<br>N -0.97493900 0.17007400 2.14397100<br>O -1.88125300 0.92722300 2.23389000	• Electronic Energy<br>= -858.211329<br>• Zero Point Energy Correction<br>= 0.242853<br>• Thermal Correction to Energy<br>= 0.262287<br>• Thermal Correction to Enthalpy<br>= 0.263231<br>• Thermal Correction to Free Energy<br>= 0.191868

Name	HPns HAT 3a-CH···NOO• Product Complex
Cartesian Coordinate:	Thermochemical Values:
O 3.22549500 -1.46270800 0.49930100<br>O 1.27112700 2.61267600 -0.51124900<br>O 3.33182700 1.17495000 0.25424000<br>C -0.23604300 -0.76471400 -0.63114900<br>C 2.09268400 -0.84926300 0.08773500<br>C 1.04910100 1.27145400 -0.45185100<br>C -0.14326600 0.63951400 -0.75156200<br>C 0.89757600 -1.49141400 -0.20351600<br>C 2.17479200 0.54448600 -0.03663300<br>C -1.44256800 -1.48152800 -0.90670400<br>C -2.64600300 -0.93208200 -1.40232500<br>C 3.18293600 -2.87183200 0.65360000<br>C 0.16407300 3.45045500 -0.81392700<br>H -1.00234400 1.21924300 -1.05703600<br>H 0.81751300 -2.56637900 -0.10785200<br>H -1.43320300 -2.54897500 -0.70118600<br>H -2.66194800 0.12097900 -1.67090700<br>H 3.18721600 2.12356300 0.13464100<br>H 2.95980500 -3.36681900 -0.29628300<br>H 2.44348300 -3.16447800 1.40482500	• Electronic Energy<br>= -858.209966<br>• Zero Point Energy Correction<br>= 0.241978<br>• Thermal Correction to Energy<br>= 0.261008<br>• Thermal Correction to Enthalpy<br>= 0.261952<br>• Thermal Correction to Free Energy<br>= 0.191910

H 4.17521300 -3.16607600 0.98956100<br>H -0.63399700 3.31646100 -0.07826400<br>H -0.21570800 3.24540000 -1.81826900<br>H 0.53648000 4.47149200 -0.76779600<br>H -3.63289200 -0.83819600 0.72820600<br>O -3.44267700 -0.45863700 1.60657500<br>C -3.80226200 -1.64145300 -1.54021000<br>H -4.70102100 -1.17972700 -1.93013900<br>H -3.84573100 -2.69748700 -1.29115700<br>N -2.76861800 0.69629300 1.32302600<br>O -2.43060500 1.24812000 2.29859800	

Name	HPns RAF C-1···NO• Product
Cartesian Coordinate:	Thermochemical Values:
O -1.82315600 2.21041900 -0.15781500<br>O -1.00946300 -2.38367400 -0.25781100<br>O -2.39707400 -0.23659800 -1.16645500<br>C 1.41421300 0.40715100 -0.03806200<br>C -0.85912900 1.27547600 -0.15783100<br>C -0.41195800 -1.17902700 -0.16984000<br>C 0.91823600 -0.93282600 -0.09099100<br>C 0.47945000 1.48754500 -0.08412100<br>C 2.81766800 0.71128500 0.05018400<br>C 3.84313700 -0.16502100 0.12981000<br>C -1.41192100 3.56935200 -0.11244800<br>C -0.17419500 -3.53391700 -0.25827900<br>C 5.27851900 0.24131300 0.21798300<br>H 1.61630300 -1.75952200 -0.10056600<br>H 0.86752100 2.49808300 -0.04891300<br>H 3.06219700 1.77240700 0.05667900<br>H 3.64510300 -1.23371100 0.13455500<br>H -2.87437800 -1.06295400 -1.00544000<br>H -0.79764700 3.81583000 -0.98289900<br>H -0.85472300 3.77456200 0.80600200<br>H -2.32430000 4.16116500 -0.12840700<br>H 0.40112000 -3.59125900 0.66972900<br>H 0.50273000 -3.51700800 -1.11668300<br>H -0.84008000 -4.39062200 -0.33198300<br>H 5.73875700 -0.14352700 1.13395600<br>H 5.85397900 -0.17039800 -0.61765000<br>H 5.38679100 1.32746100 0.20821300<br>C -1.45564100 -0.09497100 -0.16281500<br>N -2.13784700 -0.27004600 1.23961100<br>O -3.15139800 -0.87869100 1.17015400	• Electronic Energy = -782.992084<br>• Zero Point Energy Correction = 0.238859<br>• Thermal Correction to Energy = 0.255904<br>• Thermal Correction to Enthalpy = 0.256848<br>• Thermal Correction to Free Energy = 0.193612

Name	HPns RAF C-3···NO• Product
Cartesian Coordinate:	Thermochemical Values:
O 2.87434000 -1.62242300 0.11726400<br>O 0.79657300 2.53562700 -0.12955000<br>O 2.99059500 1.01046500 0.04267900<br>C -0.72642500 -0.89344200 -0.22174400<br>C 1.69535900 -0.99003800 -0.00054000<br>C 0.63492900 1.18235400 -0.15025100<br>C 0.45139300 -1.61490100 -0.12484900<br>C 1.76930000 0.43445800 -0.02121000<br>C -2.00052400 -1.56562300 -0.28668200<br>C -3.20720200 -0.96685800 -0.25564600<br>C 2.86510300 -3.04349400 0.13306100<br>C 0.05274300 3.28283500 -1.09683000<br>C -4.50638500 -1.70587400 -0.28386800<br>H 0.39275300 -2.69626800 -0.14562600<br>H -1.96445400 -2.65290000 -0.32981800<br>H -3.27726800 0.11714100 -0.20669900<br>H 2.85573800 1.96816900 0.03176700<br>H 2.47238500 -3.44208700 -0.80620800<br>H 2.27603500 -3.42068900 0.97342100<br>H 3.90315900 -3.34636300 0.25046300<br>H -1.00396000 3.32374900 -0.82806900<br>H 0.17477300 2.84660800 -2.09193600<br>H 0.46689400 4.28938200 -1.08394700<br>H -5.10061400 -1.48041000 0.60724900<br>H -5.10818400 -1.40463400 -1.14726200<br>H -4.34990900 -2.78510500 -0.33073800<br>C -0.71662400 0.58710300 -0.12331600<br>H -1.41485900 1.07994700 -0.80797600	• Electronic Energy = -782.985573<br>• Zero Point Energy Correction = 0.238866<br>• Thermal Correction to Energy = 0.255902<br>• Thermal Correction to Enthalpy = 0.256847<br>• Thermal Correction to Free Energy = 0.194068

N -1.24616000 0.99332700 1.32493000<br>O -1.59518000 2.12771300 1.37362200	

Name	HPns RAF C-4···NO• Product
Cartesian Coordinate:	Thermochemical Values:
O 2.44967500 -1.92285200 -0.36470500<br>O 1.40052900 2.58114600 -0.24100300<br>O 3.14018800 0.62551300 -0.37688700<br>C 1.44605600 -1.05023700 -0.14361700<br>C 0.86557900 1.34419800 -0.08889200<br>C -0.45462500 1.06048100 0.09755400<br>C 0.14056100 -1.37594100 0.06856000<br>C 1.84677800 0.31969600 -0.18027400<br>C -2.23903900 -0.72210000 -0.13617500<br>C -2.95970800 -0.04394000 -1.02421000<br>C 2.12441000 -3.30360100 -0.32806700<br>C 0.52536200 3.69587000 -0.12174600<br>C -4.29739200 -0.48047500 -1.53577500<br>H -1.19772300 1.84236700 0.17974000<br>H -0.18692700 -2.40667600 0.08920800<br>H -2.62428000 -1.66095500 0.25955000<br>H -2.56451000 0.88467100 -1.43083600<br>H 3.21565400 1.58998900 -0.40660800<br>H 1.40537500 -3.55732800 -1.11220100<br>H 1.72002800 -3.58381000 0.64889200<br>H 3.05662200 -3.83626600 -0.50361800<br>H 0.05548900 3.71097300 0.86491500<br>H -0.24072400 3.66904500 -0.90075200<br>H 1.14488800 4.58081900 -0.24831100<br>H -5.05551800 0.28171700 -1.33350900<br>H -4.26917500 -0.61994100 -2.62052700<br>H -4.61557100 -1.41658300 -1.07342900<br>C -0.88038000 -0.33558400 0.37123200<br>N -0.91923400 -0.28884100 1.99044800<br>O -2.00471700 -0.29709800 2.45544700	• Electronic Energy = -782.975965<br>• Zero Point Energy Correction = 0.238229<br>• Thermal Correction to Energy = 0.255024<br>• Thermal Correction to Enthalpy = 0.256390<br>• Thermal Correction to Free Energy = 0.192215

Name	HPns RAF C-1a···NO• Product
Cartesian Coordinate:	Thermochemical Values:
O 1.74921600 2.56953800 -0.02320800<br>O 2.67973600 -1.96865800 -0.03400700<br>O 3.46452200 0.54732100 -0.04602100<br>C -0.59461200 -0.27677200 -0.01456200<br>C 1.21616600 1.32654100 -0.02524000<br>C 1.67994400 -1.04396200 -0.02709400<br>C 0.31687100 -1.32951400 -0.01051000<br>C -0.15005500 1.04558000 -0.01861800<br>C 2.13702600 0.27431800 -0.03442600<br>C -2.86073000 0.02192700 -1.08661900<br>C 0.84878900 3.66536700 -0.00587500<br>C 2.30861700 -3.33939800 -0.01738500<br>C -4.23022500 -0.47433100 -1.36204400<br>H -0.03525100 -2.35311700 0.00131400<br>H -0.87239700 1.85134200 -0.00232600<br>H -2.50542400 0.93939600 -1.54053300<br>H 3.93516000 -0.29653500 -0.04378500<br>H 0.22500600 3.65009300 0.89261800<br>H 0.21272400 3.66823900 -0.89613600<br>H 1.46744100 4.56048700 -0.00157800<br>H 1.72082000 -3.59372600 -0.90351700<br>H 1.74254700 -3.57797200 0.88704900<br>H 3.23829600 -3.90438500 -0.02368000<br>H -4.88289200 -0.32909100 -0.48665900<br>H -4.68405500 0.04200900 -2.20767200<br>H -4.22932200 -1.55144100 -1.56162800<br>C -2.06631500 -0.56938500 0.00028200<br>H -2.27849100 -1.63397400 0.13148800<br>N -2.69551500 0.15042700 1.22835600<br>O -3.62077700 -0.43901900 1.69937500	• Electronic Energy = -782.997263<br>• Zero Point Energy Correction = 0.238612<br>• Thermal Correction to Energy = 0.255725<br>• Thermal Correction to Enthalpy = 0.256669<br>• Thermal Correction to Free Energy = 0.192789

Name	HPns RAF C-2a···NO$^{\bullet}$ Product
Cartesian Coordinate:	Thermochemical Values:
O -3.00725900 -1.78848400 0.14105600<br>O -1.57171100 2.61014700 -0.05385500<br>O -3.47997600 0.81868800 0.14700900<br>C 0.45246400 -0.48922700 -0.21726700<br>C -1.92774900 -0.98280700 0.02344200<br>C -1.15203800 1.31637700 -0.08469800<br>C 0.15873500 0.89896600 -0.20816200<br>C -0.61879600 -1.41479400 -0.09358500<br>C -2.20520800 0.39491800 0.02818000<br>C 1.76675800 -0.98493600 -0.34476300<br>C -2.77537400 -3.18755300 0.15366600<br>C -0.58242000 3.62703900 -0.13443100<br>C 4.15950200 -0.88817700 -1.11241200<br>H 0.95427600 1.62760700 -0.27425200<br>H -0.38735000 -2.47177900 -0.09617900<br>H 1.91858700 -2.05726600 -0.26769100<br>H -3.46976800 1.78577400 0.14238700<br>H -2.14255500 -3.47457100 0.99860900<br>H -2.31487500 -3.51960200 -0.78153500<br>H -3.75354100 -3.65214000 0.25998600<br>H -0.03798800 3.56420500 -1.08051800<br>H 0.11604300 3.55530100 0.70339000<br>H -1.11752500 4.57278700 -0.08437600<br>H 3.91527700 -1.11546100 -2.15150600<br>H 5.06543600 -0.27972400 -1.09189700<br>H 4.36352200 -1.82486700 -0.59054900<br>C 2.99554900 -0.15562100 -0.47686100<br>H 2.78489600 0.78733300 -0.99079400<br>N 3.26910400 0.28422400 0.95894100<br>O 4.25811000 -0.17688800 1.43999900	• Electronic Energy = -783.012879<br>• Zero Point Energy Correction = 0.239916<br>• Thermal Correction to Energy = 0.256519<br>• Thermal Correction to Enthalpy = 0.257463<br>• Thermal Correction to Free Energy = 0.194613

Name	HPns RAF C-1···NOO$^{\bullet}$ (Model 1) Product
Cartesian Coordinate:	Thermochemical Values:
O -2.05967500 2.34584700 -0.10158500<br>O -1.28268100 -2.05078400 -0.96876100<br>O -2.93270300 -0.16226800 -0.05997700<br>C 1.16196300 0.46402500 -0.28463900<br>C -1.12218200 1.36666600 -0.13792500<br>C 0.71609200 -0.81537800 -0.25360800<br>C 0.24664900 1.57487500 -0.22666200<br>C -1.61368500 0.06473300 -0.10279000<br>C 2.60117500 0.78946200 -0.37336900<br>C 3.58247300 0.05840100 0.15765900<br>C -1.60112000 3.68462800 -0.19519900<br>C -0.53177200 -3.23486900 -1.23217300<br>C 5.03702800 0.39207400 0.05536600<br>H 1.40571800 -1.64904600 -0.30866300<br>H 0.65146000 2.57718600 -0.26109200<br>H 2.84985600 1.71577900 -0.88911900<br>H 3.32538800 -0.83872000 0.71823100<br>H -3.07656200 -1.11317700 -0.18132200<br>H -1.06968300 3.85488400 -1.13623400<br>H -0.95087400 3.94020700 0.64670600<br>H -2.49208300 4.30874100 -0.16523500<br>H -0.18827200 -3.70136500 -0.30719300<br>H 0.31778900 -3.01563100 -1.88290700<br>H -1.21105900 -3.91226100 -1.74708000<br>H 5.47973100 0.50674000 1.04922200<br>H 5.58382200 -0.41454500 -0.44266900<br>H 5.19619900 1.31472800 -0.50579400<br>C -0.72169600 -1.13750300 -0.06644100<br>O -0.94472800 -1.84311000 1.20863600<br>O -0.42423900 -1.07072400 2.27238900<br>H -1.19876300 -0.56787800 2.56621000	• Electronic Energy = -858.199836<br>• Zero Point Energy Correction = 0.243473<br>• Thermal Correction to Energy = 0.261380<br>• Thermal Correction to Enthalpy = 0.262324<br>• Thermal Correction to Free Energy = 0.196740

Name	HPns RAF C-2···NOO$^{\bullet}$ (Model 1) Product
Cartesian Coordinate:	Thermochemical Values:

O -1.91569400 2.52618100 -0.04280600<br>O -1.30822500 -1.80340300 -1.36654300<br>O -2.89191300 0.08950800 -0.43265800<br>C 1.22603800 0.53891600 -0.36206900<br>C -1.01902700 1.52189000 -0.17415000<br>C 0.72562600 -0.71035700 -0.53557800<br>C 0.35769000 1.67057700 -0.16556400<br>C -1.56915400 0.25273000 -0.35784000<br>C 2.67885700 0.80654500 -0.36929800<br>C 3.61384300 -0.04248500 0.06055700<br>C -1.39993900 3.84044600 0.10189300<br>C -0.63619300 -3.02973300 -1.65418700<br>C 5.08258800 0.23810000 0.04401700<br>H 1.37686400 -1.55802300 -0.70955900<br>H 0.80712700 2.64442500 -0.02740700<br>H 2.97959400 1.79002000 -0.72642900<br>H 3.30616300 -1.00348000 0.46909200<br>H -3.07064800 -0.82835700 -0.68921300<br>H -0.80295500 4.12692300 -0.76884600<br>H -0.79678500 3.92887400 1.01001800<br>H -2.26622300 4.49425700 0.17832000<br>H -0.37789700 -3.56083200 -0.73604800<br>H 0.25835400 -2.84667800 -2.25286300<br>H -1.33865800 -3.62720400 -2.23205600<br>H 5.49858400 0.18225600 1.05428900<br>H 5.61053900 -0.50899100 -0.55654600<br>H 5.29556100 1.22630800 -0.36739200<br>C -0.72668200 -0.97857900 -0.41211600<br>O -0.99071400 -1.76851800 0.85787200<br>N -0.53839500 -1.10284000 1.95439800<br>O -0.73254800 -1.71725800 2.93661700	• Electronic Energy = -858.182897<br>• Zero Point Energy Correction = 0.242687<br>• Thermal Correction to Energy = 0.260778<br>• Thermal Correction to Enthalpy = 0.261722<br>• Thermal Correction to Free Energy = 0.196094

Name	HPns RAF C-3···NOO• (Model 1) Product
Cartesian Coordinate:	Thermochemical Values:
O 2.90109500 -1.66498900 0.12751700<br>O 0.95747900 2.50747800 -0.52727900<br>O 3.10291700 0.93065500 -0.23433600<br>C -0.67442400 -0.88933800 -0.34501300<br>C 1.74013400 -1.02585600 -0.07492900<br>C 0.75850700 1.15800000 -0.45167600<br>C 0.48652700 -1.62536800 -0.13918000<br>C 1.85980700 0.39520300 -0.26113200<br>C -1.94993700 -1.55025900 -0.46568400<br>C -3.14803100 -0.95641200 -0.63542400<br>C 2.85879300 -3.07283500 0.31923400<br>C 0.06897500 3.24564700 -1.36435500<br>C -4.43585400 -1.70304600 -0.77177100<br>H 0.39774300 -2.70086900 -0.04620000<br>H -1.91876100 -2.63742800 -0.41151700<br>H -3.21320000 0.12831600 -0.66947700<br>H 3.00629700 1.88655200 -0.34247900<br>H 2.46347200 -3.57497600 -0.56778700<br>H 2.25432600 -3.32828100 1.19355500<br>H 3.88855600 -3.38182900 0.48387000<br>H -0.94020300 3.26093800 -0.94559700<br>H 0.05293600 2.82855200 -2.37522300<br>H 0.45818000 4.26133800 -1.39624000<br>H -5.13786400 -1.41626600 0.01775300<br>H -4.92346500 -1.47063100 -1.72400200<br>H -4.27900800 -2.78206000 -0.71924300<br>C -0.62283800 0.60797300 -0.41533800<br>O -1.36651200 1.23138300 0.71036600<br>H -1.22025000 0.96927900 -1.25825600<br>N -0.84139500 0.83890800 1.90705600<br>O -1.42609700 1.31831800 2.80411400	• Electronic Energy = -858.191349<br>• Zero Point Energy Correction = 0.244072<br>• Thermal Correction to Energy = 0.261966<br>• Thermal Correction to Enthalpy = 0.262911<br>• Thermal Correction to Free Energy = 0.197637

Name	HPns RAF C-4···NOO• (Model 1) Product
Cartesian Coordinate:	Thermochemical Values:
O 2.40510100 -1.69144400 -0.93864200<br>O 1.12580400 2.71434200 -0.29222800<br>O 2.90616500 0.90127100 -0.90868900<br>C 1.38409600 -0.90498200 -0.53801300<br>C 0.67569500 1.43751600 -0.19724000	• Electronic Energy = -858.183865<br>• Zero Point Energy Correction

C -0.57042600 1.05289000 0.16968200<br>C 0.15212400 -1.33466200 -0.15784300<br>C 1.68420900 0.48982100 -0.54782800<br>C -2.23046900 -0.76403100 -0.37643900<br>C -2.88119600 -0.01992900 -1.26487400<br>C 2.18173900 -3.09266700 -0.92603500<br>C 0.22344500 3.75456500 0.06223700<br>C -4.14569700 -0.43834700 -1.94757000<br>H -1.33660000 1.76849000 0.43670400<br>H -0.10436500 -2.38582300 -0.15225800<br>H -2.60456300 -1.74940200 -0.10408900<br>H -2.47884100 0.95339500 -1.53782800<br>H 2.92191400 1.86853100 -0.85912800<br>H 1.37494900 -3.36690100 -1.61192600<br>H 1.94301400 -3.44085600 0.08307500<br>H 3.11224300 -3.54783800 -1.25856500<br>H -0.08897900 3.65331200 1.10466800<br>H -0.65156500 3.74422800 -0.59303100<br>H 0.76893300 4.68625700 -0.06966600<br>H -4.94522700 0.28246800 -1.75387500<br>H -4.00407400 -0.47158500 -3.03189100<br>H -4.47596500 -1.42222500 -1.61012300<br>C -0.92244500 -0.39172800 0.28393600<br>O -1.24234600 -0.68333200 1.73379000<br>N -0.15570000 -0.49747900 2.52014000<br>O -0.40065300 -0.71838500 3.65037900	= 0.243334<br>• Thermal Correction to Energy<br>= 0.261227<br>• Thermal Correction to Enthalpy<br>= 0.262171<br>• Thermal Correction to Free Energy<br>= 0.196996

Name	HPns RAF C-1a···NOO$^{\bullet}$ (Model 1) Product
Cartesian Coordinate:	Thermochemical Values:
O 3.08813800 -1.73892600 0.17878000<br>O 1.60320200 2.63301500 -0.19721000<br>O 3.54005700 0.87539500 0.09569300<br>C -0.35434100 -0.48781800 -0.25575200<br>C 2.00724300 -0.94079900 0.02416900<br>C 1.20895200 1.33087200 -0.17738100<br>C -0.10117100 0.88342100 -0.30232400<br>C 0.69062100 -1.39327900 -0.09035000<br>C 2.26661100 0.43037600 -0.01807600<br>C -2.58228100 -0.44088200 -1.45533600<br>C 2.86314000 -3.13789300 0.24291900<br>C 0.59294300 3.62557600 -0.31401300<br>C -3.99589800 -0.86799500 -1.63246600<br>H -0.91411300 1.58655700 -0.42762100<br>H 0.47225400 -2.45257000 -0.04999300<br>H -2.15830700 0.33499300 -2.07849100<br>H 3.51700800 1.84066000 0.05481100<br>H 2.41116600 -3.50876000 -0.68177200<br>H 2.22749100 -3.39711800 1.09467700<br>H 3.84318700 -3.59258500 0.37239700<br>H -0.10855400 3.56508800 0.52231300<br>H 0.05479100 3.52260700 -1.25997400<br>H 1.10743800 4.58376800 -0.29074400<br>H -4.67921000 -0.27700600 -1.00617100<br>H -4.32158200 -0.74199000 -2.66675600<br>H -4.13347000 -1.91531100 -1.34591100<br>C -1.76628000 -1.00875800 -0.35228800<br>O -2.46088700 -0.86093800 0.95089400<br>H -1.74461600 -2.10077400 -0.42748800<br>N -2.83827800 0.42356200 1.17081500<br>O -3.35585700 0.54527500 2.21939200	• Electronic Energy<br>= -858.199591<br>• Zero Point Energy Correction<br>= 0.243173<br>• Thermal Correction to Energy<br>= 0.261473<br>• Thermal Correction to Enthalpy<br>= 0.262417<br>• Thermal Correction to Free Energy<br>= 0.195110

Name	HPns RAF C-2a···NOO$^{\bullet}$ (Model 1) Product
Cartesian Coordinate:	Thermochemical Values:
O -3.29725900 -1.60050300 0.31799900<br>O -1.52704300 2.64939500 -0.18368400<br>O -3.54778800 1.03607500 0.27194200<br>C 0.20776800 -0.61724300 -0.40743500<br>C -2.16974400 -0.89535600 0.07249100<br>C -1.21989100 1.32431800 -0.19798500<br>C 0.03424200 0.79111800 -0.42351100<br>C -0.91876200 -1.44316300 -0.15044400<br>C -2.32959000 0.50054100 0.05044300<br>C 1.46462800 -1.21583300 -0.63328100<br>C -3.18885300 -3.01445100 0.33354000<br>C -0.47042100 3.57388500 -0.40447300	• Electronic Energy<br>= -858.219099<br>• Zero Point Energy Correction<br>= 0.244262<br>• Thermal Correction to Energy<br>= 0.261939<br>• Thermal Correction to Enthalpy

C 3.87303000 -1.33385000 -1.34039600<br>H 0.88148500 1.44050200 -0.59520700<br>H -0.77695200 -2.51580700 -0.13589200<br>H 1.54258900 -2.29642700 -0.58197000<br>H -3.45827700 1.99793500 0.22639100<br>H -2.50707900 -3.35009700 1.12054300<br>H -2.84962800 -3.39404800 -0.63486100<br>H -4.18968100 -3.38910800 0.53850600<br>H -0.02667900 3.42602300 -1.39253600<br>H 0.29761200 3.47617000 0.36720600<br>H -0.91900800 4.56341800 -0.35042700<br>H 3.62318100 -1.87421400 -2.25579200<br>H 4.76431600 -0.73152800 -1.52297300<br>H 4.08997000 -2.06235400 -0.55597300<br>C 2.70678000 -0.45011000 -0.94197900<br>O 3.10164800 0.41957000 0.18003600<br>H 2.51854300 0.29439500 -1.72025800<br>N 3.25273600 -0.29726500 1.32368000<br>O 3.55860900 0.39257000 2.22441600	= 0.262883<br>• Thermal Correction to Free Energy<br>= 0.197115

Name	HPns RAF C-1···NOO• (Model 2) Product
Cartesian Coordinate:	Thermochemical Values:
O 1.69311200 -2.18428500 -0.36801400<br>O 0.85758400 2.39571600 -0.36955200<br>O 2.13144200 0.25019600 -1.46571300<br>C -1.54864600 -0.40891200 -0.09085600<br>C 0.71979600 -1.26244300 -0.31489300<br>C 0.25647800 1.19199600 -0.28741600<br>C -1.06373800 0.93702200 -0.14914600<br>C -0.61403800 -1.48114200 -0.17980300<br>C -2.94605200 -0.72232800 0.05180900<br>C -3.97212300 0.14979900 0.15578900<br>C 1.30389500 -3.54728400 -0.25916300<br>C 0.02783900 3.55060300 -0.30719400<br>C -5.40076700 -0.26138100 0.30081900<br>H -1.76265800 1.76091000 -0.09487300<br>H -0.99235400 -2.49454100 -0.12700700<br>H -3.18238200 -1.78481100 0.07730900<br>H -3.78003200 1.21934500 0.13747700<br>H 2.46138300 1.15991900 -1.48302100<br>H 0.64450400 -3.82700600 -1.08515900<br>H 0.80369900 -3.72881400 0.69614600<br>H 2.22242000 -4.12708200 -0.31011400<br>H -0.50413400 3.58628600 0.64692500<br>H -0.68550700 3.55394800 -1.13542800<br>H 0.69469200 4.40543200 -0.39006400<br>H -5.82853400 0.13608700 1.22696500<br>H -6.00639500 0.13731600 -0.51956500<br>H -5.50484500 -1.34774200 0.31066400<br>C 1.28988500 0.11345900 -0.39259100<br>N 2.21094700 0.29565000 0.92490500<br>O 1.70613200 0.00109600 1.98188400<br>O 3.31527200 0.75288500 0.77280400	• Electronic Energy<br>= -858.204759<br>• Zero Point Energy Correction<br>= 0.245058<br>• Thermal Correction to Energy<br>= 0.262646<br>• Thermal Correction to Enthalpy<br>= 0.263591<br>• Thermal Correction to Free Energy<br>= 0.199002

Name	HPns RAF C-3···NOO• (Model 2) Product
Cartesian Coordinate:	Thermochemical Values:
O 3.05684400 -1.48927700 0.08942200<br>O 0.77041100 2.55859000 -0.23500600<br>O 3.04948800 1.13292000 -0.06469100<br>C -0.57064000 -0.96240100 -0.29120400<br>C 1.84984300 -0.92986100 -0.05297100<br>C 0.69560900 1.19422100 -0.25791100<br>C 0.64584100 -1.61840800 -0.16103400<br>C 1.85448200 0.50817600 -0.12345100<br>C -1.80081900 -1.70369600 -0.41542400<br>C -3.03981700 -1.18211400 -0.50585000<br>C 3.12390400 -2.90768100 0.17852500<br>C 0.02884300 3.24957700 -1.24667200<br>C -4.28353600 -2.00334200 -0.61566000<br>H 0.64201000 -2.70143200 -0.15655600<br>H -1.69578100 -2.78704700 -0.41666300<br>H -3.18163500 -0.10332400 -0.50248200<br>H 2.87795600 2.08495600 -0.07930700<br>H 2.75852600 -3.37244700 -0.74080500<br>H 2.54898100 -3.26919900 1.03500500	• Electronic Energy<br>= -858.198418<br>• Zero Point Energy Correction<br>= 0.245713<br>• Thermal Correction to Energy<br>= 0.263288<br>• Thermal Correction to Enthalpy<br>= 0.264232<br>• Thermal Correction to Free Energy<br>= 0.199817

H 4.17572300 -3.14714900 0.31559600<br>H -1.04242600 3.20858500 -1.03955600<br>H 0.24286400 2.82708000 -2.23216000<br>H 0.36100300 4.28525900 -1.21096100<br>H -4.96227500 -1.79066200 0.21625600<br>H -4.82845000 -1.76463700 -1.53446000<br>H -4.05761600 -3.07110800 -0.61348000<br>C -0.62880200 0.52724500 -0.25857100<br>H -1.30489300 0.93832000 -1.01280600<br>N -1.34893400 0.99889800 1.07496700<br>O -1.04377800 0.45411000 2.10603100<br>O -2.12557600 1.92368400 0.97286400	

Name	HPns RAF C-4···NOO• (Model 2) Product
Cartesian Coordinate:	Thermochemical Values:
O 2.44451300 -1.92206300 -0.56341400<br>O 1.53336000 2.59745400 -0.14065000<br>O 3.19813100 0.60851800 -0.47277500<br>C 1.46856200 -1.03157500 -0.29676900<br>C 0.95645700 1.37233600 -0.07802500<br>C -0.35922600 1.11848500 0.12845200<br>C 0.15912100 -1.33161400 -0.08509500<br>C 1.90605700 0.32591000 -0.27408200<br>C -2.14595000 -0.55203000 -0.51541500<br>C -2.73635300 0.28352200 -1.36435700<br>C 2.07753000 -3.29369100 -0.58768400<br>C 0.70104700 3.72901100 0.08681800<br>C -3.99278200 -0.03540600 -2.11159100<br>H -1.07008000 1.91683000 0.28355300<br>H -0.19990900 -2.35019300 -0.11418900<br>H -2.56668900 -1.53833400 -0.33267100<br>H -2.29074000 1.25671300 -1.55637100<br>H 3.30338500 1.57095500 -0.43545600<br>H 1.34606700 -3.48808400 -1.37724300<br>H 1.67144500 -3.60613700 0.37854900<br>H 2.99230400 -3.84451800 -0.79517800<br>H 0.24877600 3.67883100 1.08038600<br>H -0.07797700 3.79232700 -0.67725500<br>H 1.35061000 4.59882000 0.02068900<br>H -4.76996100 0.70041600 -1.88607700<br>H -3.81814800 0.00706400 -3.19063900<br>H -4.36848400 -1.02792300 -1.85794900<br>C -0.85002900 -0.27616800 0.21745700<br>N -1.28849500 -0.53187700 1.77138500<br>O -1.32297300 0.40943000 2.52630000<br>O -1.58274700 -1.66787800 2.06827800	• Electronic Energy = -858.189451<br>• Zero Point Energy Correction = 0.244967<br>• Thermal Correction to Energy = 0.262671<br>• Thermal Correction to Enthalpy = 0.263615<br>• Thermal Correction to Free Energy = 0.197916

Name	HPns RAF C-1a···NOO• (Model 2) Product
Cartesian Coordinate:	Thermochemical Values:
O 1.72242500 2.64729100 0.02104100<br>O 2.91860900 -1.82572800 -0.08322500<br>O 3.54794300 0.72775300 0.04155800<br>C -0.43998700 -0.32402200 -0.22452700<br>C 1.26412000 1.37901600 -0.05803300<br>C 1.86760900 -0.96260400 -0.11056600<br>C 0.52604100 -1.32508100 -0.19611400<br>C -0.08008000 1.02167100 -0.15235900<br>C 2.24367000 0.37974100 -0.04241600<br>C -2.67907100 -0.02757400 -1.37039800<br>C 0.75892500 3.68920600 0.03698400<br>C 2.63217000 -3.21705900 -0.12945500<br>C -4.09430800 -0.40852800 -1.61192000<br>H 0.23238000 -2.36565200 -0.24008100<br>H -0.84944600 1.78305800 -0.15561500<br>H -2.25450500 0.84311800 -1.85238200<br>H 4.06892200 -0.08625600 0.05151300<br>H 0.08757100 3.59177500 0.89506000<br>H 0.17610800 3.70192500 -0.88876600<br>H 1.32284300 4.61582200 0.12149900<br>H 2.11947300 -3.47706200 -1.05939100<br>H 2.02462900 -3.51672900 0.72851600<br>H 3.59365500 -3.72431100 -0.08997000<br>H -4.76886800 0.12999500 -0.93083600<br>H -4.40406600 -0.16394100 -2.62955200<br>H -4.25492300 -1.47759700 -1.44299400	• Electronic Energy = -858.208155<br>• Zero Point Energy Correction = 0.244953<br>• Thermal Correction to Energy = 0.262909<br>• Thermal Correction to Enthalpy = 0.263853<br>• Thermal Correction to Free Energy = 0.196511

C -1.89574900 -0.70708800 -0.31693800<br>H -2.00284600 -1.79269900 -0.37096700<br>N -2.51679500 -0.40055200 1.08592800<br>O -3.01571200 0.68862300 1.25993600<br>O -2.43494100 -1.26839700 1.92530400	

Name	HPns RAF C-2a···NOO• (Model 2) Product
Cartesian Coordinate:	Thermochemical Values:
O 3.33354000 -1.59198500 -0.22463100<br>O 1.51079100 2.64762000 0.15653300<br>O 3.56324700 1.04445100 -0.17215400<br>C -0.21288400 -0.62851100 0.29946000<br>C 2.19025900 -0.89498700 -0.04351300<br>C 1.21211300 1.32175300 0.16295900<br>C -0.04815500 0.78212100 0.32196000<br>C 0.93282800 -1.44878200 0.10885400<br>C 2.33976700 0.50325300 -0.01783000<br>C -1.46954500 -1.24450100 0.45280600<br>C 3.23450500 -3.00695400 -0.25590700<br>C 0.43242900 3.56735500 0.27375900<br>C -3.86406200 -1.39657500 1.19623900<br>H -0.90377100 1.43127700 0.44491300<br>H 0.79753700 -2.52193900 0.08553500<br>H -1.53513300 -2.32397300 0.37010600<br>H 3.46641000 2.00653400 -0.14256900<br>H 2.59925900 -3.33988300 -1.08186800<br>H 2.84487100 -3.39507900 0.68971700<br>H 4.24734300 -3.37416400 -0.40797100<br>H -0.07936800 3.44425000 1.23182200<br>H -0.27746700 3.43750000 -0.54744100<br>H 0.87546400 4.55940100 0.22187000<br>H -3.58490800 -1.75603800 2.18802500<br>H -4.80009400 -0.84119000 1.27686500<br>H -4.01065100 -2.25364300 0.53814300<br>C -2.74542300 -0.51194400 0.67592100<br>H -2.61515100 0.37246800 1.29900400<br>N -3.20738400 0.08609300 -0.66936500<br>O -3.57571900 -0.67980900 -1.53110600<br>O -3.15481400 1.29203000 -0.79474700	• Electronic Energy = -858.227477<br>• Zero Point Energy Correction = 0.246368<br>• Thermal Correction to Energy = 0.263615<br>• Thermal Correction to Enthalpy = 0.264559<br>• Thermal Correction to Free Energy = 0.199080

Name	HPns RAF C-2a···NOO• (Model 2) Transition States
Cartesian Coordinate:	Thermochemical Values:
O -3.17012100 -1.77596000 0.30984700<br>O -1.71288700 2.59340000 -0.16281200<br>O -3.60299200 0.83840500 0.30858900<br>C 0.22639000 -0.53414800 -0.48173900<br>C -2.10522700 -0.98670200 0.04729700<br>C -1.31679000 1.29362700 -0.20834300<br>C -0.03520500 0.84849800 -0.47576900<br>C -0.81831000 -1.43862700 -0.21477700<br>C -2.35907200 0.39110600 0.05160600<br>C 1.53897400 -1.07320500 -0.74461800<br>C -2.95326500 -3.17816200 0.32237900<br>C -0.72404600 3.59262600 -0.37470600<br>C 3.94524400 -1.03056900 -1.45652600<br>H 0.75843400 1.55839300 -0.65877600<br>H -0.60051500 -2.49869100 -0.21739800<br>H 1.63288000 -2.15447400 -0.66873000<br>H -3.58019300 1.80519100 0.28467500<br>H -2.22953300 -3.45824800 1.09318400<br>H -2.60981700 -3.53269800 -0.65388400<br>H -3.91736800 -3.62749400 0.55127000<br>H -0.29383600 3.50563300 -1.37606400<br>H 0.06454300 3.52162200 0.37900300<br>H -1.23586900 4.54773100 -0.27984300<br>H 3.97712700 -1.07592800 -2.54993200<br>H 4.82372100 -0.47504700 -1.12496300<br>H 4.00613400 -2.04849100 -1.06831100<br>C 2.67956100 -0.35903500 -1.02409900<br>H 2.59656900 0.70159600 -1.24245300<br>O 4.10153800 -0.52027400 1.50259800<br>N 3.25634800 0.16619300 0.99619500<br>O 2.55243400 0.97881700 1.53110700	• Electronic Energy = -858.203587<br>• Zero Point Energy Correction = 0.243005<br>• Thermal Correction to Energy = 0.260857<br>• Thermal Correction to Enthalpy = 0.261801<br>• Thermal Correction to Free Energy = 0.195174

Name	HPns RAF C-2a···NOO$^{\bullet}$ (Model 2) Reactant Complex
Cartesian Coordinate:	Thermochemical Values:
O -2.83118300 -1.79940900 0.68526000<br>O -1.50445800 2.57001800 -0.09048200<br>O -3.27560900 0.81782800 0.76058000<br>C 0.35461900 -0.55861800 -0.72309100<br>C -1.83990200 -1.00270300 0.22343600<br>C -1.12478200 1.26556900 -0.18773500<br>C 0.08963500 0.81673400 -0.69004300<br>C -0.61900800 -1.45719600 -0.27587600<br>C -2.09456100 0.36924200 0.27267400<br>C 1.64352600 -1.09810300 -1.19264100<br>C -2.60638300 -3.19912300 0.66580300<br>C -0.56070900 3.56216200 -0.46700200<br>C 4.06957100 -1.01684700 -1.80740600<br>H 0.82014600 1.52230100 -1.06195800<br>H -0.40492800 -2.51766800 -0.30699700<br>H 1.64700300 -2.16557900 -1.40800800<br>H -3.25311900 1.78351200 0.73438800<br>H -1.74902300 -3.47054600 1.28887200<br>H -2.45001400 -3.56076200 -0.35482400<br>H -3.50717100 -3.65230600 1.07499100<br>H -0.30740700 3.47716800 -1.52702000<br>H 0.34590500 3.48551700 0.14023200<br>H -1.04147100 4.52100200 -0.28457700<br>H 4.41944500 -0.52175700 -2.71862700<br>H 4.85590500 -0.88920400 -1.05712000<br>H 3.95887500 -2.08278900 -2.01545400<br>C 2.78236700 -0.41611900 -1.33595900<br>H 2.80932800 0.64413700 -1.09266700<br>O 0.27723900 0.13918500 2.60277100<br>N 1.28454300 0.12385900 1.98630200<br>O 2.19031800 0.87725600 1.86546800	• Electronic Energy = -858.211331<br>• Zero Point Energy Correction = 0.242840<br>• Thermal Correction to Energy = 0.262272<br>• Thermal Correction to Enthalpy = 0.263216<br>• Thermal Correction to Free Energy = 0.191451

Sampai Sini

Name	HPs (4-propylsyringol)
Cartesian Coordinate:	Thermochemical Values:
O -2.12549500 -1.93054900 0.17688900<br>O -1.17919600 2.60481400 0.08908700<br>O -2.85918100 0.58303600 0.48196000<br>C 2.49178200 -0.54992500 -1.00676400<br>C 1.05667300 -0.24390400 -0.64775000<br>C 3.30679000 -1.07616400 0.18487500<br>C 0.63263200 1.07310300 -0.47890300<br>C 0.15849600 -1.29098800 -0.43448200<br>C -1.15020600 -1.00660200 -0.05620400<br>C -0.68202200 1.35827900 -0.09782600<br>C -1.57946000 0.31140500 0.11562000<br>C 3.39390200 -0.07721800 1.33458800<br>C -1.78310200 -3.30068400 0.03442500<br>C -0.29612800 3.69551600 -0.10775100<br>H 2.51552800 -1.29140500 -1.81109600<br>H 2.96788200 0.35710800 -1.39181800<br>H 2.85916800 -2.00977900 0.54144500<br>H 4.31343300 -1.32381200 -0.16579500<br>H 1.33860500 1.87708500 -0.64356500<br>H 0.48533900 -2.31524700 -0.56730200<br>H 3.82361900 0.87067500 0.99557500<br>H 2.40576800 0.13442200 1.75105500<br>H 4.02288000 -0.46000000 2.14175900<br>H -3.31743300 -0.26185200 0.57728800<br>H -0.98994600 -3.57899900 0.73369900<br>H -1.46892800 -3.51938200 -0.98977900<br>H -2.68507400 -3.86365600 0.26543800<br>H 0.07168200 3.72740800 -1.13768000<br>H 0.55057800 3.64958400 0.58374500<br>H -0.87826400 4.59254000 0.09457300	• Electronic Energy = -654.373880<br>• Zero Point Energy Correction = 0.256607<br>• Thermal Correction to Energy = 0.271424<br>• Thermal Correction to Enthalpy = 0.272368<br>• Thermal Correction to Free Energy = 0.214524

Name	HPs 1-OH radical
Cartesian Coordinate:	Thermochemical Values:
O -2.00517500 -2.08862000 0.19267100<br>O -1.33467900 2.54796600 0.10242800<br>O -2.86869400 0.41169400 0.50288900<br>C 2.45217500 -0.40330300 -1.01894800<br>C 1.01313100 -0.17096700 -0.64658300<br>C 3.29142100 -0.90692600 0.16891100<br>C 0.52980400 1.13520400 -0.47982100<br>C 0.17986200 -1.28266400 -0.42228600<br>C -1.13209700 -1.10505500 -0.03638700<br>C -0.77910900 1.35042600 -0.08878000<br>C -1.69466900 0.23455200 0.15667000<br>C 3.32924800 0.08087000 1.33054300<br>C -1.55474800 -3.42636400 0.02813500<br>C -0.52528300 3.69827700 -0.10344800<br>H 2.49974900 -1.14047000 -1.82634400<br>H 2.88609700 0.52672400 -1.39621700<br>H 2.89133600 -1.86650000 0.51128300<br>H 4.30691600 -1.09808200 -0.18955200<br>H 1.20176000 1.96499500 -0.65889800<br>H 0.58844400 -2.27571300 -0.56287300<br>H 3.70636600 1.05446800 1.00254100<br>H 2.33368000 0.23353400 1.75549900<br>H 3.98166100 -0.27908400 2.12923800<br>H -0.73350200 -3.64795000 0.71511600<br>H -1.23575600 -3.60567800 -1.00227600<br>H -2.40763000 -4.05947000 0.26197000<br>H -0.17474700 3.74736100 -1.13781800<br>H 0.32895200 3.69982400 0.57902900<br>H -1.16334600 4.55343100 0.10737400	• Electronic Energy = -653.738697<br>• Zero Point Energy Correction = 0.244141<br>• Thermal Correction to Energy = 0.258527<br>• Thermal Correction to Enthalpy = 0.259471<br>• Thermal Correction to Free Energy = 0.202026

Name	HPs 1a-CH radical
Cartesian Coordinate:	Thermochemical Values:
O -2.92176000 -0.94827100 -0.11348300<br>O -0.07729700 2.70617900 0.00607400<br>O -2.50087900 1.64754200 -0.18512400<br>C 1.86924700 -1.77899700 0.39802100<br>C 0.77431300 -0.89940000 0.25069300<br>C 3.29833300 -1.34763900 0.47651800<br>C 0.94342700 0.51012400 0.20661400<br>C -0.54117500 -1.42555000 0.14243800<br>C -1.61664100 -0.57193800 -0.00009100<br>C -0.14679500 1.35487900 0.06188100<br>C -1.43960500 0.81982100 -0.04206500<br>C 3.90346700 -1.06830300 -0.90998300<br>C -3.20900700 -2.33873800 -0.10807200<br>C 1.20756100 3.29739900 0.09518300<br>H 1.65627900 -2.84331000 0.39487100<br>H 3.39407600 -0.44973900 1.09587900<br>H 3.88322800 -2.12935500 0.96766200<br>H 1.93803700 0.92606600 0.28337000<br>H -0.68254700 -2.49814400 0.17476100<br>H 3.86545600 -1.96458400 -1.53379500<br>H 3.34898400 -0.27949900 -1.42414700<br>H 4.94741400 -0.75608600 -0.82387400<br>H -3.29288200 1.09657900 -0.24334600<br>H -2.89970300 -2.79634800 0.83549900<br>H -2.71464700 -2.84063500 -0.94422600<br>H -4.28805500 -2.42538300 -0.21783300<br>H 1.85067300 2.97299400 -0.72853600<br>H 1.68636600 3.06217500 1.05033100<br>H 1.04804300 4.37172300 0.02682200	• Electronic Energy = -653.725499<br>• Zero Point Energy Correction = 0.242869<br>• Thermal Correction to Energy = 0.257675<br>• Thermal Correction to Enthalpy = 0.258620<br>• Thermal Correction to Free Energy = 0.200691

Name	HPs 2'-CH_3 radical
Cartesian Coordinate:	Thermochemical Values:
O -1.94764800 -2.18532200 -0.01761800<br>O -1.43364700 2.43309800 0.13558300<br>O -2.92873300 0.26037800 0.40451700<br>C 2.53016100 -0.32037100 -1.02717000<br>C 1.06940100 -0.16576400 -0.67572100	• Electronic Energy = -653.706857<br>• Zero Point Energy Correction

C 3.37989400 -0.80263800 0.15875600<br>C 0.51872800 1.09932100 -0.45590000<br>C 0.26735400 -1.29467200 -0.53019300<br>C -1.06446300 -1.14026800 -0.16199200<br>C -0.82104000 1.24893700 -0.09545200<br>C -1.62645400 0.11417800 0.05428100<br>C 3.36707000 0.16787900 1.33567400<br>C -1.45351100 -3.41484500 0.29501000<br>C -0.64973500 3.60816400 0.01230900<br>H 2.63054500 -1.03245000 -1.85157700<br>H 2.92015800 0.63895900 -1.38086300<br>H 3.01547500 -1.78257400 0.48451400<br>H 4.40745700 -0.94824400 -0.18807800<br>H 1.14866500 1.97223100 -0.57127900<br>H 0.66357300 -2.28573300 -0.72138300<br>H 3.71518200 1.15875300 1.02737400<br>H 2.36033900 0.28038100 1.74626800<br>H 4.01949700 -0.18043600 2.13978300<br>H -3.32208100 -0.61944000 0.46408600<br>H -0.56527800 -3.45527400 0.91468600<br>H -2.21922500 -4.17500200 0.34108100<br>H -0.26342000 3.72109500 -1.00495200<br>H 0.18151700 3.60535300 0.72369600<br>H -1.31660200 4.43763300 0.23917400	= 0.242303<br>• Thermal Correction to Energy = 0.257138<br>• Thermal Correction to Enthalpy = 0.258083<br>• Thermal Correction to Free Energy = 0.199701

Name	HPs HAT 1a-CH···NOO• Transition States Structure
Cartesian Coordinate:	Thermochemical Values:
O -1.21431500 2.66151000 0.60553700<br>O -2.85502300 -1.49027100 -0.60278400<br>O -3.17651700 0.97014700 0.27689400<br>C 1.97080600 -0.77360200 -0.93471900<br>C 0.63931100 -0.27435200 -0.65826600<br>C 3.09754700 0.19348600 -1.26417800<br>C -0.46357900 -1.15463100 -0.80650800<br>C 0.42694300 1.03985400 -0.16756700<br>C -0.84734000 1.44927400 0.13756700<br>C -1.74244800 -0.74840600 -0.49799300<br>C -1.94645200 0.57053100 -0.02255400<br>C 2.89652700 0.88567000 -2.61379900<br>C -0.18012400 3.60673100 0.86946700<br>C -2.69800500 -2.84186600 -1.01976000<br>H 1.95190900 -1.64740400 -1.59180200<br>H 3.20581500 0.93762000 -0.47009800<br>H 4.03352200 -0.37058400 -1.27790100<br>H -0.27449300 -2.15720100 -1.16713900<br>H 1.26598800 1.70780100 -0.03126100<br>H 2.81172500 0.14857900 -3.41678900<br>H 1.98353700 1.48713200 -2.61519600<br>H 3.73811300 1.54244400 -2.84604800<br>H -3.13373800 1.88224900 0.60509700<br>H 0.35231500 3.85628400 -0.05151000<br>H 0.51576400 3.21162300 1.61334700<br>H -0.67575500 4.49217100 1.25976000<br>H -2.05843900 -3.38995900 -0.32283900<br>H -2.28053100 -2.89133300 -2.02916400<br>H -3.69684600 -3.27174300 -1.01610000<br>H 2.26380300 -1.26039400 0.14131500<br>O 2.50761800 -1.68036600 1.52863100<br>N 1.45964900 -1.22008600 2.07151700<br>O 1.34581900 -1.39262200 3.26491500	• Electronic Energy = -859.394690<br>• Zero Point Energy Correction = 0.261919<br>• Thermal Correction to Energy = 0.280412<br>• Thermal Correction to Enthalpy = 0.281356<br>• Thermal Correction to Free Energy = 0.212905

Name	HPs HAT 1a-CH···NOO• Reactant Complex
Cartesian Coordinate:	Thermochemical Values:
O -2.01196500 1.62103000 -1.32427400<br>O -1.06819000 -2.77457700 -0.20620300<br>O -2.71530900 -0.91121500 -1.13824400<br>C 2.50081400 0.60722600 0.46868900<br>C 1.10489700 0.20246600 0.06033800<br>C 3.42809900 0.85366400 -0.73145700<br>C 0.69560000 -1.12835900 0.14712700<br>C 0.22394500 1.16553800 -0.43810000<br>C -1.05226800 0.78443900 -0.84004900<br>C -0.58536900 -1.51061900 -0.26009400	• Electronic Energy = -859.432959<br>• Zero Point Energy Correction = 0.266834<br>• Thermal Correction to Energy = 0.286482

C -1.46510700 -0.54818600 -0.75728800<br>C 3.63158900 -0.38834400 -1.59354200<br>C -1.70065500 3.00395000 -1.40502400<br>C -0.21364600 -3.77740200 0.31618900<br>H 2.93226500 -0.17501200 1.10128800<br>H 3.01507200 1.66311100 -1.34246400<br>H 4.39431200 1.20417000 -0.35593500<br>H 1.38600300 -1.86530300 0.53756000<br>H 0.53754300 2.20095900 -0.49764300<br>H 4.03288000 -1.21445300 -0.99814400<br>H 2.68993100 -0.72257400 -2.03644000<br>H 4.33320300 -0.19138500 -2.40753100<br>H -3.17785500 -0.10957600 -1.41503200<br>H -0.86100600 3.17538300 -2.08404200<br>H -1.46756400 3.40847800 -0.41608800<br>H -2.59041300 3.49193600 -1.79744200<br>H 0.06027000 -3.56298800 1.35364300<br>H 0.69162900 -3.88237600 -0.28936100<br>H -0.78212100 -4.70466000 0.27828300<br>H 2.44947300 1.51758100 1.07407000<br>O 0.33087200 1.59321700 2.95349700<br>N -0.28071900 0.64138700 2.60067500<br>O -1.42115500 0.43289100 2.37645000	• Thermal Correction to Enthalpy<br>= 0.287426<br>• Thermal Correction to Free Energy<br>= 0.214738

Name	HPs HAT 1a-CH···NOO$^{\bullet}$ Product Complex
Cartesian Coordinate:	Thermochemical Values:
O -0.32505400 2.75782600 -0.43352500<br>O -2.86631200 -1.08481200 -0.89088600<br>O -2.63788300 1.54753800 -0.72588900<br>C 1.96297200 -1.58858400 -0.41016200<br>C 0.81657700 -0.77001400 -0.51943000<br>C 3.36196800 -1.07446100 -0.28194700<br>C -0.46439500 -1.37099700 -0.67681300<br>C 0.89557600 0.64696800 -0.43267800<br>C -0.25969800 1.40099300 -0.50320500<br>C -1.61230300 -0.60148600 -0.74508900<br>C -1.51773900 0.79819600 -0.65995700<br>C 3.95561100 -0.66349000 -1.64045500<br>C 0.89052200 3.47078600 -0.25326400<br>C -3.01622400 -2.49312100 -0.96133800<br>H 1.82034200 -2.65932200 -0.52134600<br>H 3.39749100 -0.21671100 0.39834000<br>H 3.99027000 -1.85079800 0.16126600<br>H -0.52086400 -2.45019700 -0.73832300<br>H 1.85632400 1.12773900 -0.31144200<br>H 3.97615900 -1.51526400 -2.32428400<br>H 3.35609200 0.12380900 -2.10382300<br>H 4.97758200 -0.29525700 -1.52175200<br>H -2.37730200 2.47407700 -0.63312800<br>H 1.57180800 3.29474800 -1.09000500<br>H 1.37129600 3.18556100 0.68632400<br>H 0.62040300 4.52403300 -0.21914100<br>H -2.65339600 -2.97394500 -0.04807800<br>H -2.49071400 -2.90316700 -1.82881000<br>H -4.08376000 -2.67565800 -1.06649000<br>H 1.06044000 -1.18737600 1.68853800<br>O 0.88774300 -0.96885400 2.62365300<br>N -0.26181400 -0.22531600 2.58799000<br>O -0.57307900 0.12907900 3.65942700	• Electronic Energy<br>= -859.424570<br>• Zero Point Energy Correction<br>= 0.264761<br>• Thermal Correction to Energy<br>= 0.284697<br>• Thermal Correction to Enthalpy<br>= 0.285642<br>• Thermal Correction to Free Energy<br>= 0.212134

Name	HPs RAF C-4···NO$^{\bullet}$ Product
Cartesian Coordinate:	Thermochemical Values:
O -2.81276800 -0.88416500 0.14946400<br>O -0.14189700 2.89420600 0.10873400<br>O -2.50813500 1.71704000 0.17856000<br>C 2.04594500 -1.49925800 0.08185800<br>C 1.93513800 -1.81943800 1.57569100<br>C 0.98157700 0.78437800 -0.22947200<br>C -0.44976600 -1.27404500 -0.21054500<br>C -1.53276200 -0.47375500 -0.03545400<br>C -0.13348000 1.55566900 -0.05974100<br>C -1.41813700 0.94970400 0.00265900<br>C 1.87426200 -0.60213600 2.49567400<br>C -3.06423800 -2.28311100 0.11727800<br>C 1.11162100 3.55783600 0.06506200	• Electronic Energy<br>= -784.202758<br>• Zero Point Energy Correction<br>= 0.262664<br>• Thermal Correction to Energy<br>= 0.279858<br>• Thermal Correction to Enthalpy<br>= 0.280802

H 2.10200400 -2.43976000 -0.47609200<br>H 2.98077500 -0.96532900 -0.11510000<br>H 1.05414100 -2.44578800 1.74776000<br>H 2.80334700 -2.42948300 1.84341100<br>H 1.96970600 1.22388700 -0.26699900<br>H -0.53784700 -2.35267300 -0.23590700<br>H 2.70303200 0.08464300 2.29754100<br>H 0.94255000 -0.04693700 2.36654600<br>H 1.93835300 -0.91043700 3.54223200<br>H -3.27659900 1.13126300 0.23317600<br>H -2.52614100 -2.78921700 0.92300300<br>H -2.77258300 -2.70375300 -0.84851700<br>H -4.13583100 -2.40149000 0.26212700<br>H 1.60120200 3.40542900 -0.90104000<br>H 1.76658000 3.21130100 0.87002200<br>H 0.89598900 4.61545800 0.20191000<br>C 0.88675000 -0.68086700 -0.48690600<br>N 0.97684600 -0.66438700 -2.08464000<br>O 1.99179200 -1.08036500 -2.53028600	• Thermal Correction to Free Energy = 0.217156

Name	HPs RAF C-1⋯NOO$^•$ (Model 1) Product
Cartesian Coordinate:	Thermochemical Values:
O 1.17891200 -2.27366000 -0.77694400<br>O 0.96074300 2.39128100 -0.71519000<br>O 1.64732300 0.10375100 -1.93252200<br>C -2.74726400 -0.15409300 1.36589600<br>C -1.41076300 -0.07377700 0.68413900<br>C -3.87581500 -0.60220500 0.41939200<br>C -0.85219400 1.17354100 0.32844200<br>C -0.74079400 -1.27029200 0.31722000<br>C 0.45342600 -1.22684300 -0.33257000<br>C 0.34454800 1.26440400 -0.32892800<br>C -4.08361100 0.35560900 -0.74950600<br>C 0.64064700 -3.57531100 -0.58222600<br>C 0.29742700 3.62000200 -0.45507100<br>H -2.68700100 -0.85813600 2.20252200<br>H -3.00036400 0.82456000 1.78444700<br>H -3.65093000 -1.60452600 0.04034500<br>H -4.79927500 -0.68545000 1.00029600<br>H -1.39890900 2.07388700 0.58090600<br>H -1.20154900 -2.22276800 0.55307400<br>H -4.29478500 1.36790900 -0.39105800<br>H -3.19547200 0.40461000 -1.38513600<br>H -4.92373200 0.03949200 -1.37219800<br>H 2.03709100 -0.75908900 -2.13008900<br>H -0.32378900 -3.67096400 -1.08817800<br>H 0.52651700 -3.78727200 0.48392000<br>H 1.35682100 -4.26757600 -1.01905000<br>H 0.16022700 3.76755300 0.61991500<br>H -0.67165400 3.65135600 -0.96093000<br>H 0.94328900 4.40001800 -0.85176000<br>C 1.16993400 0.05499800 -0.63984500<br>O 2.42904500 0.09846900 0.17855400<br>N 2.15583300 0.07999500 1.50765800<br>O 3.14140700 0.10143800 2.14831600	• Electronic Energy = -859.415576<br>• Zero Point Energy Correction = 0.267282<br>• Thermal Correction to Energy = 0.285445<br>• Thermal Correction to Enthalpy = 0.286389<br>• Thermal Correction to Free Energy = 0.219976

Name	HPs RAF C-2⋯NOO$^•$ (Model 1) Product
Cartesian Coordinate:	Thermochemical Values:
O 1.70393200 -0.96844100 -1.59276900<br>O -0.47611300 2.86540600 -0.40834500<br>O 1.69809100 1.67154500 -1.35684400<br>C -2.21039800 -1.48809500 1.23006000<br>C -1.09849900 -0.66754100 0.61873900<br>C -3.14804900 -2.09544800 0.17429000<br>C -1.29444200 0.74904600 0.47228900<br>C 0.03553900 -1.26554400 0.18439100<br>C -0.35693700 1.53660300 -0.17919600<br>C 0.80371400 0.94742200 -0.67704700<br>C -3.85399400 -1.05371700 -0.68884300<br>C 2.04549700 -2.35426300 -1.61801200<br>C -1.67010500 3.49351900 0.03100900<br>H -1.77604400 -2.29373900 1.82705000<br>H -2.79302300 -0.85761200 1.90902600<br>H -2.56880900 -2.77070200 -0.46377500<br>H -3.89247500 -2.70841000 0.69118500	• Electronic Energy = -859.405997<br>• Zero Point Energy Correction = 0.266935<br>• Thermal Correction to Energy = 0.285197<br>• Thermal Correction to Enthalpy = 0.286141<br>• Thermal Correction to Free Energy = 0.219579

H -2.20518400 1.18563100 0.85889600<br>H 0.19876600 -2.32787100 0.32885500<br>H -4.42109600 -0.35157100 -0.06974100<br>H -3.14076100 -0.47722700 -1.28367000<br>H -4.55411200 -1.53024400 -1.37896300<br>H 2.35983900 1.06766900 -1.72711800<br>H 1.14761500 -2.97084700 -1.69709400<br>H 2.62094800 -2.63348800 -0.73327700<br>H 2.65761800 -2.49517200 -2.50678600<br>H -1.77629200 3.42400900 1.11748800<br>H -2.54719200 3.05578200 -0.45516500<br>H -1.58081300 4.53910600 -0.25653100<br>C 1.14362800 -0.48299000 -0.41644400<br>O 2.34097100 -0.50707200 0.51177400<br>N 2.02498000 -0.00115000 1.73442900<br>O 2.94817300 -0.03159000 2.46008400	

Name	HPs RAF C-3···$NOO^{\bullet}$ (Model 1) Product
Cartesian Coordinate:	Thermochemical Values:
O -0.07623400 -2.48726100 -0.94293000<br>O 3.20811200 0.63427000 0.13579700<br>O 2.49200800 -1.75677900 -0.73191600<br>C -1.51683200 2.07794700 0.57938100<br>C -0.45016300 1.07643300 0.22798200<br>C -2.57486900 2.27905700 -0.51592400<br>C 0.88423300 1.36159000 0.34278400<br>C 0.19583500 -1.20114900 -0.56642400<br>C 1.88603700 0.41828900 0.03931100<br>C 1.50805700 -0.87539200 -0.42972400<br>C -1.96803600 2.68845600 -1.85432000<br>C -1.22550600 -2.68587900 -1.76392200<br>C 3.64259900 1.90358700 0.60461400<br>H -2.02389300 1.76233600 1.49998600<br>H -1.03410100 3.03511300 0.79715100<br>H -3.16498900 1.36436200 -0.63414600<br>H -3.27399400 3.04756800 -0.17516800<br>H 1.17103500 2.35231400 0.67461500<br>H -1.37445300 3.60144800 -1.74779100<br>H -1.30537700 1.91183500 -2.24786200<br>H -2.74396300 2.87664100 -2.59978300<br>H 2.06485400 -2.57996400 -1.00476000<br>H -1.17624800 -2.05691100 -2.65755200<br>H -2.14328300 -2.47981700 -1.20756500<br>H -1.21188200 -3.73408100 -2.05585500<br>H 3.27067200 2.09497900 1.61472500<br>H 3.31908500 2.70134800 -0.06948100<br>H 4.72935600 1.85930600 0.61951700<br>C -0.90938200 -0.28172700 -0.19759700<br>O -1.76376900 -0.90605000 0.85694900<br>H -1.65321500 -0.20891500 -0.99898800<br>N -1.05714800 -1.07430400 2.00917300<br>O -1.72029200 -1.55851700 2.84857300	• Electronic Energy<br>= -859.406548<br>• Zero Point Energy Correction<br>= 0.267883<br>• Thermal Correction to Energy<br>= 0.286182<br>• Thermal Correction to Enthalpy<br>= 0.287126<br>• Thermal Correction to Free Energy<br>= 0.220174

Name	HPs RAF C-4···$NOO^{\bullet}$ (Model 1) Product
Cartesian Coordinate:	Thermochemical Values:
O -2.51880400 1.47012800 0.21369000<br>O 2.05066500 2.02977100 -0.32343500<br>O -0.41309800 2.97279100 -0.17457800<br>C 0.38419200 -2.30131000 1.05306900<br>C 0.17530700 -1.88519600 2.51098900<br>C 1.26550000 -0.24087500 -0.11634000<br>C -1.20852700 -0.56543600 0.17737500<br>C -1.35450600 0.77983900 0.10413200<br>C 1.07742300 1.10259000 -0.19127400<br>C -0.23760700 1.64677900 -0.09707300<br>C 1.06759800 -0.75008500 3.01053800<br>C -3.71266500 0.71556500 0.37597800<br>C 3.38226000 1.55120600 -0.42463800<br>H -0.29688300 -3.12636200 0.82260400<br>H 1.40356800 -2.67387700 0.90781700<br>H -0.87401100 -1.61319600 2.66219400	• Electronic Energy<br>= -859.411165<br>• Zero Point Energy Correction<br>= 0.267515<br>• Thermal Correction to Energy<br>= 0.285579<br>• Thermal Correction to Enthalpy<br>= 0.286523<br>• Thermal Correction to Free Energy<br>= 0.220768

H 0.35278600 -2.77457300 3.12285200<br>H 2.25526700 -0.67584800 -0.16471100<br>H -2.05181600 -1.22533200 0.33834800<br>H 2.12063800 -0.94569000 2.78600600<br>H 0.80112300 0.20590100 2.55531900<br>H 0.97082600 -0.64181800 4.09369800<br>H -1.36068500 3.15326600 -0.08745700<br>H -3.68255600 0.14286300 1.30670000<br>H -3.85959100 0.04200100 -0.47227700<br>H -4.52449000 1.43828400 0.41712600<br>H 3.50080200 0.90582900 -1.29988600<br>H 3.67177300 1.00509800 0.47832000<br>H 4.01090400 2.43248800 -0.53374200<br>C 0.12980000 -1.20270600 0.00707500<br>O 0.09892200 -2.03658500 -1.25584700<br>N -0.12089700 -1.27043900 -2.34648600<br>O -0.14318900 -1.91837900 -3.33064100	

Name	HPs RAF C-1···NOO$^{\bullet}$ (Model 2) Product
Cartesian Coordinate:	Thermochemical Values:
O 1.22845200 -2.26533200 -0.42948900<br>O 0.99516200 2.38688200 -0.41706100<br>O 1.67356500 0.10745700 -1.70074600<br>C -2.95698500 -0.15649300 1.16749500<br>C -1.54689500 -0.07079800 0.65706700<br>C -3.95311700 -0.62108400 0.08929300<br>C -0.95143700 1.17766200 0.37367800<br>C -0.82665800 -1.26517800 0.39541100<br>C 0.43008800 -1.22311000 -0.12371900<br>C 0.31090500 1.26634000 -0.14395600<br>C -4.01877400 0.32660900 -1.10419200<br>C 0.71310000 -3.57365100 -0.20926300<br>C 0.35598000 3.62577800 -0.13800700<br>H -2.99608900 -0.85484400 2.01005300<br>H -3.26795600 0.82294700 1.54274500<br>H -3.67381800 -1.62412800 -0.24953200<br>H -4.94167100 -0.70831500 0.54984400<br>H -1.51495800 2.07985100 0.57779300<br>H -1.29745100 -2.21839600 0.60634800<br>H -4.27987900 1.33992400 -0.78348800<br>H -3.05842800 0.37772300 -1.62414900<br>H -4.77187400 -0.00065600 -1.82467000<br>H 2.19385700 -0.69673000 -1.83947900<br>H -0.18582300 -3.73633400 -0.80969500<br>H 0.48857500 -3.72242700 0.85001800<br>H 1.49496800 -4.26237100 -0.52026100<br>H 0.11750200 3.70407200 0.92622700<br>H -0.55484000 3.73238100 -0.73333000<br>H 1.06782200 4.40011100 -0.41398700<br>C 1.12702800 0.05816000 -0.44318000<br>N 2.39815700 0.10548600 0.58022400<br>O 2.15034800 0.31789500 1.74315300<br>O 3.48916700 -0.12647900 0.12292100	• Electronic Energy = -859.420785<br>• Zero Point Energy Correction = 0.268606<br>• Thermal Correction to Energy = 0.286588<br>• Thermal Correction to Enthalpy = 0.287532<br>• Thermal Correction to Free Energy = 0.221408

Name	HPs RAF C-3···NOO$^{\bullet}$ (Model 2) Product
Cartesian Coordinate:	Thermochemical Values:
O 0.00605300 2.35793300 -1.22475200<br>O -3.21372300 -0.69690500 0.19744400<br>O -2.55690600 1.63464400 -0.84867900<br>C 1.53539600 -2.07109500 0.53822000<br>C 0.44917900 -1.07350700 0.24046800<br>C 2.58571800 -2.20600300 -0.57417500<br>C -0.87967800 -1.38256700 0.37253600<br>C -0.25030500 1.18357300 -0.56961500<br>C -1.90126500 -0.46054300 0.07758400<br>C -1.55395100 0.82488000 -0.44223500<br>C 1.96988800 -2.53157000 -1.93140000<br>C 0.96091700 3.23282900 -0.61981600<br>C -3.62268900 -1.96111200 0.70652300<br>H 2.04588600 -1.79737000 1.47087000<br>H 1.06458100 -3.04280300 0.71198600<br>H 3.17678600 -1.28736500 -0.63689000<br>H 3.28356600 -2.99606700 -0.28398400	• Electronic Energy = -859.414152<br>• Zero Point Energy Correction = 0.269739<br>• Thermal Correction to Energy = 0.287528<br>• Thermal Correction to Enthalpy = 0.288472<br>• Thermal Correction to Free Energy = 0.223258

H -1.14412200 -2.38448100 0.68808000<br>H 1.36482500 -3.44161400 -1.87577900<br>H 1.31779500 -1.72494100 -2.27984600<br>H 2.74242100 -2.68671200 -2.68782100<br>H -2.15145200 2.44243000 -1.19292300<br>H 1.95502600 2.77653900 -0.59558800<br>H 0.64627100 3.50993600 0.38944000<br>H 1.00081200 4.11997200 -1.24871700<br>H -3.21762100 -2.12598900 1.70819800<br>H -3.31208800 -2.76870500 0.03869900<br>H -4.70861300 -1.92482700 0.75290100<br>C 0.85672900 0.31435400 -0.13307000<br>H 1.70488900 0.34181600 -0.82353900<br>N 1.54569000 0.90407400 1.18838900<br>O 0.83141000 1.36453600 2.04619000<br>O 2.74711200 0.77057000 1.27430700	
Name	HPs RAF C-4···NOO$^{\bullet}$ (Model 2) Product
Cartesian Coordinate:	Thermochemical Values:
O -2.97672100 -0.01325500 0.00967600<br>O 0.63008100 2.88439000 -0.23701600<br>O -1.96677500 2.39080200 -0.22153800<br>C 1.44543500 -1.77114200 0.88226000<br>C 0.96990300 -1.65098100 2.33229500<br>C 1.14800400 0.53355300 -0.14460100<br>C -0.79448700 -1.05395200 -0.02252400<br>C -1.62245900 0.01855100 -0.06137000<br>C 0.28300000 1.58135400 -0.19818900<br>C -1.12401400 1.35157700 -0.17243800<br>C 1.05379700 -0.25053800 2.93590900<br>C -3.59972200 -1.29086800 0.06952600<br>C 2.01836300 3.18014400 -0.26154500<br>H 1.33192900 -2.81390300 0.56532400<br>H 2.50723400 -1.52168500 0.80832900<br>H -0.05804100 -2.01797000 2.40894200<br>H 1.58604500 -2.33535100 2.92303900<br>H 2.21817300 0.68367800 -0.13953300<br>H -1.17683300 -2.06127000 0.07003000<br>H 2.04763100 0.18420100 2.79282500<br>H 0.32407500 0.42741600 2.48800200<br>H 0.85631800 -0.28998200 4.00991300<br>H -2.87065100 2.04440100 -0.18427800<br>H -3.30432500 -1.82039400 0.97901700<br>H -3.34142500 -1.88493300 -0.81065300<br>H -4.67097900 -1.10365100 0.08676200<br>H 2.49628700 2.73905200 -1.14094600<br>H 2.51089800 2.81957200 0.64641900<br>H 2.09310600 4.26443200 -0.31010300<br>C 0.67505500 -0.88022400 -0.12063200<br>N 1.14656200 -1.49824900 -1.53393400<br>O 2.31981300 -1.37965800 -1.81039900<br>O 0.33202300 -2.06111900 -2.22668500	• Electronic Energy<br>= -859.417067<br>• Zero Point Energy Correction<br>= 0.269476<br>• Thermal Correction to Energy<br>= 0.287179<br>• Thermal Correction to Enthalpy<br>= 0.288123<br>• Thermal Correction to Free Energy<br>= 0.222978

Name	NO$^{\bullet}$
Cartesian Coordinate:	Thermochemical Values:
N 0.00000000 0.00000000 -0.60702100<br>O 0.00000000 0.00000000 0.53114300	• Electronic Energy<br>= -129.878149<br>• Zero Point Energy Correction<br>= 0.004747<br>• Thermal Correction to Energy<br>= 0.007108<br>• Thermal Correction to Enthalpy<br>= 0.008052<br>• Thermal Correction to Free Energy<br>= -0.015224

Name	HNO
Cartesian Coordinate:	Thermochemical Values:
N 0.05740500 0.72360800 0.00000000<br>O 0.05740500 -0.52115800 0.00000000<br>H -0.86107700 -0.89599400 0.00000000	• Electronic Energy = -130.398828<br>• Zero Point Energy Correction = 0.014282<br>• Thermal Correction to Energy = 0.017126<br>• Thermal Correction to Enthalpy = 0.018071<br>• Thermal Correction to Free Energy = -0.006951

Name	$NOO^{\bullet}$
Cartesian Coordinate:	Thermochemical Values:
N 0.00000000 0.00000000 0.31576900<br>O 0.00000000 1.09061200 -0.13814900<br>O 0.00000000 -1.09061200 -0.13814900	• Electronic Energy = -205.050922<br>• Zero Point Energy Correction = 0.009183<br>• Thermal Correction to Energy = 0.012107<br>• Thermal Correction to Enthalpy = 0.013051<br>• Thermal Correction to Free Energy = -0.014133

Name	HNO_2
Cartesian Coordinate:	Thermochemical Values:
O -1.10442700 0.12759800 0.00000000<br>N 0.00000000 0.50340700 0.00000000<br>O 0.88448600 -0.55157800 0.00000000<br>H 1.75952300 -0.13201700 0.00000000	• Electronic Energy = -205.688876<br>• Zero Point Energy Correction = 0.020894<br>• Thermal Correction to Energy = 0.024062<br>• Thermal Correction to Enthalpy = 0.025006<br>• Thermal Correction to Free Energy = -0.003026

`
